# Resolving orbital pathways for intermolecular electron transfer

**DOI:** 10.1038/s41467-018-07263-1

**Published:** 2018-11-21

**Authors:** Cameron W. Kellett, Wesley B. Swords, Michael D. Turlington, Gerald J. Meyer, Curtis P. Berlinguette

**Affiliations:** 1 Department of Chemistry, 2036 Main Mall, University of British Columbia, Vancouver, BC V6T 1Z1 Canada; 2 Department of Chemistry, University of North Carolina at Chapel Hill, Murray Hall 2202B, Chapel Hill, NC 27599-3290 USA; 3 Department of Chemical and Biological Engineering, 2360 East Mall, University of British Columbia, Vancouver, BC V6T 1Z3 Canada; 4 Stewart Blusson Quantum Matter Institute, 2355 East Mall, University of British Columbia, Vancouver, BC V6T 1Z4 Canada

## Abstract

Over 60 years have passed since Taube deduced an orbital-mediated electron transfer mechanism between distinct metal complexes. This concept of an orbital pathway has been thoroughly explored for donor–acceptor pairs bridged by covalently bonded chemical residues, but an analogous pathway has not yet been conclusively demonstrated for formally outer-sphere systems that lack an intervening bridge. In our present study, we experimentally resolve at an atomic level the orbital interactions necessary for electron transfer through an explicit intermolecular bond. This finding was achieved using a homologous series of surface-immobilized ruthenium catalysts that bear different terminal substituents poised for reaction with redox active species in solution. This arrangement enabled the discovery that intermolecular chalcogen⋯iodide interactions can mediate electron transfer only when these interactions bring the donor and acceptor orbitals into direct contact. This result offers the most direct observation to date of an intermolecular orbital pathway for electron transfer.

## Abstract (web summary)

It is known that intermolecular interactions impact electron transfer rates, but the mechanisms involved are challenging to define experimentally. Here, the authors have developed a platform that enables atomic orbital resolution of electron transfer through an explicit intermolecular interaction.

## Introduction

Molecular photoredox catalysis is widely used for a range of applications, such as energy storage^1–3^, CO_2_ valorization^4,5^, and organic transformations^6–8^. Typically, light-induced excitation of a photocatalyst creates an energetic electron or hole that enables the transformation of a substrate to a useful product. The discovery and optimization of such catalysts requires the optimization of factors that govern intermolecular electron transfer (IET). In particular, the roles that intermolecular interactions have in mediating electron transfer are very challenging to define. During the last two decades, a body of literature has shown that electrostatic interactions^9,10^, hydrogen bonding^11–13^, and other non-bonding interactions^14–19^ can enhance IET rates, but interactions that are too strong may suppress reactivity by lowering the driving force for reaction^20^. This scenario provides the imperative to understand how weak intermolecular interactions can increase IET rates without compromising chemical reactivity.

At a fundamental level, the electron transfer kinetics of molecular donor–acceptor pairs is described by the Marcus equation (equation 1), which is depicted visually in Supplementary Fig. 1. This theory stipulates that the primary factors affecting the rate of electron transfer is the thermodynamic Gibbs free energy change for the reaction (Δ*G*°_ET_) and the reorganization energy (*λ*), whereas quantum mechanical effects are accounted for by an electronic coupling factor (*H*_DA_)^21–23^. While the chemical origins of Δ*G*°_ET_ and *λ* are well understood in terms of intuitive chemical principles, *H*_DA_ can only be rigorously defined in terms of the complete wave functions of the non-adiabatic donor–acceptor states before and after electron transfer (Ψ_1_ and Ψ_2_, respectively; equation 2)^24^. For formally outer-sphere electron transfer reactions where the electronic coupling is weak, *H*_DA_ can have a significant effect on electron transfer rates^25,26^. Furthermore, early studies on intermolecular self-exchange reactions have suggested that the degree of charge delocalization in conjugated donor or acceptor molecules can have a large impact on the magnitude of *H*_DA_^27,28^. Nonetheless, for intermolecular systems of even moderate complexity, the structural factors impacting *H*_DA_ are rarely considered. These difficulties stem from the fact that *H*_DA_ is fundamentally a function of the transition state, and therefore challenging to reliably quantify^25,26,29,30^. For donor–acceptor pairs that are covalently bridged, or otherwise bound together on sufficiently long timescales, *H*_DA_ can be inferred from the degree of charge delocalization within the donor–acceptor pairs, which can be measured using vibrational spectroscopy or EPR spectroscopy^31^. Under ideal conditions, *H*_DA_ can also be determined directly using Mulliken-Hush analysis of intervalence charge-transfer excitations in UV-vis-NIR spectroscopy^32–36^. All of these methods require that the donor and acceptor remain in a fixed geometry within the time frame of the experiment, and therefore these analyses are unsuitable for all but a handful of IET reactions that are of practical interest. Computational methods offer somewhat more flexibility with respect to the systems that can be investigated, however challenges remain in balancing reliability for these approaches versus computational cost^30,31,37,38^ and thus these methods are not easily accessible to those outside the advanced computational community. Consequently, for experimentalists attempting to design novel molecules, computational methods to predict *H*_DA_ are of limited utility in said design. As such, there is a need in this community to relate *H*_DA_ back to widely understood chemical principles and thereby create intuitive structure-property relationships that can be used to tune this important parameter.

1 $$k_{{\rm Marcus}} = \frac{{2\pi }}{\hbar }\left| {H_{{\rm DA}}} \right|^2\frac{1}{{\sqrt {4\pi \lambda {\rm RT}} }}e^{\left( {\frac{{ - (\lambda + {\mathrm{\Delta }}G^\circ _{{\rm ET}})^2}}{{4\lambda {\rm RT}}}} \right)}.$$

2 $$H_{{\rm DA}} = \left\langle {\Psi _1\left| {\hat H} \right|\Psi _2} \right\rangle.$$

The physical meaning of *H*_DA_ is best understood in the context of covalently bonded, inner-sphere donor–acceptor pairs. This understanding stems from Henry Taube’s 1953 experiment, where he demonstrated that electron transfer between cobalt(III) chloride and chromium(II) complexes was accompanied by quantitative chloride ligand transfer between the two metal centers (equation 3)^39^. The principal conclusion of this foundational study was that the chloride ligand forms a bridging intermolecular interaction between the two metal centers leading to an increased electron transfer rate compared to complexes that could not form an analogous bridged intermediate. Whereas this intermediate has never been observed, it is generally accepted that it creates an orbital pathway for electron transfer resulting in stronger electronic coupling between the metal centers^29,40^. The effects of these orbital pathways on the rate of electron transfer have been studied extensively for donor–acceptor pairs featuring conjugated covalent bonds or a bridging ligand^40–44^.

3 $$\left[ {{\mathrm{Co}}^{{\mathrm{III}}}{\mathrm{Cl(NH}}_{\mathrm{3}}{\mathrm{)}}_{\mathrm{5}}} \right]^{{\mathrm{2 + }}}{\mathrm{ + }}\left[ {{\mathrm{Cr}}^{{\mathrm{II}}}{\mathrm{(OH}}_{\mathrm{2}}{\mathrm{)}}_{\mathrm{6}}} \right]^{{\mathrm{2 + }}} \to \left[ {{\mathrm{Co}} \cdots {\mathrm{Cl}} \cdots {\mathrm{Cr}}} \right]^{{\mathrm{4 + ,\ddagger }}} \\ \to \left[ {{\mathrm{Co}}^{{\mathrm{II}}}{\mathrm{(NH}}_{\mathrm{3}}{\mathrm{)}}_{\mathrm{5}}{\mathrm{OH}}_{\mathrm{2}}} \right]^{{\mathrm{2 + }}}{\mathrm{ + }}\left[ {{\mathrm{Cr}}^{{\mathrm{III}}}{\mathrm{Cl(OH}}_{\mathrm{2}}{\mathrm{)}}_{\mathrm{5}}} \right]^{{\mathrm{2 + }}}.$$

Extending this concept of an orbital pathway to non-bonded, formally outer-sphere electron transfer reactions has proven more challenging, though considerable progress has been made in the field of electron transfer in proteins^45–48^ and through the use of donor–acceptor pairs rigidly constrained at a fixed distance^49–54^. In these systems, the donor and acceptor are bridged by amino acid residues, by coordinated solvent molecules, or by portions of the rigid backbone itself. In all of these bridged systems, whether covalently bonded or not, electron transfer is understood to be mediated by tunneling pathways, hopping pathways, or superexchange pathways through the bridging moieties^55,56^.

**Fig. 1:**
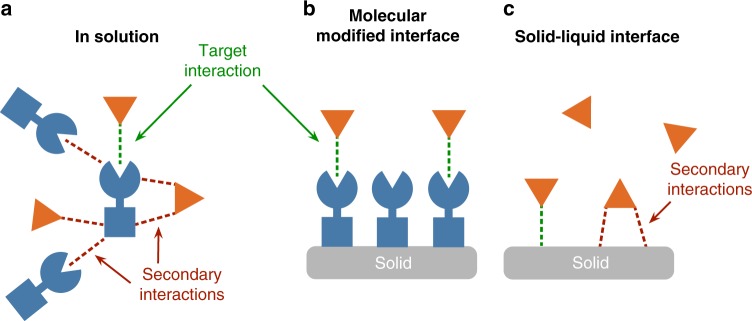
Intermolecular interactions in solution and at the interface. A schematic representation of a generic molecule (blue) interacting with a substrate in solution (orange). **a** In solution, many possible interaction sites on the molecule are exposed. **b** When the molecule is anchored to a solid surface, secondary interaction sites are blocked by adjacent molecules. **c** In the absence of a surface-anchored molecular species, the interactions between the substrate and the surface are difficult to conclusively define

The common theme among these studies of bridge-mediated electron transfer is that *H*_DA_ is a function of the electronic properties of the bridge as well as the donor and acceptor themselves. In many practically applicable redox catalysis systems; however, the donor and acceptor are not held in a fixed geometry and therefore the bridge is either ill-defined or non-existent. Unfortunately, this means that the knowledge gained from donor-bridge-acceptor systems is of limited utility for the design of molecular redox partners for practical applications. There is consequently a need to understand how the chemical and electronic structure of the isolated donor and acceptor molecules affect electronic coupling in IET processes. Computational studies over the last 10 years have demonstrated that, for bridge-free donor–acceptor pairs, the expression for *H*_DA_ presented in equation 2 can be reasonably simplified to rely only on the frontier molecular orbitals of the donor and acceptor^37,38,57,58^. Following from this description, intermolecular interactions could in principle be leveraged to encourage more interaction between these frontier molecular orbitals and encourage stronger electronic coupling. In practice, while halogen bonding and π-stacking between donor–acceptor pairs have recently been shown to enhance *H*_DA_^16–19^, the implied orbital pathways for electron transfer have not yet been resolved at the same level of detail possible for covalently bonded systems. This shortcoming stems from the fact that these studies were performed on donor and acceptor molecules in solution, where the interaction of interest is in competition with other secondary interactions (Fig. 1a). Consequently, such studies are typically limited to simple molecules bearing few functional groups in order to limit the number of competing interactions. As a result of this simplicity, modifications intended to perturb the electronic structure of these molecules will invariably change the thermodynamics for IET, complicating any investigation into the effects of electron delocalization. Moreover, any reliance upon dissolved species restricts the accessible time domains for studying IET processes.

We show herein a strategy for overcoming the shortcomings of solution studies by leveraging a solid–liquid interface. The interactions with a solution-phase substrate are poorly resolved on a bare solid surface (Fig. 1c), however, by affixing an appropriately designed molecular species to that surface in a common orientation, specific intermolecular interactions with the substrate can be targeted (Fig. 1b). Constraining the molecules to a surface precludes self-interaction and confines interactions with the soluble substrate to a specific site on the surface-anchored catalyst. We have used this approach previously to demonstrate how specific atoms on organic molecules anchored to TiO_2_ can impact IET rates with solution-phase nucleophiles^14,15^. Moreover, the use of photoactive redox catalysts with appropriate excited-state energies relative to the conduction band energy of the metal oxide allows for the generation of strongly oxidizing molecules on ultrafast timescales. This feature of these systems enables the use of pump-probe laser spectroscopy to investigate IET kinetics on timescales that are faster than can be achieved by traditional stop-flow techniques.

**Fig. 2:**
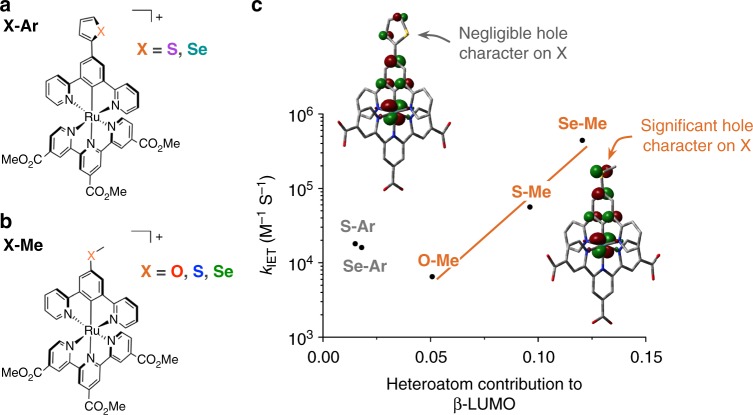
Ruthenium complexes to control β-LUMO delocalization. **a**, **b** Chemical structures of the **X-Ar** **a** and **X-Me** **b** compounds under investigation. The methyl ester groups are converted to acids to enable binding to titania. **c** Plot of the second-order IET rate constant (*k*_IET_) for the indicated compounds versus the heteroatom contribution to the β-LUMO (*c*_A,X_), overlaid with representative visualizations of the β-LUMO of **X-Ar** (left) and **X-Me** (right) plotted at an iso-value of 0.05

We have employed this strategy to demonstrate an explicit atomic orbital pathway for IET mediated by weak intermolecular interactions. To accomplish this, we synthesized two parallel series of cyclometalated ruthenium photoredox catalysts, **X-Ar** (Fig. 2a) and **X-Me** (Fig. 2b), substituted with chalcogen-containing functional groups para- to the ruthenium-carbon bond. With these compounds, we studied the readily accessible oxidation of iodide by the one-electron oxidized catalyst. In this reaction, no covalent bridge exists between the donor and acceptor, and therefore IET must be mediated by transient intermolecular contacts between the iodide donor and the catalyst molecules. It has been previously demonstrated that electron deficient chalcogens can interact with nucleophiles^59–61^, albeit quite weakly in most cases, and previous transient spectroscopic studies have demonstrated that interactions between iodide and sulfur or selenium affect the electron transfer rate constants with iodide^14,62–64^. For the **X-Ar** series, the positive hole, represented by the lowest unoccupied β-spin single electron molecular orbital (β-LUMO), of the oxidized catalyst is delocalized onto the heteroaromatic ring, but not onto the chalcogen atom itself (Fig. 2c). By contrast the positive hole for the **X-Me** series extends significantly onto the heteroatom (Fig. 2c). This difference in hole delocalization means that chalcogen⋯iodide interactions encourage direct overlap of the donor orbitals of iodide with the acceptor orbitals of the **X-Me** compounds, while analogous interactions with the **X-Ar** compounds have no effect on the overlap of orbitals relevant to IET. Moreover, because the extent to which the positive hole is delocalized onto the terminal chalcogen atom of the **X-Me** catalysts is a function of heteroatom identity, the degree to which these interactions encourage donor–acceptor orbital overlap varies across this series. As a result, the large rate enhancement of iodide oxidation observed for the **X-Me** series, but not the **X-Ar** series, can be attributed to this orbital pathway through the chalcogen⋯iodide interactions.

## Results

### Synthesis and preparation of surface-anchored samples

The ruthenium compounds under investigation were prepared in low to moderate yields from (trimethoxycarbonylterpyridine) ruthenium trichloride either by direct cyclometalation of the N^C^N ligand (**X-Ar**, Supplementary Fig. 2), or by transmetalation of an organomercuric chloride precursor (**X-Me**, Supplementary Fig. 3). NMR spectra of the final compounds and synthetic intermediates are presented in Supplementary Note 1. Solution phase optical and electronic characterization was carried out on the methyl ester complexes as tetrafluoroborate salts. To prepare surface-anchored samples for photophysical experiments, the compounds were saponified as described in the Supplementary Methods and reacted with mesoporous semiconductor films from saturated ethanol solutions. Depending on the experiment, mesoporous thin films made from TiO_2_ nanoparticles, SnO_2_–TiO_2_ core-shell nanoparticles, or indium-doped SnO_2_ nanoparticles (nano-ITO) were employed as indicated. All thin films were prepared on glass substrates coated with fluorine-doped tin oxide. The oxygen containing analogue of the **X-Ar** series, **O-Ar**, decomposed rapidly following saponification and was therefore not evaluated.

### Optical and redox properties

The absorption maxima, *λ*_max_, for each of the major bands for the **X-Me** and **X-Ar** series were determined by UV-vis spectroscopy and varied by less than 10 nm across all compounds when measured in acetonitrile (MeCN) solution (Supplementary Fig. 4a, Table 1). Upon saponification and adhesion to TiO_2_, the lowest energy metal-to-ligand charge-transfer (MLCT) transition of each catalyst was hypsochromically shifted ~20 nm relative to the methyl esters, with no other significant changes (Supplementary Fig. 4b, Table 1). The Ru^III/II^ redox couples for the compounds were determined by cyclic voltammetry in acetonitrile solution (Supplementary Fig. 4c, Table 1). All compounds displayed quasi-reversible redox couples with 75–80 mV peak separations. All Ru^III/II^ couples fall within the narrow electrochemical window of 0.84–0.88 V vs NHE with the exception of **O-Me**, which displayed a redox couple cathodically shifted ~60 mV.

**Table 1:** Optical and redox properties of ruthenium complexes in solution and immobilized on mesoporous metal oxide (MO_X_) thin films

Compound	*λ*_max, MeCN_ (nm)^a^	*ε*_MeCN_ (×10^4^ M^−1^ cm^−1^)^a^	*E*_1/2, MeCN_ (Ru^III/II^) (V)^b^	*λ*_max, MOx_ (nm)^c^	*E*_1/2, MOx_ (Ru^III/II^) (V)^d^
**O-Me**	589	1.04	0.78	570	0.73
**S-Me**	583	1.09	0.84	566	0.80
**Se-Me**	583	1.12	0.86	564	0.82
**S-Ar**	582	1.09	0.88	562	0.85
**Se-Ar**	582	1.19	0.88	561	0.84

^a^Measurements are reported in V vs NHE and were collected in acetonitrile solution

^b^Collected in 0.1 M (NBu_4_)BF_4_ acetonitrile solution and referenced to an external ferrocene/ferrocenium standard (0.630 V vs NHE)^75^

^c^Saponified catalysts on TiO_2_ thin films

^d^Measurements of the saponified catalysts are reported in NHE and were collected on nano-ITO thin films immersed in 0.5 M NaClO_4_ acetonitrile solution and *E*_1/2_ values determined by deconvolution of the resulting spectra into oxidized and reduced components (Supplementary Fig. 5b)

The optical and redox properties of the saponified catalysts anchored to metal oxide films were investigated through spectroelectrochemistry on nano-ITO (Supplementary Fig. 5, Table 1). The Ru^III/II^ couples were cathodically shifted ~40 mV versus the methyl esters in solution, though they remained in a relatively narrow window of 0.80–0.85 V vs NHE, except for **O-Me** which was still ~60 mV more cathodic than **S-Me**. Upon oxidation by one electron, the absorbance spectra recorded for each of the oxidized complexes featured a prominent new low-energy absorption band centered between 700 and 850 nm coupled with a bleach of the Ru^II^ MLCT band, consistent with the formation of a Ru^III^ complex. Sharp isosbestic points were observed for each of the compounds between 650 and 700 nm, indicating clean conversion of the Ru^II^ to the Ru^III^ complexes with no side reactions. Exchange of oxygen to sulfur and selenium in the **X-Me** catalysts resulted in an increasing bathochromic shift of the new absorption band by 95 nm (1690 cm^−1^) and 45 nm (810 cm^−1^), respectively. In contrast the exchange of selenium for sulfur in **X-Ar** results in a much more modest 25 nm (240 cm^−1^) bathochromic shift.

### Interfacial chemistry

**Fig. 3:**
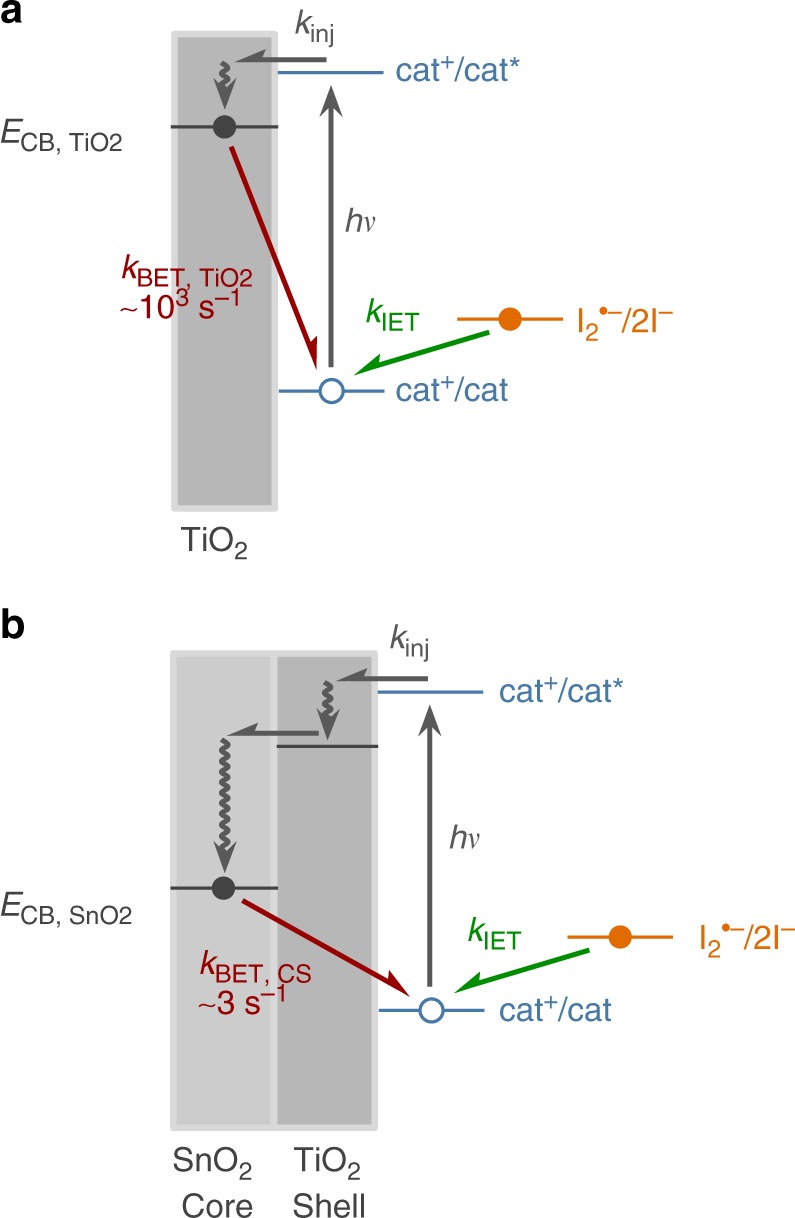
Electron transfer reactions at photoredox functionalized metal oxide interfaces. A schematic depicting the major electron transfer reactions that occur at functionalized TiO_2_ (**a**) or SnO_2_-TiO_2_ core-shell (**b**) interfaces: excitation of the catalyst by a photon (*hν*), electron injection into the semiconductor (*k*_inj_), back-electron transfer (*k*_BET_), and intermolecular electron transfer (*k*_IET_). The energy levels of the catalyst redox potentials in the ground and excited states (cat^+^/cat and cat^+^/cat*, respectively), the diiodide redox potential (I_2_^•–^/2I^–^), and the conduction bands of TiO_2_ and SnO_2_ (*E*_CB, TiO2_ and *E*_CB, SnO2_, respectively) are depicted on an approximate relative energy scale

Electron-transfer kinetics were studied at the interface of mesoporous TiO_2_ thin films functionalized with **X-Me** or **X-Ar** submerged in solutions containing either 0.5 M NaClO_4_/MeCN or 0.5 M NaI/MeCN. The critical electron transfer reactions that occur at photocatalyst-functionalized TiO_2_ surfaces are depicted in Fig. 3a. Following laser excitation of the films at 532 nm, electrons are injected from the excited photoredox catalyst into the TiO_2_ conduction band. This process results in the appearance of an optical signal consistent with oxidized **X-Me**^**•+**^ or **X-Ar**^**•+**^ (Supplementary Fig. 6). In the redox inert NaClO_4_ electrolyte, the injected electrons and oxidized catalysts on the surface recombine in a process called back-electron transfer (BET). In the presence of NaI electrolyte, the photo-oxidized catalyst instead oxidizes iodide through an IET process. To accurately resolve IET kinetics, it is necessary that the rate constant for IET (*k*_IET_) be much greater than that for BET (*k*_BET_). On pure TiO_2_ thin films, the BET rates were found to be too similar to the IET rates to enable accurate determination of the true second-order *k*_IET_, and TiO_2_ was therefore not a good substrate to fully analyze the iodide oxidation kinetics (Supplementary Fig. 7a, Table 2).

**Table 2:** Electron transfer kinetics and β-LUMO partition

Compound	*k*_BET, TiO2_ (×10^4^ s^−1^)^a^	*k*_BET, CS_ (s^−1^)^b,c^	*k*_IET_ (×10^4^ M^−1^ s^−1^)^b^	Heteroatom contribution to β-LUMO, *c*_A,X_^d^
**O-Me**	4.8	1.1	0.65	0.051
**S-Me**	2.2	2.7	5.6	0.096
**Se-Me**	0.44	1.9	44	0.121
**S-Ar**	0.080	3.0	1.8	0.015
**Se-Ar**	0.092	3.4	1.6	0.018

^a^Saponified catalysts adsorbed to TiO_2_ thin films

^b^Saponified catalysts anchored to SnO_2_–TiO_2_ core-shell thin films

^c^Modeled with the KWW function, β fixed to 0.3

^d^Calculated from DFT models using a Hirshfeld partition of the MO density and expressed as a fraction of unity

SnO_2_–TiO_2_ core-shell mesoporous thin films have been shown to drastically prolong BET reaction lifetimes^65^. In these films, an excited electron injected into the TiO_2_ shell quickly migrates into the more positive conduction band of the SnO_2_ core (Fig. 3b). From there, in order to undergo BET, the electron must tunnel through the shell to recombine with the oxidized catalyst on the surface. When **X-Me** and **X-Ar** were anchored to these core-shell thin films, *k*_BET_ was found to decrease by almost two orders of magnitude compared to the TiO_2_ thin film samples (Supplementary Fig. 7b), and was the same within experimental error for all catalyst compounds (Supplementary Fig. 8, Table 2). This allowed IET rate constants to be determined. The IET rates were studied in an analogous method to BET, but in the presence of increasing concentrations of iodide (Supplementary Fig. 9). From this data, we extracted the true second-order *k*_IET_ values (Supplementary Fig. 10, Table 2). In the **X-Me** series, an order of magnitude increase in *k*_IET_ was observed on exchanging S for O, and again when exchanging Se for S (Supplementary Fig. 10a). By contrast, in the **X-Ar** series, no significant change in *k*_IET_ was observed between **S-Ar** and **Se-Ar** (Supplementary Fig. 10b). In previous studies, catalyst structure has also been shown to have an effect on the rate of charge recombination between injected electrons and the oxidized form of the electrolyte^62,63^; however, the rate constant for this reaction was found to be the same for all compounds currently under investigation (Supplementary Fig. 11).

## Discussion

The compounds under investigation were designed to ensure optimal electronic and geometric properties for the systematic study of both intermolecular interactions and electron transfer (Fig. 2a, b). The highest occupied molecular orbital (HOMO) energy of the compounds is primarily a product of ruthenium centered molecular orbitals. Cyclometalation of the ruthenium shifts the HOMO higher in energy relative to the far more extensively studied ruthenium complexes bearing neutral ligands, but remains sufficiently positive to oxidize iodide^66,67^. The tricarboxyterpyridine ligand was chosen to ensure that the lowest unoccupied molecular orbital (LUMO) energy of the reduced Ru^II^ catalysts were appropriately positioned to inject electrons into TiO_2_ and to anchor the catalysts to the metal oxide substrates^67^. Previous studies have shown that incorporation of chalcogen atoms in positions with access to the metal oxide surface can encourage side reactions between the electrolyte and the semiconductor^62–64^, and therefore **X-Me** and **X-Ar** were substituted para- to the ruthenium center on the central ring to direct the chalcogen atoms away from the surface. The N^C^N cyclometalating motif was chosen such that, upon oxidation, the resulting positive hole will be delocalized to encompass the central ring and its substituents, ensuring that this hole will be exposed to the solution.

The HOMO and LUMO are important orbitals in chemical transformations like IET. As a result, the delocalization of these frontier orbitals within a molecular structure strongly impacts the properties and reactivity of that molecule. Upon substitution with either −XCH_3_ groups or heteroaromatic groups, the electronic properties of the Ru^II^ complexes were well conserved (Supplementary Fig. 4, Table 1) suggesting that the Ru^II^ center is relatively well insulated from any electronic effects of substitution on the ligand. This was necessary to ensure that the only factors that would affect the rate of IET would be the hole delocalization in the oxidized photocatalyst and the degree of iodide–chalcogen interaction. A very different picture of the frontier molecular orbitals emerges for the one-electron oxidized Ru^III^ complexes. The bathochromic shifts of the emergent Ru^III^ absorption band suggest considerably more heteroatom involvement in the frontier orbitals of the Ru^III^ compounds, particularly the **X-Me** series, compared to the Ru^II^ compounds. Indeed, computational models generated of these compounds describe the new transition as a ligand-to-metal charge-transfer (LMCT) band with significant contributions from the heteroatom for the **X-Me** series, or from the aryl ring (but not the heteroatom) for the **X-Ar** series (Supplementary Fig. 12, Supplementary Table 1). Furthermore, our DFT models of these Ru^III^ complexes showed dramatic differences in the degree of delocalization of the frontier molecular orbitals (Supplementary Table 2). Of particular relevance to this study is the lowest unoccupied β-spin single electron molecular orbital (β-LUMO), which is equivalent to the positive hole of the oxidized catalyst. The degree of heteroatom contribution to this frontier orbital (*c*_A,X_) was determined using a Hirshfeld partitioning scheme (Table 2). In the **X-Me** series, *c*_A,X_ increased in the order O < S < Se, while in the **X-Ar** series, the heteroatoms contributed only nominally to the β-LUMO.

To investigate the relationship between *c*_A,X_ and IET, we have targeted the oxidation of iodide as a model reaction. The relevant energetically accessible one-electron oxidation process for iodide is expressed in equation 4^68^. The redox reaction with a photo-oxidized catalyst, Ox, ostensibly forms a short-lived adduct with one iodide ion prior to complete electron transfer with a second iodide ion (equation 5)^69–71^. Depending on the localization of the frontier molecular orbitals of Ox, it is conceivable that the [Ox···I···I]^2–,‡^ transition state would bring the acceptor orbitals of the catalyst into contact with the donor orbitals of one of the iodide ions. This would create a direct orbital pathway for electron transfer reminiscent of Taube’s 1953 experiment. In contrast to Taube’s model, however, no bridge is proposed in this study and the electron transfer occurs through the pathway created by direct overlap between of the iodide HOMO and the β-LUMO orbital of Ox.

4 $${\mathrm{2I}}^{\mathrm{-}} \to {\mathrm{I}}_{\mathrm{2}}^{{\mathrm{ \bullet -}}}{\mathrm{ + e}}^{\mathrm{-}}E^\circ \approx {\mathrm{ 0}}{\mathrm{.79}}\,{\mathrm{V}}\,{\mathrm{vs}}\,{\mathrm{NHE}}^{68}.$$

5 $${\mathrm{Ox + I}}^-\begin{array}{*{20}{c}} {K_{\mathrm{1}}} \\ \rightleftharpoons \\ {} \end{array}\left[ {{\mathrm{Ox}} \cdots {\mathrm{I}}} \right]^-{\mathrm{ + I}}^- \\ \begin{array}{*{20}{c}} {K_{\mathrm{2}}} \\ \rightleftharpoons \\ {} \end{array}\left[ {{\mathrm{Ox}} \cdots {\mathrm{I}} \cdots {\mathrm{I}}} \right]^{2-,{\mathrm{\ddagger }}}\mathop{\longrightarrow}\limits^{{k_{{\rm Marcus}}}}{\mathrm{Ox}}^-{\mathrm{ + I}}_2^{ \bullet -}.$$

**Fig. 4:**
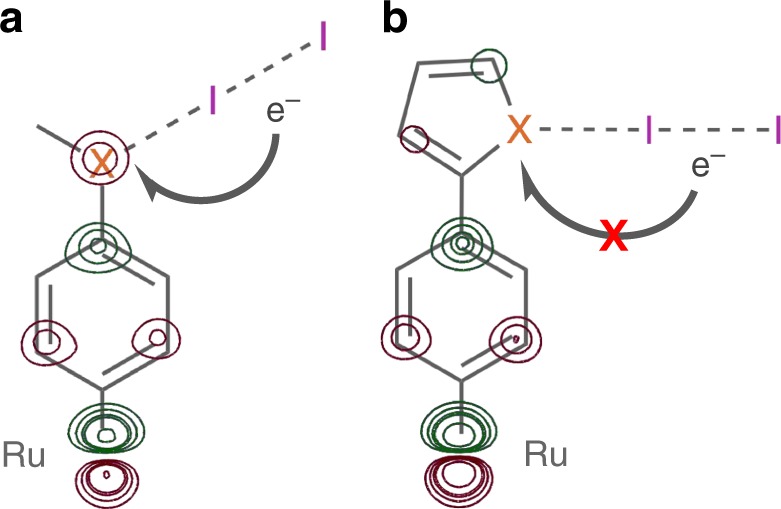
Orbital pathway for IET. A schematic depicting the electron transfer pathway between diiodide **X-Me** **a** or **X-Ar** **b**, through the iodide···chalcogen interaction. The Ru complex fragment is overlaid with a contour plot of the β-LUMO down to an iso-value of 0.05

Figure 2c plots the log of *k*_IET_ as a function of *c*_A,X_. A clear linear relationship is observed between the **X-Me** compounds that is not followed by the **X-Ar** series. These results suggest that there exists an orbital pathway for electron transfer through the chalcogen atoms of **X-Me** that does not exist for **X-Ar** (Fig. 4). In other words, iodide–chalcogen interactions in **X-Me** encourage overlap between the donor orbital of diiodide and the acceptor orbital of the oxidized catalyst, enabling fast IET. By contrast, the **X-Ar** series demonstrates that, in the absence of this orbital pathway, the iodide–chalcogen interaction is not capable of mediating IET. Whereas these results do not eliminate the possibility of other interactions mediating electron transfer in **X-Ar**, such as iodide interacting with the π-system of the thiophene or selenophene rings, the absence of any rate dependence on the identity of the chalcogen atom demonstrates that no orbital pathways exist through that atom.

If iodide–chalcogen interactions are indeed mediating an orbital interaction for IET, then it follows that the observed differences in *k*_IET_ among the **X-Me** series are predominantly a result of differences in *H*_DA_. Unfortunately, the transient pre-ET [Ox···I···I]^2–^ encounter complex could not be observed experimentally, nor could a stable geometry for this complex be found computationally, and therefore *H*_DA_ could not be evaluated directly. In order to investigate the dependance of *k*_IET_ on *H*_DA_, the individual components that make up *k*_IET_ must be deconvoluted and evaluated separately. In practice, the observed second-order *k*_IET_ is related to the first-order electron transfer rate constant, as described by the Marcus equation (*k*_Marcus_, equation 1), by an equilibrium constant, *K*_A_, which represents the stepwise formation for the transient pre-ET [Ox···I···I]^2–^ encounter complex (equation 6)^72^. As a result of this relationship, the observed trends in *k*_IET_ could result from differences either in *K*_A_, *k*_Marcus_, or both.

6 $$k_{{\mathrm{IET}}} = K_{\mathrm{A}}k_{{\mathrm{{Marcus}}}}.$$

The stepwise equilibrium constant *K*_A_ describes the formation of the [Ox···I···I]^2–^ encounter complex. Due to the transient nature of this complex, *K*_A_ could not be quantified. As such, the effects of *K*_A_ on *k*_IET_ could only be evaluated approximately. In the absence of strongly electron-withdrawing groups bonded to the chalcogen, the interactions between iodide and soft atoms like sulfur and selenium are effectively van der Waals interactions and therefore expected to be too weak to draw iodide out of solution toward the oxidized catalyst. Furthermore, the rates of IET (10^3^–10^5^ M^−1^ s^−1^) for our systems are low compared to the diffusion limit (~10^10^ M^−1^ s^−1^)^73^, and thus many transient catalyst···iodide interactions form and break within the lifetime of the oxidized complex. Stronger intermolecular interactions could therefore increase the lifetime of the initial [Ox···I^–^] adduct, increasing the likelihood that a second iodide will encounter that adduct to create the [Ox···I···I]^2–^ encounter complex and complete electron transfer. While this mechanism is plausible, it would predict the same rate enhancement for both **X-Ar** and **X-Me**, which is clearly not the case. Nonetheless, to eliminate this possibility, these adducts were modeled via DFT methods. The optimized interaction geometries are presented in Supplementary Figure 13 and the electronic energies of these interactions (Δ*E*_int_) presented in Supplementary Table 3. These computational models show only a modest increase in adduct stability, *K*_1_, between **O-Me** and the remaining compounds in the **X-Me** series, and actually predict a slight decrease in stability between **S-Me** and **Se-Me**. The initial [Ox···I^–^] adduct stability clearly cannot account for the observed trends in *k*_IET_. We have approximated the overall association constant *K*_A_ using semi-empirical methods (Supplementary Discussion, Supplementary Table 3), and found that, despite the differences in the association of the first iodide, the second iodide association, *K*_2_, dominated the values for *K*_A_. As a result, *K*_A_ was invariant with respect to compound identity. Because the calculations yielding these values for *K*_A_ are based on a number of rough approximations, we do not consider these values to be quantitatively accurate, however, given the homologous nature of the compounds under investigation in this study, any errors introduced by our assumptions should be consistent across both compound series, and therefore these *K*_A_ values are suitable to draw qualitative conclusions.

Because the observed trends in *k*_IET_ cannot be explained by the association constant *K*_A_, it follows from equation 6 that these trends must arise from differences in *k*_Marcus_ instead. Following the Marcus equation (equation 1), *k*_Marcus_—and by extension *k*_IET_—primarily depends on the driving force for electron transfer, Δ*G*°_ET_, the reorganization energy, *λ*, and the electronic coupling, *H*_DA_. Of these, Δ*G*°_ET_ can be obtained from electrochemical measurements and *λ* can be estimated using relatively straight-forward computational techniques, leaving *H*_DA_ as the only variable that cannot be directly evaluated. Among the compounds under investigation here, *λ* is unlikely to vary significantly as the compounds are structurally well conserved. This is supported by computational analysis which predict *λ* = ~1.33 ± 0.03 eV for all compounds under investigation (Supplementary Discussion, Supplementary Table 3). While the Ru^III/II^ redox couples were relatively well conserved, the small differences that did exist will necessarily affect Δ*G*°_ET_ and in turn impact *k*_IET_ to some extent (Supplementary Discussion, Supplementary Table 3). That said, the trends in Δ*G*°_ET_ do not match the trends in *k*_IET_, and therefore significant contributions from *H*_DA_ must be involved in our kinetic results. To better understand the importance of these contributions, and therefore better understand the relationship between *k*_IET_ and the heteroatom contributions to the β-LUMO, *c*_A,X_, it is necessary to deconvolute the effects of Δ*G*°_ET_ and *H*_DA_ via a more involved analysis of our kinetic data.

Recently, *H*_DA_ has been shown to be roughly proportional to the overlap integral, *S*_DA_, between the donor and acceptor frontier molecular orbitals, *ϕ*_D_ and *ϕ*_A_, respectively (equation 7)^38^. In turn, *ϕ*_A_ can be approximated as a linear combination of atomic orbitals, *φ*_A,i_, from each atom i in the acceptor molecule, weighted by their individual contribution coefficients, *c*_A,i_ (equation 8). In the present study, we are concerned only with the iodide-heteroatom interaction, therefore for our purposes *S*_DA_ is approximated as the overlap between an empty p-orbital on the heteroatom, *φ*_A,X_, weighted by *c*_A,X_ and a filled p-orbital on iodide, *φ*_D,I_ (equation 9). For simplicity, the donor is approximated as a single iodide, and therefore the corresponding contribution coefficient, *c*_D,I_, is taken to be 1. The value *S*°_DA_ is the overlap integral of a valence p-orbital of an isolated heteroatom cation and the 5p orbital of iodide, which can be calculated analytically using formulas developed by Mulliken (Supplementary Discussion, Supplementary Table 3)^74^. This approximate overlap integral *c*_A,X_*S*°_DA_ is unitless and directly proportional to *H*_DA_, and therefore we can introduce a constant, *A*, with units of eV and a value of 1 to correct for units. Using this value *Ac*_A,X_*S*°_DA_ in place of *H*_DA_ in the Marcus equation allows us to generate a theoretical pseudo-rate constant, which we have denoted *γ* (Equation 10). This pseudo-rate constant differs from the true *k*_Marcus_ by a constant that is equivalent across all 5 compounds under investigation here.

7 $$H_{{\mathrm{DA}}} \propto S_{{\mathrm{DA}}} = \left\langle {\phi _{\mathrm{D}}\left| {\phi _{\mathrm{A}}} \right.} \right\rangle.$$

8 $$\phi _{\mathrm{A}} \approx {\sum} {c_{{\mathrm{A,i}}}\varphi _{{\mathrm{A,i}}}}.$$

9 $$S_{{\mathrm{DA}}} \approx c_{{\mathrm{A,X}}}\left\langle {\varphi _{{\mathrm{D,I}}}\left| {\varphi _{{\mathrm{A,X}}}} \right.} \right\rangle = c_{{\mathrm{A,X}}}S^\circ _{{\mathrm{DA}}}.$$

10 $$\gamma = \frac{{2\pi }}{\hbar }\left| {Ac_{A,X}S^\circ _{XI}} \right|^2\frac{1}{{\sqrt {4\pi \lambda {\rm RT}} }}e^{\left( {\frac{{ - (\lambda + \Delta G^\circ _{{\rm ET}})^2}}{{4\lambda {\rm RT}}}} \right)}.$$

**Fig. 5:**
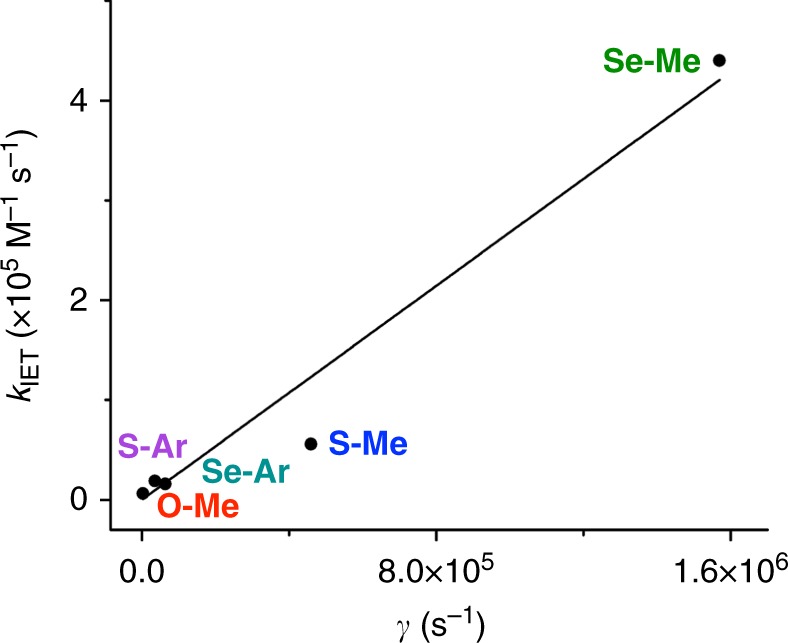
Relationship between *k*_IET_ and the pseudo-rate constant *γ* proportional to *k*_Marcus_. A plot of *k*_IET_ for the indicated compounds as a function of the pseudo-rate-constant, *γ*, calculated analogously to *k*_Marcus_ but substituting *Ac*_A,X_*S*°_DA_ for *H*_DA_ (equation 10). The black line is a linear fit of the data with the *x* and *y* intercepts held at 0 (adj. *R*^2^ = 0.968)

Following equation 6, *γ* should also be proportional to *k*_IET_, assuming a uniform association constant *K*_A_ across all compounds. We can therefore evaluate the relative importance of Δ*G*°_ET_, *λ*, and *Ac*_A,X_*S*°_DA_ (as a surrogate for *H*_DA_) by relating *k*_IET_ directly to *γ* (Fig. 5). Using Δ*G*°_ET_ values calculated from electrochemical data and *λ* values determined computationally (Supplementary Discussion, Supplementary Table 3), this plot reveals an approximately linear relationship between *k*_IET_ and *γ* for all 5 compounds under investigation. This analysis shows that our measured rate constants are consistent with Marcus theory and implies that, while not a perfect analogue, *Ac*_A,X_*S*°_DA_ provides a reasonable—and easily calculated—first approximation of the trends in *H*_DA_. Additionally, this analysis can be used to illustrate the relative importance of Δ*G*°_ET_, *λ*, and *Ac*_A,X_*S*°_DA_ to the observed trends in *k*_IET_. Supplemental Figure 14 shows how the linear relationship between *k*_IET_ and *γ* is affected when *γ* is recalculated using the average value of each of the critical variables. Ignoring the differences in *Ac*_A,X_*S*°_DA_ causes a complete loss in linearity (Supplementary Fig. 14a), while averaging Δ*G*°_ET_ or *λ* results in only minor deviations from linearity (Supplementary Fig. 14b,c). This result shows that *Ac*_A,X_*S*°_DA_, and by extension *H*_DA_, is the principal factor affecting *k*_IET_. Finally, Supplemental Figures 14d and 14e show that if the differences in *c*_A,X_ are ignored then linearity is completely lost, while only a partial loss in linearity is observed if the differences in *S*°_DA_ are ignored, thereby suggesting that that *c*_A,X_ is the more important component of *Ac*_A,X_*S*°_DA_.

Because, *c*_A,X_ and *λ* were both calculated using computational methods, there exists the possibility that these values, and by extension the conclusions we have drawn from Fig. 5, are sensitive to the DFT methodology used. To address this possibility, we have calculated *c*_A,X_ and *λ* using several popular DFT functionals (Supplementary Table 4). The absolute value of *c*_A,X_ varied somewhat between different functionals, particularly the pure generalized gradient functional BP86, however the trend among these values is well conserved in all cases. *λ* was largely invariant depending on the functional. Using these values, the linear relationship between *k*_IET_ and *γ* was found to be at least as strong, and in some cases stronger, with other functionals compared to PBE0 (Supplementary Figure 15). This result demonstrates that, while the choice of functional can have an effect of the quantitative results of our analysis, the qualitative conclusions are nonetheless supported under a wide variety of computational methods.

In conclusion, two parallel series of ruthenium-based catalysts have been synthesized bearing chalcogen-containing substituents in direct electronic contact with the ruthenium center. In the **X-Me** series, the β-LUMO of the oxidized compounds is significantly delocalized onto the chalcogen atom, with increasing participation of that atom in the order O < S < Se. This increase in β-LUMO character on the heteroatom correlates with an increase in the electronic coupling term *H*_DA_ between the photo-oxidized catalyst and iodide, resulting in logarithmic increases in the observed rate of IET. The participation of the heteroatom in the β-LUMO is minimal for the **X-Ar** series and thus no change in *k*_IET_ is observed. These differences in *k*_IET_ clearly imply that iodide interacts with the chalcogen in oxidized **X-Me**^**•+**^ to create an orbital pathway between iodide and the β-LUMO of the ruthenium complex, similar to the purported chloride bridge in Taube’s 1953 experiment. This observation motivates the design of next-generation redox catalysts that enable orbital pathways for IET and suggests that simple, easily accessible DFT methods can serve as a predictive tool informing catalyst design.

## Methods

### Characterization of methyl esters in solution

Acetonitrile was purchased from Sigma-Aldrich and purified on an MBraun solvent purification system prior to use. Tetrabutylammonium tetrafluoroborate was purchased from Alfa Aesar, recrystallized from boiling 50% water/ethanol, and dried under high vacuum prior to use. UV-visible spectra were recorded using ~30 μM acetonitrile solutions on a Varian Cary 5000 spectrophotometer at a resolution of 1 nm. Cyclic voltammograms were recorded using a CH Instruments 660D potentiostat at room temperature using a standard three-electrode configuration (working electrode: 2 mm diameter Pt disc; reference electrode: RE-5B Ag/AgCl electrode in saturated aqueous potassium chloride (BASi Inc.), referenced externally to ferrocene/ferrocenium (0.630 V vs NHE);^75^ counter electrode: Pt wire) in 0.1 M tetrabutylammonium tetrafluoroborate acetonitrile solutions.

### Characterization of functionalized thin films

Acetonitrile was purchased from Honeywell (Burdick and Jackson, 99.99 %) and used as received. Sodium iodide (NaI, 99.9%), sodium perchlorate (NaClO_4_,>98%), and lithium perchlorate (LiClO_4_, 99.99%) were purchased from Sigma-Aldrich and used as received. UV-visible absorption spectra of the functionalized films were recorded on a Varian Cary 60 spectrophotometer with a resolution of 1 nm. Spectroelectrochemical measurements were performed using a WaveNow potentiostat (Pine Research Instrumentation, Inc.) at room temperature (22 ± 1 °C) with an AvaSpec-2048 fiber-optic spectrometer (Avantes) and an AvaLight-DHc light source (Avantes). Measurements used a standard three-electrode configuration (working electrode: functionalized nano-ITO on FTO; reference electrode: non-aqueous Ag/AgCl (0.5 M NaClO_4_ in MeCN), referenced externally to ferrocene/ferrocenium (0.630 V vs NHE);^75^ counter electrode: platinum wire) in 0.5 M NaClO_4_. The reference electrode was mounted in a Vycor-tipped glass tube with electrolyte to avoid contamination.

### General procedure for transient absorption experiments

Transient absorption spectra and kinetics were acquired on an apparatus that has been described in literature^76^. Briefly, 532 nm excitation was achieved either with a frequency doubled Q-switched, pulsed Nd:YAG laser (Quantel USA (BigSky) Brilliant B; 532 nm, 5–6 ns full width at half-maximum (fwhm), 1 Hz, ∼10 mm in diameter) or a laser of the same model, frequency tripled (355 nm) coupled to an optical parametric oscillator (OPO, Opotek, Inc.) tuned to 532 nm. The 532 nm beam was then directed through two Glan laser polarizers to attenuate the pulse fluence (typically kept between 0.5 and 1 mJ per pulse) and was directed 45° to the film surface. A 150 W xenon arc lamp (Applied Photophysics), pulsed with 70 V, served as the probe beam and was aligned orthogonally to the excitation laser. Before the sample, the light was focused through a monochrometer (GM 252) to minimize background excitation of the samples. Detection was achieved with a monochromator (Spex 1702/04) optically coupled to an R928 photomultiplier tube (Hamamatsu). Transient data were acquired on a computer-interfaced digital oscilloscope (LeCroy 9450, Dual 350 MHz) with 2.5 ns resolution terminated at 50 Ω. For longer timescale data acquisition, the xenon arc lamp was run continuously (not pulsed or shuttered) and the oscilloscope was terminated at 50 kΩ. To completely model the full kinetic decays data taken at multiple timescales were stitched together.

NaClO_4_ and NaI electrolytes were prepared in acetonitrile and purged with argon for at least 15 min prior to use. The functionalized metal oxide thin films were placed in a 1 cm^2^ cuvette with a 24/40 ground glass joint and fully submerged in argon-purged electrolyte solutions. The cuvette was then purged with argon for an additional 5 min and argon was continuously purged through the headspace of the cuvette throughout the measurements.

All single wavelength kinetic decays were modeled through the Kolrausch-Williams-Watts stretched exponential function^77,78^. The decay fitting was performed in Origin 2016pro, and least-squares error minimization was accomplished using the Levenberg–Marquardt iteration method.

### Full spectrum transient absorption

Kinetic traces were monitored in 0.5 M LiClO_4_/MeCN from −10 to 90 μs and collected every 10 nm between 400 and 800 nm. The laser power was ~2 mJ per pulse and 30 sequential laser pulses were averaged at each collected wavelength.

### IET and BET kinetics

IET and BET kinetics were investigated using the functionalized SnO_2_–TiO_2_ core-shell films submerged in fixed NaClO_4_/NaI acetonitrile solutions with a total salt concentration of 0.5 M. The ratio between the two was varied to monitor IET at varied iodide concentrations between 0 and 0.5 M. The laser power was attenuated to ensure the amount of oxidized dye produced upon excitation was low and similar between all compounds studied, between 0.5 and 1 mJ per pulse. Single wavelength kinetic decays were collected at 580 nm, close to an isosbestic point of the electric field effect for all compounds to ensure only IET was monitored. The final data were averaged between 90 and 150 laser pulses, to achieve an acceptable signal to noise ratio.

### Electrolyte recombination kinetics

The kinetics of charge recombination between injected electrons and triiodide were monitored at 375 nm in 0.5 M NaI from −10 μs to 1 s after pulsed laser excitation. The laser power was between 1 and 3 mJ per pulse. The final data were averaged over 500 laser pulses to achieve an acceptable signal to noise ratio.

### Additional methods

Detailed descriptions of the synthetic procedures to prepare and characterize the **X-Me** and **X-Ar** compounds, the procedures to prepare functionalized metal oxide thin film samples, and the computational methods employed in this investigation can be found in Supplementary Methods section of Supplementary Information.

## Electronic supplementary material

Supplementary Information

Description Of Additional Supplementary Files

Supplementary Data 1

Supplementary Data 2

## Data Availability

Supplementary Figures, Supplementary Methods, Supplementary Tables, and Supplementary Notes are included in the Supplementary Information file. DFT optimized molecular geometries and optical transitions calculated by time-dependent DFT are reported in the Supplementary Data 1 and Supplementary Data 2 files, respectively. Additional data supporting these findings are available from the corresponding authors upon request.
