# Supplementary material for: Resolving orbital pathways for intermolecular electron transfer
Source: Nat Commun. 2018 Nov 21;9:4916. doi: 10.1038/s41467-018-07263-1 (PMC6249235; doi:10.1038/s41467-018-07263-1)
Supplement: Supplementary file 1 — Supplementary Information [file 41467_2018_7263_MOESM1_ESM.pdf]

Supplementary Information for

Resolving Orbital Pathways for Intermolecular Electron Transfer

Cameron W. Kellett[1]; Wesley B. Swords[2]; Michael D. Turlington[2]; Gerald J. Meyer[2,*]; Curtis P. Berlinguette[1,3,4,*]

correspondence to: gjmeyer@email.unc.edu; cberling@chem.ubc.ca

[1]Department of Chemistry, 2036 Main Mall, University of British Columbia, Vancouver, BC V6T 1Z1, Canada.

[2]Department of Chemistry, University of North Carolina at Chapel Hill, Murray Hall 2202B, Chapel Hill, NC 27599-3290, USA.

[3]Department of Chemical and Biological Engineering, 2360 East Mall, University of British Columbia, Vancouver, BC V6T 1Z3, Canada.

[4]Stewart Blusson Quantum Matter Institute, 2355 East Mall, University of British Columbia, Vancouver, BC V6T 1Z4, Canada

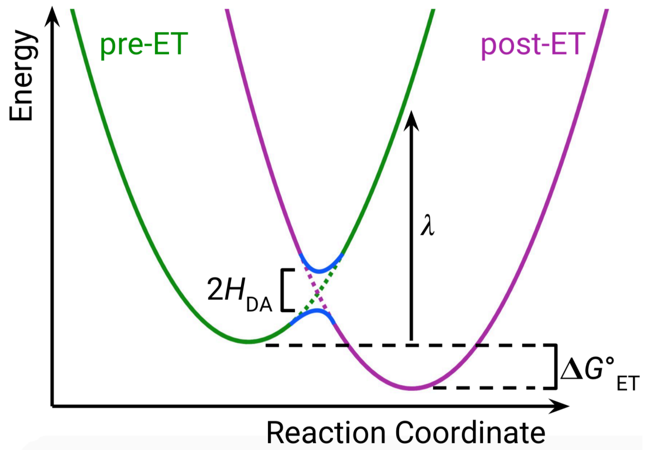

Supplementary Figure 1 | Marcus theory. A simplified potential energy surface for a generic electron transfer reaction. In this diagram, the green and purple parabolas represent the energy of the donor-acceptor pair before and after electron transfer, respectively, and the blue region represents the deviation of the reaction coordinate from ideality due to electronic coupling. The variables described in this plot correspond to their values in the Marcus equation (equation 1 in the main text): $G°_{ET}$ represents the driving force for electron transfer, λ represents the reorganization energy of the donor-acceptor pair, and H_{DA} represents the electronic coupling factor.

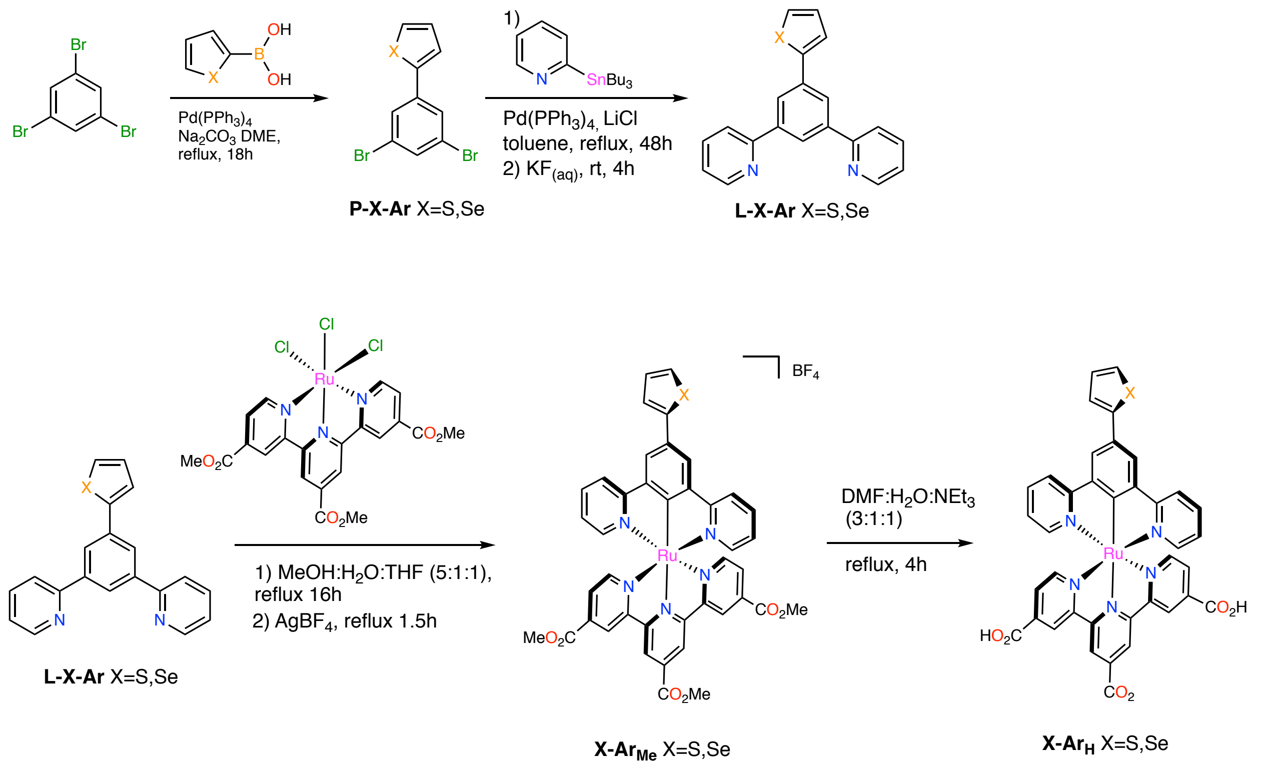

Supplementary Figure 2 | Preparation of X-Ar compounds. General synthetic scheme describing the preparation of the **X-Ar** compound series.

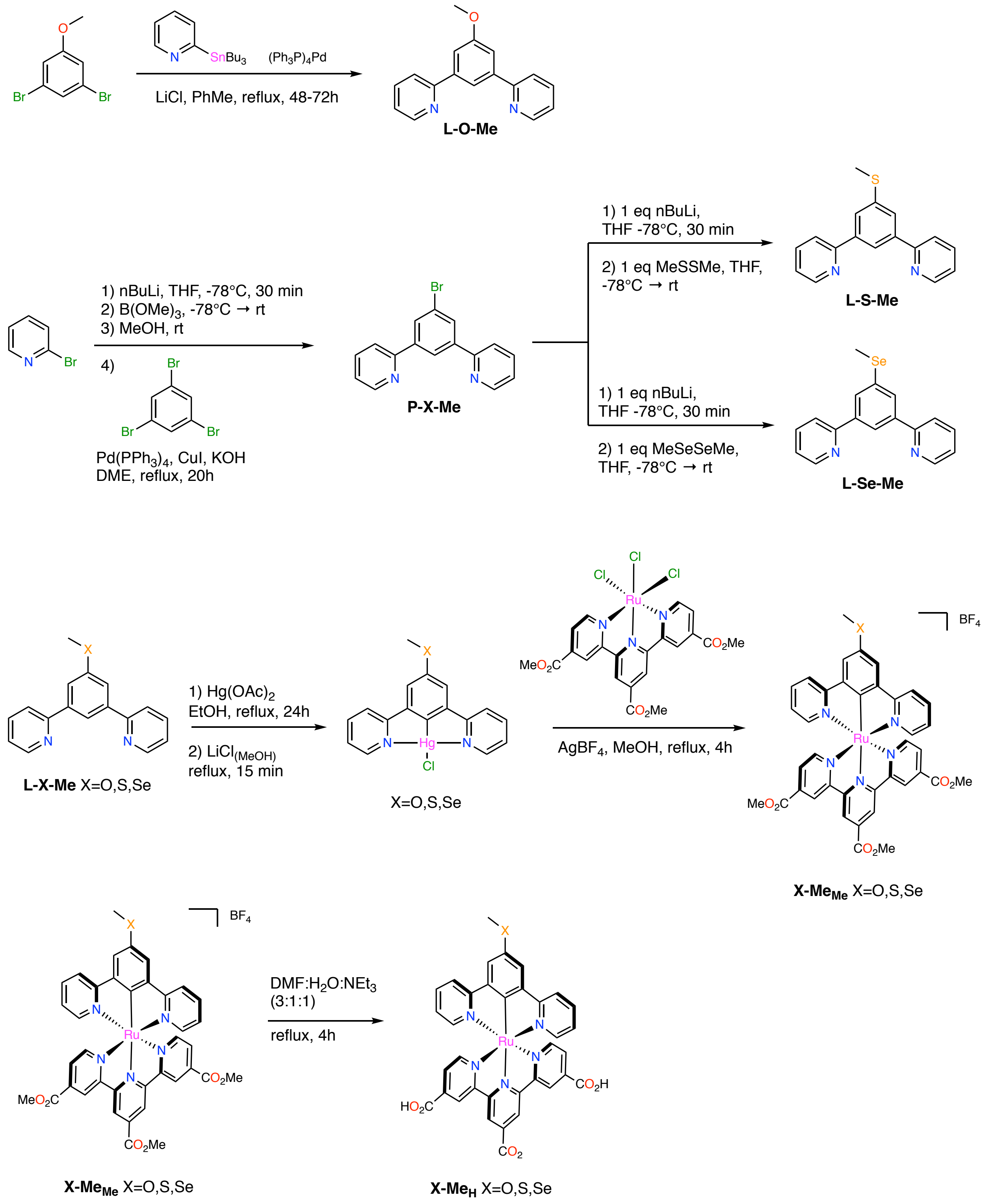

Supplementary Figure 3 | Preparation of X-Me compounds. General synthetic scheme describing the preparation of the **X-Me** compound series.

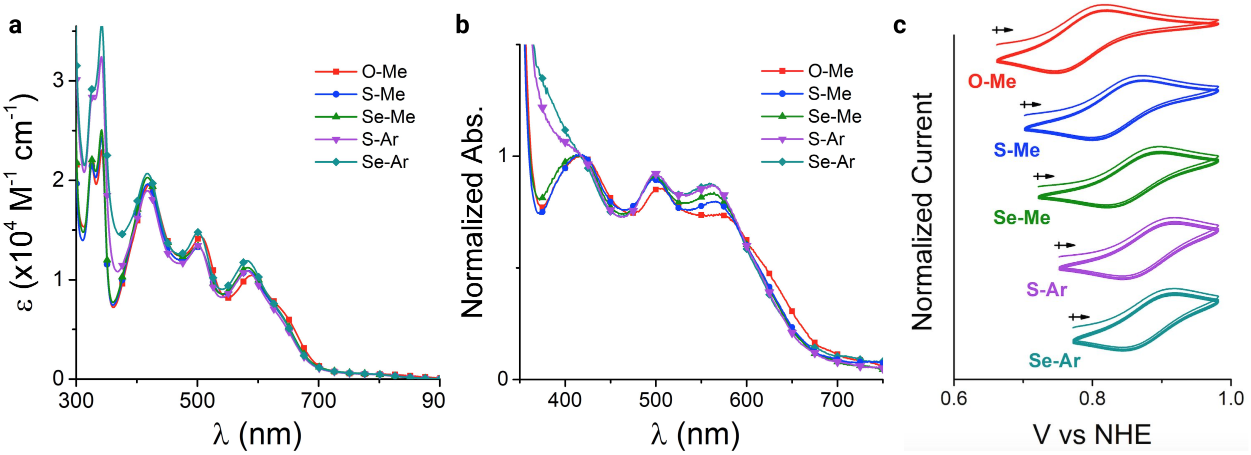

Supplementary Figure 4 | Optical and redox properties in solution and at the interface. a,b, UV-Vis spectra of **O-Me** (red squares), **S-Me** (blue circles), **Se-Me** (green triangles), **S-Ar** (purple inverted triangles), and **Se-Me** (teal diamonds) as methyl esters in acetonitrile solution (**a**) and saponified catalysts anchored to TiO_2 thin films (**b**). **c,** CVs of the indicated compounds as methyl esters in 0.1 M $(NBu_4)BF_4$ acetonitrile solution, collected at 100 mV/s using a Pt working electrode, Pt wire counter electrode, and a Ag/AgCl reference electrode externally referenced to ferrocene/ferrocenium (0.630 V vs NHE).[1]

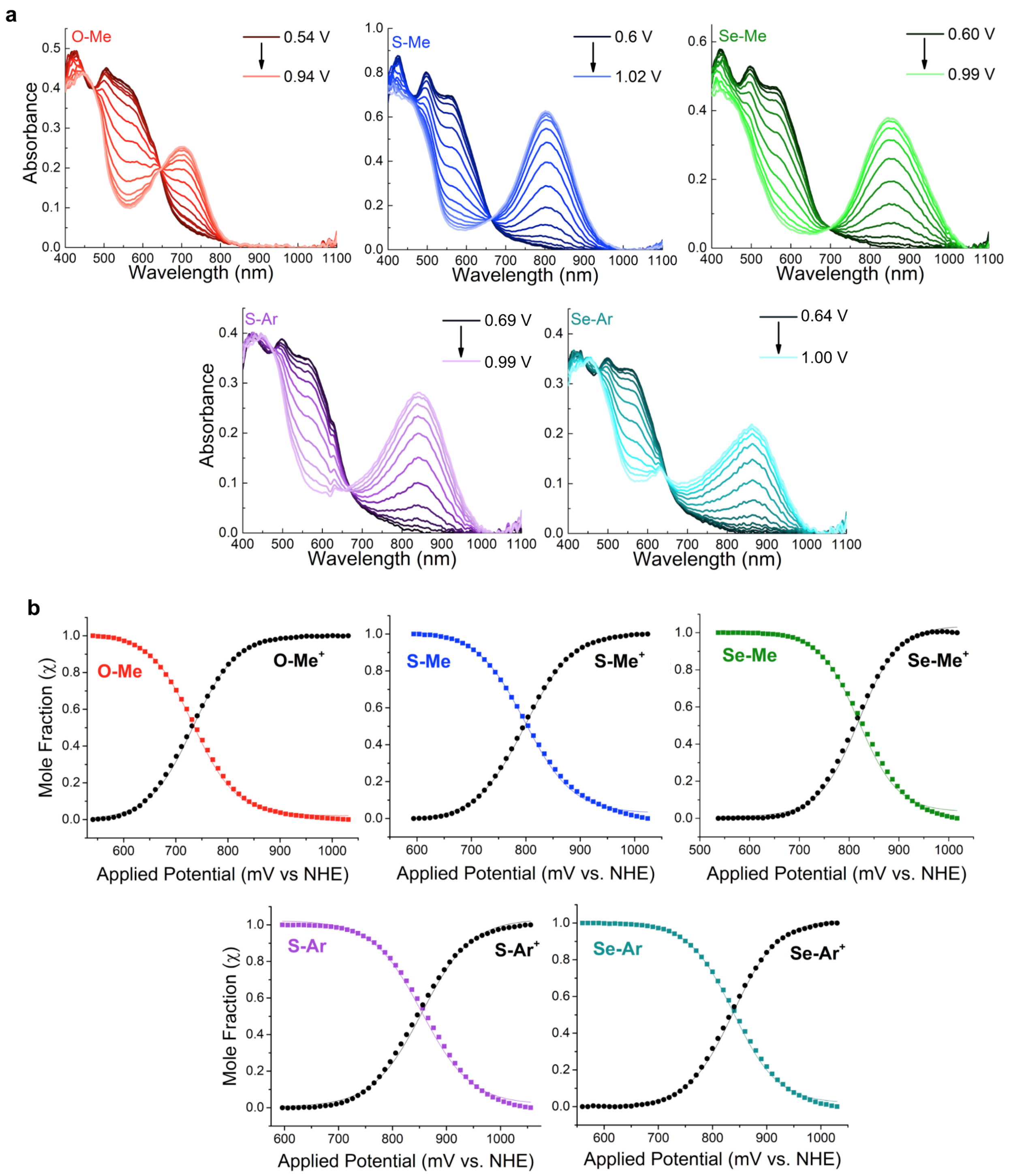

Supplementary Figure 5 | Spectroelectrochemical analysis of functionalized nano-ITO. a, Optical spectra of the indicated compounds anchored to nano-ITO under a range of applied potentials (indicated in the top left corner of each plot in V vs NHE) in 0.5 M $NaClO_4$ acetonitrile electrolyte. **b,** Spectral modeling was performed to determine the mole fraction of oxidized (black squares) and reduced (colored squares) ruthenium complexes at each potential.

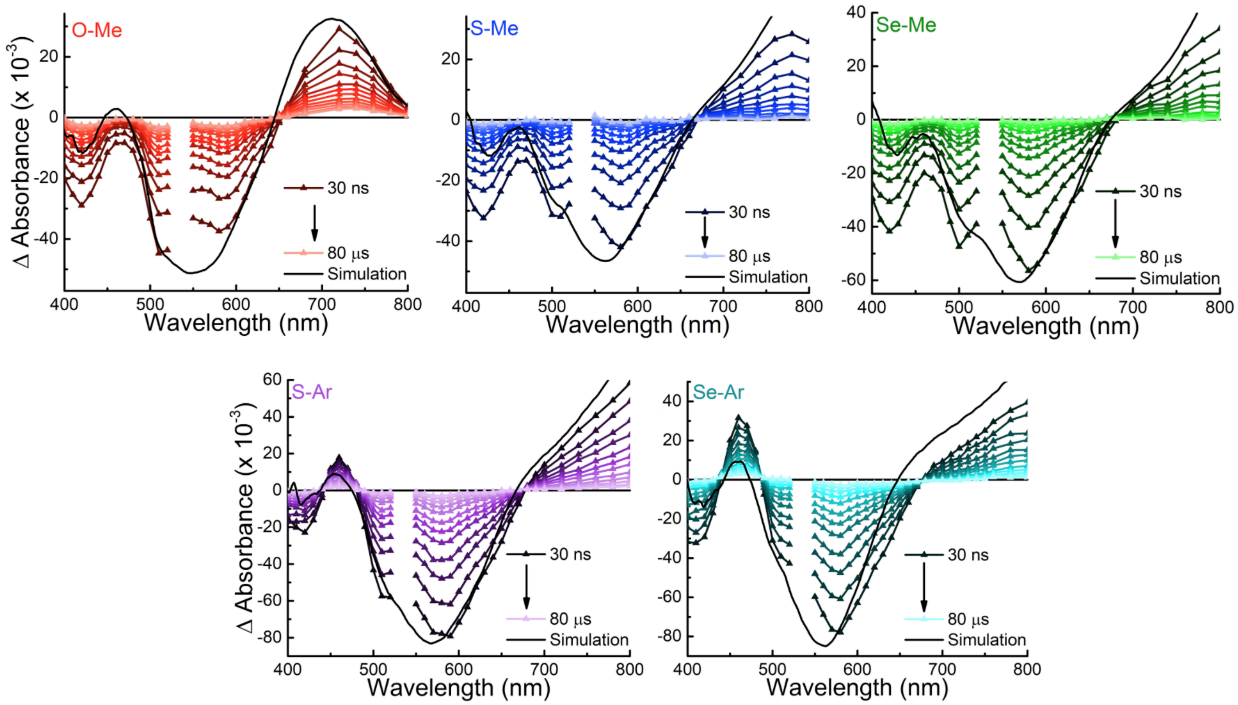

Supplementary Figure 6 | Full spectrum transient absorption. Absorption difference spectra measured at the indicated time delay after 532 nm pulsed laser excitation of the indicated compound anchored to TiO_2 immersed in 0.5 M $LiClO_4$ acetonitrile solution. Overlaid on each plot is the predicted difference spectrum (solid black line) based on the optical spectra of the reduced and oxidized ruthenium complexes, as determined from spectroelectrochemical analysis (Supplemental Figure 5a).

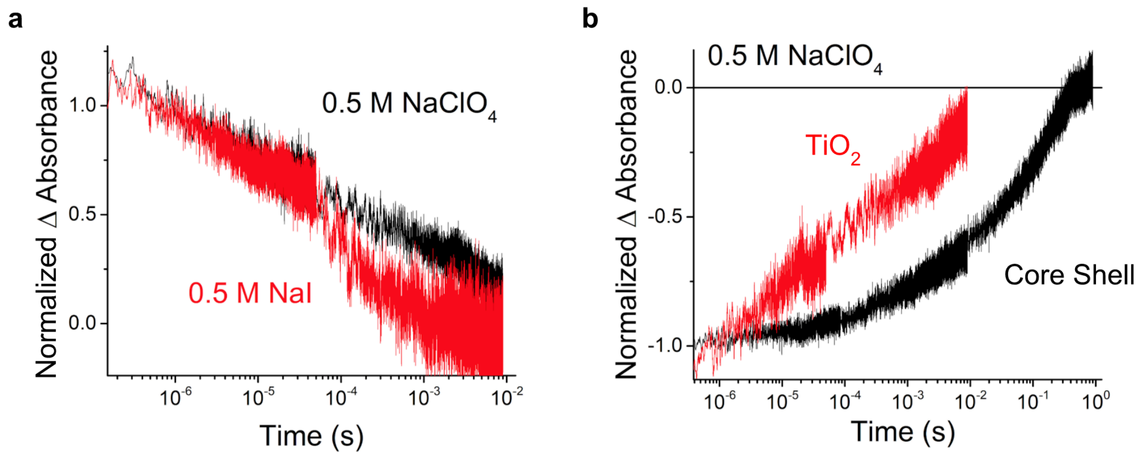

Supplementary Figure 7 | Comparative electron transfer kinetics on TiO_2. Absorption changes monitored following pulsed laser excitation of **Se-Ar** functionalized films at 532 nm. Measurements were taken at 730 nm in 0.5 M $NaClO_4$ electrolyte (black line) or 0.5 M NaI electrolyte (red line) on a TiO_2 substrate (**a**), or at 570 nm in 0.5 M $NaClO_4$ electrolyte using either TiO_2 (red line) or SnO_2/TiO_2 core-shell (black line) substrates (**b**).

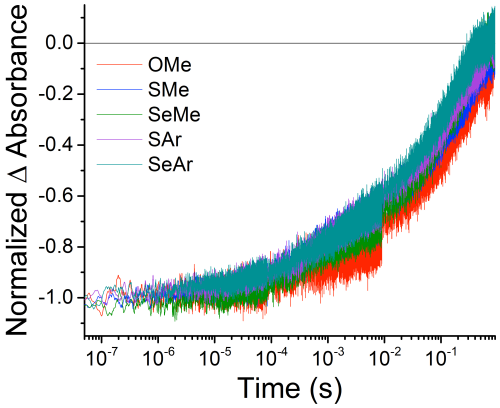

Supplementary Figure 8 | Back-electron transfer kinetics on SnO_2/TiO_2 core shell films. Absorption changes monitored at 570 nm following pulsed laser excitation at 532 nm of SnO_2/ TiO_2 core-shell thin films functionalized with **O-Me** (red line), **S-Me** (blue line), **Se-Me** (green line), **S-Ar** (purple line), or **Se-Ar** (teal line) in 0.5 M $NaClO_4$ acetonitrile electrolyte.

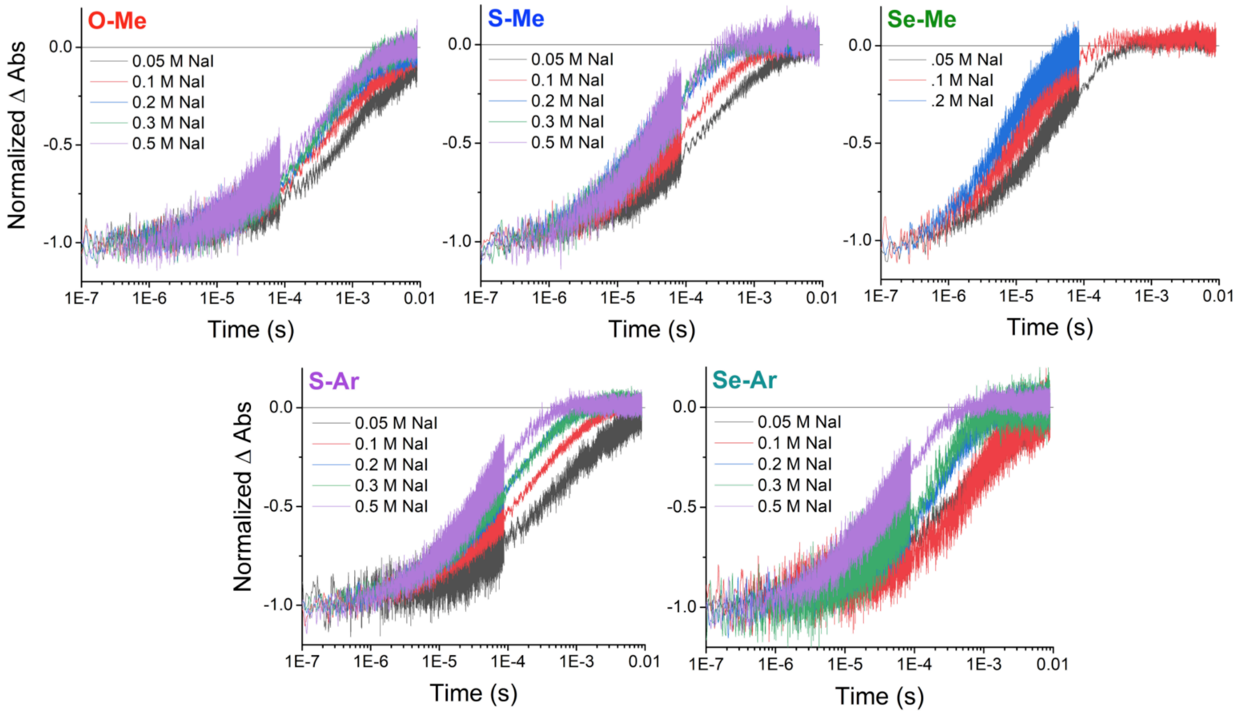

Supplementary Figure 9 | Pseudo-first order IET kinetics. Absorption changes monitored at 570 nm following pulsed laser excitation of SnO_2/TiO_2 core-shell thin films functionalized with the indicated compounds at 532 nm. Experiments were performed in the presence of 0.05 M (black line), 0.1 M (red line), 0.2 M (blue line), 0.3 M (green line), or 0.5 M (purple line) NaI in acetonitrile solutions (see Methods section of the main manuscript for full details).

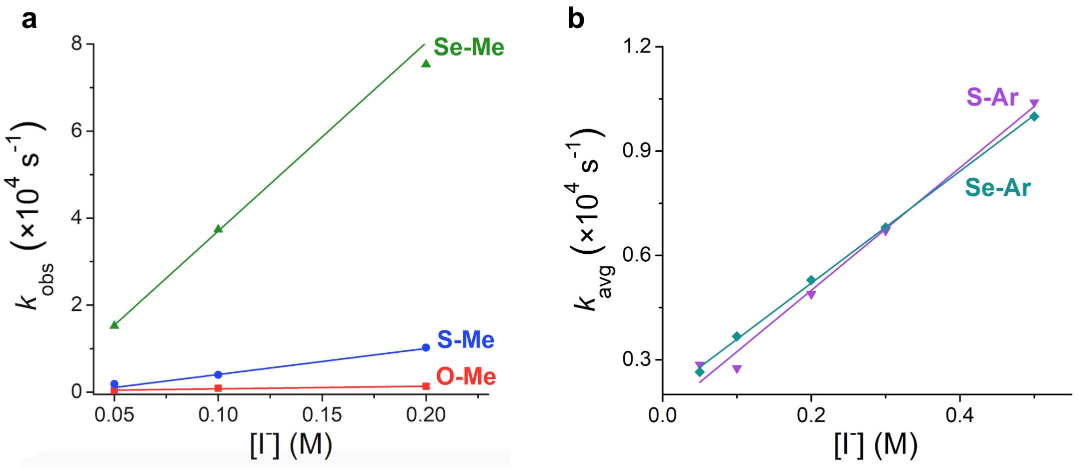

Supplementary Figure 10 | Second order IET kinetics. **a**, Plot of observed pseudo-first order rate constants (k_{obs}) for IET with **O-Me** (red squares), **S-Me** (blue circles), and **Se-Me** (green triangles) anchored to SnO_2-TiO_2 core-shell thin films as a function of iodide concentration in acetonitrile electrolyte. **b**, Plot of observed pseudo-first order rate constants for IET averaged from two different experiments (k_{avg}) with **S-Ar** (purple inverted triangles) and **Se-Ar** (teal diamonds) anchored to SnO_2-TiO_2 core-shell thin films as a function of iodide concentration in acetonitrile electrolyte. In both plots, linear fits of the data for each compound are indicated by solid lines matching the color of the corresponding symbols.

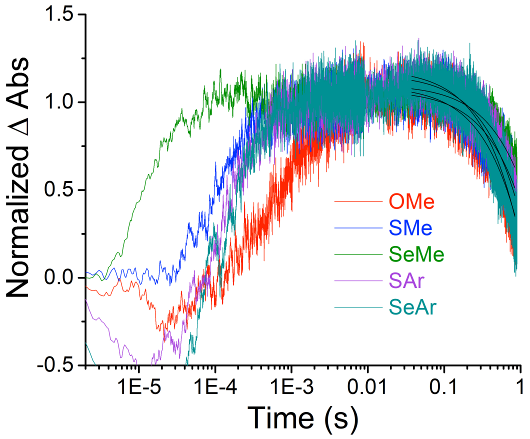

Supplementary Figure 11 | Recombination with triiodide. Absorption changes monitored at 375 nm following pulsed laser excitation at 532 nm of SnO_2/TiO_2 core-shell thin films functionalized with **O-Me** (red line), **S-Me** (blue line), **Se-Me** (green line), **S-Ar** (purple line), or **Se-Ar** (teal line). Experiments were performed in 0.5 M NaI acetonitrile solutions. The rate constant of recombination between injected electrons and photogenerated I_3^- was found to be 2.4×10^{-1} s^{-1} for all compounds.

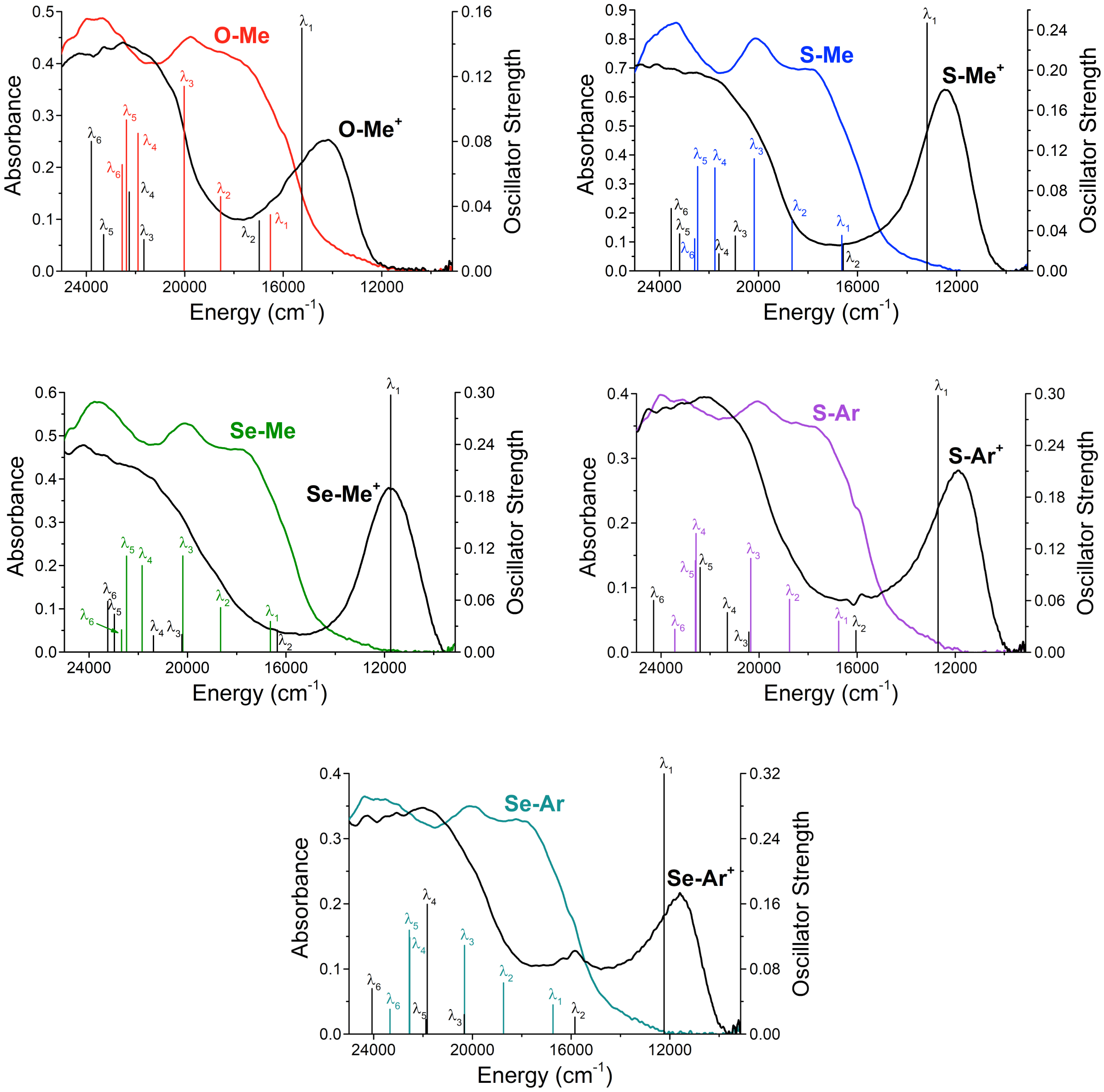

Supplementary Figure 12 | Optical transitions of Ru(II) and Ru(III) compounds on nano-ITO. Comparison of the optical spectra of the reduced (colored lines) and oxidized (black lines) forms of the indicated compounds anchored to nano-ITO, from spectroelectrochemical analysis (Supplemental Figure 5a). The vertical lines represent the major optical transitions calculated with time-dependent density functional theory (TD-DFT). Each transition is visualized in Supplementary Table 1 and details of the calculated transitions are reported in the Supplementary Data 2 file associated with this manuscript.

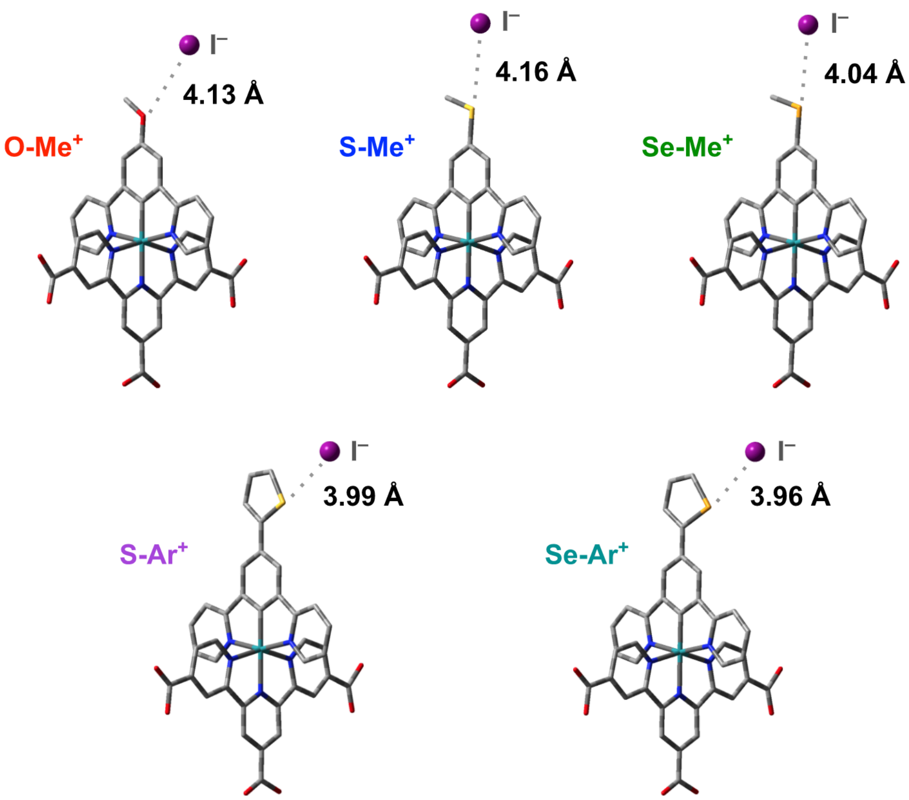

Supplementary Figure 13 | Optimized cat⁺···I⁻ adduct structures. Geometry optimized structures for the cat⁺···I⁻ adducts using the indicated complexes generated by DFT methods. Interatomic distances for the chalcogen-iodide interactions are indicated next to each structure. The interatomic chalcogen-iodide distances predicted from van der Waals interactions are 3.68 Å, 3.96 Å, and 4.06 Å for O···I⁻, S···I⁻, and Se···I⁻, respectively.

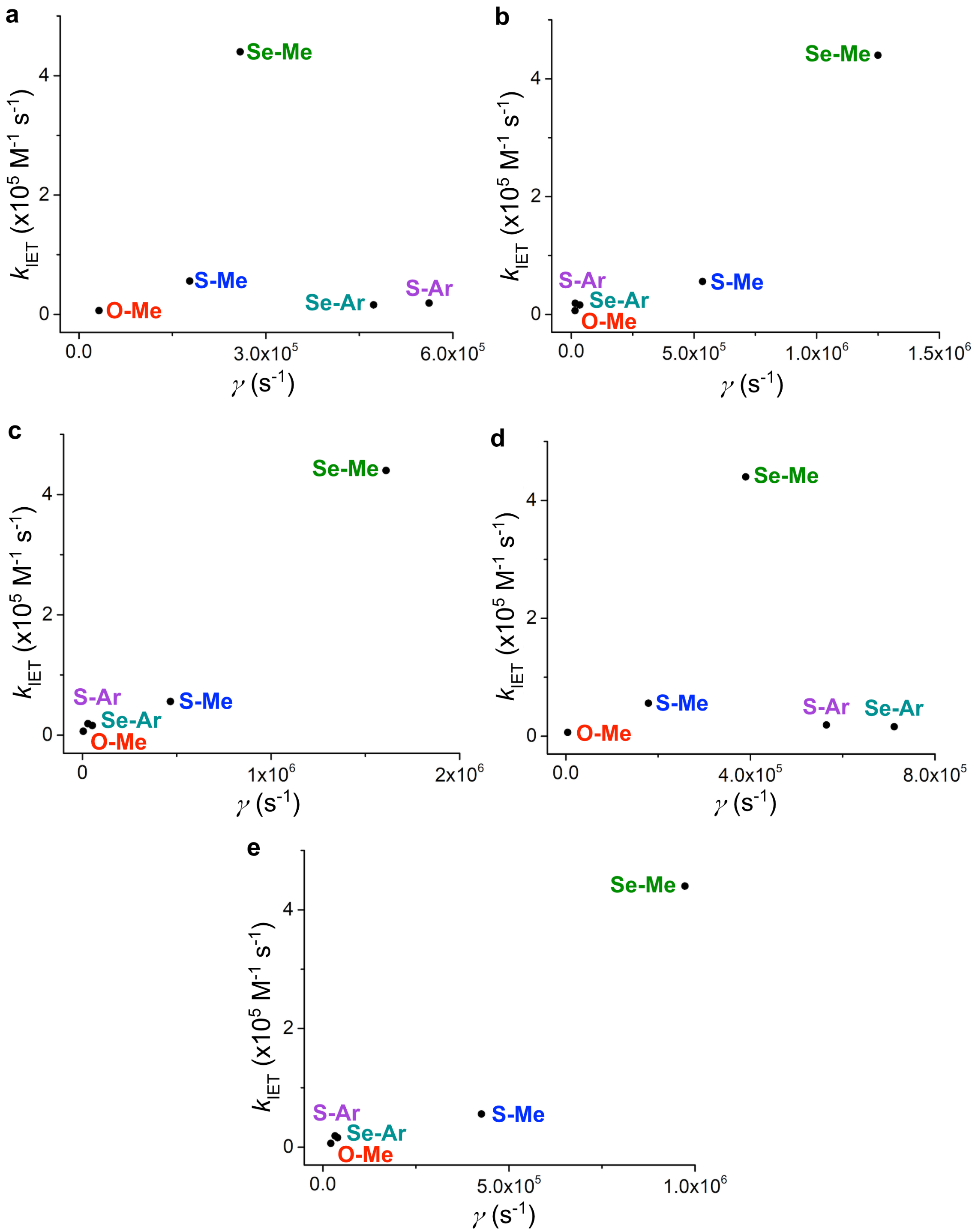

Supplementary Figure 14 | Deconvolution of the effect of the individual variables of the Marcus surrogate on the relationship between k_{IET} and γ. Plot of k_{IET} for the indicated compounds as a function of a Marcus surrogate, γ, calculated analogously to k_{Marcus}, but substituting $Ac_{A,X}S°_{DA}$ (see equation 10 in the main text) for H_{DA} and setting each of the variables $Ac_{A,X}S°_{DA}$ (**a**), $\Delta G°$ (**b**), λ (**c**), $c_{A,X}$ (**d**), and $S°_{DA}$ (**e**) to their average value.

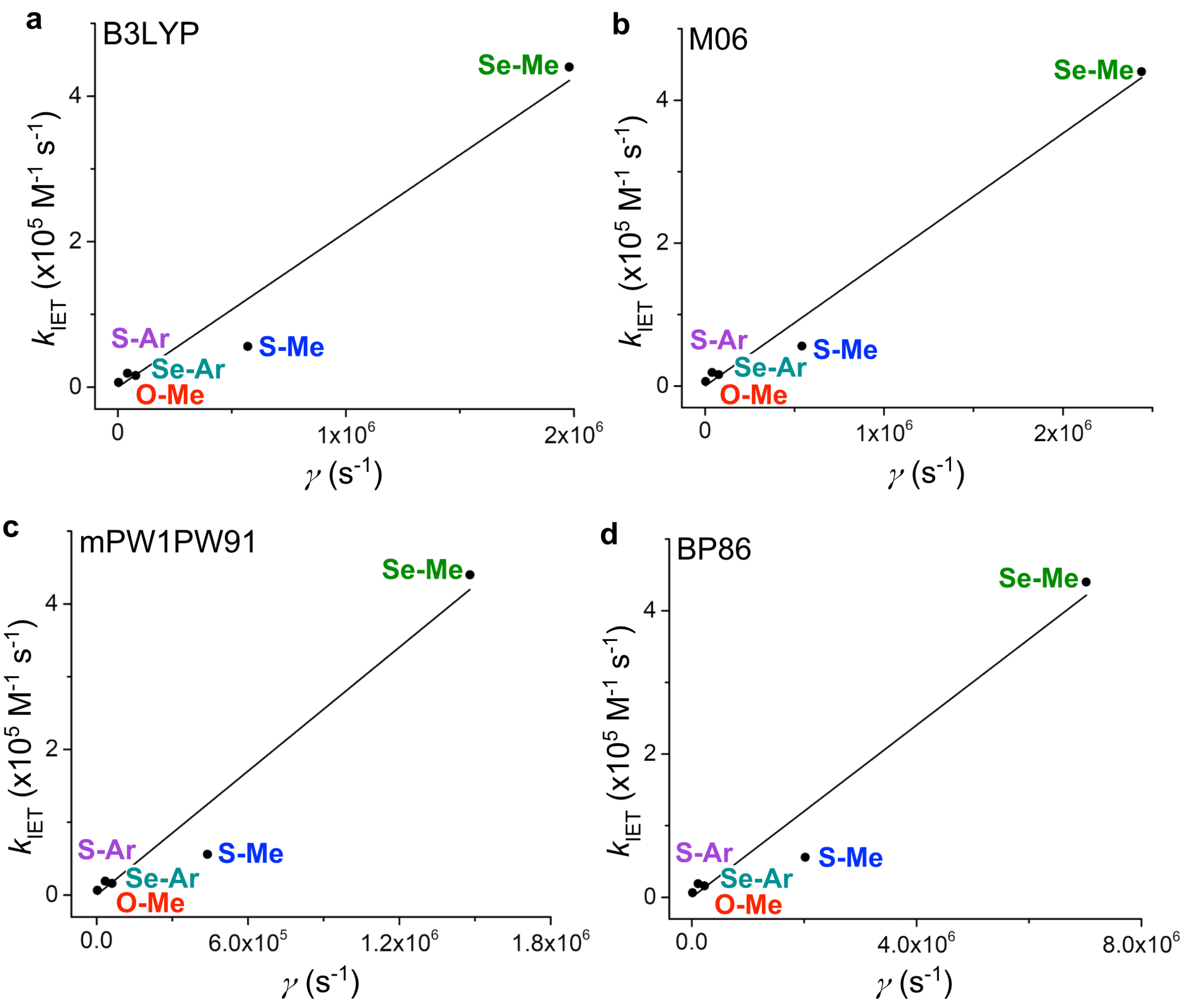

Supplementary Figure 15 | Dependence of the relationship between k_{IET} and γ on DFT functional. Plot of k_{IET} for the indicated compounds as a function of a Marcus surrogate, γ, calculated analogously to k_{Marcus}, but substituting $Ac_{A,X}S°_{DA}$ for H_{DA} (see equation 10 in the main text) and using $c_{A,X}$ and λ values calculated using B3LYP (**a**), M06 (**b**), mPW1PW91 (**c**), and BP86 (**d**). The $c_{A,X}$ and λ values used to generate these plots are reported in Supplementary Table 4. The black line is a linear fit of the data with the x and y intercepts held at 0. Adjusted R squared values for the linear fits of the above plots are 0.970 (**a**), 0.988 (**b**), 0.967 (**c**), and 0.970 (**d**).

Supplementary Methods

Synthesis of compounds

All reagents were obtained from commercial sources and were used as received. All reactions were carried out under a dry nitrogen atmosphere using standard Schlenk techniques. Methanol, dichloromethane, acetonitrile, and tetrahydrofuran were purified on an MBraun solvent purification system prior to use; all other solvents were obtained from commercial sources and used as received. Purification by column chromatography was carried out using silica (Silicycle: SiliaFlash F60). Analytical thin-layer chromatography (TLC) was performed on aluminum-backed sheets pre-coated with silica 60 F-254 adsorbent (250 µm thick; Millipore) and visualized under UV light. ^{1}H, ^{13}C, and ^{19}F NMR spectra were collected on Bruker Avance 300 or 400 instruments at the UBC Nuclear Magnetic Resonance Facility. Chemical shifts (δ) are reported in parts per million (ppm). ^{1}H and ^{13}C spectra were referenced to residual solvent signals,[2] ^{19}F spectra were referenced to an internal standard of $CFCl_3$ at 0 ppm. Coupling constants (J) are reported in Hertz (Hz). Multiplicities are reported by using the following abbreviations: s, singlet; d, doublet; dd, doublet of doublets; ddd, doublet of doublet of doublets; t, triplet; dt, doublet of triplets; td, triplet of doublets; m, multiplet. 0.1M $NaOCD_3$/ CD_3OD solvent for the NMR analysis of the saponified catalysts was prepared immediately before use from methanol-d_4 by the addition of 2.4 mg/ml dry NaH. High-resolution mass spectra were recorded using electrospray ionization (ESI) on a Waters/Micromass LCT TOF-MS or electron ionization (EI) on a Kratos MS-50, as indicated, in the UBC Mass Spectrometry Centre. Precursor compounds 2-thienylboronic acid,[3] 1,3-dibromo-5-(2-thienyl)-benzene (**P-S-Ar**),[4] 2-tributylstannyl-pyridine,[5] dimethyl 2-pyridyl-borane,[6] 1,3-(2-pyridyl)-5-bromo-benzene,[7] 3,5-bis(2-pyridyl)-anisole (**L-O-Me**),[8] 4,4′,4′′-tricarboxymethyl-2,2′:6′,2′′-terpyridine (Me_3tctpy),[9,10] and Ru(Me_3tctpy)Cl_3[11] were prepared as previously reported.

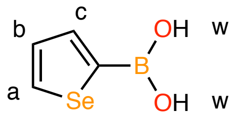

2-selenylboronic acid. To a solution of selenophene (1.12 ml, 12.2 mmol) in 20 ml tetrahydrofuran at -78°C was added n-butyl lithium (1.6M in hexanes, 7.6 ml, 12 mmol) dropwise. The mixture was allowed to warm to room temperature, then cooled back to -78°C and triisopropylborate (3.95 ml, 17.1 mmol) was added dropwise. The resulting mixture was allowed to warm slowly to room temperature overnight. The mixture was then quenched by the dropwise addition of 1 M $HCl_{(aq)}$ (30 ml) and extracted with 5x30 ml dichloromethane. The combined organic layers were dried with $MgSO_4$ and concentrated to an off-white solid. The crude product was dissolved in boiling water, filtered while hot, and concentrated under

reduced pressure to yield 2-selenylboronic acid as a colorless solid (1.97 g, 92% yield). ^{1}H NMR (300 MHz, $(CD_3)_2SO$): δ = 8.41 (d, 1H, ^3J = 5.2 Hz, $^2J_{1H\text{-}77Se}$ = 22.0 Hz, H_a), 8.16 (s, 2H, H_w), 7.94 (d, 1H, ^3J = 3.5 Hz, H_c), 7.41 (dd, ^3J = 5.0 Hz, ^3J = 3.7 Hz, H_b).

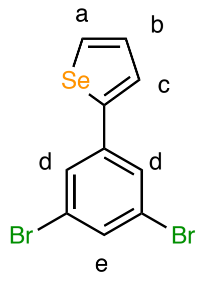

1,3-dibromo-5-(2-selenyl)-benzene (P-Se-Ar). A mixture of 12 ml dimethoxyethane and sodium carbonate (0.91 g, 8.6 mmol) in 13 ml water was degassed by refluxing under a nitrogen atmosphere overnight. After cooling to room temperature, 1,3,5-tribromobenzene (0.982 g, 3.12 mmol), 2-selenylboronic acid (0.599 g, 3.42 mmol), and tetrakis(triphenylphosphine)palladium (0.11 g, 0.095 mmol) were added and the mixture brought to reflux for 23 hours. The mixture was then taken up in 25 ml water and 25 ml DCM, separated, and the organic phase washed with water (3x25 ml). The organic phase was then dried with $MgSO_4$ and concentrated to a black oil that solidified on standing. Purification by column chromatography (SiO_2: eluting with hexanes, R_f = 0.55) yielded **P-Se-Ar** as a pale yellow oil that solidified on standing (0.550 g, 48% yield). ^{1}H NMR (400 MHz, $CDCl_3$): δ = 8.02 (dd, 1H, $^2J_{1H\text{-}77Se}$ = 48.2 Hz, ^3J = 5.6 Hz, ^4J = 1.0 Hz, H_a), 7.62 (d, 2H, ^4J = 1.6 Hz, H_d), 7.56 (t, ^4J = 1.7 Hz, H_e), 7.47 (dd, 1H, ^3J = 3.8, ^4J = 1.0 Hz, H_c), 7.33 (dd, 1H, ^3J = 5.6, 3.9 Hz, H_b). ^{13}C NMR (100 MHz, $CDCl_3$): δ = 147.20, 139.97, 132.74, 132.07, 130.89, 128.09, 127.14, 123.48. EI-MS: calc for $C_{10}H_6{}^{81}Br_2{}^{82}Se$ $[M]^+$ m/z = 369.7962, found: 369.7965.

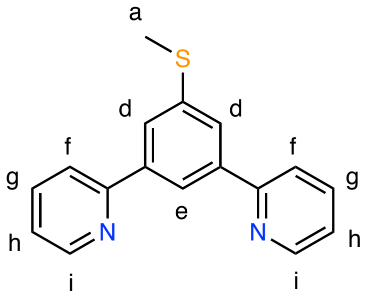

3,5-di(2-pyridyl)-thioanisole (L-S-Me). A solution of 1,3-bis(2-pyridyl)-5-bromo-benzene (0.352, 1.13 mmol) in tetrahydrofuran (40 ml) was cooled to -78°C in a dry ice-acetone bath, and n-butyl lithium (1.6M in hexanes, 0.73 ml, 1.2 mmol) was added dropwise. The resulting black mixture was treated with dimethyldisulfide (0.15 ml, 1.7 mmol) and the mixture allowed to

warm slowly to room temperature overnight. The resulting orange mixture was quenched by the addition of ammonium acetate (0.517 g, 6.71 mmol) in 20 ml methanol and solvent removed under high vacuum. The crude product was taken up in 25 ml dichloromethane and poured into 25 ml water. This mixture was separated and the aqueous phase extracted with 2x25 ml dichloromethane. The organic phase was dried with $MgSO_4$ and concentrated under reduced pressure. The crude product was purified on a 27 cm silica column, eluting with 10% diethyl ether in toluene (R_f = 0.41 in 1:1 toluene/diethyl ether). The resulting oil was taken up in a small amount of methanol and concentrated under high vacuum to yield **L-S-Me** as a viscous yellow oil (0.199 g, 63% yield). ^{1}H NMR (400 MHz, CD_2Cl_2): δ = 8.70 (ddd, 2H, ^3J = 4.8 Hz, ^4J = 1.8 Hz, ^5J = 1.0 Hz, H_i), 8.42 (t, 1H, ^4J = 1.6 Hz, H_e), 7.99 (d, 2H, ^4J = 1.6 Hz, H_d), 7.85 (dt, 2H, ^3J = 8.0 Hz, ^4J = ^5J = 1.2 Hz, H_f), 7.80 (td, 2H, ^3J = 7.6 Hz, ^4J = 1.8 Hz, H_g), 7.28 (ddd, 2H, ^3J = 7.3 Hz, ^3J = 4.8 Hz, ^4J = 1.3 Hz, H_h), 2.63 (s, 3H, H_a). ^{13}C NMR (75 MHz, CD_2Cl_2): δ = 156.83, 150.08, 140.76, 140.30, 137.18, 125.48, 122.94, 122.57, 120.93, 16.09. EI-MS: calc for $C_{17}H_{14}N_2S$ $[M]^+$ m/z = 278.0878, found: 278.0875.

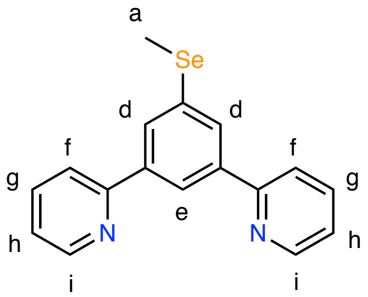

3,5-di(2-pyridyl)-selenoanisole (L-Se-Me). A solution of 1,3-bis(2-pyridyl)-5-bromo-benzene (0.353, 1.13 mmol) in tetrahydrofuran (50 ml) was cooled to -78°C in a dry ice-acetone bath, and n-butyl lithium (1.6M in hexanes, 1.05 ml, 1.7 mmol) was added dropwise. To the resulting black mixture was added dimethyldiselenide (0.16 ml, 1.7 mmol) and the mixture allowed to warm slowly to room temperature overnight. The resulting pale brown mixture was quenched by the addition of methanol (45 ml) and solvent removed under high vacuum. The crude product was taken up in 25 ml dichloromethane and poured into 25 ml water. This mixture was separated and the aqueous phase extracted with 2x25 ml dichloromethane. The organic phase was dried with $MgSO_4$ and concentrated under reduced pressure. The crude product was purified on a 28 cm silica column, eluting with 10% diethyl ether in toluene (R_f = 0.56 in 1:1 toluene/diethyl ether), to yield **L-Se-Me** as a viscous beige oil (0.203 g, 55% yield). ^{1}H NMR (400 MHz, CD_2Cl_2): δ = 8.70 (ddd, 2H, ^{3} = 4.8 Hz, ^4J = 1.8 Hz, ^5J = 1.0 Hz, H_i), 8.49 (t, 1H, ^4J = 1.6 Hz, H_e), 8.14 (d, 2H, ^4J = 1.6 Hz, H_d), 7.85 (dt, 2H, ^3J = 8.0 Hz, ^4J = ^5J = 1.2 Hz, H_f), 7.81 (td, 2H, ^3J = 7.6 Hz, ^4J = 1.8 Hz, H_g), 7.29 (ddd, 2H, ^3J = 7.3 Hz, ^3J = 4.8 Hz, ^5J = 1.3 Hz, H_h), 2.49 (s, 3H, $^2J_{1H\text{-}77Se}$ = 5.6

Hz, H_a). ^{13}C NMR (75 MHz, CD_2Cl_2): δ = 156.73, 150.09, 140.83, 137.19, 133.58, 129.19, 123.63, 122.95, 120.94, 7.50. ESI-MS: calc for $C_{17}H_{15}N_2S$ $[M+H]^+$ m/z = 327.0404, found: 327.0400.

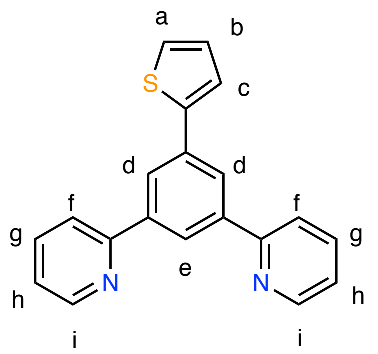

1,3-di(2-pyridyl)-5-(2-thienyl)-benzene (L-S-Ar). This compound was prepared following a modification of a previously reported synthesis.[4] A mixture of **P-S-Ar** (0.353 g, 1.11 mmol), 2-tributylstannyl-pyridine (1.304 g at 84% purity by ^1H NMR, 3.0 mmol), tetrakis(triphenylphosphine)palladium (0.103 g, 0.0891 mmol), and lithium chloride (0.467 g, 11.0 mmol) in 10 ml toluene was heated at reflux under nitrogen for 2 days. After cooling to room temperature, 10 ml of saturated aqueous potassium fluoride was added and the mixture stirred for 5.5 hours. The resulting black mixture was filtered, rinsed with dichloromethane, and the orange filtrate washed with 150 ml 50% saturated aqueous sodium bicarbonate. This mixture was separated, the aqueous phase extracted with dichloromethane (3x15 ml), and the combined organic phases back extracted into 1 M aqueous hydrochloric acid (3x25 ml). The acid layer was neutralized by addition of 1 M potassium hydroxide and the colorless suspension extracted with dichloromethane (3x25 ml). The combined organic layers were dried with potassium carbonate and concentrated to a colorless solid. The remaining impurities were removed by recrystallization from ethanol (20 ml) yielding **L-S-Ar** as a colorless solid (0.213 g, 61% yield). ^1H NMR (400 MHz, CD_2Cl_2): δ = 8.74 (ddd, 2H, ^3J = 4.8 Hz, ^4J = 1.8 Hz, ^5J = 0.9 Hz, H_i), 8.60 (t, 1H, ^4J = 1.6 Hz, H_e), 8.34 (d, ^4J = 1.6 Hz, H_d), 7.92 (dt, 2H, ^3J = 8.0 Hz, ^4J = ^5J = 1.0 Hz, H_f), 7.83 (td, 2H, ^3J = 7.7, ^4J = 1.8 Hz, H_g), 7.54 (dd, 1H, ^3J = 3.6 Hz, ^4J = 1.1 Hz, H_c), 7.38 (dd, 1H, ^3J = 5.1, ^4J = 1.1 Hz, H_a), 7.31 (ddd, 2H, ^3J = 7.4, 4.8 Hz, ^4J = 1.1 Hz, H_h), 7.16 (dd, 1H, ^3J = 5.1, 3.6 Hz, H_b). ESI-MS: calc for $C_{20}H_{15}N_2S$ $[M+H]^+$ m/z = 315.0960, found: 315.0956.

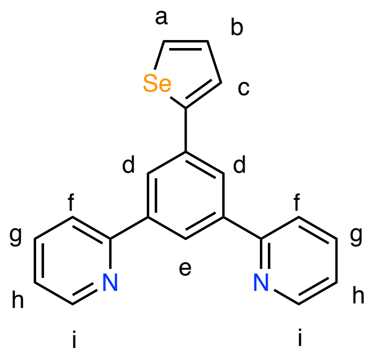

1,3-di(2-pyridyl)-5-(2-selenyl)-benzene (L-Se-Ar). A mixture of **P-Se-Ar** (0.404 g, 1.11 mmol), 2-tributylstannyl-pyridine (1.254 g at 84% purity, 2.8 mmol), tetrakis(triphenylphosphine)palladium (0.070 g, 0.061 mmol), and lithium chloride (0.461 g, 10.9 mmol) in 10 ml toluene was heated at reflux for 4 days. After cooling to room temperature, 10 ml of saturated aqueous potassium fluoride was added and the mixture stirred for 4.5 hours. The resulting black mixture was filtered, rinsed with toluene, and the orange-brown filtrate washed with 150 ml 50% saturated aqueous sodium bicarbonate. This mixture was separated, the aqueous phase extracted with dichloromethane (3x50 ml), and the combined organic phases back extracted into 1 M aqueous hydrochloric acid (3x25 ml). The acid layer was neutralized by addition of 1 M potassium hydroxide and the colorless suspension extracted with DCM (5x25 ml). The combined organic layers were dried with potassium carbonate and concentrated to a colorless solid. The remaining impurities were removed by column chromatography on alumina (gradient elution from petroleum ether to diethyl ether/ petroleum ether 6:4, R_f = 0.50 in 1:1 diethyl ether/petroleum ether) yielding **L-Se-Ar** as a colorless solid (0.267 g, 67% yield). It was observed that this product turned yellow upon prolonged storage in air, however this impurity could be removed by recrystallization from ethanol. ^{1}H NMR (400 MHz, CD_2Cl_2): δ = 8.74 (ddd, 2H, ^3J = 4.8 Hz, ^4J = 1.7 Hz, ^5J = 0.9 Hz, H_i), 8.60 (t, 1H, ^4J = 1.6 Hz, H_e), 8.29 (d, ^4J = 1.6 Hz, H_d), 8.04 (dd, 1H, $^2J_{1H\text{-}77Se}$ = 47.8 Hz, ^3J = 5.6 Hz, ^4J = 1.0 Hz, H_a), 7.91 (dt, 2H, ^3J = 8.0 Hz, ^4J = ^5J = 1.0 Hz, H_f), 7.83 (td, 2H, ^3J = 7.7, ^4J = 1.8 Hz, H_g), 7.70 (dd, 1H, ^3J = 3.8, ^4J = 1.1 Hz, H_c), 7.39 (dd, 1H, ^3J = 5.6, 3.8 Hz, H_b), 7.31 (ddd, 2H, ^3J = 7.4, 4.8 Hz, ^4J = 1.1 Hz, H_h). ^{13}C NMR (75 MHz, CD_2Cl_2): δ = 156.87, 150.78, 150.13, 140.96, 137.68, 137.21, 131.10, 130.89, 126.34, 125.55, 124.92, 122.98, 120.97. ESI-MS: calc for $C_{20}H_{15}N_2{}^{76}Se$ $[M+H]^+$ m/z = 359.0427, found: 359.0426.

General procedure to prepare X-Me methyl esters. A mixture of the appropriate **L-X-Me** ligand (1 equivalent) and mercuric acetate (1 equivalent) in 20 ml dry ethanol were heated to reflux. After 24 hours, lithium chloride (2.2 equivalents) was added as a solution in 20 ml methanol and the mixture returned to reflux for an additional 15 minutes. After cooling to room temperature, the mixture was poured into 100 ml water and filtered. The resulting colorless solid was rinsed

with water and a minimal amount of ice-cold methanol, dried in air, and transferred immediately to a mixture of [Ru(Me_3tctpy)Cl_3] (1 equivalent) and silver tetrafluoroborate (4 equivalents) in methanol. This mixture was heated to reflux in the dark for 4 hours. The resulting black mixture was concentrated, taken up in dichloromethane, filtered, and concentrated to a black solid. Initial purification was performed on a 15 cm silica column eluting as rapidly as possible with a 400:1 mixture of acetonitrile and 0.1M aqueous ammonium tetrafluoroborate. The resulting crude black solid was then purified twice on sephadex LH-20, eluting first with acetone, then with methanol. Finally, the complexes were dissolved in minimal dichloromethane and precipitated by the addition of diethyl ether, filtered, and washed with copious diethyl ether to afford **X-Me$_{Me}$** in high purity as fine black powders.

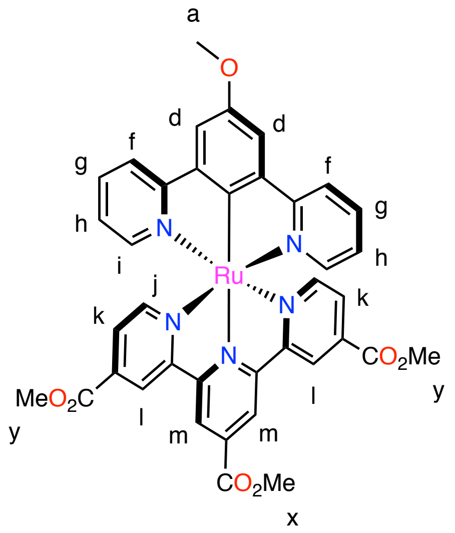

[Ru(L-O-Me)(Me_3tctpy)]BF_4 (O-Me$_{Me}$). 0.099 g (31% yield) ^{1}H NMR (400 MHz, CD_2Cl_2): δ = 9.35 (s, 2H, H_m), 8.95 (dd, 2H, ^{4}J = 1.8 Hz, ^{5}J = 0.8 Hz, H_l), 8.03 (ddd, 2H, ^{3}J = 8.2 Hz, ^{4}J = 1.4 Hz, ^{5}J = 0.8 Hz, H_f), 7.90 (s, 2H, H_d), 7.59 (ddd, 2H, ^{3}J = 8.1 Hz, ^{3}J = 7.4 Hz, ^{4}J = 1.6 Hz, H_g), 7.53 (dd, 2H, ^{3}J = 5.9 Hz, ^{4}J = 1.8 Hz, H_k), 7.50 (dd, 2H, ^{3}J = 5.9 Hz, ^{5}J = 0.8 Hz, H_j), 6.66 (ddd, 2H, ^{3}J = 5.7 Hz, ^{4}J = 1.6 Hz, ^{5}J = 0.8 Hz, H_i), 6.57 (ddd, 2H, ^{3}J = 7.4 Hz, ^{3}J = 5.7 Hz, ^{4}J = 1.4 Hz, H_h), 4.23 (s, 3H, H_x), 4.15 (s, 3H, H_a), 3.94 (s, 6H, H_y). ^{13}C NMR (100 MHz, CD_2Cl_2): δ = 207.18, 172.33, 168.75, 165.42, 164.33, 159.67, 157.74, 155.43, 153.59, 151.92, 141.24, 136.70, 136.05, 131.82, 126.17, 122.90, 122.71, 122.14, 120.67, 56.78. ^{19}F NMR (282 MHz, $CDCl_3$): δ = -154.06 (s, ^{10}B**F**$_4$), -154.11 (s, ^{11}B**F**$_4$). ESI-MS: calc for $C_{38}H_{30}N_5O_7{}^{96}Ru$ $[M-BF_4]^+$ m/z = 764.1222, found: 764.1221.

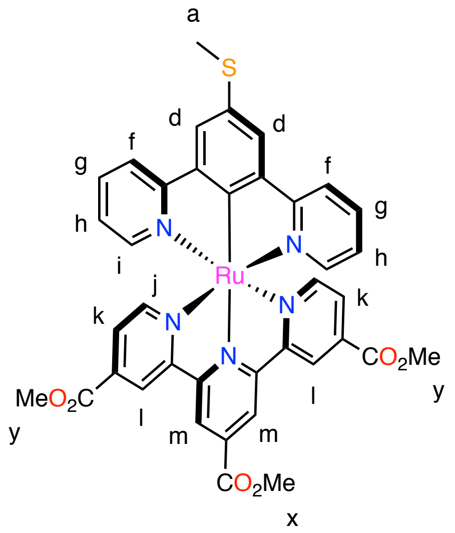

[Ru(L-S-Me)(Me_3tctpy)]BF_4 (S-Me_{Me}). 0.088 g (28% yield) ^{1}H NMR (400 MHz, CD_2Cl_2): δ = 9.36 (s, 2H, H_m), 8.96 (dd, 2H, ^4J = 1.8 Hz, ^5J = 0.8 Hz, H_l), 8.26 (s, 2H, H_d), 8.07 (d, 2H, ^3J = 8.1 Hz, H_f), 7.62 (ddd, 2H, ^3J = 8.1 Hz, ^3J = 7.4 Hz, ^4J = 1.6 Hz, H_g), 7.52 (dd, 2H, ^3J = 5.9 Hz, ^4J = 1.8 Hz, H_k), 7.48 (d, 2H, ^3J = 5.9 Hz, H_j), 6.72 (dd, 2H, ^3J = 5.7 Hz, ^4J = 0.9 Hz, H_i), 6.62 (ddd, 2H, ^3J = 7.4 Hz, ^3J = 5.7 Hz, ^4J = 1.4 Hz, H_h), 4.23 (s, 3H, H_x), 3.94 (s, 6H, H_y), 2.80 (s, 3H, H_a). ^{13}C NMR (100 MHz, CD_2Cl_2): δ = 215.72, 168.20, 165.33, 164.27, 159.56, 155.44, 153.30, 151.92, 142.04, 136.85, 136.31, 132.53, 130.96, 126.17, 125.84, 123.00, 122.77, 122.49, 120.67, 19.09. ^{19}F NMR (282 MHz, $CDCl_3$): δ = -154.14 (s, $^{10}BF_4$), -154.19 (s, $^{11}BF_4$). ESI-MS: calc for $C_{38}H_{30}N_5O_6{}^{96}RuS$ $[M-BF_4]^+$ m/z = 780.0991, found: 780.0993.

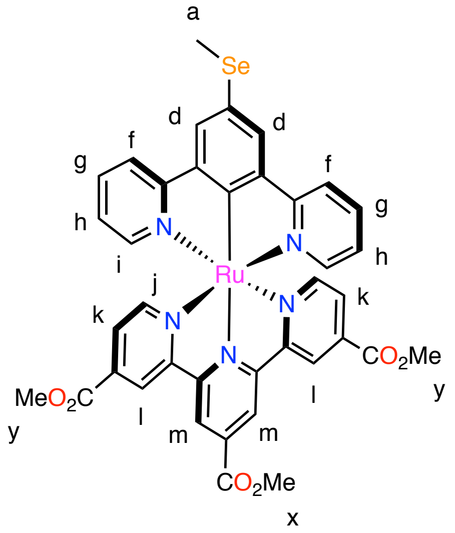

[Ru(L-Se-Me)(Me_3tctpy)]BF_4 (Se-Me_{Me}). 0.053 g (13% yield) ^{1}H NMR (400 MHz, CD_2Cl_2): δ = 9.36 (s, 2H, H_m), 8.96 (d, 2H, ^4J = 1.2 Hz, H_l), 8.39 (s, 2H, H_d), 8.06 (d, 2H, ^3J = 8.0 Hz, H_f), 7.61 (ddd, 2H, ^3J = 8.1 Hz, ^3J = 7.4, ^4J = 1.6 Hz, H_g), 7.53 (dd, 2H, ^3J = 5.8 Hz, ^4J = 1.8 Hz, H_k), 7.49 (d,

2H, ^3J = 5.9 Hz, H_j), 6.72 (ddd, 2H, ^3J = 5.7 Hz, ^4J = 1.6 Hz, ^5J = 0.8 Hz, H_i), 6.61 (ddd, 2H, ^3J = 7.0 Hz, ^3J = 5.6 Hz, ^4J = 1.4 Hz, H_h), 4.23 (s, 3H, H_x), 3.94 (s, 6H, H_y), 2.68 (s, 3H, H_a). ^{13}C NMR (100 MHz, CD_2Cl_2): δ = 216.49, 168.12, 165.31, 164.26, 159.52, 155.47, 153.25, 151.89, 142.25, 136.82, 136.31, 132.55, 128.65, 126.17, 123.59, 123.00, 122.75, 122.47, 120.66, 9.56. ^{19}F NMR (282 MHz, $CDCl_3$): δ = -154.01 (s, ^{10}**B**F_4), -154.06 (s, ^{11}**B**F_4). ESI-MS: calc for $C_{38}H_{30}N_5O_6{}^{96}Ru^{76}Se$ $[M\text{-}BF_4]^+$ m/z = 824.0468, found: 824.0464.

General procedure to prepare X-Ar methyl esters. A mixture of 5:1:1 methanol/water/ tetrahydrofuran (20 ml) was degassed by bubbling nitrogen for 1 hour. The appropriate **L-X-Ar** ligand (1 equivalent) and [Ru(Me_3tctpy)Cl_3] (1 equivalent) were then added and the mixture heated to reflux under nitrogen in the dark. After 16 hours, silver tetrafluoroborate (3 equivalents) was added as a solution in acetonitrile (5 ml) and the mixture returned to reflux for 2 hours. The resulting black mixture was concentrated to a black solid, and initial purification carried out on a 15 cm silica column eluting as rapidly as possible with a 400:1 mixture of acetonitrile and 0.1M aqueous ammonium tetrafluoroborate. The resulting crude black solid was then purified twice on sephadex LH-20, eluting first with acetone, then with methanol. Finally, the complexes were dissolved in minimal dichloromethane and precipitated by the addition of diethyl ether, filtered, and washed with copious diethyl ether to afford **X-Ar$_{Me}$** in high purity as fine black powders.

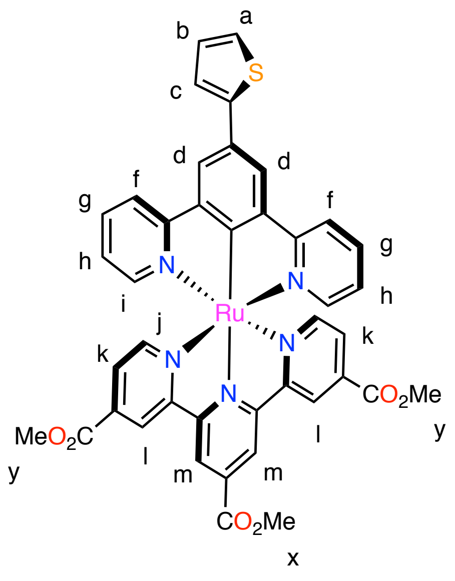

[Ru(L-S-Ar)(Me_3tctpy)]BF_4 (S-Ar$_{Me}$). 0.026 g (46% yield). ^{1}H NMR (400 MHz, CD_2Cl_2): δ = 9.37 (s, 2H, H_m), 8.97 (ddd, 2H, ^4J = 1.6 Hz, ^5J = 1.0 Hz, H_l), 8.48 (s, 2H, H_d), 8.15 (dt, 2H, ^3J = 8.0 Hz, ^4J = 1.1 Hz, H_f), 7.67-7.61 (m, 3H, H_a, H_g), 7.55-7.51 (m, 4H, H_j, H_k), 7.41 (dd, 2H, ^3J = 5.2 Hz, ^4J = 1.2 Hz, H_c), 7.26 (dd, 1H, ^3J = 5.2 Hz, ^3J = 3.5 Hz, H_b), 6.74 (ddd, ^3J = 5.7 Hz, ^4J = 1.6 Hz, ^5J = 0.8 Hz,

H_i), 6.64 (ddd, ^3J = 7.4 Hz, ^3J = 5.7 Hz, ^4J = 1.4 Hz, H_h), 4.24 (s, 3H, H_x), 3.94 (s, 6H, H_y). ^{13}C NMR (100 MHz, CD_2Cl_2): δ = 217.95, 168.48, 165.31, 164.26, 159.52, 155.49, 153.24, 151.92, 145.91, 141.82, 136.86, 136.38, 132.64, 129.32, 128.76, 126.22, 124.38, 123.04, 122.82, 122.78, 122.53, 122.36, 120.75. ^{19}F NMR (282 MHz, $CDCl_3$): δ = -154.11 (s, ^{10}**B**F_4), -154.16 (s, ^{11}**B**F_4). ESI-MS: calc for $C_{41}H_{30}N_5O_6{}^{96}RuS$ $[M-BF_4]^+$ m/z = 816.0993, found: 816.0991.

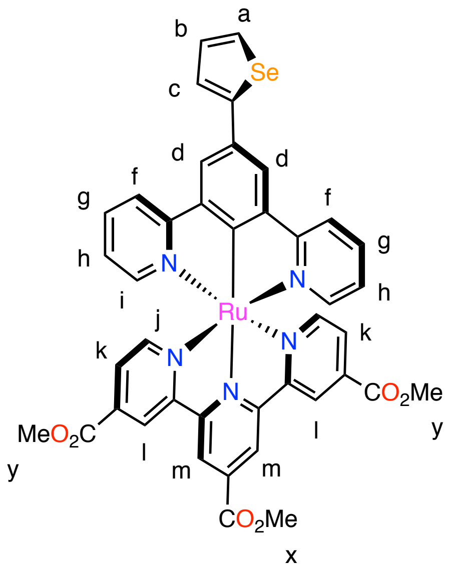

[Ru(L-Se-Ar)(Me₃tctpy)]BF₄ (Se-Ar$_{Me}$). 0.025 g (41% yield). ^1H NMR (400 MHz, CD_2Cl_2): δ = 9.37 (s, 2H, H_m), 8.97 (dd, 2H, ^4J = 1.6 Hz, ^5J = 1.0 Hz, H_l), 8.43 (s, 2H, H_d), 8.15 (ddd, 2H, ^3J = 8.2 Hz, ^4J = 1.4 Hz, ^5J = 0.8 Hz, H_f), 8.05 (dd, 1H, ^3J = 5.6 Hz, ^4J = 1.1 Hz, $^2J_{1H-77Se}$ = 23.9 Hz, H_a), 7.79 (dd, 1H, ^3J = 3.7 Hz, ^4J = 1.1 Hz, H_c), 7.64 (ddd, 2H, ^3J = 8.2 Hz, ^3J = 7.4 Hz, ^4J = 1.6 Hz, H_g), 7.55-7.51 (m, 4H, H_j, H_k), 7.49 (dd, 2H, ^3J = 5.6 Hz, ^3J = 3.7 Hz, H_b), 6.74 (ddd, 2H, ^3J = 5.7 Hz, ^4J = 1.6 Hz, ^5J = 0.8 Hz, H_i), 6.64 (ddd, 2H, ^3J = 7.4 Hz, ^3J = 5.7 Hz, ^4J = 1.4 Hz, H_h), 4.24 (s, 3H, H_x), 3.94 (s, 6H, H_y). ^{13}C NMR (100 MHz, CD_2Cl_2): δ = 218.25, 168.44, 165.31, 164.26, 159.52, 155.49, 153.24, 152.28, 151.94, 141.83, 136.87, 136.41, 132.70, 131.38, 131.30, 129.49, 126.22, 124.88, 123.05, 122.79, 122.71, 122.55, 120.74. ^{19}F NMR (282 MHz, $CDCl_3$): δ = -154.12 (s, ^{10}**B**F_4), -154.17 (s, ^{11}**B**F_4). ESI-MS: calc for $C_{41}H_{30}N_5O_6{}^{96}Ru^{76}Se$ $[M-BF_4]^+$ m/z = 860.0464, found: 860.0474.

General procedure to produce saponified catalysts. An approximately 5 mM solution of the **X-Me** or **X-Ar** methyl esters in a mixture of dimethyl formamide, water, and triethylamine (3:1:1) was brought to reflux for 4 hours. The solution was then concentrated to a black solid, suspended in dichloromethane and collected by vacuum filtration to yield the corresponding free acid compound as a black powder. The protonation state of the as-prepared compounds was ambiguous, though the compounds were presumed to be in their doubly protonated

zwitterionic state based on their solubility properties. The products were only sparingly soluble, and were therefore analyzed in alkaline methanol-d_4 as their deprotonated analogues.

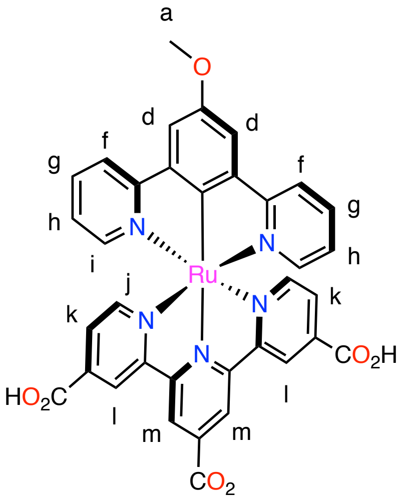

[Ru(L-O-Me)($H_x$tctpy)] O-Me$_H$. 0.026 g (85% yield, assuming zwitterionic product). ^{1}H NMR (400 MHz, 0.1M $NaOCD_3/CD_3OD$): δ = 9.32 (s, 2H, H_m), 8.93 (d, 2H, ^4J = 1.7 Hz, H_l), 8.17 (d, 2H, ^3J = 8.1 Hz, H_f), 8.01 (s, 2H, H_d), 7.60 (td, 2H, ^3J = 7.8 Hz, ^4J = 1.5 Hz, H_g), 7.38 (dd, 2H, ^3J = 5.8 Hz, ^4J = 1.7 Hz, H_k), 7.20 (d, 2H, ^3J = 5.8 Hz, H_j), 6.95 (d, 2H, ^3J = 5.6 Hz, H_i), 6.64 (ddd, 2H, ^3J = 7.1 Hz, ^3J = 5.9 Hz, ^4J = 1.2 Hz, H_h), 4.14 (s, 3H, H_a).

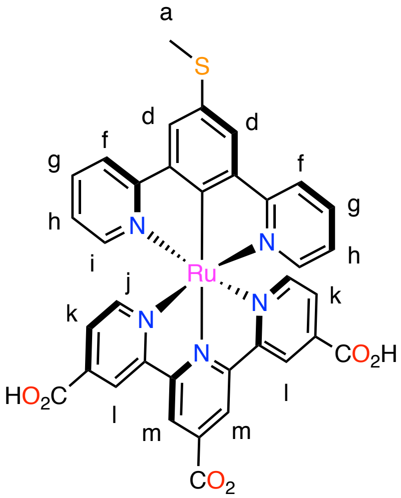

[Ru(L-S-Me)($H_x$tctpy)] S-Me$_H$. 0.021 g (73% yield, assuming zwitterionic product). ^{1}H NMR (400 MHz, 0.1M $NaOCD_3/CD_3OD$): δ = 9.33 (s, 2H, H_m), 8.94 (d, 2H, ^4J = 1.3 Hz, H_l), 8.36 (s, 2H, H_d), 8.22 (d, 2H, ^3J = 8.1 Hz, H_f), 7.63 (td, 2H, ^3J = 7.8 Hz, ^4J = 1.5 Hz, H_g), 7.37 (dd, 2H, ^3J = 5.8 Hz, ^4J = 1.7 Hz, H_k), 7.18 (d, 2H, ^3J = 5.6 Hz, H_j), 7.01 (d, 2H, ^3J = 5.5 Hz, H_i), 6.69 (ddd, 2H, ^3J = 7.4 Hz, ^3J = 5.7 Hz, ^4J = 1.4 Hz, H_h), 2.77 (s, 3H, H_a).

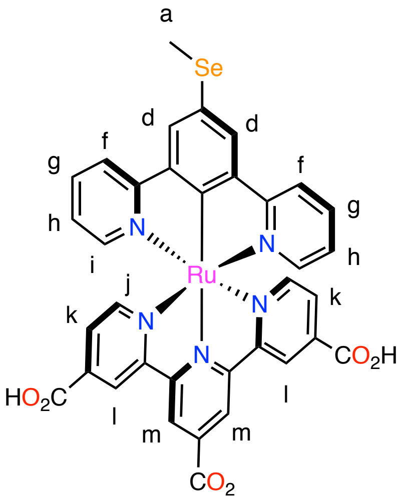

[Ru(L-Se-Me)($H_x$tctpy)] Se-Me$_H$. 0.019 g (77% yield, assuming zwitterionic product). ^{1}H NMR (400 MHz, 0.1M $NaOCD_3/CD_3OD$): δ = 9.33 (s, 2H, H_m), 8.94 (d, 2H, ^4J = 1.7 Hz, H_l), 8.47 (s, 2H, H_d), 8.22 (d, 2H, ^3J = 8.1 Hz, H_f), 7.63 (td, 2H, ^3J = 7.8 Hz, ^4J = 1.5 Hz, H_g), 7.37 (dd, 2H, ^3J = 5.8 Hz, ^4J = 1.7 Hz, H_k), 7.18 (d, 2H, ^3J = 5.8 Hz, H_j), 7.01 (d, 2H, ^3J = 5.7 Hz, H_i), 6.69 (ddd, 2H, ^3J = 7.4 Hz, ^3J = 5.7 Hz, ^4J = 1.4 Hz, H_h), 2.62 (s, 3H, $^2J_{1H\text{-}77Se}$ = 5.3 Hz, H_a).

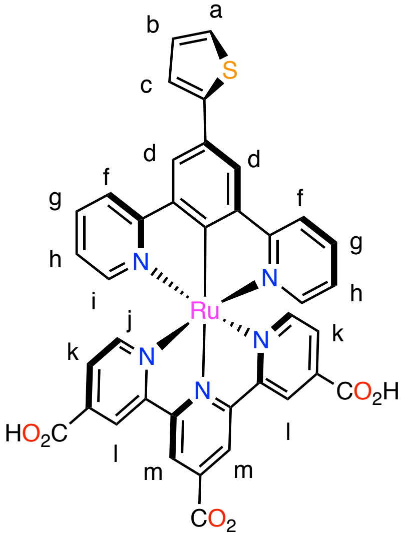

[Ru(L-S-Ar)(H_2tctpy)] S-Ar$_H$. 0.041 g (93% yield, assuming zwitterionic product). ^{1}H NMR (400 MHz, 0.1M $NaOCD_3/CD_3OD$): δ = 9.34 (s, 2H, H_m), 8.95 (d, 2H, ^4J = 1.1 Hz, H_l), 8.56 (s, 2H, H_d), 8.29 (d, 2H, ^3J = 8.0 Hz, H_f), 7.69 (dd, 1H, ^3J = 3.6 Hz, ^4J = 1.1 Hz, H_a), 7.65 (ddd, 2H, ^3J = 8.0 Hz, ^3J = 7.5 Hz, ^4J = 1.6 Hz, H_g), 7.42 (dd, 1H, ^3J = 5.2 Hz, ^4J = 1.1 Hz, H_c), 7.38 (dd, 2H, ^3J = 5.8 Hz, ^4J = 1.7 Hz, H_k), 7.25 (d, 2H, ^3J = 5.8 Hz, H_j), 7.22 (dd, 1H, ^3J = 5.2 Hz, ^3J = 3.5 Hz, H_b), 7.02 (d, 2H, ^3J = 5.2 Hz, H_i), 6.71 (ddd, 2H, ^3J = 7.4 Hz, ^4J = 5.7 Hz, ^5J = 1.4 Hz, H_h).

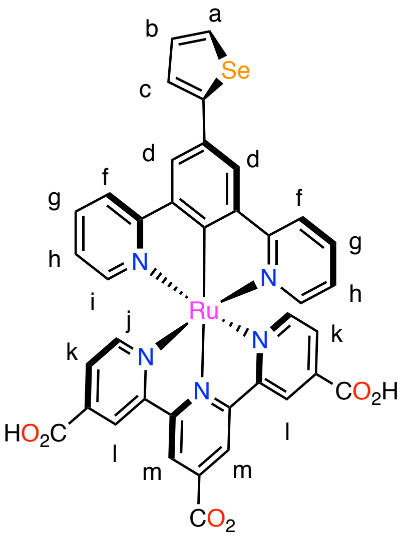

[Ru(L-Se-Ar)(H_2tctpy)] Se-Ar$_H$. 0.028 g (91% yield, assuming zwitterionic product). ^1H NMR (400 MHz, 0.1M $NaOCD_3/CD_3OD$): δ = 9.34 (s, 2H, H_m), 8.94 (d, 2H, ^4J = 1.6 Hz, H_l), 8.51 (s, 2H, H_d), 8.29 (d, 2H, ^3J = 8.1 Hz, H_f), 8.05 (dd, 1H, ^3J = 5.6 Hz, ^4J = 0.7 Hz, $^2J_{1H\text{-}77Se}$ = 24.1 Hz, H_a), 7.82 (dd, 1H, ^3J = 3.8 Hz, ^4J = 0.7 Hz, H_c), 7.65 (td, 2H, ^3J = 7.7 Hz, ^4J = 1.3 Hz, H_g), 7.44 (dd, 1H, ^3J = 5.6 Hz, ^3J = 3.8 Hz, H_b), 7.38 (dd, 2H, ^3J = 5.8 Hz, ^4J = 1.7 Hz, H_k), 7.25 (d, 2H, ^3J = 5.8 Hz, H_j), 7.03 (d, 2H, ^3J = 5.5 Hz, H_i), 6.71 (ddd, 2H, ^3J = 7.1 Hz, ^3J = 6.0 Hz, ^4J = 1.0 Hz H_h).

<u>Preparation of functionalized metal oxide thin films</u>

In_2O_3:Sn (ITO) nanoparticles (TC8 DE; 30 wt % dispersion in ethanol) were purchased from Evonik Industries and deposited in thin films following literature procedures.[12] Briefly, a small aliquot of the 30 wt % ITO dispersion was sonicated for 20 minutes in a sonication bath and diluted to 10 wt % by addition of hydroxypropyl cellulose suspension in ethanol (10 wt %). The resulting mixture was stirred at room temperature overnight prior to use. This mixture was deposited on fluorine-doped tin oxide coated glass (FTO, Hartford Glass Co. Inc., 2.3 mm thick, 15 Ω cm^{-2}) by doctor blading masked with Scotch™ tape. These films were then sintered under air at 450°C for 1 hour.

Optically transparent TiO_2 thin films were prepared following literature procedures.[13] In short, 0.42 ml concentrated nitric acid was dissolved in 60 ml deionized water in a 125 ml erlenmeyer flask. The flask was covered with aluminum foil and 10 ml of titanium(IV) isopropoxide was added dropwise over the course of 20 minutes with vigorous stirring. The flask was then heated and stirred in a water bath at ~95°C for 6 hours, resulting in an opaque pale blue mixture. Excess water was allowed to boil away until the volume was reduced to 20 ml. The mixture was transferred to a Teflon-lined stainless steel acid digestion bomb and heated to 200°C for 12 hours. The resulting viscous opaque white mixture was allowed to cool

to nearly room temperature, and, while the mixture was still warm to the touch, 1 g of ground polyethyleneglycol bisphenol A epichlorohydrin copolymer (mol wt. 15,000-20,000 Da) was added with stirring. The final mixture was transferred to a glass vial protected from light and stirred for 24 hours prior to use. This mixture could be stored for several months maintaining constant stirring. This mixture was deposited on fluorine-doped tin oxide coated glass (FTO, Hartford Glass Co. Inc., 2.3 mm thick, 15 Ω cm^{-2}) by doctor blading masked with Scotch™ tape. After allowing to dry in air for 30 minutes, the films were sintered under oxygen at 450°C in a tube furnace for 30 minutes.

The SnO_2-TiO_2 core-shell thin films were prepared by deposition of a SnO_2 mesoporous thin film, followed by sintering, and finally atomic-layer deposition of a 4.5 nm amorphous TiO_2 layer following literature procedures.[14] In brief, a colloidal suspension of SnO_2 (15 wt % in water) was acidified by the addition of glacial acetic acid (1 ml acid per 30 ml dispersion). After stirring overnight, this mixture was heated in a pressure vessel to 240°C for 60 hours. This mixture was then cooled to room temperature, sonicated in a sonication bath, and 2.5 wt % each of polyethylene oxide (mol wt. 100,000 Da) and polyethylene glycol (mol wt. 12,000 Da) were added. After stirring for 12 hours, this mixture was deposited on fluorine-doped tin oxide coated glass (FTO, Hartford Glass Co. Inc., 2.3 mm thick, 15 Ω cm^{-2}) by doctor blading masked with Scotch™ tape. These films were sintered under oxygen at 450°C in a tube furnace for 30 minutes. The TiO_2 shell was applied by atomic-layer deposition using a Savannah S200 reactor (Cambridge Nanotech) with tetrakis(dimethylamido)titanium and water. The reactor temperature was 130°C and the tetrakis(dimethylamido)titanium reservoir was 75°C during deposition. Each 0.6 Å TiO_2 layer was deposited with the following pulse sequence: 0.3 seconds Ti precursor dose, 10 second hold, 20 second N_2 purge, 0.02 second water dose, 10 second hold, 20 second N_2 purge. This sequence was repeated until a 4.5 nm TiO_2 layer was deposited.

The metal-oxide thin films described above were functionalized by immersion in saturated ethanol solutions of the saponified catalysts overnight in the dark. The functionalized films were washed thoroughly with ethanol and stored in neat acetonitrile until use.

<u>Computational methods</u>

Density functional theory (DFT) calculations were carried out with the Gaussian 16 computational package.[15] Except where noted, all calculations were performed using using the PBE0 functional[16] with an ultrafine integration grid (99 radial shells, 590 angular points) in SMD modeled acetonitrile.[17] Ruthenium complex models for molecular orbital and UV-vis analysis were generated using the cc-pVDZ-PP basis set on ruthenium and cc-pVDZ on all other atoms.[18-24] All structures were optimized to a minimum and frequency calculations performed at the same level of theory to verify the absence of negative frequencies. Atomic coordinates of the optimized structures and their energies are listed in the Supplementary Data 1 file associated

with this manuscript. Molecular orbitals were visualized at an iso value of 0.05 from the formatted checkpoint files using the GaussView 5 software package.[25] UV-vis spectra were modeled using time-dependent density functional theory (TD-DFT) methods to calculate the 50 lowest energy singlet transitions.[26-32] Electron density difference maps (EDDMs) were generated using the GaussSum 3.0 software package.[33] Individual atom contributions ($c_{A,X}$) to molecular orbitals were determined using Hirshfeld population analysis[34-36] performed using the MultiWFN software package.[37,38]

Additional models to investigate the $cat^{+}\cdots I^{-}$ adduct were optimized and analyzed for negative frequencies using similar methods to those specified above, but also including Grimme's D3 dispersion correction[39] and using aug-cc-pVDZ on the interacting O, S, or Se atom and aug-cc-pVDZ-PP on the iodide ion.[18-24,40] Subsequent calculations were performed using the cc-pVTZ-PP basis set on ruthenium, aug-cc-pVTZ-PP on iodine, aug-cc-pVTZ on the interacting chalcogen atom, and cc-pVTZ on all other atoms.[18-24,40] To ensure that charges were distributed appropriately on the interacting pairs, a fragment guess was first generated specifying a negative charge on iodide and all subsequent calculations were performed reading their initial guess from a checkpoint file based off this fragment guess. The basis set superposition error (BSSE) for the $cat^{+}\cdots I^{-}$ adduct was estimated in the gas phase using the counterpoise method for the interacting dimers.[41-44]

Parameters for the cc-pVDZ-PP, cc-pVTZ-PP, aug-cc-pVDZ-PP, and aug-cc-pVTZ-PP basis sets were obtained from the ESML basis set exchange.[45,46]

To explore the impact of different functionals on our results, additional geometry optimized structures were prepared as described above substituting B3LYP,[47-50] M06,[51,52] mPW1PW91,[53-55] or BP86[56,57] for the PBE0 functional, as indicated. As before, values for $c_{A,X}$ were determined using a Hirshfeld population analysis[34-36] and the reorganization energies ($\boldsymbol{\lambda}$) were calculated as described in the Supplementary Discussion below.

Data Availability

The data supporting these findings are available from the corresponding authors upon request.

Supplementary Discussion

<u>Stepwise equilibrium constant (K_A)</u>

In principal, the observed second-order electron transfer rate, k_{IET}, is related to the first-order electron transfer rate, k_{Marcus}, by an equilibrium constant, K_A:[58]

$$k_{IET} = K_A k_{Marcus} \tag{1}$$

As mentioned in the main text, the iodide oxidation reaction under investigation is formally a trimolecular reaction, however it is believed to follow a two-step mechanism of sequential bimolecular reaction.[59-62] The overall observed iodide oxidation process can therefore be separated into individual physicochemical reaction steps represented by the scheme:

$$cat^{+} + I^{-} \underset{k_{1r}}{\overset{k_{1f}}{\rightleftharpoons}} cat^{+}\cdots I^{-} + I^{-} \underset{k_{2r}}{\overset{k_{2f}}{\rightleftharpoons}} cat^{+}\cdots I^{-}\cdots I^{-} \xrightarrow{k_{Marcus}} cat\cdots I_2^{\bullet-} \xrightarrow{k_{diss}} cat + I_2^{\bullet-} \tag{2}$$

Where k_{1f} and k_{1r} are the rate constants of association/dissociation of the $cat^{+}\cdots I^{-}$ adduct, k_{2f} and k_{2r} are the rate constants of association/dissociation of the $cat^{+}\cdots I^{-}\cdots I^{-}$ encounter complex, k_{Marcus} is the rate of electron transfer from diiodide to cat^{+} in the encounter complex, and k_{diss} is the rate of dissociation of the products after electron transfer. In this analysis, it is assumed that the product $cat\cdots I_2^{\bullet-}$ complex is unstable and will dissociate on the time scale of vibrational motion, k_{diss} ~10^{13} s^{-1}.[63] Since, in this condition, k_{diss} is likely much larger than k_{Marcus}, and we can therefore consider the final electron transfer step to be irreversible.

In our transient absorption experiments, our observed signal comes from the spectroscopic signatures of the oxidized catalyst molecules on the TiO_2 surface. During the course of iodide oxidation, these oxidized catalysts form multiple different complexes with iodide such that the total surface concentration of oxidized catalysts, in all forms, can be expressed as:

$$\chi_{cat+,tot} = \chi_{cat+} + \chi_{cat\cdot I} + \chi_{cat\cdot I\cdot I} \tag{3}$$

Where χ_{cat+}, $\chi_{cat\cdot I}$, and $\chi_{cat\cdot I\cdot I}$ are the surface fractions of free oxidized catalyst, the $cat^{+}\cdots I^{-}$ adduct, and the $cat^{+}\cdots I^{-}\cdots I^{-}$ encounter complex, respectively. The rate of disappearance of the oxidized catalysts is given by:

$$rate_{obs} = \frac{d\chi_{cat+,tot}}{dt} = k_{obs}\chi_{cat+,tot} \tag{4}$$

Where k_{obs} is the observed pseudo-first order rate constant from our transient absorption experiments at a known iodide concentration. This pseudo-first order rate constant is related to the second order rate constant for intermolecular electron transfer, k_{IET}, by the concentration of iodide, [I⁻]:

$$k_{obs} = k_{IET}[I^-] \tag{5}$$

Because the electron transfer step in the reaction scheme represented by Supplementary Equation 2 is the only irreversible step before product dissociation, Supplementary Equation 4 can be rewritten as:

$$rate_{obs} = k_{obs}\chi_{cat+,tot} = k_{Marcus}\chi_{cat\cdot I\cdot I} \tag{6}$$

Assuming the law of mass action, the surface fractions of all oxidized catalyst species as a function of time are given as:

$$\frac{d\chi_{cat+}}{dt} = -k_{1f}\chi_{cat+}[I^-] + k_{1r}\chi_{cat\cdot I} \tag{7}$$

$$\frac{d\chi_{cat\cdot I}}{dt} = k_{1f}\chi_{cat+}[I^-] - k_{1r}\chi_{cat\cdot I} - k_{2f}\chi_{cat\cdot I}[I^-] + k_{2r}\chi_{cat\cdot I\cdot I} \tag{8}$$

$$\frac{d\chi_{cat\cdot I\cdot I}}{dt} = k_{2f}\chi_{cat\cdot I}[I^-] - k_{2r}\chi_{cat\cdot I\cdot I} - k_{Marcus}\chi_{cat\cdot I\cdot I} \tag{9}$$

In order to simplify this series of competing rates, we can make several reasonable assumptions regarding the relative rates of these processes. First, given that $cat^+\cdots I^-$ and $cat^+\cdots I^-\cdots I^-$ are effectively electrostatic interactions and no major chemical transformations occur in their formation, we can assume that the association/dissociation reactions represented by k_{1f}/k_{1r} and k_{2f}/k_{2r} are effectively barrier-less beyond their inherent free energy. Next, we can assume that the formation of the $cat^+\cdots I^-\cdots I^-$ encounter complex is unfavorable compared to the $cat^+\cdots I^-$ adduct because electrostatic forces encourage the formation of the latter, but impede the formation of the former. This notion implies in turn that the $cat^+\cdots I^-$ adduct will reach equilibrium with free ions quickly on the time-scale of $cat^+\cdots I^-\cdots I^-$ encounter complex formation. Finally, because the $cat^+\cdots I^-\cdots I^-$ encounter complex is inherently unstable, it will dissociate back to $cat^+\cdots I^-$ and free I^- on the time scale of molecular vibrations (k_{2r} ~10^{13} s^{-1})[63] such that $k_{2r} >> k_{Marcus}$, implying that the any $cat^+\cdots I^-\cdots I^-$ encounter complexes that form are far more likely to dissociate rather than undergo electron transfer. Taking these three assumptions together, we can consider χ_{cat+}, $\chi_{cat\cdot I}$, and $\chi_{cat\cdot I\cdot I}$ to rapidly achieve equilibrium with each other on the time-scale of electron transfer:

$$K_1 = \frac{k_{1f}}{k_{1r}} = \frac{\chi_{cat\cdot I}}{\chi_{cat+}[\mathrm{I}^-]} \tag{10}$$

$$K_2 = \frac{k_{2f}}{k_{2r}} = \frac{\chi_{cat\cdot I\cdot I}}{\chi_{cat\cdot I}[\mathrm{I}^-]} \tag{11}$$

Knowing that, under our experimental conditions, $[\mathrm{I}^-] >> \chi_{cat+,tot}$, it follows that the formation of $cat^+\cdots I^-$ and $cat^+\cdots I^-\cdots I^-$ will not appreciably affect $[\mathrm{I}^-]$. With this in mind, we can combine Supplementary Equations 10 and 11 with Supplementary Equation 3 to arrive at:

$$\chi_{cat+,tot} = \frac{1}{K_1K_2}\frac{\chi_{cat\cdot I\cdot I}}{[\mathrm{I}^-]^2} + \frac{1}{K_2}\frac{\chi_{cat\cdot I\cdot I}}{[\mathrm{I}^-]} + \chi_{cat\cdot I\cdot I} \tag{12}$$

By applying Supplementary Equation 6 and solving for $\chi_{cat\cdot I\cdot I}$, we obtain an expression relating k_{obs} directly to k_{Marcus}:

$$k_{obs} = \frac{k_{Marcus}[\mathrm{I}^-]^2}{\frac{1}{K_1K_2} + \frac{[\mathrm{I}^-]}{K_2} + [\mathrm{I}^-]^2} \tag{13}$$

Using Supplementary Equation 13 in conjunction with Supplementary Equation 5 gives us an expression for K_A:

$$K_A = \frac{[\mathrm{I}^-]}{\frac{1}{K_1K_2} + \frac{[\mathrm{I}^-]}{K_2} + [\mathrm{I}^-]^2} \tag{14}$$

The first equilibrium constant, K_1, represents the formation of a catalyst-iodide adduct. These adducts have been modeled by DFT and the optimized structures are visualized in Supplementary Figure 13. The electron stabilization energy (ΔE_{int}) from the formation of these adducts was obtained from:

$$\Delta E_{int} = E_{cat\cdot I} - (E_{cat+} + E_{I-}) \tag{15}$$

Where $E_{cat\cdot I}$ is the electronic energy of the adduct, and E_{cat+} and E_{I-} are the electronic energies of the free oxidized catalysts and the free iodide ion, respectively. Using our computational methods, E_{I-} was found to be -295.906658157 Hartrees, and the values for E_{cat+} and $E_{cat\cdot I}$ can be found in the Supplementary Data 1 file associated with this manuscript along with their corresponding molecular coordinates. The values of ΔE_{int} for each catalyst compound are

presented in Supplementary Table 3. If we assume the free energy of adduct formation (ΔG_{int}) to be approximately equal to the electronic stabilization energy, then we can calculate K_1 at T = 298 K from the free energy expression:

$$K_1 = e^{-\left(\frac{\Delta G_{\text{int}}}{RT}\right)} \tag{16}$$

The values of K_1 obtained by this method are presented in Supplementary Table 3.

The second equilibrium constant, K_2, represents the approach of a second iodide to a contact distance with the iodide in the cat$^+\cdots$I$^-$ adduct, forming an encounter complex. To obtain this value, we have employed the model of N. Sutin for the association constant of free ions in solution (K_{ion}) with center-to-center distances in the range of r to r+δr:[64]

$$K_2 = K_{ion}(r) = \frac{4\pi S N_A r^2 \delta r}{1000} e^{\left(\frac{-w(r)}{RT}\right)} \tag{17}$$

Where S is a steric correction factor accounting for restricted access to one of the ions, N_A is Avogadro's number, $w(r)$ is a function defining the thermodynamic work (vide infra), and the remaining terms have their standard definitions. For the purposes of this analysis, it was assumed that electron transfer occurs when the iodide ions are at a contact distance defined twice their ionic radii (r = 4.24 Å), and δr was set at r/3, a value which has been shown to give reasonable values for ion association.[65] Because the cat$^+\cdots$I$^-$ adduct is anchored to a surface, it was assumed that the second iodide equivalent could only approach from half of the spherical volume surrounding the adduct, and therefore S = ½ was used. In Supplementary Equation 17, $w(r)$ represents the thermodynamic work of bringing two ions in solution from an infinite distance to a center-to-center distance of r, and is given by:[64,66]

$$w(r) = \frac{z_1 z_2 \varepsilon^2}{2D_S r}\left(\frac{e^{\left(\sigma_1\sqrt{\frac{8\pi\mu N_A \varepsilon^2}{D_S k_B T}}\right)}}{1+\sigma_1\sqrt{\frac{8\pi\mu N_A \varepsilon^2}{D_S k_B T}}} + \frac{e^{\left(\sigma_2\sqrt{\frac{8\pi\mu N_A \varepsilon^2}{D_S k_B T}}\right)}}{1+\sigma_2\sqrt{\frac{8\pi\mu N_A \varepsilon^2}{D_S k_B T}}}\right) e^{\left(-r\sqrt{\frac{8\pi\mu N_A \varepsilon^2}{D_S k_B T}}\right)} \tag{18}$$

Where z_1 and z_2 are the charges of the two ions, σ_1 and σ_2 are the radii of two ions (2.12 Å for iodide), $\boldsymbol{\varepsilon}$ is the elementary charge, D_S is the static absolute permittivity of the solvent (D_S = 5.06x10^{-39} C^2 eV^{-1} Å^{-1} for acetonitrile), μ is the ionic strength of the solvent (0.5 M in the current study), and the remaining terms have their standard definitions. Using Supplementary Equations 17 and 18, and assuming that the work term is entirely due to two iodides coming into contact, we obtain a value of K_2 = 3.64x10^{-5} M^{-1}.

Using the values for K_1 and K_2 in Supplementary Equation 14 at a defined iodide concentration (0.2 M, in this case), values for K_A can be calculated (Supplemental Table 3).

Free energy for electron transfer ($\Delta G°_{ET}$)

The values for $\Delta G°_{ET}$ are presented in Supplementary Table 3, and were obtained from the expression:

$$\Delta G^{\circ}_{ET} = -n_e F E_{cell} \tag{19}$$

Where n_e is the number of electrons passed, F is Faraday's constant, and E_{cell} is the difference between the redox potentials of the catalysts and the redox potential of the $I_2^{\bullet-}/2I^-$ couple (0.79 V vs NHE).[59]

Reorganization energy (λ)

Traditionally, the reorganization energy is divided into separate terms for bond reorganization of the donor-acceptor molecules (λ_i) and solvent reorganization (λ_o):

$$\lambda = \lambda_i + \lambda_o \tag{20}$$

Both terms can be evaluated simultaneously using DFT methods.[67,68] By taking the differences in electronic energies of the reactant pair before electron transfer at their optimized geometries, with an equilibrium PCM-modeled solvent shell, and at their non-equilibrium geometry (defined as the optimized geometries of the product pair after electron transfer), with the inertial charges of the solvent set to values corresponding to the non-equilibrium geometry, we can obtain a reasonable estimation of the overall λ value. In order to simplify these calculations, λ can instead be divided into individual terms treating the electron donor and acceptor separately:

$$\lambda = \lambda_{cat+} + \lambda_{2I-} \tag{21}$$

Where λ_{cat+} is the reorganization energy of the catalysts and λ_{2I-} is the reorganization energy of the iodide ions. Using the DFT models developed for the **X-Me** and **X-Ar** series, λ_{cat+} was determined to be between 0.78 and 0.83 eV for all compounds. The value for λ_{2I-} was taken from literature as 0.522 eV.[68] Combining these values, we obtain the total λ for iodide oxidation (Supplementary Table 3).

Formal overlap integral ($S°_{DA}$)

In order to evaluate our orbital pathway hypothesis, it was necessary to estimate the overlap integral between isolated iodide and chalcogen atoms. During the electron transfer

reaction, the geometry of the transient pre-electron transfer [Ox···I⁻···I⁻] encounter complex is unknown, however there are only two relevant interactions: either the iodide interacts with the chalcogen in the plane of the beta-LUMO, whereupon the chalcogen-iodide orbital overlap relevant to electron transfer is a pi-type overlap between the valence p-orbitals of both atoms, or the iodide interacts orthogonal to the beta-LUMO, whereupon the relevant overlap will be sigma-type. If we assume that both interactions are possible, collision theory states that both will occur and therefore the larger overlap will dominate the electron transfer mechanism. At a fixed inter-nuclear distance, this will necessarily be the sigma-type interaction, and therefore, for the purpose of calculating $S°_{DA}$, the relevant overlap is assumed to be sigma-type at the van der Waals distance between the chalcogen and iodide. Using these assumptions, the integral can be calculated as the overlap between Slater-type orbitals of two atoms a and b at a distance R using equations developed by Mulliken.[69] To simplify the equations, these integrals are defined in terms of spheroidal coordinates $\xi=(r_a+r_b)/R$ and $\eta=(r_a-r_b)/R$, where r_a and r_b are the distances from a point in space to atoms a and b, respectively, and R is the separation between a and b. For a sigma-type interaction between p-orbitals with principal quantum number n, the equation for the overlap integral takes the form:

$$S(p,t)=\frac{3N_aN_b}{2}\left(\frac{R}{2}\right)^{m_a+m_b+1}\int_1^{\infty}\int_{-1}^{1}(\xi+\eta)^{m_a-2}(\xi-\eta)^{m_b-2}(\xi^2\eta^2-1)(\xi^2-\eta^2)e^{-p(\xi+\eta t)}\,d\eta\, d\xi \qquad (22)$$

where

$$p=\frac{(\mu_a+\mu_b)R}{2a_H} \qquad (23)$$

$$t=\frac{(\mu_a-\mu_b)}{(\mu_a+\mu_b)} \qquad (24)$$

and the variables m_j, N_j, and μ_j are defined for a given atom j by:

$$m_j=n-\delta \qquad (25)$$

$$N_j=\sqrt{\frac{1}{\int_0^{\infty}r^{2n-2\delta}e^{-2\mu_j r/a_H}\,dr}} \qquad (26)$$

$$\mu_j=\frac{Z_{eff}}{m_j} \qquad (27)$$

In the above Supplementary Equations, δ is a correction factor (δ=0 for n=1,2,3; δ=0.3 for n=4; and δ=1 for n=5), a_H is the Bohr radius (0.529 Å), and Z_{eff} is the effective nuclear charge. Given that the relevant overlap for electron transfer involves an electron rich iodine and an electron poor chalcogen, the best approximation for $S°_{DA}$ is the overlap between an isolated iodide anion and isolated chalcogen cations. Using Z_{eff} determined according to Slater's rules,[70] the calculated μ values for these species were 2.450 for O^+, 1.933 for S^+, 1.973 for Se^+, and 1.813 for I^-. Based on the van der Waals radii of the chalcogens, and the ionic radius of iodide, R was determined to be 3.68, 3.96, and 4.06 for $O^+\cdots I^-$, $S^+\cdots I^-$, and $Se^+\cdots I^-$ interactions, respectively. Using these values and evaluating the integrals in Supplementary Equations 22 and 26 using the Mathematica software suite,[71] values for $S°_{DA}$ were calculated and are reported in Supplementary Table 3.

Supplementary Table 1 | Electron density difference maps of major optical transitions. Visualizations of each of the TD-DFT calculated optical transitions indicated in Supplementary Figure 12 as electron density difference maps (EDDMs). The purple regions represent a negative change in electron density, while the green regions represent a positive change in electron density. All EDDMs were plotted at an isodensity of 0.0025. The inset numbers indicate the absolute transition number which are detailed in the Supplementary Data 2 file associated with this manuscript.

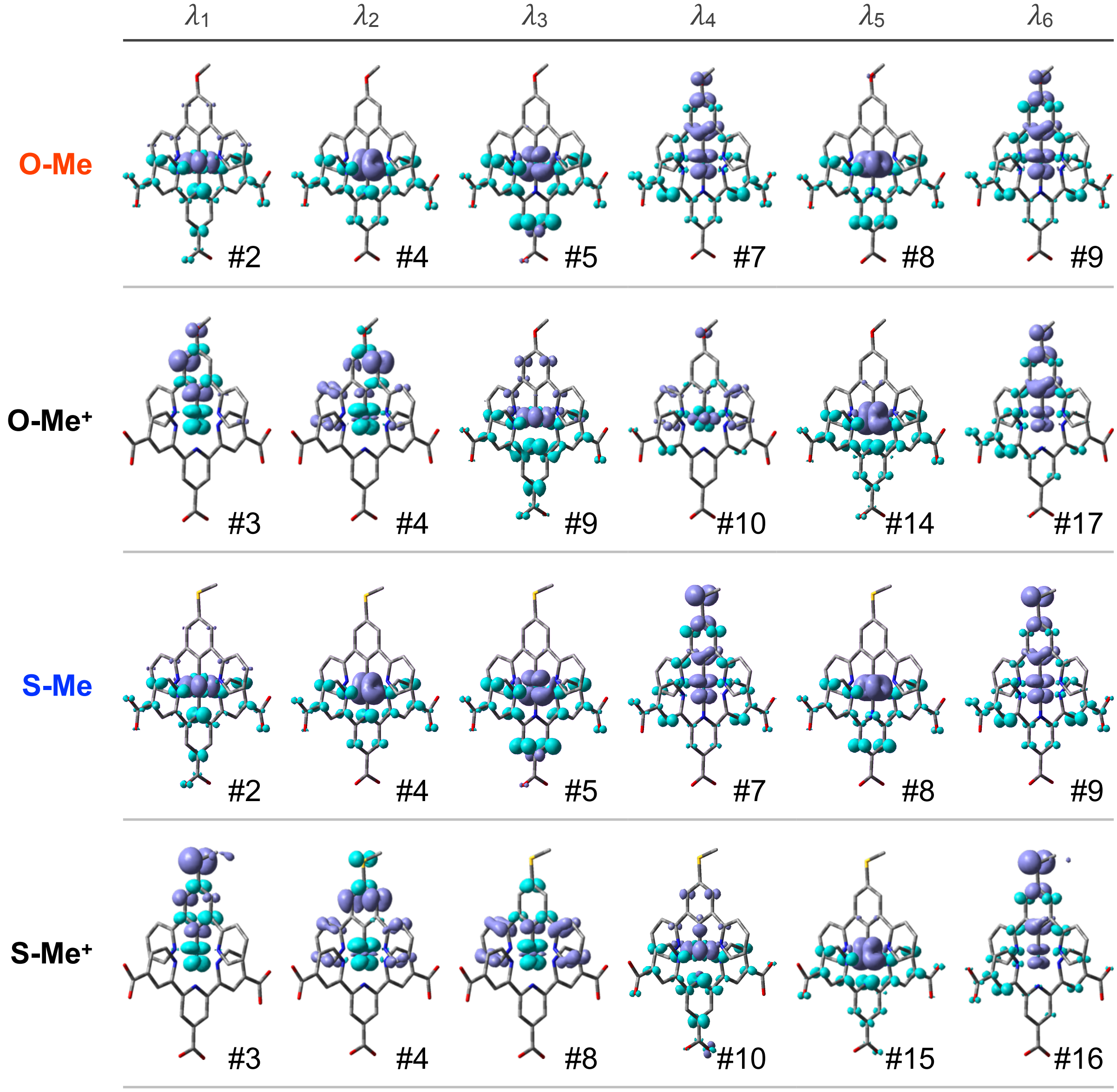

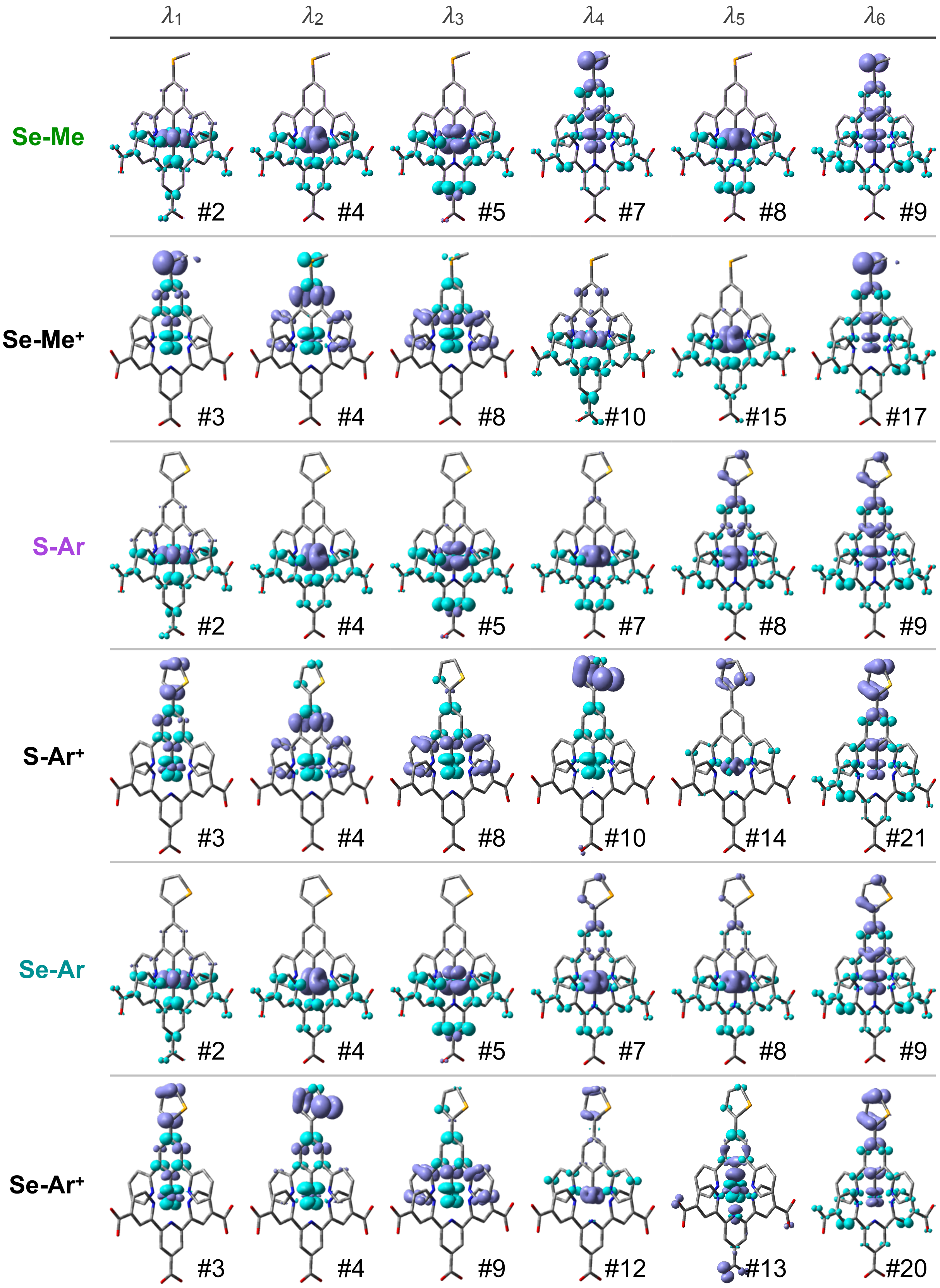
λ1
λ2
λ3
λ4
λ5
λ6
Se-Me
#2
#4
#5
#7
#8
#9
Se-Me+
#3
#4
#8
#10
#15
#17
S-Ar
#2
#4
#5
#7
#8
#9
S-Ar+
#3
#4
#8
#10
#14
#21
Se-Ar
#2
#4
#5
#7
#8
#9
Se-Ar+
#3
#4
#9
#12
#13
#20

Supplementary Table 2 | Selected molecular orbital plots. Visualizations of selected DFT-calculated molecular orbitals (MOs), plotted at an iso value of 0.05. The reduced ruthenium complexes were modeled using a restricted functional, and therefore the MOs represent 2-electron orbitals. The oxidized complexes were modeled using an unrestricted functional, and therefore the MOs represent one-electron orbitals, split between alpha and beta spins, as indicated.

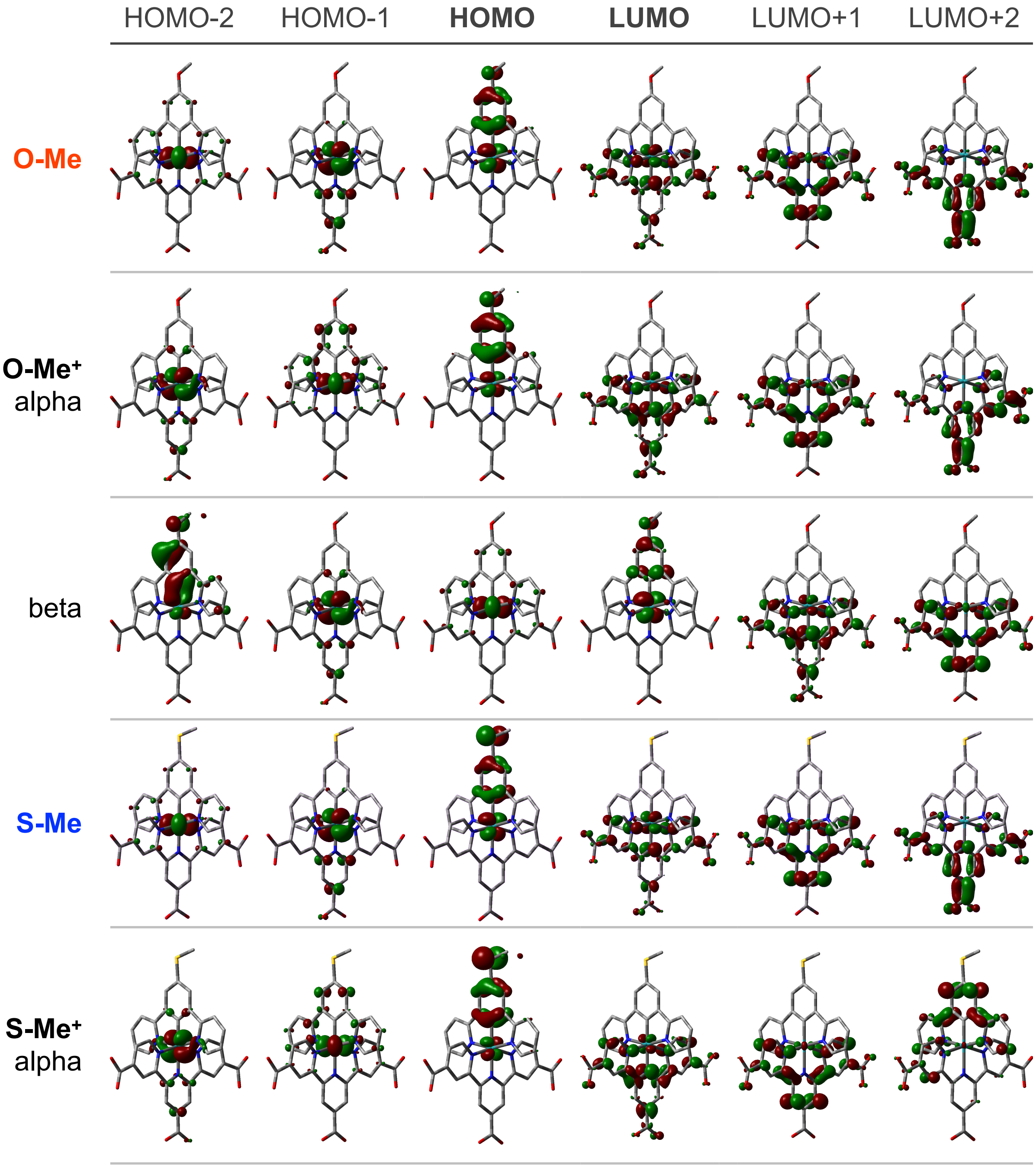

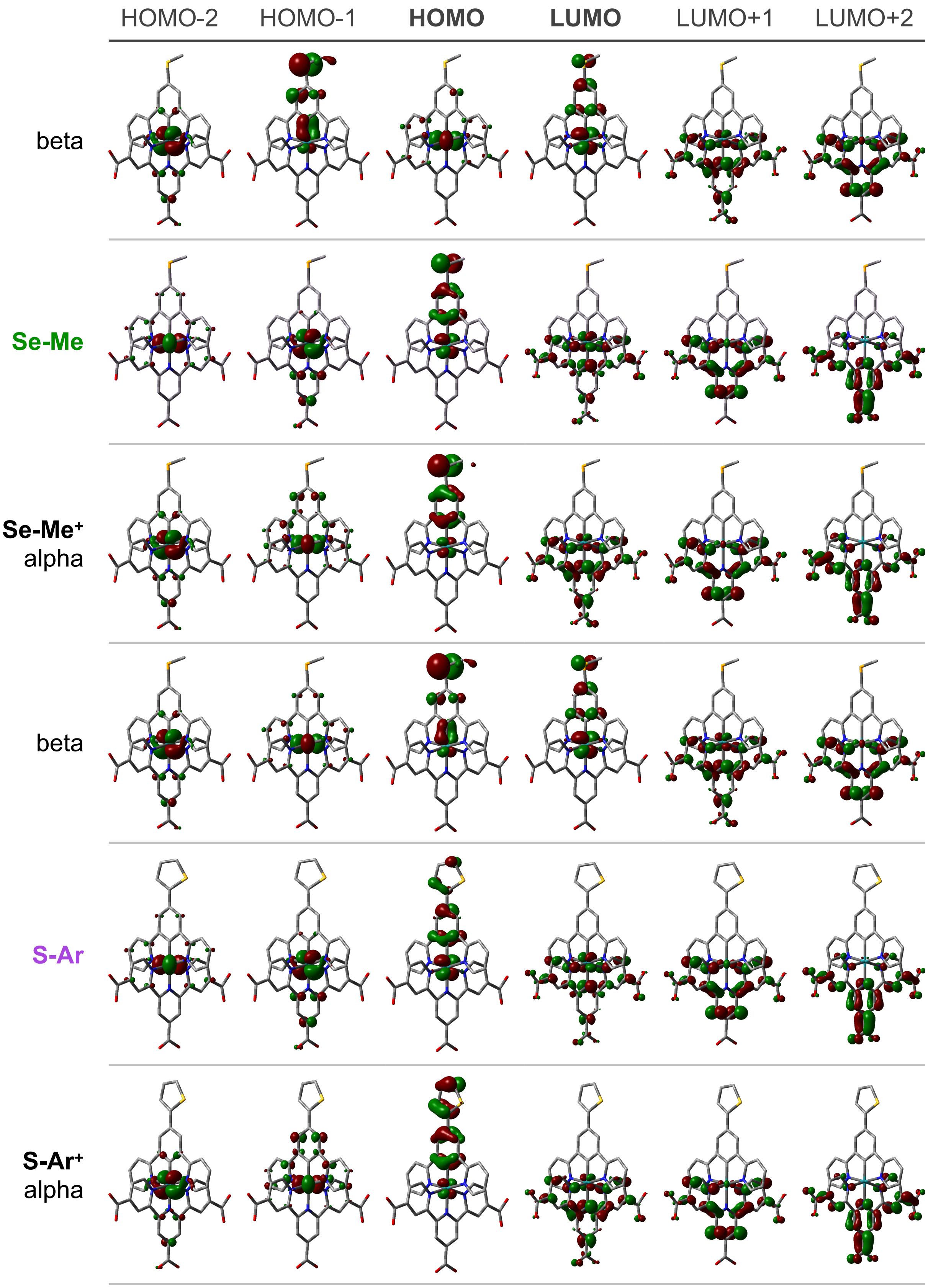
HOMO-2
HOMO-1
HOMO
LUMO
LUMO+1
LUMO+2
beta
Se-Me
Se-Me+
alpha
beta
S-Ar
S-Ar+
alpha

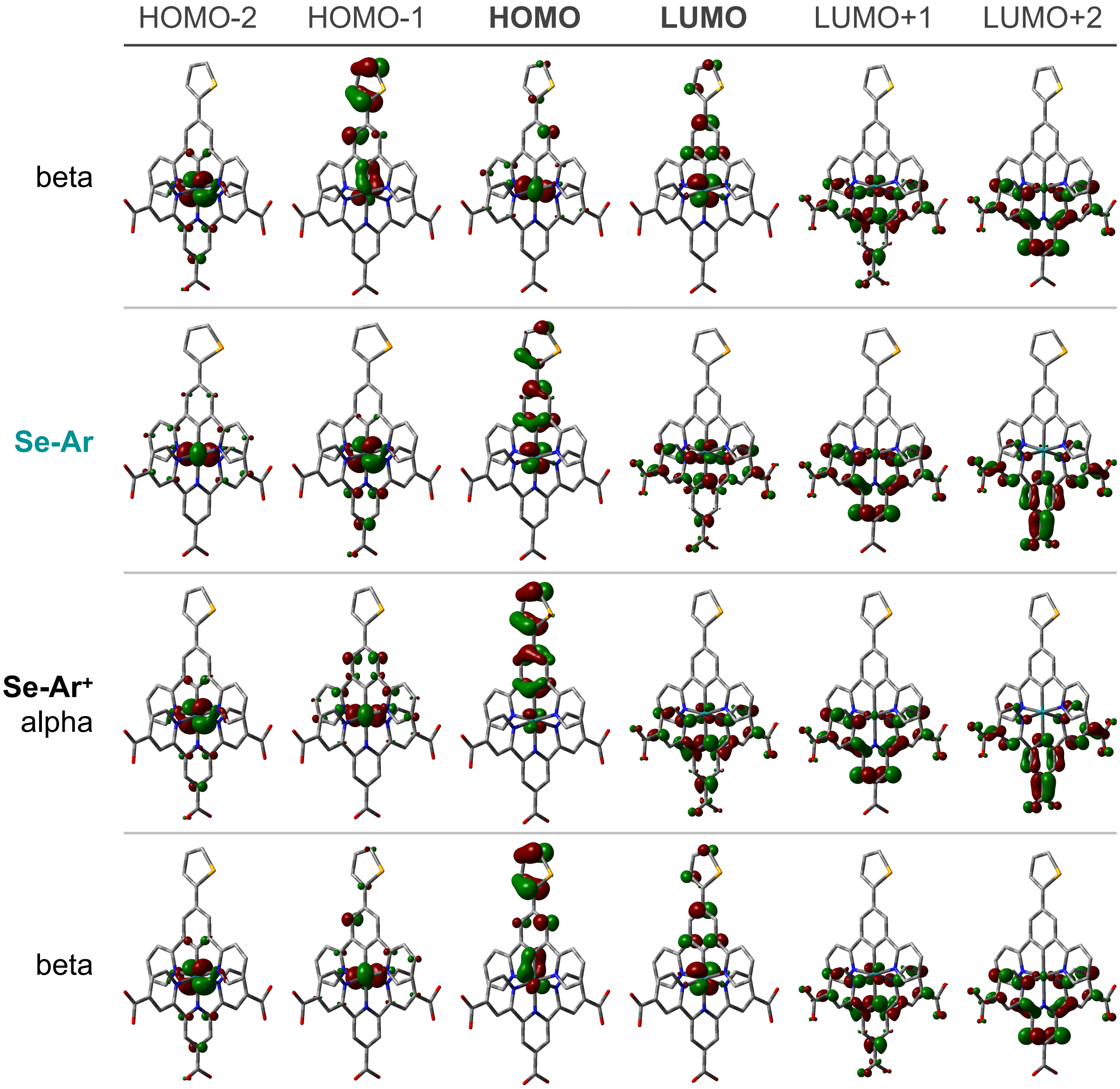

HOMO-2
HOMO-1
HOMO
LUMO
LUMO+1
LUMO+2
beta
Se-Ar
Se-Ar+
alpha
beta

Supplementary Table 3 | Measured and calculated values relevant to the analysis of k_{IET}.

	ΔE_{int}	K_1	K_A	λ	$\Delta G°_{ET}$	$\lvert S°_{DA}\rvert$
	kcal mol^{-1}	M^{-1}	x10^{-5} M^{-1}	eV	eV	
**O-Me**	-2.86	125	3.50	1.36	0.06	0.01237
**S-Me**	-3.33	277	3.58	1.32	-0.01	0.03324
**Se-Me**	-3.24	238	3.57	1.32	-0.03	0.04066
**S-Ar**	-3.59	430	3.60	1.30	-0.06	0.03324
**Se-Ar**	-3.43	328	3.59	1.30	-0.05	0.04066

Supplementary Table 4 | Values of $c_{A,X}$ and λ calculated using different DFT functionals.

	B3LYP		M06		mPW1PW91		BP86	
	$c_{A,X}$	λ	$c_{A,X}$	λ	$c_{A,X}$	λ	$c_{A,X}$	λ
		eV		eV		eV		eV
**O-Me**	0.054	1.35	0.051	1.35	0.050	1.35	0.086	1.32
**S-Me**	0.101	1.31	0.102	1.32	0.094	1.32	0.171	1.29
**Se-Me**	0.129	1.31	0.150	1.32	0.117	1.32	0.236	1.31
**S-Ar**	0.016	1.30	0.016	1.30	0.015	1.30	0.024	1.28
**Se-Ar**	0.019	1.30	0.020	1.30	0.018	1.30	0.031	1.28

Supplementary Note 1: NMR Spectra of Compounds

2-selenylboronic acid 300 MHz ^1H NMR in $(CD_3)_2SO$

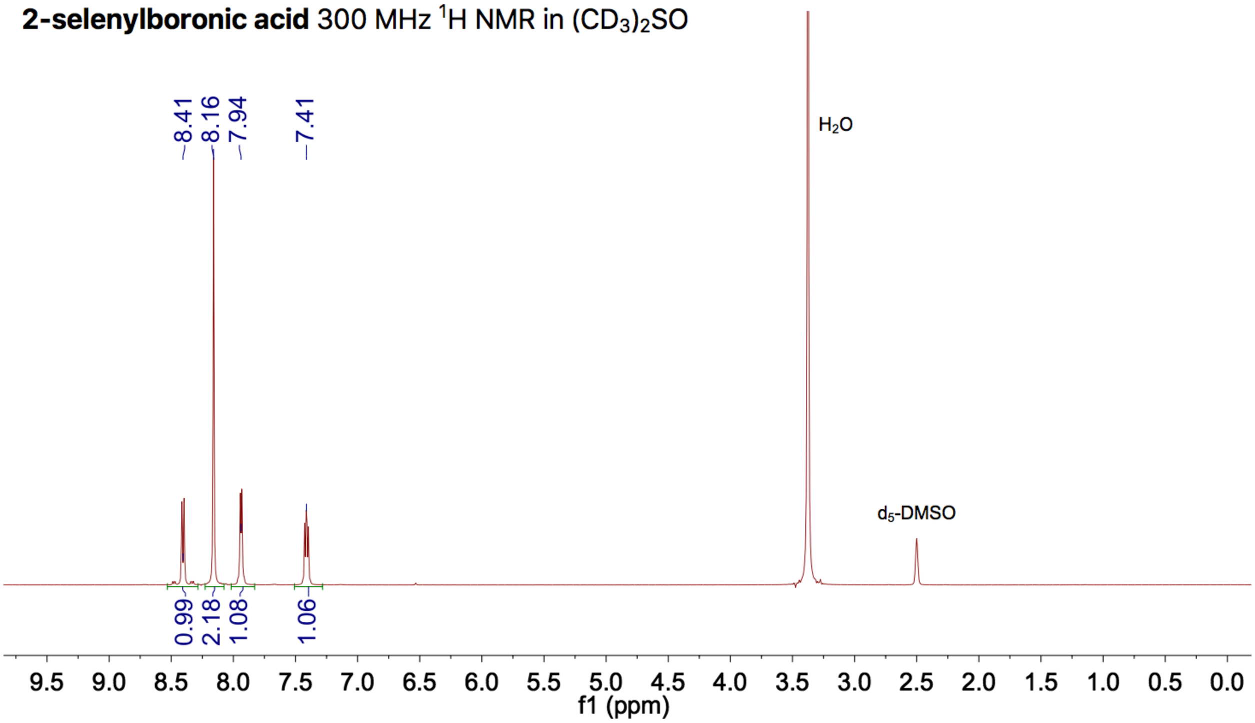

P-Se-Ar 400 MHz ^1H NMR in $CDCl_3$

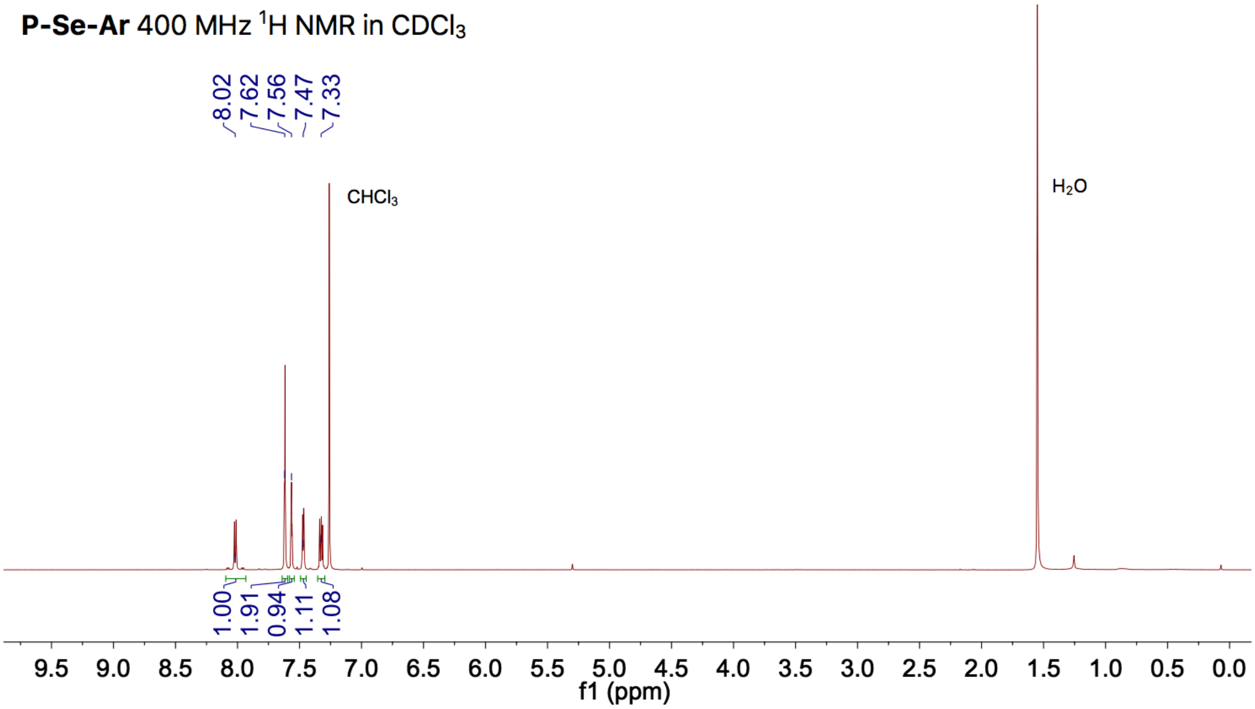

P-Se-Ar 100 MHz ^{13}C NMR in $CDCl_3$

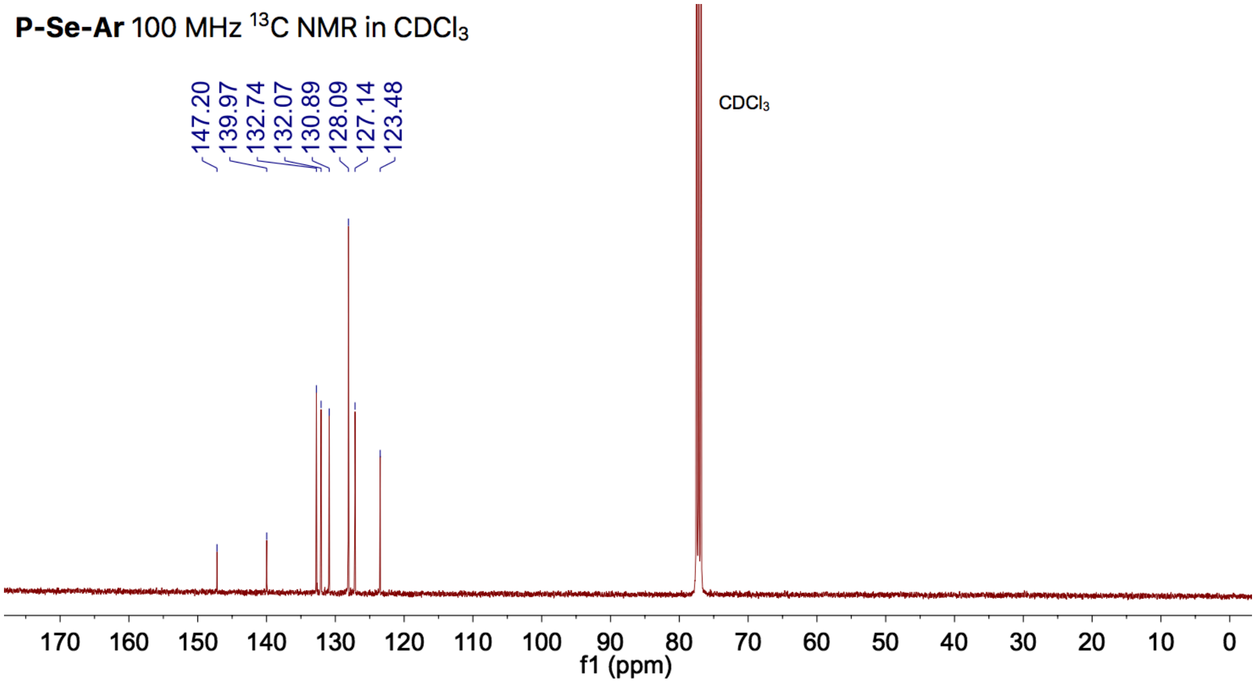

L-S-Me 400 MHz ^{1}H NMR in CD_2Cl_2

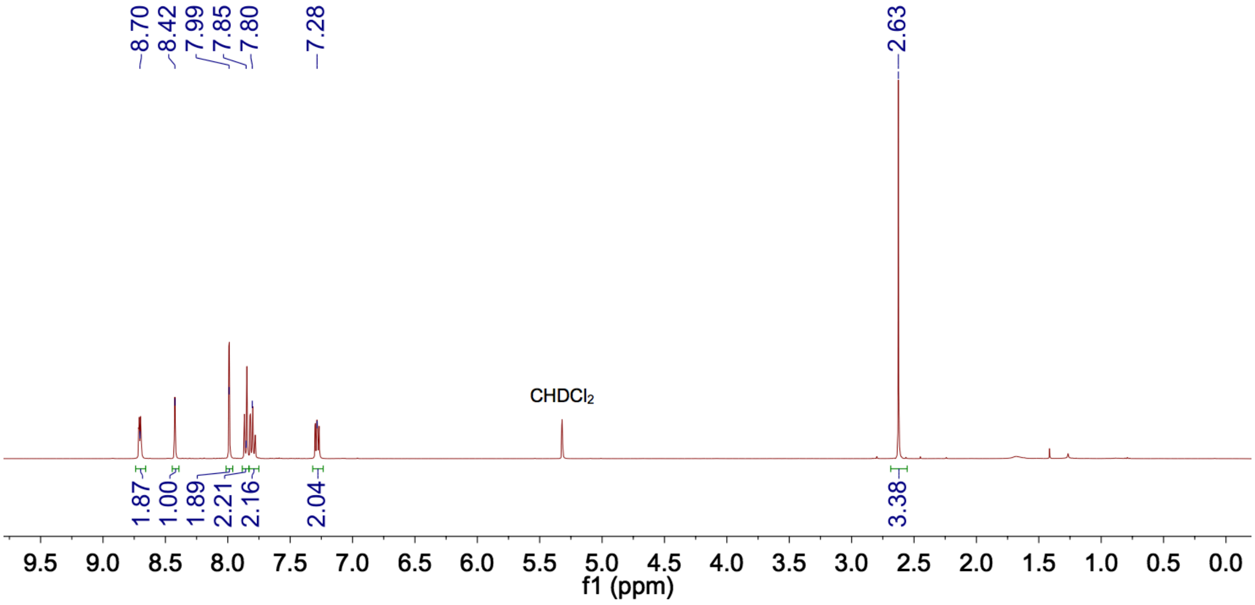

L-S-Me 75 MHz ^{13}C NMR in CD_2Cl_2

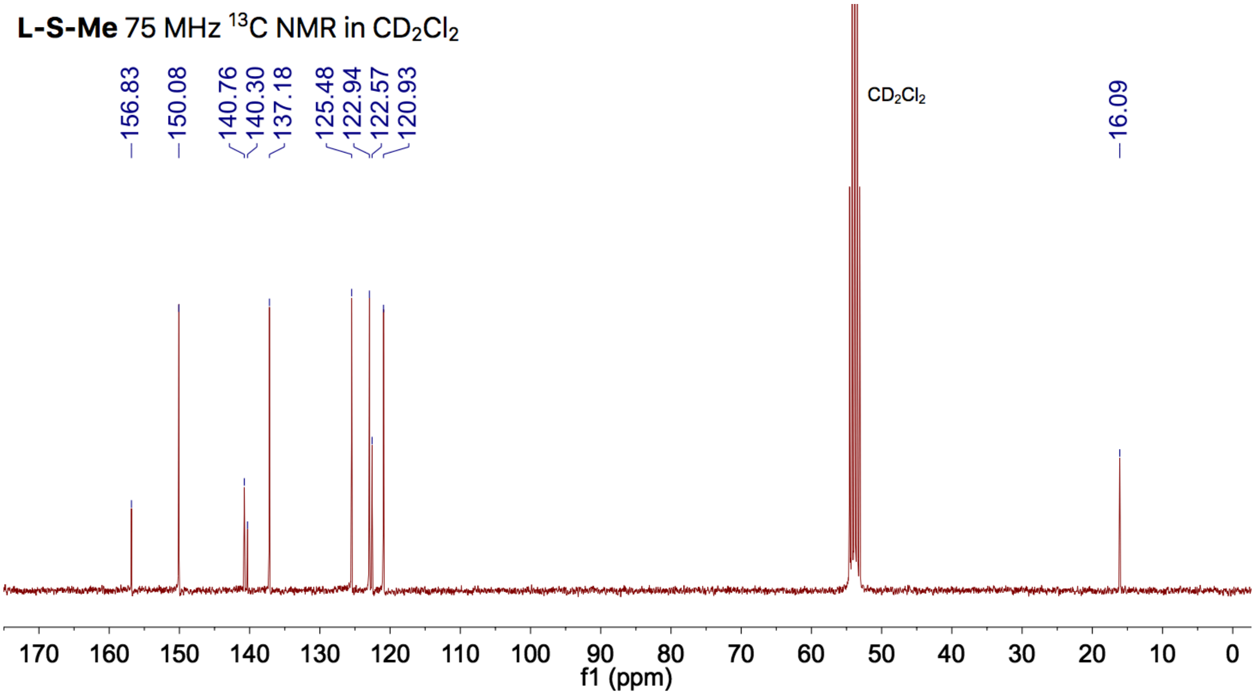

L-Se-Me 400 MHz ^1H NMR in CD_2Cl_2

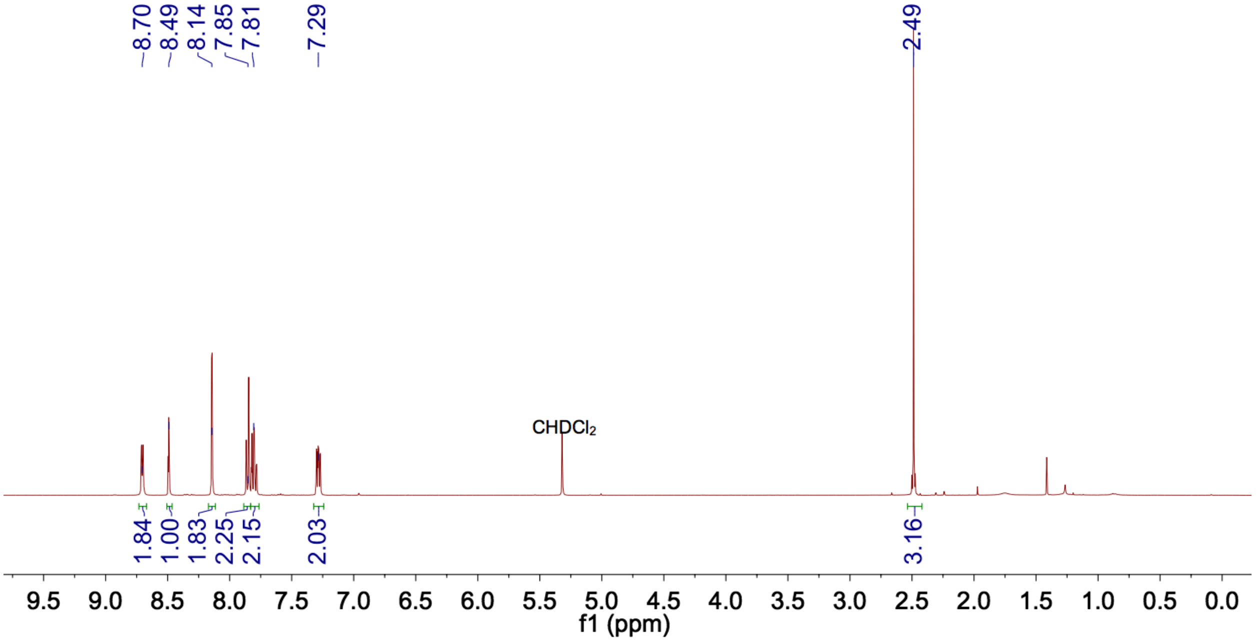

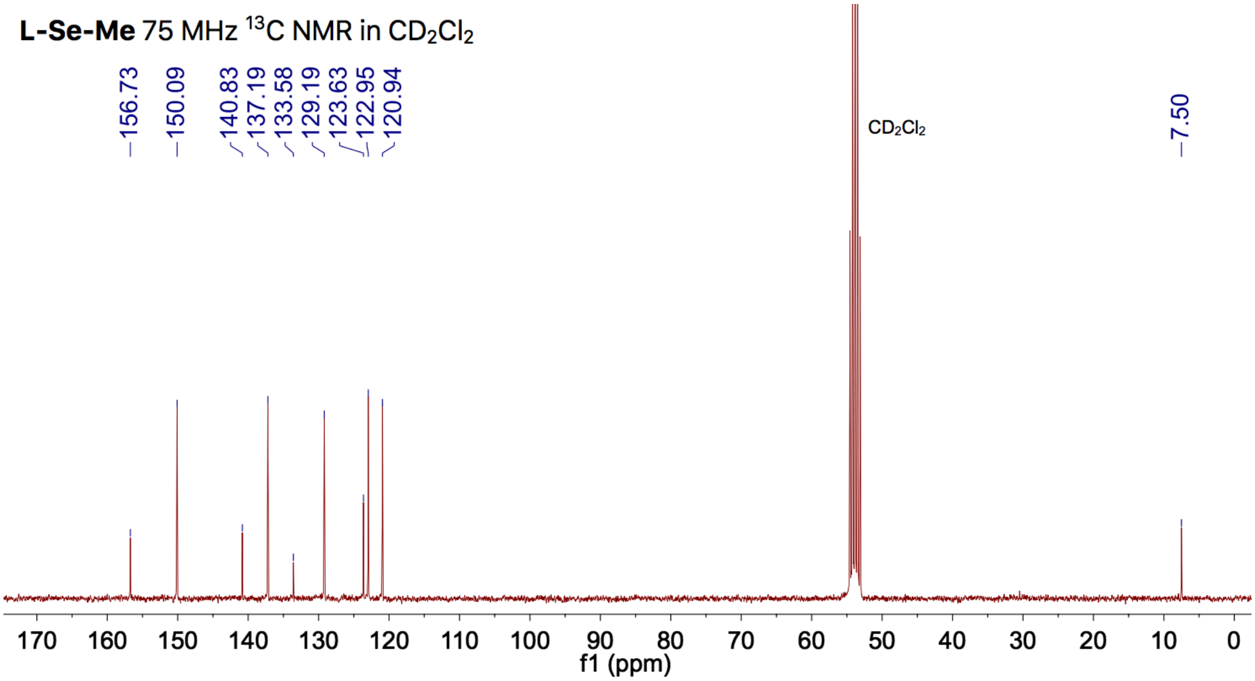
L-Se-Me 75 MHz 13C NMR in CD2Cl2
156.73
150.09
140.83
137.19
133.58
129.19
123.63
122.95
120.94
CD2Cl2
7.50
170 160 150 140 130 120 110 100 90 80 70 60 50 40 30 20 10 0
f1 (ppm)

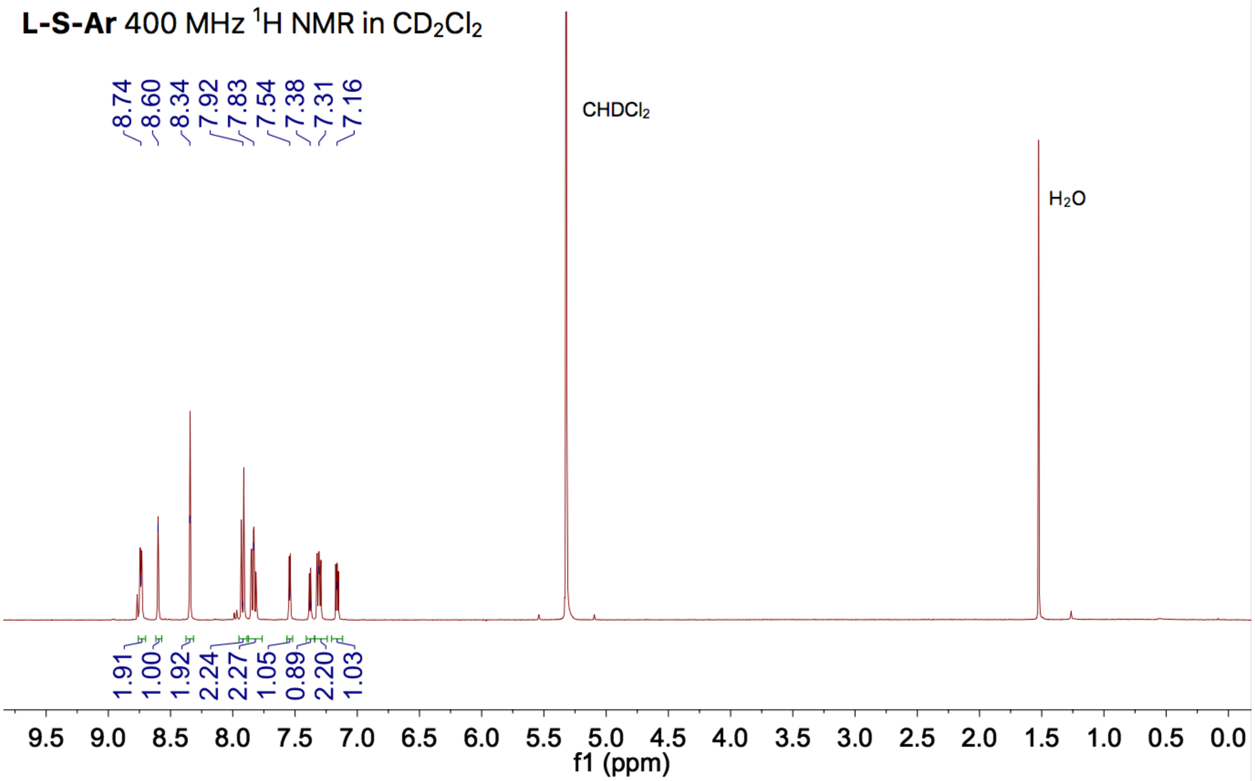
L-S-Ar 400 MHz 1H NMR in CD2Cl2
8.74
8.60
8.34
7.92
7.83
7.54
7.38
7.31
7.16
CHDCl2
H2O
1.91
1.00
1.92
2.24
2.27
1.05
0.89
2.20
1.03
9.5 9.0 8.5 8.0 7.5 7.0 6.5 6.0 5.5 5.0 4.5 4.0 3.5 3.0 2.5 2.0 1.5 1.0 0.5 0.0
f1 (ppm)

L-Se-Ar 400 MHz ^{1}H NMR in CD_2Cl_2

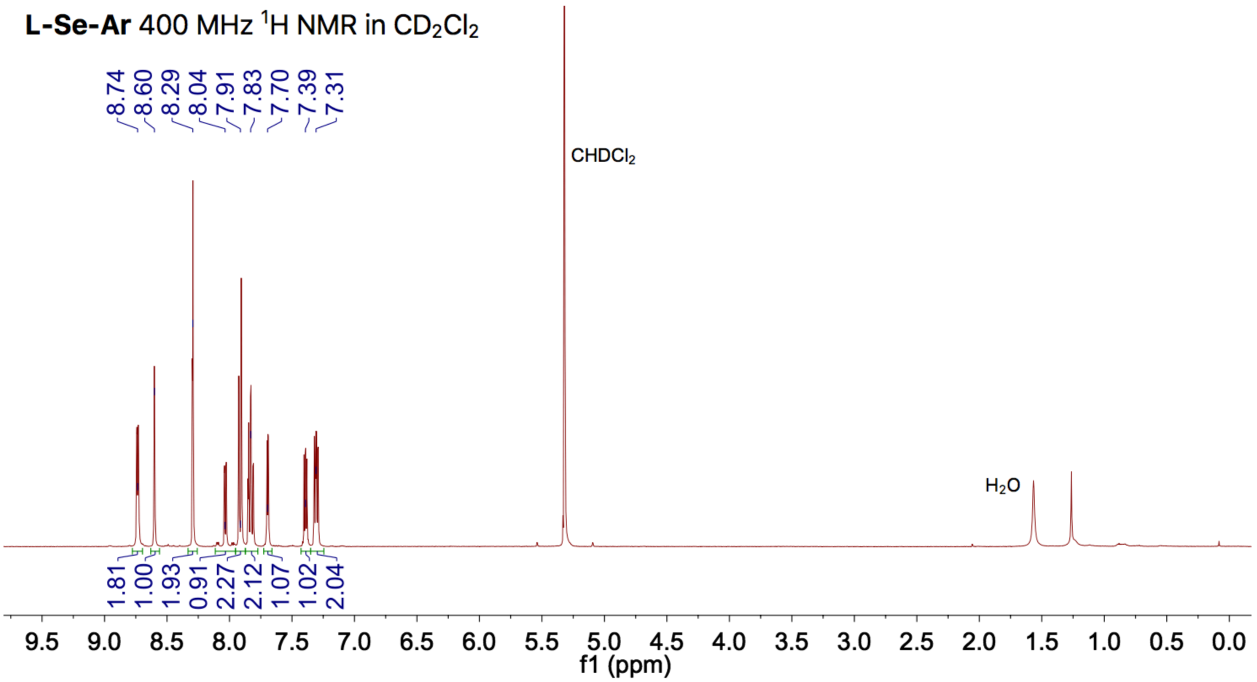

L-Se-Ar 75 MHz ^{13}C NMR in CD_2Cl_2

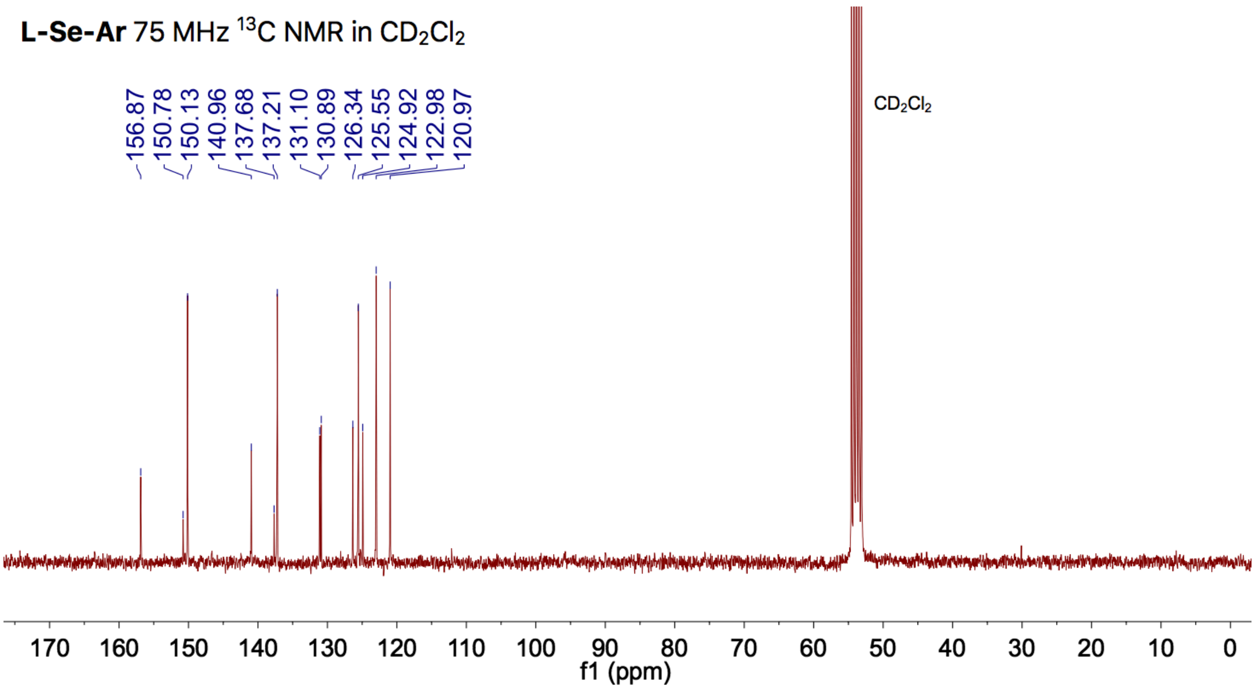

O-Me$_{Me}$ 400 MHz ^1H NMR in CD_2Cl_2

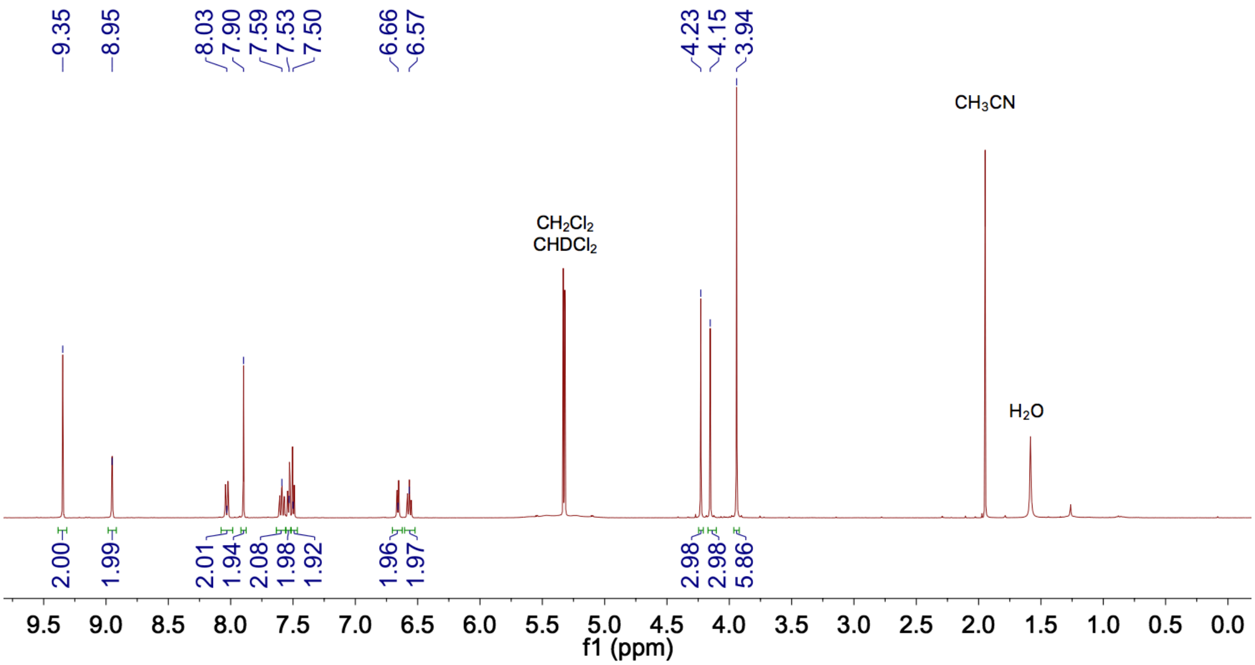

O-Me$_{Me}$ 100 MHz ^{13}C NMR in CD_2Cl_2

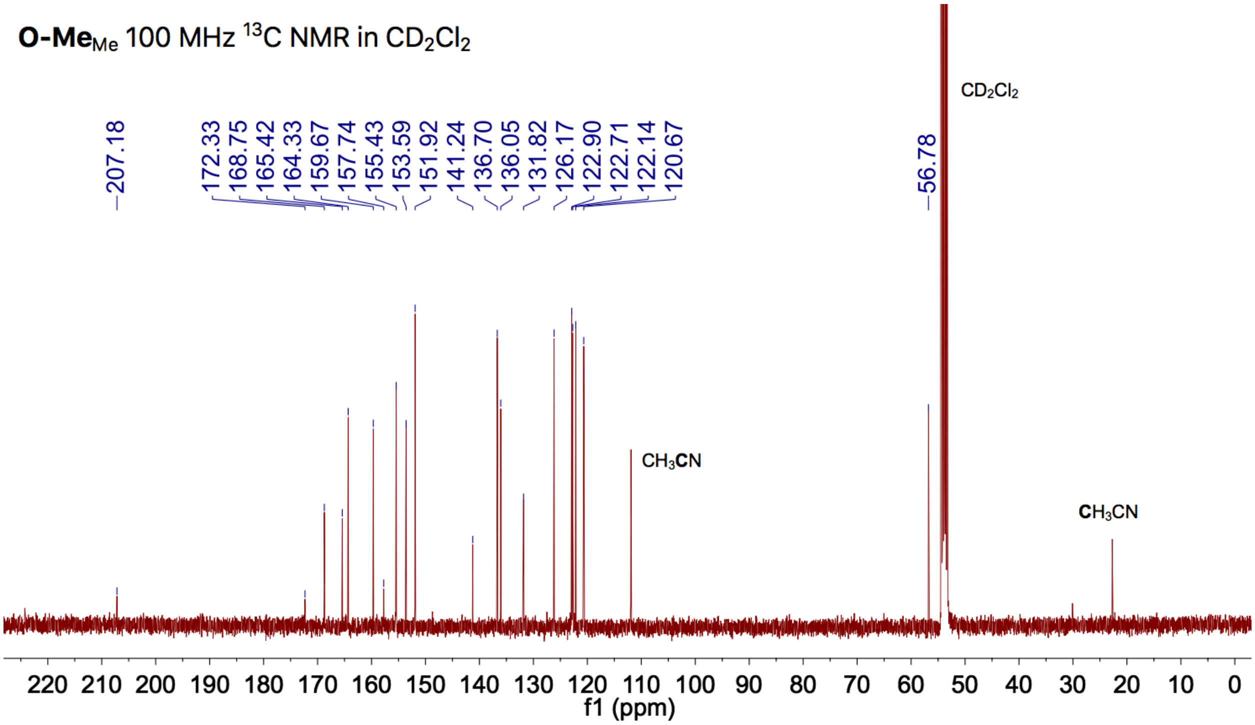

S-Me$_{Me}$ 400 MHz ^1H NMR in CD_2Cl_2

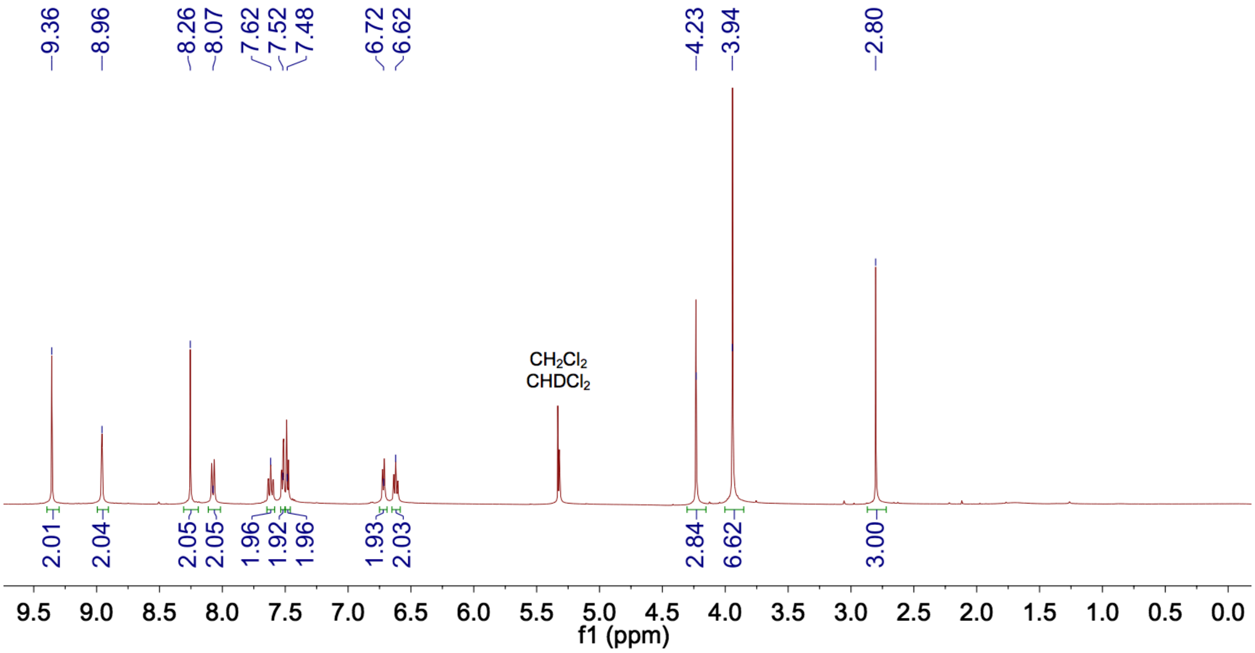

S-Me$_{Me}$ 100 MHz ^{13}C NMR in CD_2Cl_2

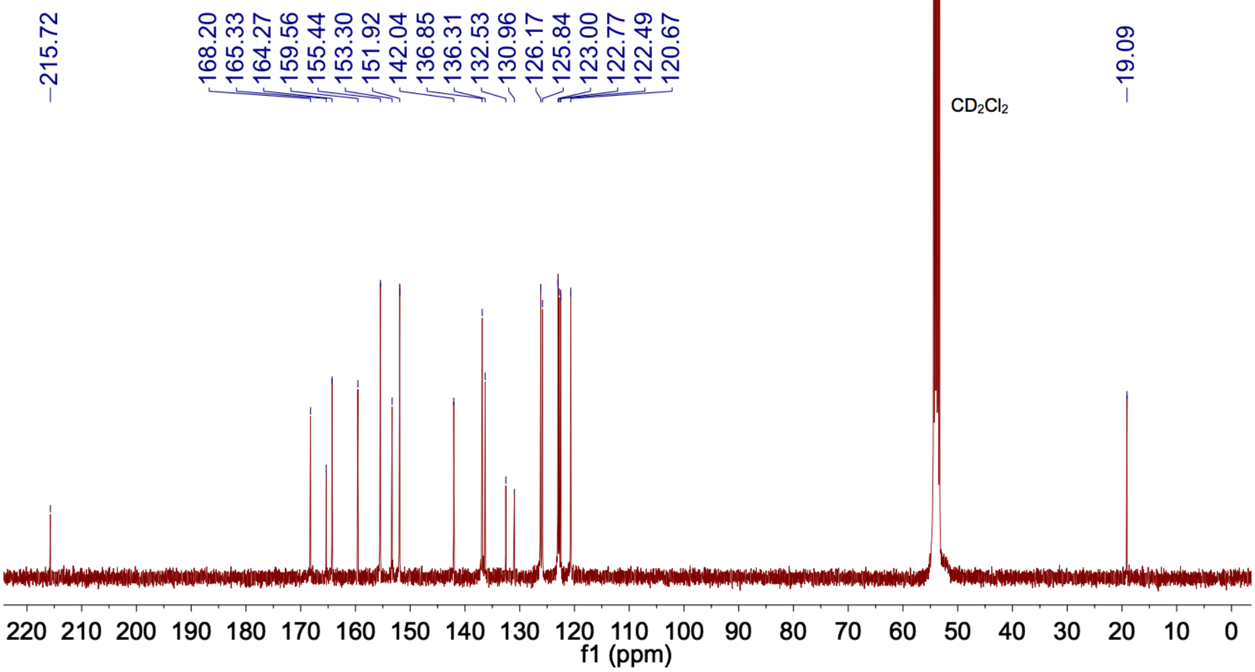

Se-Me$_{Me}$ 400 MHz ^{1}H NMR in CD_2Cl_2

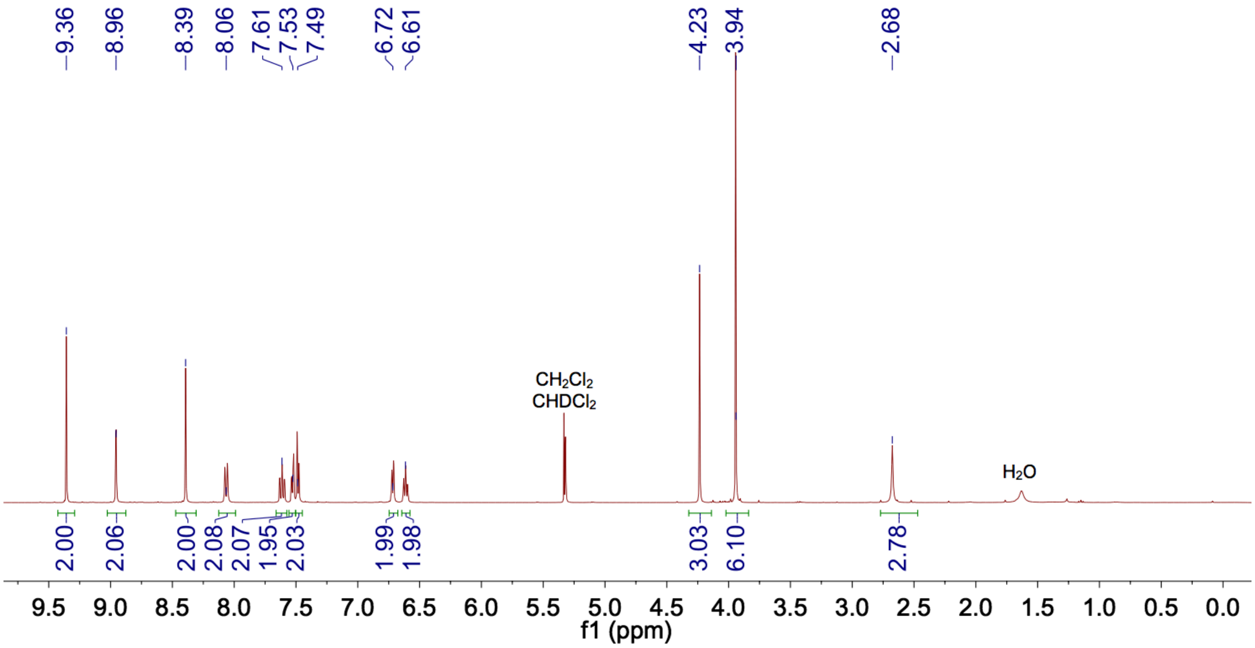

Se-Me$_{Me}$ 100 MHz ^{13}C NMR in CD_2Cl_2

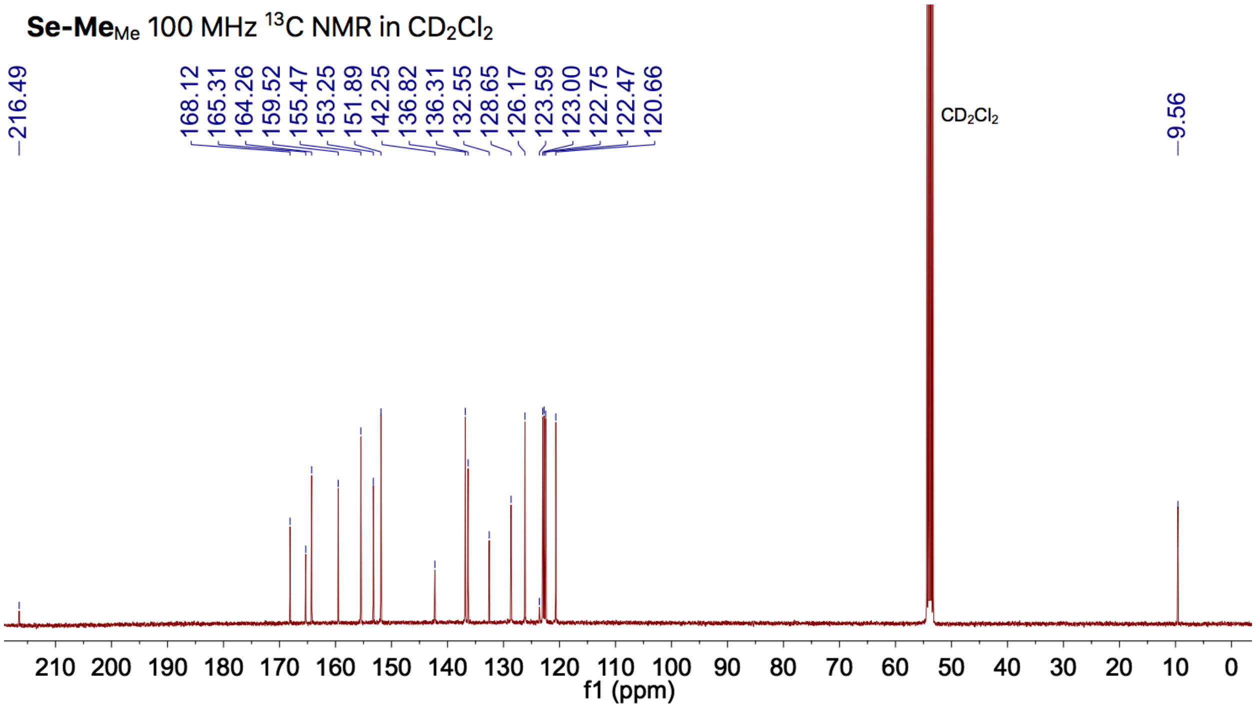

S-Ar$_{Me}$ 400 MHz ^1H NMR in CD_2Cl_2

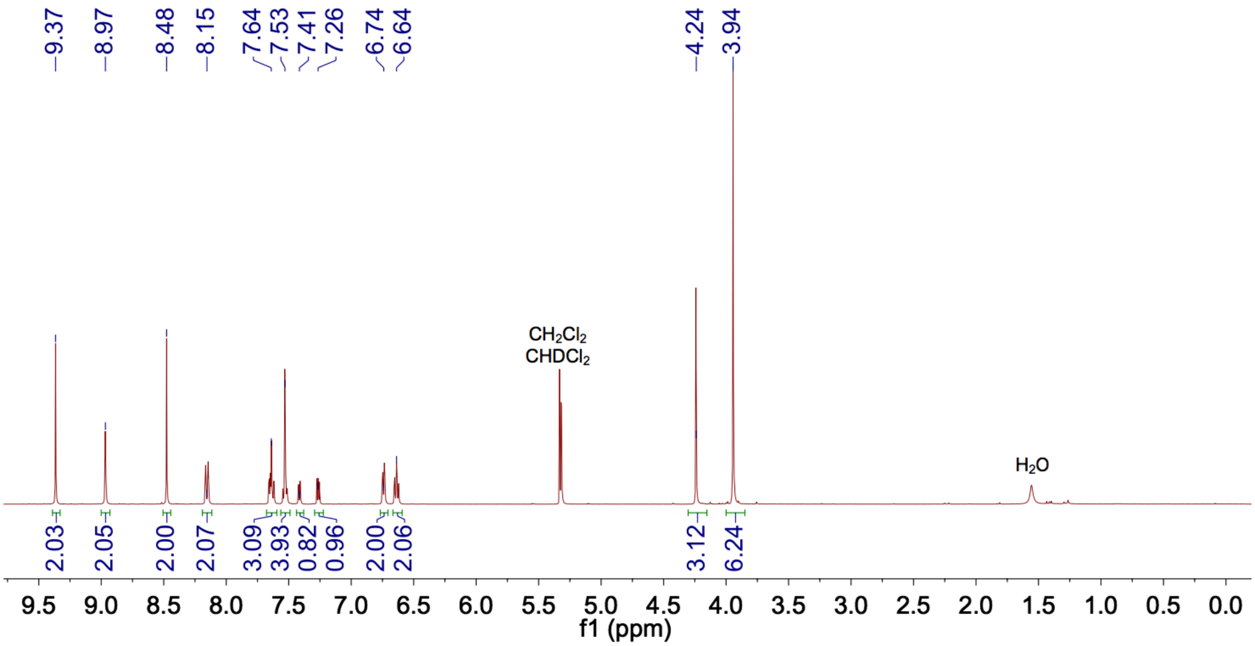

S-Ar$_{Me}$ 100 MHz ^{13}C NMR in CD_2Cl_2

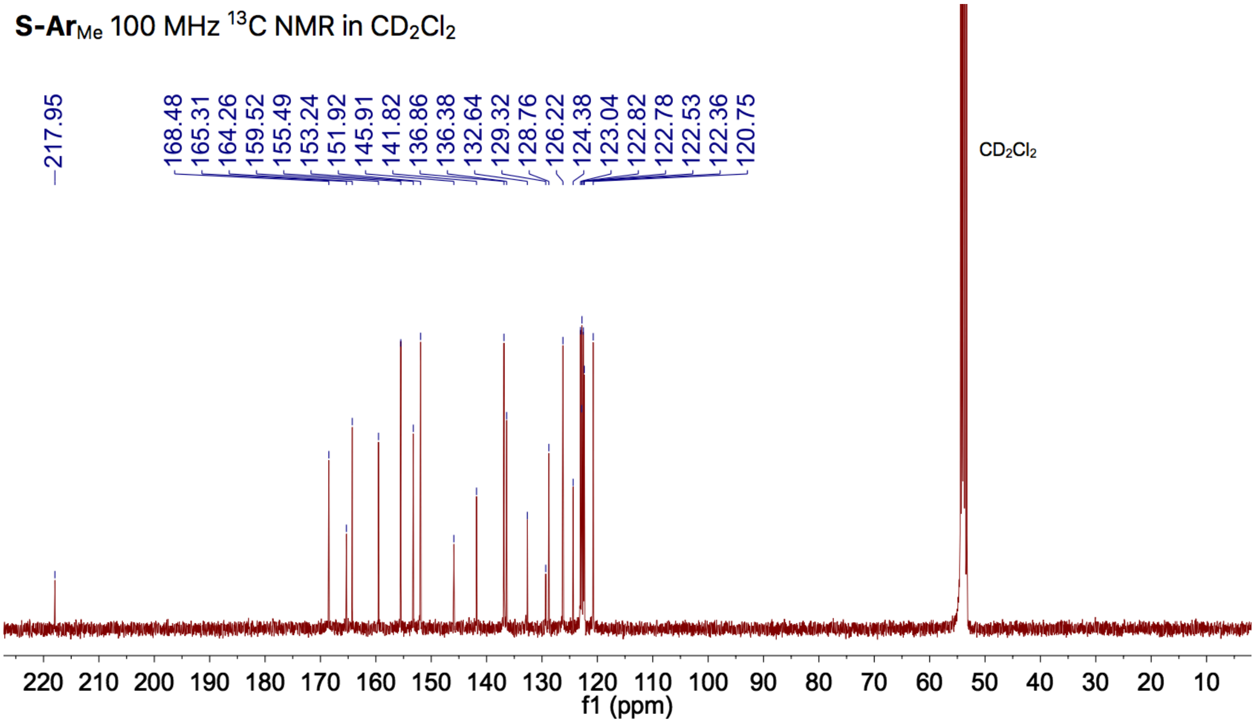

Se-Ar$_{Me}$ 400 MHz ^{1}H NMR in CD_2Cl_2

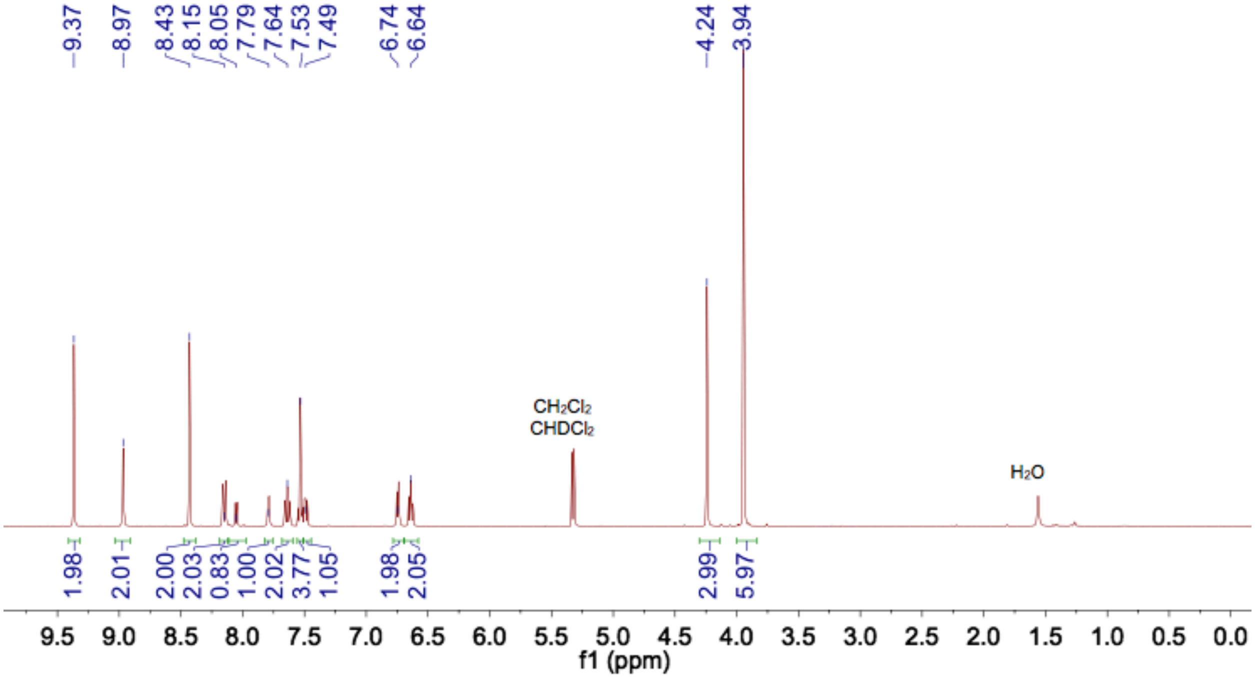

Se-Ar$_{Me}$ 100 MHz ^{13}C NMR in CD_2Cl_2

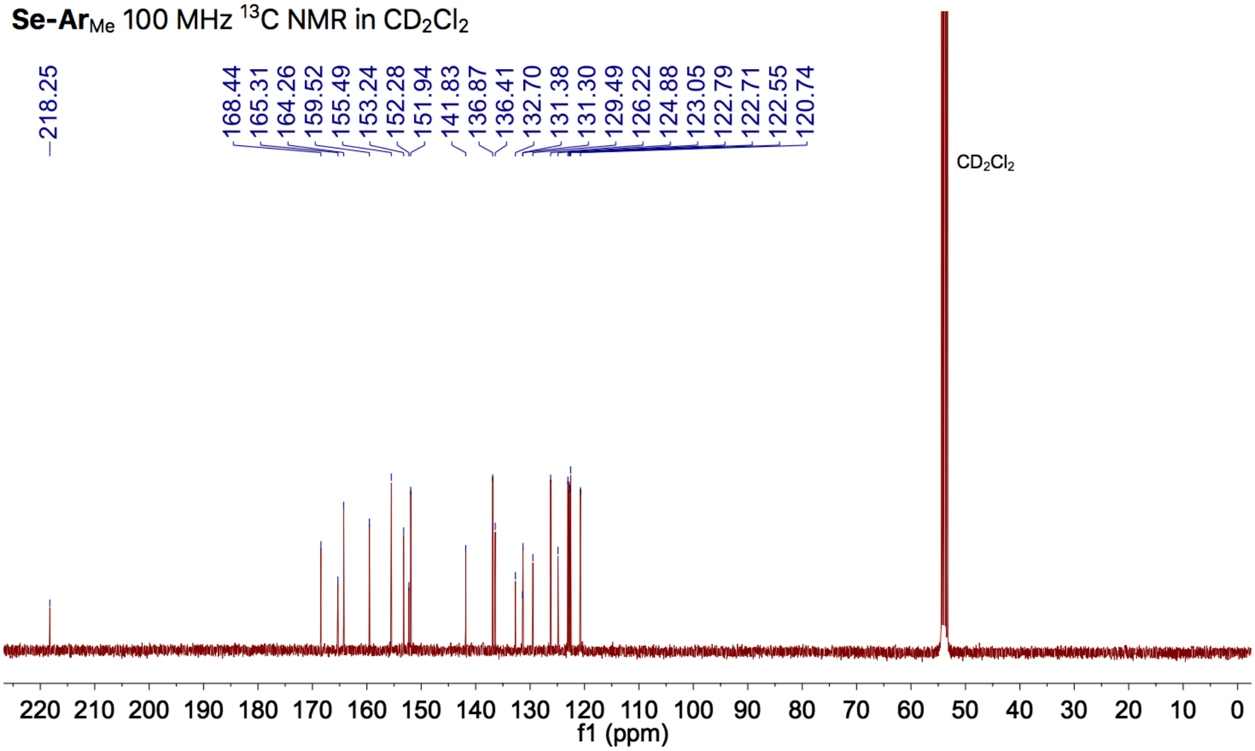

O-Me$_H$ 400 MHz ^{1}H NMR in 0.1M $NaOCD_3/CD_3OD$

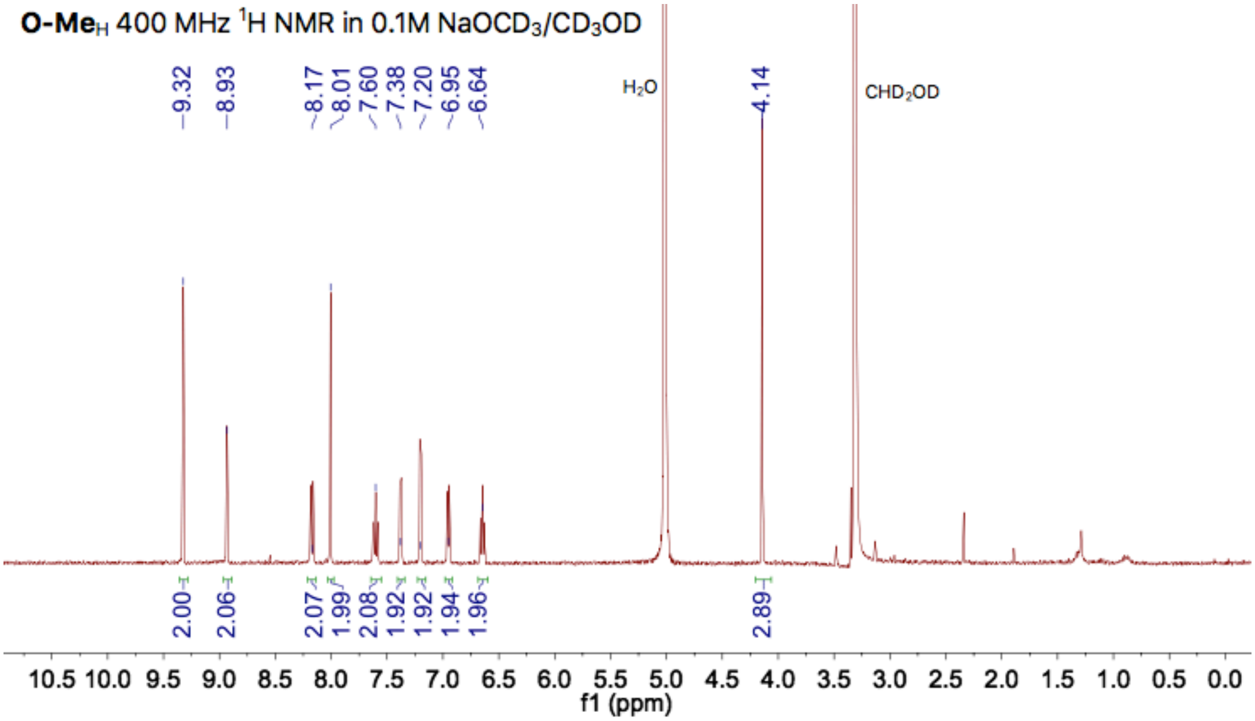

S-Me$_H$ 400 MHz ^{1}H NMR in 0.1M $NaOCD_3/CD_3OD$

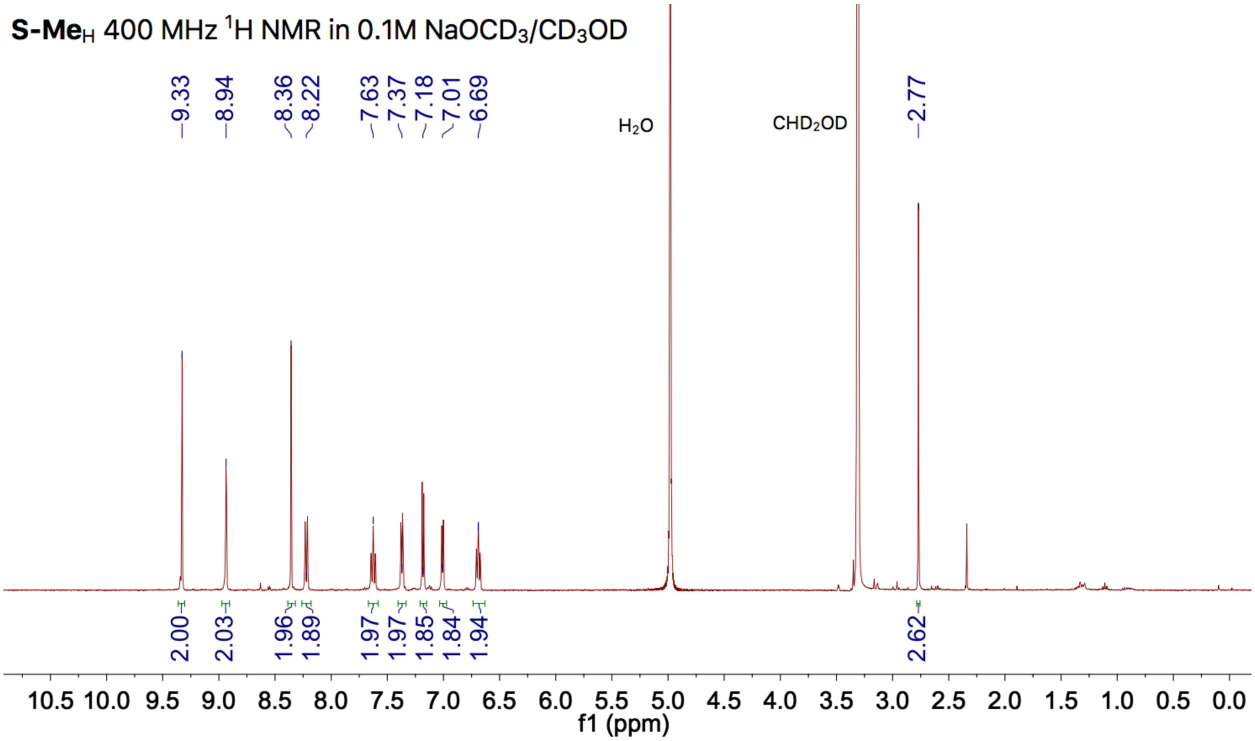

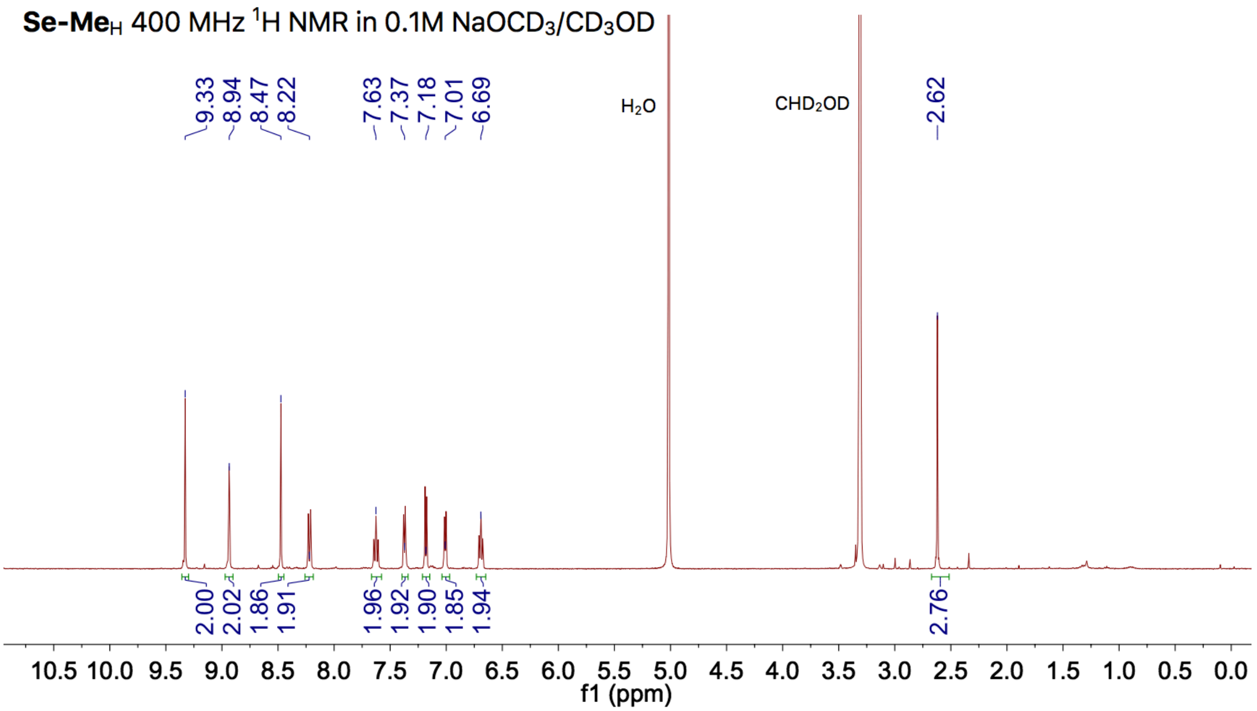
Se-Me_H 400 MHz 1H NMR in 0.1M NaOCD3/CD3OD
9.33
8.94
8.47
8.22
7.63
7.37
7.18
7.01
6.69
H2O
CHD2OD
2.62
2.00
2.02
1.86
1.91
1.96
1.92
1.90
1.85
1.94
2.76
10.5 10.0 9.5 9.0 8.5 8.0 7.5 7.0 6.5 6.0 5.5 5.0 4.5 4.0 3.5 3.0 2.5 2.0 1.5 1.0 0.5 0.0
f1 (ppm)

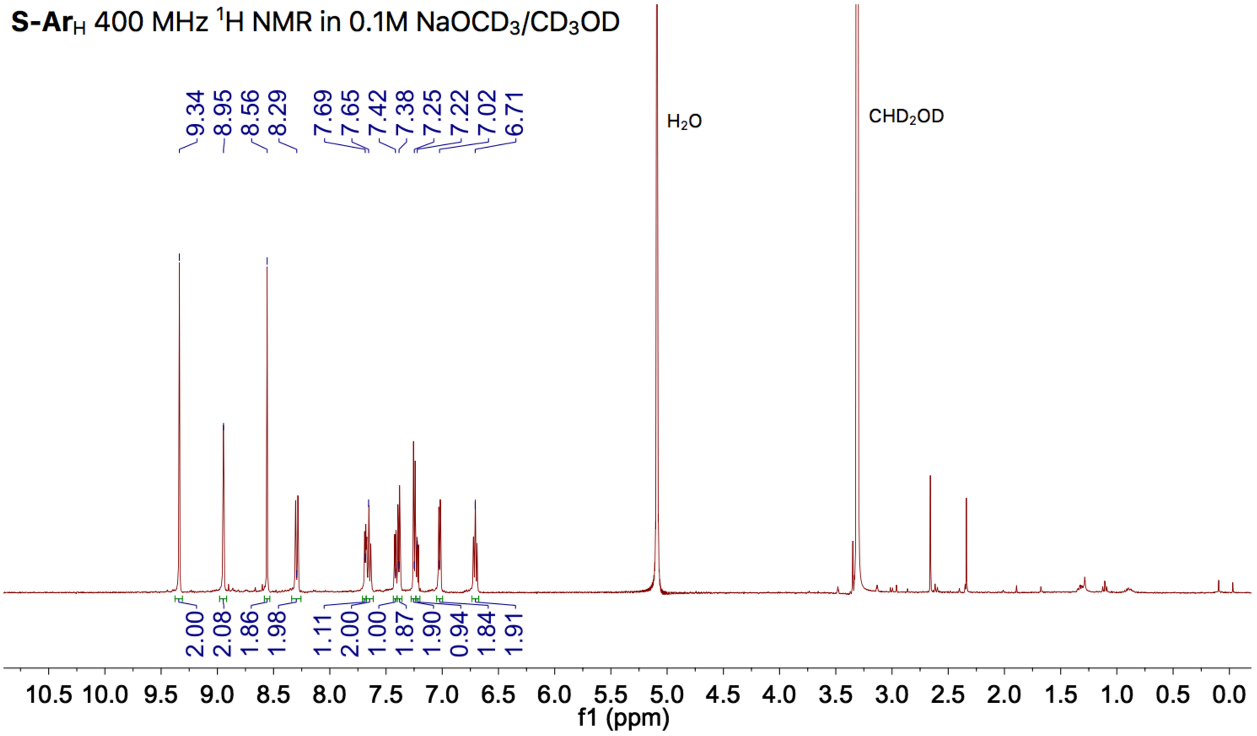
S-Ar_H 400 MHz 1H NMR in 0.1M NaOCD3/CD3OD
9.34
8.95
8.56
8.29
7.69
7.65
7.42
7.38
7.25
7.22
7.02
6.71
H2O
CHD2OD
2.00
2.08
1.86
1.98
1.11
2.00
1.00
1.87
1.90
0.94
1.84
1.91
10.5 10.0 9.5 9.0 8.5 8.0 7.5 7.0 6.5 6.0 5.5 5.0 4.5 4.0 3.5 3.0 2.5 2.0 1.5 1.0 0.5 0.0
f1 (ppm)

Se-Ar$_H$ 400 MHz ^{1}H NMR in 0.1M $NaOCD_3/CD_3OD$

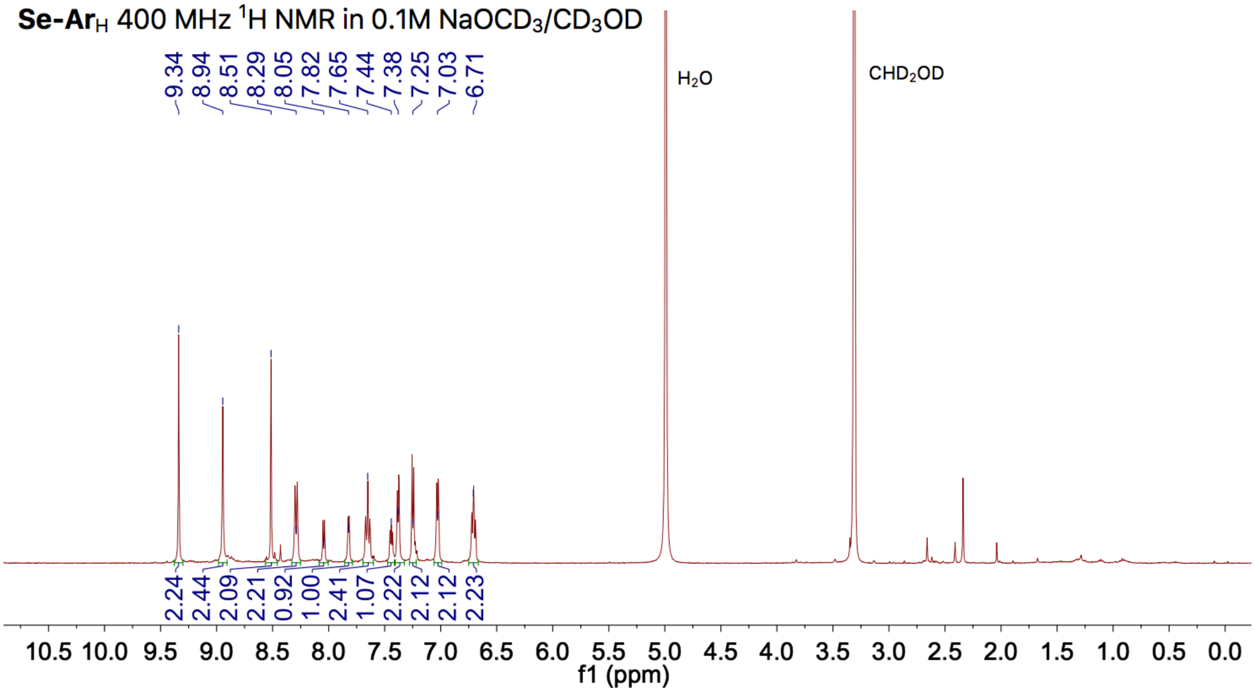

Supplementary References

1. Pavlishchuk, V. V. & Addison, A. W. Conversion constants for redox potentials measured versus different reference electrodes in acetonitrile solutions at 25°C. *Inorganica Chimica Acta* **298**, 97–102 (2000).
2. Fulmer, G. R. *et al*. NMR Chemical Shifts of Trace Impurities: Common Laboratory Solvents, Organics, and Gases in Deuterated Solvents Relevant to the Organometallic Chemist. *Organometallics* **29**, 2176–2179 (2010).
3. Collis, G. E., Burrell, A. K., Scott, S. M. & Officer, D. L. Toward Functionalized Conducting Polymers: Synthesis and Characterization of Novel β-(Styryl)terthiophenes. *J. Org. Chem.* **68,** 8974–8983 (2003).
4. Farley, S. J., Rochester, D. L., Thompson, A. L., Howard, J. A. K. & Williams, J. A. G. Controlling Emission Energy, Self-Quenching, and Excimer Formation in Highly Luminescent N^C^N-Coordinated Platinum(II) Complexes. *Inorg. Chem.* **44**, 9690–9703 (2005).
5. Hoang, T. N. Y. *et al.* A Polyaromatic Terdentate Binding Unit with Fused 5,6-Membered Chelates for Complexing s-, p-, d-, and f-Block Cations. *Inorg. Chem.* **52**, 5570–5580 (2013).
6. Sindkhedkar, M. D., Mulla, H. R., Wurth, M. A. & Cammers-Goodwin, A. Aromatic interactions in the synthesis and conformation of two collapsible tetracationic cyclophanes. *Tetrahedron* **57**, 2991–2996 (2001).
7. Wadman, S. H., Tooke, D. M., Spek, A. L., van Klink, G. P. M. & van Koten, G. Cyclo-ruthenated and -platinated complexes bearing phosphonate substituents. *Inorganica Chim. Acta* **363**, 1701–1706 (2010).
8. Wang, Z. *et al.* Facile Synthesis and Characterization of Phosphorescent Pt(N ∧C ∧N)X Complexes. *Inorg. Chem.* **49**, 11276–11286 (2010).
9. Husson, J., Dehaudt, J. & Guyard, L. Preparation of carboxylate derivatives of terpyridine via the furan pathway. *Nat. Protoc.* **9**, 21–26 (2013).
10. Dehaudt, J., Husson, J. & Guyard, L. A more efficient synthesis of 4,4′,4′′-tricarboxy-2,2′: 6′,2′′-terpyridine. *Green Chem.* **13**, 3337 (2011).
11. Nazeeruddin, M. K. *et al.* Engineering of Efficient Panchromatic Sensitizers for Nanocrystalline TiO-Based Solar Cells. *J. Am. Chem. Soc.* **123**, 1613–1624 (2001).

12. Farnum, B. H., Morseth, Z. A., Brennaman, M. K., Papanikolas, J. M. & Meyer, T. J. Application of Degenerately Doped Metal Oxides in the Study of Photoinduced Interfacial Electron Transfer. *J. Phys. Chem. B* **119**, 7698–7711 (2015).
13. Heimer, T. A., D'Arcangelis, S. T., Farzad, F., Stipkala, J. M. & Meyer, G. J. An Acetylacetonate-Based Semiconductor−Sensitizer Linkage. *Inorg. Chem.* **35**, 5319–5324 (1996).
14. Alibabaei, L., Sherman, B. D., Norris, M. R., Brennaman, M. K. & Meyer, T. J. Visible photoelectrochemical water splitting into H_2 and O_2 in a dye-sensitized photoelectrosynthesis cell. *Proc. Natl. Acad. Sci. U. S. A.* **112**, 5899–5902 (2015).
15. Gaussian 16, Revision A.03, Frisch, M. J. *et al.* Gaussian, Inc., Wallingford CT (2016).
16. Adamo, C. & Barone, V. Toward reliable density functional methods without adjustable parameters: The PBE0 model. *J. Chem. Phys.* **110**, 6158–6114 (1999).
17. Marenich, A. V., Cramer, C. J. & Truhlar, D. G. Universal solvation model based on solute electron density and on a continuum model of the solvent defined by the bulk dielectric constant and atomic surface tensions. *J. Phys. Chem. B* **113**, 6378–6396 (2009).
18. Dunning, T. H. Gaussian basis sets for use in correlated molecular calculations. I. The atoms boron through neon and hydrogen. *J. Chem. Phys.* **90**, 1007–1018 (1989).
19. Kendall, R. A., Dunning, T. H. & Harrison, R. J. Electron affinities of the first-row atoms revisited. Systematic basis sets and wave functions. *J. Chem. Phys.* **96**, 6796–6712 (1992).
20. Woon, D. E. & Dunning, T. H. Gaussian basis sets for use in correlated molecular calculations. III. The atoms aluminum through argon. *J. Chem. Phys.* **98**, 1358–1315 (1993).
21. Peterson, K. A., Woon, D. E. & Dunning, T. H. Benchmark calculations with correlated molecular wave functions. IV. The classical barrier height of the $H+H_2 \rightarrow H_2+H$ reaction. *J. Chem. Phys.* **100**, 7410–7417 (1994).
22. Davidson, E. R. Comment on 'Comment on Dunning's correlation-consistent basis sets'. *Chem. Phys. Lett.* **260**, 514–518 (1996).
23. Peterson, K. A., Figgen, D., Goll, E., Stoll, H. & Dolg, M. Systematically convergent basis sets with relativistic pseudopotentials. II. Small-core pseudopotentials and correlation consistent basis sets for the post-d group 16–18 elements. *J. Chem. Phys.* **119**, 11113–11112 (2003).
24. Peterson, K. A., Figgen, D., Dolg, M. & Stoll, H. Energy-consistent relativistic pseudopotentials and correlation consistent basis sets for the 4d elements Y–Pd. *J. Chem. Phys.* **126**, 124101–124113 (2007).
25. GaussView, Version 5, Dennington, R., Keith, T. A. & Millam, J. M. Semichem Inc., Shawnee Mission, KS (2016).

26. Bauernschmitt, R. & Ahlrichs, R. Treatment of electronic excitations within the adiabatic approximation of time dependent density functional theory. *Chem. Phys. Lett.* 256, 454–464 (1996).
27. Casida, M. E., Jamorski, C., Casida, K. C. & Salahub, D. R. Molecular excitation energies to high-lying bound states from time-dependent density-functional response theory: Characterization and correction of the time-dependent local density approximation ionization threshold. *J. Chem. Phys.* **108**, 4439–4449 (1998).
28. Stratmann, R. E., Scuseria, G. E. & Frisch, M. J. An efficient implementation of time-dependent density-functional theory for the calculation of excitation energies of large molecules. *J. Chem. Phys.* **109**, 8218–8218 (1998).
29. Van Caillie, C. & Amos, R. D. Geometric derivatives of excitation energies using SCF and DFT. *Chem. Phys. Lett.* **308**, 249–255 (1999).
30. Van Caillie, C. & Amos, R. D. Geometric derivatives of density functional theory excitation energies using gradient-corrected functionals. *Chem. Phys. Lett.* **317**, 159–164 (2000).
31. Furche, F. & Ahlrichs, R. Adiabatic time-dependent density functional methods for excited state properties. *J. Chem. Phys.* **117,** 7433–7447 (2002).
32. Scalmani, G. *et al.* Geometries and properties of excited states in the gas phase and in solution: theory and application of a time-dependent density functional theory polarizable continuum model. *J. Chem. Phys.* **124,** 94107 (2006).
33. O'Boyle, N. M., Tenderholt, A. L. & Langner, K. M., cclib: a library for package-independent computational chemistry algorithms. *J. Comp. Chem.* **29**, 839-845 (2008).
34. Hirshfeld, F. L. Bonded-atom fragments for describing molecular charge densities. *Theor. Chim. Acta* **44,** 129–138 (1977).
35. Ritchie, J. P. Electron density distribution analysis for nitromethane, nitromethide, and nitramide. *J. Am. Chem. Soc.* **107,** 1829–1837 (1985).
36. Ritchie, J. P. & Bachrach, S. M. Some methods and applications of electron density distribution analysis. *J. Comp. Chem.* **8**, 499–509 (1987).
37. Lu, T. & Chen, F. Multiwfn: a multifunctional wavefunction analyzer, *J. Comp. Chem.* **33**, 580-592 (2012).
38. Lu, T. & Chen, F. Calculation of molecular orbital composition, *Acta Chim. Sinica*, **69**, 2393-2406 (2011).
39. Grimme, S., Antony, J., Ehrlich, S. & Krieg, H. A consistent and accurate ab initio parametrization of density functional dispersion correction (DFT-D) for the 94 elements H-Pu. *J. Chem. Phys.* **132,** 154104–154120 (2010).
40. Peterson, K. A., Shepler, B. C., Figgen, D. & Stoll, H. On the Spectroscopic and Thermochemical Properties of ClO, BrO, IO, and Their Anions. *J. Phys. Chem. A* **110,** 13877–13883 (2006).

41. Boys, S. F. & Bernardi, F. The calculation of small molecular interactions by the differences of separate total energies. Some procedures with reduced errors. *Mol. Phys.* **19,** 553–566 (2006).
42. Simon, S., Duran, M. & Dannenberg, J. J. How does basis set superposition error change the potential surfaces for hydrogen-bonded dimers? *J. Chem. Phys.* **105,** 11024–11029 (1996).
43. Lipparini, F., Scalmani, G. & Mennucci, B. Non covalent interactions in RNA and DNA base pairs: a quantum-mechanical study of the coupling between solvent and electronic density. *Phys. Chem. Chem. Phys.* **11,** 11617–11617 (2009).
44. Zawada, A., Góra, R. W., Mikołajczyk, M. M. & Bartkowiak, W. On the Calculations of Interaction Energies and Induced Electric Properties within the Polarizable Continuum Model. *J. Phys. Chem. A* **116,** 4409–4416 (2012).
45. Feller, D. The role of databases in support of computational chemistry calculations. *J. Comput. Chem.* **17,** 1571–1586 (1996).
46. Schuchardt, K. L. *et al.* Basis set exchange: a community database for computational sciences. *J. Chem. Inf. Model.* **47,** 1045–1052 (2007).
47. Vosko, S. H., Wilk, L. & Nusair, M. Accurate spin-dependent electron liquid correlation energies for local spin density calculations: a critical analysis. *Can. J. Phys.* **58**, 1200–1211 (1980).
48. Lee, C., Yang, W. & Parr, R. G. Development of the Colle-Salvetti correlation-energy formula into a functional of the electron density. *Phys. Rev. B Condens. Matter* **37**, 785–789 (1988).
49. Becke, A. D. Density-functional thermochemistry. III. The role of exact exchange. *J. Chem. Phys.* **98**, 5648–5646 (1993).
50. Stephens, P. J., Devlin, F. J., Chabalowski, C. F. & Frisch, M. J. Ab Initio Calculation of Vibrational Absorption and Circular Dichroism Spectra Using Density Functional Force Fields. *J. Phys. Chem.* **98**, 11623–11627 (1994).
51. Zhao, Y. & Truhlar, D. G. The M06 suite of density functionals for main group thermochemistry, thermochemical kinetics, noncovalent interactions, excited states, and transition elements: two new functionals and systematic testing of four M06-class functionals and 12 other functionals. *Theor. Chem. Acc.* **120**, 215–241 (2007).
52. Zhao, Y. & Truhlar, D. G. Density Functionals with Broad Applicability in Chemistry. *Acc. Chem. Res.* **41**, 157–167 (2008).
53. Perdew, J. P. et al. Atoms, molecules, solids, and surfaces: Applications of the generalized gradient approximation for exchange and correlation. *Phys. Rev. B Condens. Matter* **46**, 6671 (1992).

54. Perdew, J. P. et al. Erratum: Atoms, molecules, solids, and surfaces: Applications of the generalized gradient approximation for exchange and correlation. *Phys. Rev. B Condens. Matter* **48**, 4978 (1993).
55. Adamo, C. & Barone, V. Exchange functionals with improved long-range behavior and adiabatic connection methods without adjustable parameters: The mPW and mPW1PW models. *J. Chem. Phys.* **108**, 664–613 (1998).
56. Perdew, J. P. Density-functional approximation for the correlation energy of the inhomogeneous electron gas. *Phys. Rev. B Condens. Matter* **33**, 8822–8824 (1986).
57. Becke, A. D. Density-functional exchange-energy approximation with correct asymptotic behavior. *Phys. Rev. A Gen. Phys.* **38**, 3098–3100 (1988).
58. Marcus, R. A. & Sutin, N. Electron transfers in chemistry and biology. *Biochimica et Biophysica Acta (BBA) - Reviews on Bioenergetics* **811,** 265–322 (1985).
59. Boschloo, G., Gibson, E. A. & Hagfeldt, A. Photomodulated Voltammetry of Iodide/Triiodide Redox Electrolytes and Its Relevance to Dye-Sensitized Solar Cells. *J. Phys. Chem. Lett.* **2**, 3016–3020 (2011).
60. Boschloo, G. & Hagfeldt, A. Characteristics of the iodide/triiodide redox mediator in dye-sensitized solar cells. *Acc. Chem. Res.* **42**, 1819–1826 (2009).
61. Anderson, A. Y., Barnes, P. R. F., Durrant, J. R. & O'Regan, B. C. Quantifying Regeneration in Dye-Sensitized Solar Cells. *J. Phys. Chem. C* **115**, 2439–2447 (2011).
62. Privalov, T., Boschloo, G., Hagfeldt, A., Svensson, P. H. & Kloo, L. A Study of the Interactions between I^-/I_3^- Redox Mediators and Organometallic Sensitizing Dyes in Solar Cells. *J. Phys. Chem. C* **113**, 783–790 (2009).
63. Marcus, R. A. On the Theory of Oxidation-Reduction Reactions Involving Electron Transfer. I. *J. Chem. Phys.* **24,** 966–978 (1956).
64. Sutin, N. Theory of Electron Transfer Reactions: Insights and Hindsights. in *Progress in Inorganic Chemistry* (ed. Lippard, S. J.) **30**, 441–498 (John Wiley & Sons Inc., 1983).
65. Prue, J. E. Ion pairs and complexes: Free energies, enthalpies, and entropies. *J. Chem. Educ.* **46,** 12 (1969).
66. Debye, P. Reaction Rates in Ionic Solutions. *Trans. Soc. Adv. Electrochem. Sci. Technol.* **82,** 265–272 (1942).
67. Vaissier, V., Barnes, P., Kirkpatrick, J. & Nelson, J. Influence of polar medium on the reorganization energy of charge transfer between dyes in a dye sensitized film. *Phys. Chem. Chem. Phys.* **15,** 4804–4814 (2013).
68. Jeon, J. *et al.* Rapid Dye Regeneration Mechanism of Dye-Sensitized Solar Cells. *J. Phys. Chem. Lett.* **5,** 4285–4290 (2014).

69. Mulliken, R. S., Rieke, C. A., Orloff, D. & Orloff, H. Formulas and Numerical Tables for Overlap Integrals. *J. Chem. Phys.* **17**, 1248–1267 (1949).
70. Slater, J. C. Atomic Shielding Constants. *Phys. Rev.* **36**, 57–64 (1930).
71. Mathematica, Version 10.1, Wolfram Research, Inc., Champaign, IL (2015).
