# Supplementary material for: Resolving orbital pathways for intermolecular electron transfer
Source: Nat Commun. 2018 Nov 21;9:4916. doi: 10.1038/s41467-018-07263-1 (PMC6249235; doi:10.1038/s41467-018-07263-1)
Supplement: Supplementary file 2 — Description Of Additional Supplementary Files [file 41467_2018_7263_MOESM2_ESM.docx]

**Description of Additional Supplementary Files**

**File Name: Supplementary Data 1:**

**Description:** **DFT Optimized Molecular Coordinates.​ This file contains the**

**density functional theory (DFT) optimized atomic coordinates for all computational**

**models employed in this study, along with their calculated electronic energies generated**

**using the indicated functional.**

**File Name: Supplementary Data 2:**

**Description:** **TD-DFT Calculated Optical Transitions.​ This file contains**

**details about the theoretical optical transitions for each ruthenium complex calculated**

**using time-dependent density functional theory (TD-DFT). Only the first transition and**

**the six most intense optical transitions below 400 nm are reported.**
