# Supplementary material for: Resolving orbital pathways for intermolecular electron transfer
Source: Nat Commun. 2018 Nov 21;9:4916. doi: 10.1038/s41467-018-07263-1 (PMC6249235; doi:10.1038/s41467-018-07263-1)
Supplement: Supplementary file 3 — Supplementary Data 1 [file 41467_2018_7263_MOESM3_ESM.pdf]

Supplementary Data 1 for

Resolving Orbital Pathways for Intermolecular Electron Transfer

DFT Optimized Molecular Coordinates

Cameron W. Kellett[1]; Wesley B. Swords[2]; Michael D. Turlington[2]; Gerald J. Meyer[2,*]; Curtis P. Berlinguette[1,3,4,*]

correspondence to: gjmeyer@email.unc.edu; cberling@chem.ubc.ca

[1]Department of Chemistry, 2036 Main Mall, University of British Columbia, Vancouver, BC V6T 1Z1, Canada.

[2]Department of Chemistry, University of North Carolina at Chapel Hill, Murray Hall 2202B, Chapel Hill, NC 27599-3290, USA.

[3]Department of Chemical and Biological Engineering, 2360 East Mall, University of British Columbia, Vancouver, BC V6T 1Z3, Canada.

[4]Stewart Blusson Quantum Matter Institute, 2355 East Mall, University of British Columbia, Vancouver, BC V6T 1Z4, Canada

O-Me geometry optimized using PBE0

E_{PBE0} = -2240.97874267 Hartrees

Ru	0.34518	-0.01673	0.03458	C	-1.08154	-4.65706	-0.02847
N	0.69394	-0.02567	2.10467	C	-1.97442	-3.59002	-0.05211
N	0.87920	-0.03829	-1.99428	H	-3.04800	-3.76767	-0.08907
N	-0.14137	-2.02552	0.01599	C	-1.48604	-2.28619	-0.02956
N	-1.66330	0.05379	-0.04022	C	-2.35941	-1.10501	-0.05591
N	-0.00041	2.02113	0.01124	C	-3.74828	-1.08644	-0.09234
C	2.95702	-0.10955	1.37007	H	-4.33825	-2.00236	-0.10441
C	2.30581	-0.08691	0.12123	C	-4.40245	0.14918	-0.11239
C	3.06651	-0.11586	-1.05727	C	-3.66620	1.33812	-0.09638
C	4.47011	-0.16712	-1.00579	H	-4.17217	2.30197	-0.11164
H	5.06213	-0.18802	-1.92150	C	-2.27768	1.25676	-0.05979
C	5.10055	-0.18952	0.24536	C	-1.32478	2.37489	-0.03591
C	4.35069	-0.16148	1.43462	C	-1.71719	3.70910	-0.06043
H	4.89577	-0.18168	2.38141	H	-2.77260	3.97849	-0.09885
C	2.01132	-0.07309	2.48782	C	-0.75348	4.71187	-0.03615
C	-0.25585	0.01224	3.05459	C	0.59333	4.34831	0.01223
H	-1.28708	0.04958	2.70078	H	1.38557	5.09546	0.03274
C	0.03033	0.00560	4.41209	C	0.92036	3.00121	0.03402
H	-0.78572	0.03811	5.13457	H	1.95965	2.67569	0.07161
C	1.36486	-0.04273	4.81278	C	-1.53613	-6.07498	-0.05224
H	1.62930	-0.04945	5.87185	O	-0.78278	-7.02189	-0.03752
C	2.35650	-0.08234	3.84224	O	-2.86562	-6.18588	-0.09193
H	3.40920	-0.12000	4.12462	C	-5.88885	0.14443	-0.14894
C	2.22700	-0.08751	-2.25487	O	-6.55900	-0.86452	-0.16573
C	0.01987	-0.00916	-3.02704	O	-6.40965	1.37425	-0.16087
H	-1.03910	0.02924	-2.76768	C	-1.20959	6.12934	-0.06487
C	0.42792	-0.02634	-4.35274	O	-2.37361	6.45848	-0.11098
H	-0.31877	-0.00071	-5.14694	O	-0.19485	6.99460	-0.03477
C	1.79394	-0.07669	-4.62979	O	6.44877	-0.23880	0.41875
H	2.15348	-0.09219	-5.66039	C	7.26175	-0.27086	-0.73635
C	2.69324	-0.10746	-3.57326	H	8.29840	-0.30862	-0.37760
H	3.76736	-0.14799	-3.75845	H	7.06268	-1.16401	-1.35357
C	0.70957	-3.06815	0.03867	H	7.12906	0.63233	-1.35684
H	1.76907	-2.81569	0.07502	H	-3.07571	-7.13922	-0.10496
C	0.28660	-4.38661	0.01764	H	-7.37997	1.27215	-0.18645
H	1.01311	-5.19864	0.03758	H	-0.58014	7.89156	-0.05530

S-Me geometry optimized using PBE0

E_{PBE0} = -2563.89783626 Hartrees

Ru	0.18850	-0.00930	0.04080	C	-1.15010	-4.67640	-0.03840
N	0.52660	-0.01420	2.11190	C	-2.06350	-3.62680	-0.06320
N	0.72520	-0.01790	-1.98730	H	-3.13350	-3.82430	-0.10580
N	-0.26090	-2.02770	0.01790	C	-1.59990	-2.31410	-0.03420
N	-1.82290	0.02200	-0.04140	C	-2.49580	-1.14980	-0.06110
N	-0.19820	2.02180	0.01660	C	-3.88470	-1.15850	-0.10140
C	2.79290	-0.04800	1.38560	H	-4.45610	-2.08610	-0.11660
C	2.14560	-0.03870	0.13470	C	-4.56240	0.06420	-0.12110
C	2.91030	-0.04930	-1.04400	C	-3.84970	1.26720	-0.10220
C	4.31150	-0.06920	-0.98440	H	-4.37490	2.22060	-0.11810
H	4.90430	-0.07680	-1.90010	C	-2.45990	1.21250	-0.06210
C	4.94460	-0.07840	0.26660	C	-1.52900	2.34900	-0.03610
C	4.18830	-0.06810	1.45370	C	-1.94800	3.67490	-0.06550
H	4.70760	-0.07590	2.41600	H	-3.00830	3.92300	-0.10890
C	1.84280	-0.03380	2.50070	C	-1.00450	4.69670	-0.04050
C	-0.42840	0.00030	3.05720	C	0.34890	4.36030	0.01480
H	-1.45850	0.01570	2.69850	H	1.12590	5.12320	0.03690
C	-0.14830	-0.00350	4.41600	C	0.70270	3.02000	0.04130
H	-0.96810	0.00890	5.13480	H	1.74820	2.71580	0.08400
C	1.18510	-0.02330	4.82250	C	-1.57620	-6.10310	-0.07060
H	1.44480	-0.02690	5.88280	O	-0.80440	-7.03480	-0.04600
C	2.18200	-0.03860	3.85670	O	-2.90240	-6.23980	-0.13090
H	3.23350	-0.05400	4.14530	C	-6.04870	0.03190	-0.16030
C	2.07370	-0.03740	-2.24500	O	-6.70040	-0.98890	-0.17960
C	-0.13220	-0.00630	-3.02200	O	-6.59100	1.25220	-0.17130
H	-1.19230	0.00880	-2.76530	C	-1.48860	6.10480	-0.07650
C	0.27980	-0.01280	-4.34660	O	-2.65880	6.41050	-0.12790
H	-0.46520	-0.00260	-5.14280	O	-0.49130	6.99000	-0.04700
C	1.64710	-0.03250	-4.62040	C	7.34330	-0.10690	-1.21720
H	2.00950	-0.03830	-5.65010	H	8.43820	-0.12100	-1.11890
C	2.54430	-0.04490	-3.56150	H	7.02390	-1.00430	-1.76700
H	3.61950	-0.06090	-3.74400	H	7.04660	0.80140	-1.76170
C	0.60980	-3.05360	0.04290	S	6.71360	-0.10380	0.47430
H	1.66410	-2.78150	0.08520	H	-0.89410	7.87910	-0.07250
C	0.21220	-4.37990	0.01660	H	-7.55950	1.13370	-0.19850
H	0.95410	-5.17780	0.03830	H	-3.09440	-7.19680	-0.14770

Se-Me geometry optimized using PBE0

E_{PBE0} = -4567.07984794 Hartrees

Ru	-0.13010	-0.00610	0.02380	C	-1.43300	-4.68390	-0.03030
N	0.23740	-0.00910	2.09010	C	-2.35500	-3.64160	-0.04590
N	0.37590	-0.00950	-2.01260	H	-3.42400	-3.84750	-0.07350
N	-0.56420	-2.02810	0.00760	C	-1.90140	-2.32520	-0.02650
N	-2.14310	0.00900	-0.03100	C	-2.80670	-1.16800	-0.04340
N	-0.53370	2.02190	0.00780	C	-4.19590	-1.18790	-0.06820
C	2.49290	-0.02290	1.33060	H	-4.75980	-2.12010	-0.07770
C	1.82790	-0.01930	0.08900	C	-4.88350	0.02930	-0.07980
C	2.57500	-0.02310	-1.10200	C	-4.18020	1.23800	-0.06780
C	3.97690	-0.03090	-1.06180	H	-4.71340	2.18710	-0.07710
H	4.55770	-0.03380	-1.98570	C	-2.78970	1.19440	-0.04320
C	4.62500	-0.03450	0.18030	C	-1.86770	2.33830	-0.02630
C	3.89020	-0.03050	1.37840	C	-2.29790	3.66080	-0.04560
H	4.42360	-0.03310	2.33310	H	-3.36070	3.90020	-0.07370
C	1.55930	-0.01740	2.45920	C	-1.36260	4.69020	-0.03010
C	-0.70330	-0.00370	3.04990	C	-0.00580	4.36470	0.00560
H	-1.73880	0.00280	2.70720	H	0.76510	5.13400	0.01960
C	-0.40260	-0.00590	4.40420	C	0.35920	3.02730	0.02330
H	-1.21150	-0.00120	5.13540	H	1.40770	2.73170	0.05100
C	0.93690	-0.01420	4.79070	C	-1.84800	-6.11410	-0.05190
H	1.21270	-0.01630	5.84690	O	-1.06820	-7.03930	-0.03460
C	1.91910	-0.02000	3.80980	O	-3.17360	-6.26170	-0.09340
H	2.97520	-0.02670	4.08200	C	-6.36980	-0.01420	-0.10330
C	1.72040	-0.01770	-2.29040	O	-7.01410	-1.03980	-0.11510
C	-0.49680	-0.00430	-3.03460	O	-6.92110	1.20210	-0.10910
H	-1.55320	0.00190	-2.76270	C	-1.85810	6.09460	-0.05460
C	-0.10430	-0.00660	-4.36510	O	-3.03110	6.39140	-0.08990
H	-0.86110	-0.00200	-5.15010	O	-0.86730	6.98720	-0.03450
C	1.25890	-0.01470	-4.65910	C	7.08780	-0.04200	-1.49700
H	1.60600	-0.01680	-5.69410	H	8.18660	-0.04640	-1.46770
C	2.17170	-0.02030	-3.61360	H	6.73170	-0.94420	-2.01280
H	3.24420	-0.02700	-3.81170	H	6.73860	0.86600	-2.00730
C	0.31490	-3.04700	0.02310	Se	6.53790	-0.04560	0.36190
H	1.36750	-2.76670	0.05070	H	-1.27720	7.87330	-0.05210
C	-0.07260	-4.37640	0.00540	H	-7.88890	1.07670	-0.12530
H	0.67590	-5.16840	0.01910	H	-3.35830	-7.22020	-0.10450

S-Ar geometry optimized using PBE0

E_{PBE0} = -2678.09843144 Hartrees

Ru	-0.20690	-0.01654	0.02046	H	-3.58093	-3.78795	-0.08757
N	0.17538	-0.04641	2.08409	C	-2.02718	-2.29779	-0.03983
N	0.27754	-0.00949	-2.02186	C	-2.90901	-1.12225	-0.04210
N	-0.68399	-2.02897	-0.01158	C	-4.29849	-1.11367	-0.06253
N	-2.22208	0.04065	-0.02031	H	-4.88194	-2.03363	-0.08071
N	-0.57097	2.01977	0.02870	C	-4.96095	0.11736	-0.05796
C	2.42297	-0.10339	1.30391	C	-4.23338	1.31127	-0.03487
C	1.74571	-0.07218	0.06870	H	-4.74648	2.27142	-0.03121
C	2.48283	-0.07717	-1.13118	C	-2.84378	1.23882	-0.01714
C	3.87852	-0.11950	-1.09635	C	-1.89825	2.36363	0.00668
H	4.45526	-0.14683	-2.02495	C	-2.30131	3.69464	0.00569
C	4.55752	-0.14627	0.13802	H	-3.35919	3.95606	-0.01247
C	3.81844	-0.13339	1.33906	C	-1.34496	4.70457	0.02826
H	4.35565	-0.13041	2.29019	C	0.00481	4.35114	0.05098
C	1.49989	-0.08594	2.44108	H	0.79162	5.10402	0.06913
C	-0.75640	-0.02822	3.05226	C	0.34255	3.00629	0.05024
H	-1.79454	0.00255	2.71897	H	1.38499	2.68952	0.06763
C	-0.44360	-0.04703	4.40368	C	-2.05422	-6.08650	-0.09433
H	-1.24557	-0.03053	5.14227	O	-1.29491	-7.02861	-0.09347
C	0.89886	-0.08717	4.77786	O	-3.38315	-6.20461	-0.12286
H	1.18388	-0.10332	5.83144	H	-3.58855	-7.15891	-0.14073
C	1.87188	-0.10706	3.78807	C	-6.44826	0.10272	-0.07750
H	2.93005	-0.13934	4.04995	O	-7.11110	-0.91072	-0.09953
C	1.61836	-0.04442	-2.31242	O	-6.97678	1.32889	-0.06777
C	-0.60404	0.02483	-3.03547	H	-7.94679	1.22141	-0.08270
H	-1.65759	0.05217	-2.75428	C	-1.81232	6.11893	0.02614
C	-0.22331	0.02593	-4.36947	O	-2.97955	6.43893	0.00675
H	-0.98680	0.05499	-5.14741	O	-0.80375	6.99134	0.04744
C	1.13638	-0.01095	-4.67617	H	-1.19584	7.88564	0.04460
H	1.47411	-0.01217	-5.71416	C	6.02379	-0.19503	0.19335
C	2.05824	-0.04642	-3.63903	C	6.83291	-0.69040	1.19302
H	3.12853	-0.07657	-3.84672	S	7.00487	0.44276	-1.10211
C	0.17374	-3.06597	-0.00951	C	8.22268	-0.56499	0.91544
H	1.23220	-2.80806	0.01376	H	6.43715	-1.15435	2.09727
C	-0.24144	-4.38702	-0.03544	C	8.47139	0.02577	-0.29374
H	0.49056	-5.19433	-0.03295	H	9.01080	-0.91022	1.58597
C	-1.60806	-4.66570	-0.06444	H	9.43057	0.24206	-0.76282
C	-2.50794	-3.60419	-0.06577				

Se-Ar geometry optimized using PBE0

E_{PBE0} = -4681.27517186 Hartrees

Ru	-0.52569	-0.02461	0.05460	H	-3.95561	-3.74004	-0.17328
N	-0.20943	-0.06307	2.12850	C	-2.38104	-2.27435	-0.06872
N	0.02248	-0.02514	-1.97080	C	-3.24284	-1.08400	-0.09211
N	-1.03507	-2.02785	0.00139	C	-4.63091	-1.05123	-0.15203
N	-2.53711	0.06712	-0.04742	H	-5.22985	-1.96064	-0.18800
N	-0.85451	2.01735	0.05600	C	-5.27208	0.19106	-0.16453
C	2.06063	-0.16037	1.42021	C	-4.52512	1.37225	-0.11821
C	1.42296	-0.11555	0.16455	H	-5.02117	2.34128	-0.12762
C	2.19727	-0.13360	-1.01159	C	-3.13807	1.27584	-0.05967
C	3.59001	-0.20219	-0.93344	C	-2.17444	2.38420	-0.00434
H	4.19642	-0.23724	-1.84277	C	-2.55402	3.72210	-0.01256
C	4.23170	-0.24662	0.32085	H	-3.60625	4.00205	-0.06104
C	3.45372	-0.21923	1.49798	C	-1.58140	4.71511	0.04209
H	3.96052	-0.22821	2.46558	C	-0.23919	4.33816	0.10355
C	1.10218	-0.12585	2.52782	H	0.55986	5.07696	0.14747
C	-1.17218	-0.02842	3.06533	C	0.07502	2.98765	0.10810
H	-2.19793	0.02098	2.69730	H	1.11081	2.65257	0.15553
C	-0.90438	-0.05241	4.42624	C	-2.46576	-6.06226	-0.14432
H	-1.72962	-0.02177	5.13826	O	-1.72043	-7.01539	-0.13022
C	0.42433	-0.11562	4.84383	O	-3.79518	-6.16105	-0.20620
H	0.67449	-0.13614	5.90619	H	-4.01300	-7.11238	-0.23468
C	1.42895	-0.15281	3.88660	C	-6.75824	0.20127	-0.22707
H	2.47688	-0.20299	4.18427	O	-7.43647	-0.80118	-0.27140
C	1.37083	-0.08454	-2.21931	O	-7.26702	1.43586	-0.22891
C	-0.82695	0.02363	-3.01092	H	-8.23770	1.34409	-0.27421
H	-1.88790	0.06979	-2.76140	C	-2.02377	6.13750	0.03046
C	-0.40536	0.01648	-4.33253	O	-3.18386	6.47825	-0.02652
H	-1.14366	0.05810	-5.13387	O	-1.00133	6.99185	0.08908
C	0.96239	-0.04491	-4.59687	H	-1.37702	7.89303	0.07752
H	1.33184	-0.05348	-5.62398	C	5.69373	-0.33282	0.42268
C	1.85114	-0.09583	-3.53186	C	6.44038	-0.86129	1.44980
H	2.92657	-0.14604	-3.70666	C	7.85616	-0.79637	1.28462
C	-0.19432	-3.07842	0.02304	H	5.97594	-1.32290	2.32358
H	0.86691	-2.83750	0.07946	C	8.27409	-0.21337	0.12256
C	-0.62948	-4.39238	-0.02297	H	8.55358	-1.19262	2.02577
H	0.08902	-5.21149	-0.00309	H	9.29684	-0.06283	-0.22249
C	-1.99891	-4.64870	-0.09411	Se	6.83605	0.34026	-0.91729
C	-2.88124	-3.57284	-0.11693				

O-Me⁺ geometry optimized using PBE0

E_{PBE0} = -2240.80996551 Hartrees

Ru	0.39997	-0.02367	0.03758	C	-1.13647	-4.63572	-0.03672
N	0.68499	-0.04162	2.12388	C	-2.02125	-3.56142	-0.05631
N	0.87364	-0.04389	-2.01084	H	-3.09623	-3.72895	-0.09000
N	-0.17706	-2.02306	0.00777	C	-1.51987	-2.26465	-0.03347
N	-1.67749	0.07438	-0.03937	C	-2.37839	-1.07054	-0.05633
N	0.01288	2.02194	0.00840	C	-3.76864	-1.03987	-0.09309
C	2.94750	-0.14810	1.38749	H	-4.36887	-1.94870	-0.10657
C	2.30940	-0.11593	0.12366	C	-4.40321	0.20294	-0.11203
C	3.05956	-0.14902	-1.07185	C	-3.65668	1.38216	-0.09536
C	4.44886	-0.21256	-1.02074	H	-4.14836	2.35291	-0.11076
H	5.04839	-0.23602	-1.93030	C	-2.26802	1.27871	-0.05849
C	5.07355	-0.24341	0.24380	C	-1.30252	2.38830	-0.03541
C	4.32782	-0.21221	1.44858	C	-1.67696	3.72580	-0.05777
H	4.88205	-0.23977	2.38853	H	-2.72847	4.00995	-0.09413
C	1.99680	-0.10559	2.50969	C	-0.69660	4.71297	-0.03314
C	-0.26929	0.00319	3.06198	C	0.64224	4.33291	0.01200
H	-1.29845	0.05407	2.70478	H	1.44650	5.06686	0.03194
C	0.01577	-0.01287	4.42275	C	0.95312	2.98014	0.03070
H	-0.80164	0.02588	5.14305	H	1.98808	2.64328	0.06567
C	1.34487	-0.07828	4.82602	C	-1.60513	-6.05331	-0.05920
H	1.60598	-0.09285	5.88553	O	-0.85594	-7.00176	-0.04898
C	2.34354	-0.12539	3.85796	O	-2.93365	-6.14837	-0.09128
H	3.39400	-0.17659	4.14509	C	-5.89635	0.21531	-0.15009
C	2.21568	-0.10864	-2.27485	O	-6.56893	-0.78933	-0.17174
C	0.00961	-0.00292	-3.03222	O	-6.39732	1.44947	-0.15714
H	-1.04780	0.04750	-2.77020	C	-1.13375	6.14057	-0.05923
C	0.41841	-0.02365	-4.36133	O	-2.29342	6.47913	-0.10995
H	-0.32954	0.01183	-5.15371	O	-0.10680	6.98706	-0.02128
C	1.77885	-0.09040	-4.64138	O	6.39792	-0.30307	0.41579
H	2.13550	-0.10958	-5.67254	C	7.24356	-0.33612	-0.72883
C	2.68468	-0.13361	-3.58577	H	8.26701	-0.37783	-0.33840
H	3.75729	-0.18821	-3.77415	H	7.04754	-1.22984	-1.34162
C	0.67007	-3.06593	0.02548	H	7.11988	0.57130	-1.34045
H	1.73198	-2.82709	0.05767	H	-3.15889	-7.09880	-0.10378
C	0.23146	-4.38101	0.00414	H	-7.37011	1.36770	-0.18475
H	0.95156	-5.19860	0.02030	H	-0.47298	7.89248	-0.04071

S-Me+ geometry optimized using PBE0

E_{PBE0} = -2563.72690949 Hartrees

Ru	-0.24272	-0.01087	0.04650	C	1.15868	-4.66646	-0.04959
N	-0.72043	-0.01380	-2.00117	C	2.07435	-3.61817	-0.06901
N	-0.52343	-0.02215	2.13222	H	3.14438	-3.81501	-0.10966
N	0.27492	-2.02720	0.01231	C	1.60986	-2.30780	-0.03673
N	1.83580	0.02572	-0.03656	C	2.50246	-1.13930	-0.05858
N	0.20425	2.02271	0.01766	C	3.89275	-1.15016	-0.09898
C	-2.90630	-0.04930	-1.05817	H	4.46453	-2.07708	-0.11674
C	-2.15359	-0.04247	0.13727	C	4.56399	0.07322	-0.11562
C	-2.78953	-0.05674	1.40197	C	3.85296	1.27422	-0.09582
C	-4.17299	-0.07877	1.46541	H	4.37458	2.22925	-0.11122
H	-4.70238	-0.09130	2.42090	C	2.46197	1.21185	-0.05631
C	-4.92455	-0.08483	0.26277	C	1.52990	2.34917	-0.03252
C	-4.29530	-0.06983	-1.00017	C	1.94486	3.67426	-0.06333
H	-4.89383	-0.07399	-1.91074	H	3.00419	3.92597	-0.10612
C	-2.06413	-0.03335	-2.26304	C	0.99534	4.69110	-0.04048
C	0.14241	0.00058	-3.02462	C	-0.35411	4.35218	0.01424
H	1.20142	0.01545	-2.76490	H	-1.13531	5.11061	0.03507
C	-0.26968	-0.00332	-4.35275	C	-0.70585	3.00956	0.04110
H	0.47743	0.00890	-5.14663	H	-1.75051	2.70512	0.08219
C	-1.63219	-0.02316	-4.63029	C	1.58465	-6.09713	-0.08661
H	-1.99135	-0.02711	-5.66073	O	0.80847	-7.02336	-0.06108
C	-2.53670	-0.03850	-3.57287	O	2.90847	-6.23034	-0.15226
H	-3.61092	-0.05520	-3.75902	C	6.05677	0.04300	-0.15446
C	-1.83543	-0.04535	2.52223	O	6.70138	-0.97977	-0.17740
C	0.43522	-0.00941	3.06693	O	6.59152	1.26295	-0.15988
H	1.46420	0.00911	2.70614	C	1.47580	6.10430	-0.08009
C	0.15462	-0.01873	4.42860	O	2.64517	6.40676	-0.13624
H	0.97539	-0.00746	5.14605	O	0.47522	6.98208	-0.04846
C	-1.17450	-0.04234	4.83637	C	-7.32175	-0.11256	-1.20967
H	-1.43231	-0.05031	5.89675	H	-8.41361	-0.12975	-1.08668
C	-2.17765	-0.05592	3.87192	H	-7.03394	0.80020	-1.74996
H	-3.22772	-0.07436	4.16433	H	-7.00629	-1.01018	-1.75979
C	-0.60152	-3.04540	0.03264	S	-6.66409	-0.11179	0.47036
H	-1.65598	-2.77677	0.07223	H	0.86937	7.87554	-0.07654
C	-0.20092	-4.37237	0.00367	H	7.56177	1.15474	-0.18757
H	-0.94418	-5.16891	0.02159	H	3.10687	-7.18649	-0.17204

Se-Me⁺ geometry optimized using PBE0

E_{PBE0} = -4566.90873624 Hartrees

Ru	0.07442	-0.00525	0.02809	C	1.41888	-4.67882	-0.03521
N	-0.37334	-0.00575	-2.02711	C	2.34723	-3.64163	-0.04721
N	-0.23824	-0.00817	2.10937	H	3.41534	-3.85125	-0.07363
N	0.56594	-2.02842	0.00590	C	1.89800	-2.32557	-0.02580
N	2.15283	0.00518	-0.02644	C	2.80522	-1.16818	-0.03962
N	0.54550	2.02267	0.00743	C	4.19564	-1.19637	-0.06415
C	-2.57338	-0.01539	-1.11737	H	4.75574	-2.13049	-0.07470
C	-1.83737	-0.01338	0.08993	C	4.88223	0.01858	-0.07441
C	-2.49300	-0.01660	1.34517	C	4.18594	1.22840	-0.06290
C	-3.87809	-0.02254	1.38821	H	4.72014	2.17654	-0.07248
H	-4.42181	-0.02517	2.33562	C	2.79400	1.18350	-0.03872
C	-4.60998	-0.02561	0.17531	C	1.87594	2.33231	-0.02443
C	-3.96302	-0.02158	-1.07852	C	2.30863	3.65198	-0.04557
H	-4.54930	-0.02329	-1.99754	H	3.37161	3.88972	-0.07295
C	-1.71309	-0.01090	-2.30913	C	1.37234	4.68116	-0.03313
C	0.50448	-0.00146	-3.03812	C	0.01791	4.35960	0.00211
H	1.55975	0.00238	-2.76340	H	-0.75339	5.12828	0.01416
C	0.11221	-0.00193	-4.37212	C	-0.35151	3.02166	0.02091
H	0.87119	0.00172	-5.15478	H	-1.40048	2.73075	0.04690
C	-1.24629	-0.00721	-4.66995	C	1.82729	-6.11471	-0.06073
H	-1.59012	-0.00786	-5.70561	O	1.03913	-7.03096	-0.04112
C	-2.16625	-0.01182	-3.62603	O	3.15009	-6.26504	-0.10898
H	-3.23782	-0.01632	-3.82755	C	6.37474	-0.02936	-0.09701
C	-1.55628	-0.01357	2.47907	O	7.00810	-1.05939	-0.10975
C	0.70536	-0.00532	3.05968	O	6.92348	1.18451	-0.10071
H	1.74018	-0.00118	2.71584	C	1.87098	6.08813	-0.06253
C	0.40312	-0.00752	4.41665	O	3.04454	6.37654	-0.10437
H	1.21252	-0.00505	5.14700	O	0.88108	6.97830	-0.03938
C	-0.93262	-0.01292	4.80361	C	-7.06431	-0.03903	-1.48724
H	-1.20714	-0.01481	5.85980	H	-8.16173	-0.04493	-1.43586
C	-1.92010	-0.01603	3.82333	H	-6.72159	0.87002	-1.99914
H	-2.97514	-0.02036	4.09793	H	-6.71175	-0.94419	-1.99930
C	-0.32257	-3.03638	0.01887	Se	-6.48793	-0.03623	0.36007
H	-1.37414	-2.75514	0.04456	H	1.28680	7.86675	-0.06080
C	0.06231	-4.36810	-0.00023	H	7.89267	1.06532	-0.11650
H	-0.69063	-5.15560	0.01107	H	3.33664	-7.22363	-0.12269

S-Ar^{+} geometry optimized using PBE0

E_{PBE0} = -2677.92414424 Hartrees

Ru	-0.15474	-0.01022	0.02976
N	0.17036	-0.02109	2.11048
N	0.28220	-0.00778	-2.02940
N	-0.66715	-2.02860	0.00594
N	-2.23762	0.01662	-0.01774
N	-0.61531	2.02070	0.01722
C	2.42056	-0.04147	1.33027
C	1.75789	-0.03060	0.08132
C	2.48592	-0.03084	-1.12952
C	3.87205	-0.04372	-1.08886
H	4.44888	-0.04984	-2.01627
C	4.54816	-0.05479	0.15724
C	3.80581	-0.05276	1.36744
H	4.33658	-0.05361	2.31971
C	1.49101	-0.03562	2.47049
C	-0.76610	-0.01492	3.06744
H	-1.80337	-0.00379	2.73152
C	-0.45360	-0.02243	4.42229
H	-1.25757	-0.01685	5.15857
C	0.88470	-0.03709	4.79931
H	1.16699	-0.04344	5.85342
C	1.86497	-0.04394	3.81157
H	2.92220	-0.05574	4.07750
C	1.62048	-0.01930	-2.31778
C	-0.60050	0.00365	-3.03590
H	-1.65442	0.01248	-2.75641
C	-0.21429	0.00412	-4.37175
H	-0.97686	0.01369	-5.15083
C	1.14258	-0.00802	-4.67585
H	1.48165	-0.00840	-5.71306
C	2.06765	-0.01994	-3.63628
H	3.13823	-0.03025	-3.84293
C	0.21467	-3.04233	0.01515
H	1.26836	-2.76893	0.03849
C	-0.18002	-4.37125	-0.00519
H	0.56742	-5.16401	0.00288
C	-1.53879	-4.67194	-0.03710
C	-2.45978	-3.62815	-0.04512
H	-3.52939	-3.83000	-0.06906
C	-2.00110	-2.31556	-0.02306
C	-2.89907	-1.15124	-0.03315
C	-4.28967	-1.16874	-0.05689
H	-4.85702	-2.09845	-0.06968
C	-4.96673	0.05142	-0.06339
C	-4.26119	1.25565	-0.04896
H	-4.78742	2.20825	-0.05556
C	-2.86957	1.19956	-0.02645
C	-1.94287	2.34117	-0.01082
C	-2.36435	3.66440	-0.02661
H	-3.42536	3.91127	-0.05089
C	-1.41915	4.68540	-0.01227
C	-0.06754	4.35244	0.01865
H	0.71042	5.11434	0.03153
C	0.29078	3.01137	0.03206
H	1.33742	2.71202	0.05474
C	-1.95842	-6.10482	-0.06366
O	-1.17738	-7.02712	-0.04827
O	-3.28244	-6.24422	-0.10750
H	-3.47701	-7.20125	-0.12209
C	-6.45987	0.01452	-0.08585
O	-7.10007	-1.01111	-0.10204
O	-6.99965	1.23216	-0.08534
H	-7.96974	1.12018	-0.10168
C	-1.90619	6.09681	-0.03403
O	-3.07759	6.39451	-0.06803
O	-0.90879	6.97840	-0.01333
H	-1.30683	7.87046	-0.02938
C	6.00139	-0.06888	0.21140
C	6.82257	-0.17058	1.32268
S	6.97557	0.05098	-1.23519
C	8.20203	-0.15372	1.01054
H	6.44290	-0.26162	2.34002
C	8.43764	-0.03766	-0.33654
H	8.99666	-0.22608	1.75331
H	9.39433	0.00242	-0.85651

Se-Ar⁺ geometry optimized using PBE0

E_{PBE0} = -4681.10155629 Hartrees

Ru	-0.47277	-0.02365	0.06605	H	-3.93101	-3.75924	-0.15247
N	-0.23114	-0.03915	2.15664	C	-2.36983	-2.28120	-0.05008
N	0.04278	-0.04025	-1.97220	C	-3.23860	-1.09485	-0.08464
N	-1.03058	-2.02741	0.02155	C	-4.62820	-1.07696	-0.14443
N	-2.54865	0.05647	-0.05314	H	-5.21929	-1.99152	-0.16958
N	-0.87726	2.01872	0.02862	C	-5.27387	0.16002	-0.17033
C	2.04778	-0.09328	1.46757	C	-4.53866	1.34607	-0.13982
C	1.43279	-0.08088	0.19329	H	-5.03944	2.31204	-0.16136
C	2.20937	-0.09206	-0.98830	C	-3.14988	1.25519	-0.08096
C	3.59184	-0.11483	-0.89388	C	-2.19486	2.37309	-0.04163
H	4.20523	-0.12632	-1.79739	C	-2.58118	3.70683	-0.07591
C	4.22281	-0.12346	0.37723	H	-3.63431	3.98100	-0.13408
C	3.42996	-0.11406	1.55746	C	-1.61093	4.70336	-0.03553
H	3.92134	-0.11403	2.53047	C	-0.26987	4.33592	0.03766
C	1.07341	-0.07379	2.57053	H	0.52709	5.07731	0.07117
C	-1.20711	-0.02107	3.07308	C	0.05309	2.98616	0.06690
H	-2.22878	0.00612	2.69250	H	1.09051	2.65997	0.12305
C	-0.95242	-0.03517	4.43977	C	-2.41761	-6.07012	-0.10802
H	-1.78635	-0.01927	5.14176	O	-1.66001	-7.01153	-0.07956
C	0.36869	-0.06958	4.87218	O	-3.74330	-6.17733	-0.18418
H	0.60662	-0.08164	5.93717	H	-3.95943	-7.12958	-0.20890
C	1.38966	-0.08933	3.92674	C	-6.76630	0.15930	-0.23032
H	2.43368	-0.11754	4.23928	O	-7.42969	-0.85103	-0.26560
C	1.39066	-0.07415	-2.20928	O	-7.27787	1.38911	-0.23971
C	-0.80182	-0.02479	-3.01095	H	-8.24942	1.29908	-0.28272
H	-1.86501	0.00148	-2.76955	C	-2.06099	6.12657	-0.07629
C	-0.36612	-0.04086	-4.33124	O	-3.22287	6.45382	-0.14683
H	-1.09851	-0.02720	-5.13868	O	-1.04269	6.98288	-0.02730
C	1.00110	-0.07397	-4.58383	H	-1.41712	7.88467	-0.05658
H	1.37868	-0.08729	-5.60761	C	5.67146	-0.13624	0.48592
C	1.88639	-0.09088	-3.51040	C	6.43363	-0.22829	1.63802
H	2.96357	-0.11816	-3.67706	C	7.83785	-0.21140	1.44885
C	-0.17405	-3.06215	0.05239	H	5.98924	-0.31513	2.63021
H	0.88464	-2.81377	0.10965	C	8.23817	-0.10339	0.14232
C	-0.59971	-4.38104	0.01270	H	8.54581	-0.27995	2.27655
H	0.12756	-5.19196	0.03977	H	9.25816	-0.06951	-0.24053
C	-1.96383	-4.64825	-0.06200	Se	6.80287	-0.01145	-1.01983
C	-2.85848	-3.58234	-0.09338				

O-Me^{+} (for cat^{+}···I^{-} adduct model) geometry optimized using PBE0

$E_{PBE0\text{-}D3}$ = -2241.404663 Hartrees

Ru	0.39609400	-0.02536900	0.03113500	C	-1.13682200	-4.62688900	-0.03816400
N	0.65162800	-0.03320400	2.11016200	C	-2.02309200	-3.55369200	-0.05846600
N	0.87996100	-0.05624100	-2.00510000	H	-3.09799600	-3.72200300	-0.09142000
N	-0.17904800	-2.01675900	0.00309300	C	-1.52208900	-2.25732100	-0.03763300
N	-1.67394600	0.08070100	-0.05200000	C	-2.37815400	-1.06202600	-0.06263600
N	0.02744600	2.01457100	-0.00797000	C	-3.76786900	-1.02563100	-0.09442900
C	2.92341100	-0.15294600	1.40655800	H	-4.37126900	-1.93236500	-0.10304100
C	2.30095500	-0.12375300	0.13552300	C	-4.39664000	0.22036200	-0.11454800
C	3.05940000	-0.16602800	-1.05354900	C	-3.64566800	1.39708900	-0.10305800
C	4.44740100	-0.23621500	-0.98880300	H	-4.13282500	2.37004200	-0.11842200
H	5.05410900	-0.26690900	-1.89283900	C	-2.25780600	1.28806900	-0.07082700
C	5.05671900	-0.26450600	0.28258600	C	-1.28516500	2.39100900	-0.05099200
C	4.30271200	-0.22446400	1.48124500	C	-1.64761100	3.73126500	-0.07466800
H	4.84608100	-0.25135800	2.42759000	H	-2.69653100	4.02505300	-0.10995900
C	1.95752900	-0.09947400	2.51576200	C	-0.65739200	4.70876300	-0.05248900
C	-0.31954100	0.02216100	3.02937400	C	0.67862300	4.31748400	-0.00972500
H	-1.34124300	0.07505600	2.65072900	H	1.48859900	5.04517700	0.00744600
C	-0.05663300	0.01433800	4.39462200	C	0.97799300	2.96240200	0.01044100
H	-0.88507300	0.06113700	5.10166000	H	2.00916600	2.61342100	0.04398000
C	1.26595600	-0.05304400	4.81969500	C	-1.60420400	-6.04479800	-0.05864800
H	1.50928000	-0.06087800	5.88347900	O	-0.85356000	-6.99223000	-0.04802400
C	2.28152800	-0.11044100	3.86927300	O	-2.93292000	-6.14045000	-0.08941400
H	3.32689600	-0.16262900	4.17408300	C	-5.88977400	0.23860100	-0.14730500
C	2.22319500	-0.12728100	-2.26189200	O	-6.56602300	-0.76380900	-0.16364400
C	0.01830700	-0.01673400	-3.02797600	O	-6.38533100	1.47516700	-0.15607900
H	-1.03920500	0.03849100	-2.76661400	C	-1.08208200	6.14005900	-0.07837800
C	0.43264700	-0.04485800	-4.35517400	O	-2.23900400	6.48827800	-0.12915900
H	-0.31155300	-0.01031000	-5.15108300	O	-0.04723800	6.97710100	-0.03966600
C	1.79442300	-0.11759100	-4.62921300	O	6.38417600	-0.33028400	0.46476000
H	2.15461500	-0.14248200	-5.65898600	C	7.23414800	-0.37093500	-0.68113800
C	2.69712600	-0.15954200	-3.57039100	H	8.25657000	-0.41704900	-0.28861400
H	3.77030900	-0.21888600	-3.75396800	H	7.03320200	-1.26553900	-1.29024400
C	0.67022400	-3.05766600	0.02095200	H	7.11514200	0.53606900	-1.29358700
H	1.73165100	-2.81605800	0.05206100	H	-3.15942700	-7.09047100	-0.10074800
C	0.23146800	-4.37243900	0.00112900	H	-7.35856200	1.39938900	-0.17973500
H	0.95071900	-5.19077600	0.01731600	H	-0.40323500	7.88643700	-0.05884600

S-Me$^+$ (for cat$^+$···I$^-$ adduct model) geometry optimized using PBE0

$E_{PBE0-D3}$ = -2564.315475 Hartrees

Ru	-0.23918800	-0.01320600	0.03752400	C	1.16150900	-4.65713400	-0.05486900
N	-0.72525800	-0.02078400	-1.99888200	C	2.07802900	-3.60941500	-0.07318800
N	-0.49203200	-0.02136700	2.11546000	H	3.14813600	-3.80635700	-0.11097100
N	0.27758900	-2.02099600	0.00245300	C	1.61308800	-2.29973700	-0.04373100
N	1.83198600	0.03306700	-0.04893100	C	2.50269000	-1.12946600	-0.06546100
N	0.18749800	2.01563200	0.00367700	C	3.89265400	-1.13360600	-0.09974900
C	-2.90561300	-0.06176700	-1.04535100	H	4.46838500	-2.05810300	-0.11347400
C	-2.14542000	-0.05121100	0.14458000	C	4.55742600	0.09361400	-0.11541800
C	-2.76698900	-0.06595400	1.41602300	C	3.84116100	1.29184600	-0.09943900
C	-4.14944800	-0.09342400	1.49150300	H	4.35737800	2.24977300	-0.11292200
H	-4.67026100	-0.10749200	2.45191000	C	2.45079200	1.22288100	-0.06573200
C	-4.90962400	-0.10334000	0.29384600	C	1.51057600	2.35312500	-0.04391500
C	-4.29334200	-0.08689900	-0.97566400	C	1.91292700	3.68157600	-0.07297800
H	-4.89815700	-0.09355400	-1.88174400	H	2.96991700	3.94357900	-0.11308600
C	-2.07013700	-0.04454200	-2.25472800	C	0.95266200	4.68847400	-0.05179700
C	0.13593200	-0.00581600	-3.02312700	C	-0.39414700	4.33742700	-0.00147300
H	1.19504300	0.01209200	-2.76328500	H	-1.18164700	5.08931400	0.01702700
C	-0.28099900	-0.01288700	-4.34969000	C	-0.73377100	2.99195400	0.02430100
H	0.46292800	-0.00002100	-5.14651600	H	-1.77496700	2.67482000	0.06264100
C	-1.64485200	-0.03664200	-4.62237900	C	1.58732800	-6.08778600	-0.08840300
H	-2.00693000	-0.04310100	-5.65175600	O	0.81030600	-7.01350700	-0.06496500
C	-2.54701900	-0.05287500	-3.56249000	O	2.91169100	-6.22064800	-0.14811200
H	-3.62189300	-0.07292500	-3.74450200	C	6.05037700	0.06978400	-0.14839500
C	-1.79839800	-0.04869200	2.52421000	O	6.69885900	-0.95077000	-0.16742200
C	0.48325500	-0.00183100	3.03184300	O	6.57963800	1.29234600	-0.15349900
H	1.50495000	0.02038700	2.65028000	C	1.42010000	6.10602300	-0.08805800
C	0.22419100	-0.00869300	4.39773800	O	2.58711100	6.41861700	-0.14028300
H	1.05571400	0.00797900	5.10252300	O	0.41087100	6.97420700	-0.05779700
C	-1.09868300	-0.03676300	4.82630500	C	-7.31453300	-0.14141000	-1.16588700
H	-1.33916900	-0.04289400	5.89073800	H	-8.40568300	-0.16285300	-1.03990700
C	-2.11846800	-0.05692700	3.87887500	H	-7.03071000	0.77199400	-1.70645100
H	-3.16344000	-0.07838200	4.18855200	H	-6.99581700	-1.03786300	-1.71536100
C	-0.60036900	-3.03768800	0.02072500	S	-6.64891200	-0.13698100	0.51210000
H	-1.65449000	-2.76680200	0.05704500	H	0.79480400	7.87202600	-0.08331800
C	-0.19877200	-4.36412800	-0.00619600	H	7.55050900	1.19005500	-0.17706000
H	-0.94075900	-5.16188200	0.01005600	H	3.11207500	-7.17630500	-0.16589600

Se-Me^{+} (for cat^{+}···I^{-} adduct model) geometry optimized using PBE0

$E_{PBE0-D3}$ = -4567.554504 Hartrees

Ru	0.07714500	-0.00587100	0.01111600
N	-0.38136800	-0.00810200	-2.03237400
N	-0.20454500	-0.00669200	2.08485300
N	0.56026800	-2.02220500	-0.01227000
N	2.14834300	0.00656700	-0.04424600
N	0.53548400	2.01588900	-0.01276700
C	-2.57530000	-0.01767200	-1.10980400
C	-1.83025700	-0.01457800	0.09109600
C	-2.46971800	-0.01695000	1.35420000
C	-3.85388900	-0.02356700	1.41103200
H	-4.38801900	-0.02583400	2.36413300
C	-4.59655800	-0.02801700	0.20405000
C	-3.96388100	-0.02450100	-1.05746100
H	-4.55739600	-0.02711900	-1.97157600
C	-1.72276000	-0.01367100	-2.30712200
C	0.49396100	-0.00449700	-3.04492900
H	1.54959700	-0.00043300	-2.77087600
C	0.09559400	-0.00597300	-4.37709200
H	0.85074900	-0.00288300	-5.16341300
C	-1.26464700	-0.01152300	-4.66883400
H	-1.61236000	-0.01291500	-5.70317500
C	-2.18144500	-0.01549000	-3.62160800
H	-3.25395200	-0.02023000	-3.81819700
C	-1.51678300	-0.01229500	2.47507500
C	0.75778200	-0.00174400	3.01535500
H	1.78524500	0.00276600	2.64885300
C	0.47902700	-0.00225700	4.37730100
H	1.30051300	0.00185800	5.09396300
C	-0.85029300	-0.00810800	4.78703300
H	-1.10587600	-0.00874300	5.84796200
C	-1.85639100	-0.01318100	3.82515400
H	-2.90617000	-0.01770400	4.11894000
C	-0.33363900	-3.02516000	-0.00297800
H	-1.38375200	-2.73755900	0.01636500
C	0.04716400	-4.35781500	-0.01753300
H	-0.70754700	-5.14363300	-0.00910100
C	1.40325900	-4.67270200	-0.04339600
C	2.33648200	-3.63970000	-0.05323000
H	3.40391900	-3.85347400	-0.07353900
C	1.89180800	-2.32260100	-0.03756000
C	2.80040600	-1.16679800	-0.04914100
C	4.19051300	-1.19343500	-0.06142600
H	4.75103200	-2.12732100	-0.06552600
C	4.87522300	0.02290000	-0.06712100
C	4.17820900	1.23263300	-0.06151400
H	4.71070100	2.18171800	-0.06563800
C	2.78665300	1.18626600	-0.04939500
C	1.86473400	2.33152400	-0.03819000
C	2.29006300	3.65303900	-0.05406400
H	3.35181600	3.89702100	-0.07506300
C	1.34684400	4.67608400	-0.04429100
C	-0.00640200	4.34755800	-0.01812200
H	-0.78106800	5.11283200	-0.00944300
C	-0.36896000	3.00796900	-0.00333100
H	-1.41580000	2.70845900	0.01651400
C	1.80581300	-6.11020300	-0.06126900
O	1.01298600	-7.02264400	-0.04602000
O	3.12864900	-6.26561200	-0.09677000
C	6.36781200	-0.02396200	-0.07693100
O	7.00152600	-1.05398100	-0.08243200
O	6.91523000	1.19074200	-0.07796700
C	1.83802900	6.08567700	-0.06559800
O	3.01064600	6.37972900	-0.09660400
O	0.84266600	6.97015800	-0.04809300
C	-7.06114300	-0.04514600	-1.44135800
H	-8.15826200	-0.05227300	-1.38627200
H	-6.72010600	0.86349600	-1.95496000
H	-6.70814700	-0.95004500	-1.95343600
Se	-6.47424100	-0.03996900	0.40403300
H	1.24184700	7.86156500	-0.06358200
H	7.88473200	1.07423200	-0.08439200
H	3.31338400	-7.22447500	-0.10603200

S-Ar$^+$ (for cat$^+$···I$^-$ adduct model) geometry optimized using PBE0

$E_{PBE0\text{-}D3}$ = -2678.537836 Hartrees

Ru	-0.15805700	-0.01662200	0.01557300	H	-3.55325700	-3.80689100	-0.06614300
N	0.14294100	-0.02894100	2.08789500	C	-2.01628400	-2.30127600	-0.03212800
N	0.28254900	-0.01573700	-2.03338800	C	-2.90487200	-1.13021800	-0.04011300
N	-0.68018400	-2.02354400	-0.00940600	C	-4.29518300	-1.13315400	-0.05457500
N	-2.23291300	0.03149100	-0.03040100	H	-4.87179700	-2.05716000	-0.06291900
N	-0.58636700	2.01192800	0.00326600	C	-4.95896000	0.09462400	-0.05715100
C	2.40117500	-0.06110800	1.33378100	C	-4.24149000	1.29214500	-0.04654400
C	1.74981900	-0.04808100	0.07908400	H	-4.75680200	2.25065300	-0.04854000
C	2.48301400	-0.04970200	-1.12816800	C	-2.85083800	1.22170200	-0.03320300
C	3.86807100	-0.06744200	-1.07866200	C	-1.90952000	2.35101700	-0.01930300
H	4.45051300	-0.07484300	-2.00243200	C	-2.31073200	3.68003300	-0.02943400
C	4.53462400	-0.08170000	0.17312200	H	-3.36796500	3.94335500	-0.04850000
C	3.78564500	-0.07736400	1.37985300	C	-1.34896200	4.68563900	-0.01555200
H	4.31024600	-0.08005700	2.33536700	C	-0.00199200	4.33276700	0.00826400
C	1.45914100	-0.05023300	2.46420100	H	0.78663400	5.08356300	0.01978300
C	-0.80918400	-0.01604900	3.02845800	C	0.33655900	2.98678300	0.01671900
H	-1.84043500	0.00049000	2.67334800	H	1.37801300	2.66886600	0.03455100
C	-0.51575200	-0.02366000	4.38748900	C	-1.99443500	-6.08931500	-0.06031700
H	-1.32951500	-0.01248100	5.11281800	O	-1.21817500	-7.01586900	-0.05149200
C	0.81751200	-0.04571000	4.78295900	O	-3.32001800	-6.22043500	-0.08775700
H	1.08432100	-0.05248500	5.84107400	H	-3.52224200	-7.17582500	-0.09777100
C	1.81325400	-0.05934100	3.81021400	C	-6.45239300	0.07199800	-0.07032100
H	2.86616500	-0.07682100	4.09219900	O	-7.10168100	-0.94811500	-0.08189300
C	1.62141100	-0.03293400	-2.31909300	O	-6.98058000	1.29494200	-0.06761700
C	-0.60097500	0.00058100	-3.03854800	H	-7.95179000	1.19360200	-0.07738700
H	-1.65432200	0.01327100	-2.75651100	C	-1.81511100	6.10409400	-0.02915900
C	-0.21316400	0.00082800	-4.37388200	O	-2.98254400	6.41830800	-0.05470700
H	-0.97450500	0.01445500	-5.15407500	O	-0.80431300	6.97070100	-0.01051300
C	1.14440500	-0.01686100	-4.67653700	H	-1.18733100	7.86923100	-0.02062400
H	1.48391600	-0.01761700	-5.71357200	C	5.98700500	-0.09987300	0.23602200
C	2.06968800	-0.03407600	-3.63675500	C	6.80137700	-0.20335600	1.35208200
H	3.14051600	-0.04919000	-3.84198700	S	6.97172800	0.01626500	-1.20435300
C	0.19760400	-3.04056700	-0.00355400	C	8.18300600	-0.19051100	1.04944900
H	1.25241600	-2.77055200	0.01351800	H	6.41402400	-0.29280400	2.36664000
C	-0.20536400	-4.36682900	-0.01917500	C	8.42910700	-0.07562300	-0.29613200
H	0.53640300	-5.16492100	-0.01366400	H	8.97213400	-0.26483700	1.79795400
C	-1.56649300	-4.65893300	-0.04201300	H	9.38868000	-0.03838600	-0.81101600
C	-2.48252200	-3.61064800	-0.04831300				

Se-Ar+ (for cat+···I− adduct model) geometry optimized using PBE0

$E_{PBE0\text{-}D3}$ = -4681.772062 Hartrees

Ru	-0.47587500	-0.02736700	0.05919600	H	-3.93968200	-3.74748600	-0.14537900
N	-0.24802400	-0.03818200	2.14091100	C	-2.37593200	-2.27191300	-0.04999300
N	0.03548200	-0.04951500	-1.97092200	C	-3.24004600	-1.08264000	-0.08549900
N	-1.03572700	-2.02180100	0.01831800	C	-4.62917800	-1.05617100	-0.14160200
N	-2.54449200	0.06520200	-0.05853600	H	-5.22552500	-1.96735000	-0.16321000
N	-0.85770700	2.01058600	0.01936500	C	-5.26652100	0.18537700	-0.16868100
C	2.03468300	-0.10129700	1.46773900	C	-4.52445900	1.36751000	-0.14138400
C	1.42538200	-0.09177200	0.19107400	H	-5.01869800	2.33676600	-0.16298500
C	2.20214100	-0.10598500	-0.98976500	C	-3.13660900	1.26829000	-0.08540200
C	3.58363500	-0.13024700	-0.89255300	C	-2.17208700	2.37770300	-0.04738400
H	4.19818500	-0.14374700	-1.79522600	C	-2.54402900	3.71512400	-0.07822700
C	4.21053900	-0.13704800	0.38109400	H	-3.59429800	4.00098400	-0.13263300
C	3.41615900	-0.12357900	1.56073400	C	-1.56193400	4.70018300	-0.03873900
H	3.90496800	-0.12098800	2.53492000	C	-0.22414700	4.31882800	0.02901300
C	1.05347400	-0.07556600	2.56471100	H	0.58001700	5.05244300	0.06058800
C	-1.23363100	-0.01365100	3.04608900	C	0.08484000	2.96605000	0.05549800
H	-2.25053900	0.01599000	2.65256000	H	1.11814300	2.62585100	0.10796300
C	-0.99093700	-0.02410300	4.41491500	C	-2.42854800	-6.05964900	-0.09786600
H	-1.83060300	-0.00314000	5.10984500	O	-1.67134400	-7.00155600	-0.07073300
C	0.32666600	-0.06135700	4.85887100	O	-3.75501400	-6.16490500	-0.16731800
H	0.55474000	-0.07059300	5.92602600	H	-3.97428800	-7.11639900	-0.18903400
C	1.35716200	-0.08746200	3.92338700	C	-6.75892300	0.19332500	-0.22629200
H	2.39811000	-0.11773800	4.24566000	O	-7.42778500	-0.81361700	-0.25914900
C	1.38290900	-0.08743500	-2.21010200	O	-7.26293100	1.42649400	-0.23676100
C	-0.81355200	-0.03328300	-3.00547300	H	-8.23506500	1.34408900	-0.27839100
H	-1.87541600	-0.00406800	-2.75792800	C	-1.99681200	6.12813400	-0.07429100
C	-0.38086700	-0.05230800	-4.32667800	O	-3.15544700	6.46754400	-0.14284500
H	-1.11484100	-0.03797600	-5.13263900	O	-0.96883200	6.97294900	-0.02274300
C	0.98609500	-0.08912700	-4.58256100	H	-1.33139700	7.87951900	-0.04881500
H	1.36047300	-0.10471200	-5.60744800	C	5.65866700	-0.15070200	0.49298000
C	1.87530800	-0.10696600	-3.51192900	C	6.41760000	-0.24283100	1.64716500
H	2.95215700	-0.13722800	-3.68043900	C	7.82248600	-0.22654100	1.46274200
C	-0.17915200	-3.05633100	0.04885100	H	5.96911700	-0.32934700	2.63749200
H	0.87963600	-2.80715200	0.10280800	C	8.22809600	-0.11902800	0.15753900
C	-0.60767200	-4.37410900	0.01299100	H	8.52765800	-0.29511200	2.29283300
H	0.11712100	-5.18725100	0.03978500	H	9.24979000	-0.08575400	-0.22145700
C	-1.97288600	-4.63838400	-0.05718600	Se	6.79518400	-0.02736400	-1.00986300
C	-2.86682500	-3.57176900	-0.08907400				

O-Me^{+}···I^{-} adduct geometry optimized using PBE0

BSSE corrected $E_{PBE0\text{-}D3}$ = -2537.315883 Hartrees BSSE Energy = 0.000051565460 Hartrees

Ru	-0.87271600	-0.03253500	0.12994200
N	-0.21438200	-0.07881500	-1.85816500
N	-0.79996600	-0.05819800	2.22321100
N	-1.15802500	2.01918700	0.08329900
N	-2.91654100	0.15278000	-0.17200500
N	-1.51726900	-1.99956000	0.04599800
C	1.86588600	-0.29467500	-0.72117400
C	1.00852200	-0.21404800	0.40255700
C	1.51660000	-0.27818700	1.71791200
C	2.88462900	-0.42248800	1.92534000
H	3.30288300	-0.47974800	2.92936200
C	3.72899200	-0.49948100	0.79799800
C	3.22706200	-0.43717600	-0.52550900
H	3.94165600	-0.50510800	-1.34808500
C	1.14052600	-0.21350600	-1.99817600
C	-0.98166600	0.00297900	-2.95177900
H	-2.05398300	0.10996700	-2.78202200
C	-0.45561600	-0.04447300	-4.23788300
H	-1.12544800	0.02537400	-5.09508100
C	0.92009200	-0.18123200	-4.39309000
H	1.36685600	-0.22181800	-5.38788400
C	1.72460800	-0.26573600	-3.26032600
H	2.80608700	-0.37258500	-3.34833300
C	0.46278600	-0.18230700	2.73884600
C	-1.84185100	0.03520500	3.05759700
H	-2.82466500	0.13282000	2.59506200
C	-1.69708500	0.01265300	4.44046500
H	-2.58037800	0.09201900	5.07431900
C	-0.41964300	-0.11029500	4.97649400
H	-0.26782000	-0.12956300	6.05697700
C	0.66957600	-0.20820900	4.11535100
H	1.68222800	-0.30319400	4.50753600
C	-0.17920400	2.92836100	0.22517500
H	0.82762000	2.53806600	0.36649300
C	-0.42518700	4.29201000	0.19571900
H	0.39485700	4.99964300	0.31322400
C	-1.73157600	4.73833500	0.01507300
C	-2.75118700	3.80235400	-0.13440200
H	-3.78183400	4.12249500	-0.27652500
C	-2.44110200	2.44746000	-0.10037000
C	-3.44612700	1.38398000	-0.25208000
C	-4.81219400	1.54358000	-0.45783200
H	-5.27604400	2.52618400	-0.53336000
C	-5.60282000	0.39859400	-0.56656700
C	-5.03297300	-0.87263600	-0.47512100
H	-5.65214000	-1.76396800	-0.55559300
C	-3.65677200	-0.96134300	-0.27826300
C	-2.85437600	-2.18970400	-0.16190600
C	-3.38935900	-3.46783700	-0.25487400
H	-4.45440900	-3.61871600	-0.42819000
C	-2.55309200	-4.57254300	-0.12583400
C	-1.19255200	-4.37011500	0.09168800
H	-0.49900800	-5.20265400	0.19922100
C	-0.71561200	-3.06923700	0.16920700
H	0.34151100	-2.86538300	0.33408800
C	-1.98854600	6.20886200	-0.01279600
O	-1.11577800	7.03724400	0.10232800
O	-3.27921500	6.49649900	-0.17731700
C	-7.06833700	0.59067000	-0.78158100
O	-7.59088500	1.67821700	-0.86279900
O	-7.72630800	-0.56424900	-0.87208300
C	-3.16275900	-5.93187400	-0.22655800
O	-4.34102200	-6.11964200	-0.42383600
O	-2.26380000	-6.90209800	-0.07322900
O	5.05902900	-0.63774300	0.87910800
C	5.67098300	-0.69726800	2.16682500
H	6.74622600	-0.79348300	1.97460200
H	5.47990700	0.22467100	2.73720400
H	5.31441900	-1.57171600	2.73298000
H	-3.36579000	7.46901800	-0.18680100
H	-8.67085700	-0.35476000	-1.00476500
H	-2.73419700	-7.75455300	-0.15061600
I	8.81632400	0.06737500	-0.67916500

S-Me^{+}···I^{-} adduct geometry optimized using PBE0

BSSE corrected $E_{PBE0\text{-}D3}$ = -2860.227446 Hartrees   BSSE Energy = 0.000047726132 Hartrees

Ru	-1.09124800	-0.01175700	-0.00905100
N	-0.78840700	-0.01625800	2.06144200
N	-0.65268400	-0.01208700	-2.05853200
N	-1.58710100	-2.02513500	-0.02464400
N	-3.16355200	0.01289700	-0.05159900
N	-1.53865100	2.01250300	-0.02106900
C	1.46855200	-0.02644400	1.30559700
C	0.81633100	-0.02394400	0.05207100
C	1.55023700	-0.02317300	-1.15816000
C	2.93330500	-0.02666500	-1.11215100
H	3.53553700	-0.02606200	-2.02414200
C	3.58584500	-0.03033600	0.14829100
C	2.85692200	-0.03015200	1.35818000
H	3.38036100	-0.03225900	2.31347700
C	0.52848500	-0.02302200	2.43638800
C	-1.73891300	-0.01224700	3.00354100
H	-2.77089600	-0.00722500	2.65017500
C	-1.44337900	-0.01468000	4.36230900
H	-2.25625100	-0.01128100	5.08873500
C	-0.10944100	-0.02150000	4.75627900
H	0.15883700	-0.02362500	5.81403300
C	0.88486300	-0.02575600	3.78212400
H	1.93835100	-0.03123500	4.06251300
C	0.68546600	-0.01725600	-2.34746800
C	-1.53861500	-0.00697600	-3.06214100
H	-2.59138200	-0.00365500	-2.77759400
C	-1.15414600	-0.00627400	-4.39815300
H	-1.91735000	-0.00204400	-5.17664800
C	0.20301300	-0.01112200	-4.70391000
H	0.54023100	-0.01072700	-5.74170700
C	1.13062800	-0.01677800	-3.66627900
H	2.20099700	-0.02109600	-3.87387500
C	-0.69882200	-3.03306000	-0.01537700
H	0.35298300	-2.75147000	0.00081600
C	-1.08738900	-4.36359500	-0.02637500
H	-0.33709600	-5.15365300	-0.01829500
C	-2.44535300	-4.67058800	-0.04782000
C	-3.37261900	-3.63220500	-0.05774100
H	-4.44126500	-3.83993500	-0.07472300
C	-2.92040200	-2.31772200	-0.04609300
C	-3.82234500	-1.15665400	-0.05620300
C	-5.21260800	-1.17509500	-0.06631400
H	-5.77872000	-2.10560600	-0.07018000
C	-5.89017200	0.04524500	-0.07002500
C	-5.18613700	1.25089500	-0.06388500
H	-5.71281600	2.20322600	-0.06582600
C	-3.79480400	1.19633900	-0.05421000
C	-2.86601900	2.33613100	-0.04270000
C	-3.28327200	3.66022600	-0.05326400
H	-4.34356800	3.91077100	-0.07102800
C	-2.33382000	4.67750100	-0.04173900
C	-0.98258000	4.34076400	-0.01954600
H	-0.20312500	5.10116100	-0.00992500
C	-0.62803300	2.99893400	-0.00984000
H	0.41708600	2.69328900	0.00697600
C	-2.85704400	-6.10556000	-0.06043800
O	-2.07030500	-7.02327200	-0.04935300
O	-4.18119300	-6.25234000	-0.08559100
C	-7.38302700	0.00671000	-0.07836400
O	-8.02234700	-1.01984100	-0.08430400
O	-7.92396600	1.22429800	-0.07792100
C	-2.81676600	6.09005600	-0.05583100
O	-3.98786100	6.39095900	-0.07900900
O	-1.81622900	6.96864500	-0.04089800
C	5.85320600	-0.03744600	1.80912600
H	6.95151200	-0.03952200	1.77057700
H	5.50688600	0.86775800	2.32658000
H	5.50326500	-0.94285100	2.32376600
S	5.33619500	-0.03376400	0.08033200
H	-2.21012200	7.86244600	-0.05134300
H	-8.89405000	1.11277400	-0.08376200
H	-4.37207400	-7.21001700	-0.09233600
I	9.48150000	-0.02566000	-0.25123000

Se-Me^{+}···I^{-} adduct geometry optimized using PBE0

BSSE corrected $E_{PBE0-D3}$ = -4863.46632 Hartrees BSSE Energy = 0.000063576123 Hartrees

Ru	-1.32001800	-0.01301900	-0.01044100	C	-3.61110700	-3.62628600	-0.05385600
N	-1.00651800	-0.01862400	2.05855900	H	-4.68047900	-3.83031900	-0.06887100
N	-0.89107500	-0.01392800	-2.06179200	C	-3.15432500	-2.31332800	-0.04318800
N	-1.81980300	-2.02512900	-0.02420400	C	-4.05285500	-1.14947900	-0.05192000
N	-3.39032100	0.01815700	-0.04708900	C	-5.44316300	-1.16336200	-0.06173800
N	-1.75854100	2.01274700	-0.02004900	H	-6.01247200	-2.09193300	-0.06585700
C	1.24674500	-0.03238700	1.29261500	C	-6.11692300	0.05918500	-0.06518400
C	0.58711200	-0.02921100	0.04157600	C	-5.40900300	1.26265600	-0.05903400
C	1.31593600	-0.02890700	-1.17237400	H	-5.93269400	2.21664500	-0.06101500
C	2.69985300	-0.03378500	-1.13313800	C	-4.01783300	1.20380000	-0.04965600
H	3.29744900	-0.03329600	-2.04819000	C	-3.08515300	2.34057000	-0.03923300
C	3.35805900	-0.03818100	0.12349300	C	-3.49845700	3.66598800	-0.04852000
C	2.63517300	-0.03749400	1.33691300	H	-4.55804600	3.91970300	-0.06436800
H	3.16398600	-0.04021500	2.28989500	C	-2.54610500	4.68055500	-0.03810100
C	0.31208900	-0.02757300	2.42744700	C	-1.19577400	4.33972200	-0.01827100
C	-1.95245600	-0.01319000	3.00559100	H	-0.41407700	5.09782300	-0.00955100
H	-2.98611800	-0.00665000	2.65723500	C	-0.84529600	2.99684800	-0.00982000
C	-1.65063800	-0.01617800	4.36281200	H	0.19894800	2.68803200	0.00520200
H	-2.46007400	-0.01154500	5.09307900	C	-3.10476600	-6.10130000	-0.05710000
C	-0.31469300	-0.02513900	4.75056800	O	-2.32180000	-7.02232200	-0.04787200
H	-0.04147500	-0.02775100	5.80707100	O	-4.42962200	-6.24304000	-0.07907400
C	0.67473500	-0.03097700	3.77173400	C	-7.60969700	0.02517600	-0.07383000
H	1.72965500	-0.03820200	4.04673500	O	-8.25229000	-0.99940500	-0.07999000
C	0.44569100	-0.02123500	-2.35711200	O	-8.14732100	1.24434300	-0.07357900
C	-1.78170500	-0.00708200	-3.06149400	C	-3.02492000	6.09438700	-0.05055600
H	-2.83316200	-0.00230400	-2.77218200	O	-4.19518000	6.39891700	-0.07102900
C	-1.40380800	-0.00657300	-4.39924200	O	-2.02185500	6.97026500	-0.03691900
H	-2.17074700	-0.00087300	-5.17405800	C	5.69962400	-0.04225300	1.93708100
C	-0.04796500	-0.01351000	-4.71153000	H	6.79818900	-0.04327500	1.95396800
H	0.28428700	-0.01332100	-5.75094600	H	5.31746400	0.86558800	2.42226000
C	0.88436900	-0.02105300	-3.67840500	H	5.31562100	-0.94870000	2.42339800
H	1.95373500	-0.02710400	-3.89099900	Se	5.24525600	-0.04281000	0.05462500
C	-0.93539000	-3.03653700	-0.01630700	H	-2.41341800	7.86508000	-0.04600700
H	0.11741300	-2.75850200	-0.00190800	H	-9.11765800	1.13522400	-0.07978000
C	-1.32849200	-4.36573500	-0.02645100	H	-4.62394300	-7.20002100	-0.08533700
H	-0.58098700	-5.15845100	-0.01955600	I	9.26701300	-0.01981900	-0.27468100
C	-2.68763300	-4.66804100	-0.04547700				

S-Ar^{+}···I^{-} adduct geometry optimized using PBE0

BSSE corrected $E_{PBE0\text{-}D3}$ = -2974.450217 Hartrees BSSE Energy = 0.000069556756 Hartrees

Ru	-1.37766600	-0.00701600	0.20907600
N	-1.57212900	0.01812000	2.29391100
N	-0.46751600	-0.04293300	-1.67856600
N	-1.85569200	-2.02092600	0.10710400
N	-3.37276300	0.00902400	-0.36212100
N	-1.81429900	2.01516700	0.07057900
C	0.79973500	0.00522700	2.09345000
C	0.46018900	-0.01284400	0.72074200
C	1.45778000	-0.03035800	-0.28000800
C	2.79154600	-0.02926900	0.09466500
H	3.57588500	-0.04316400	-0.66546200
C	3.14610600	-0.01046200	1.46870500
C	2.13369700	0.00660100	2.46511600
H	2.41792700	0.02114500	3.51764700
C	-0.38142600	0.02096700	2.96947700
C	-2.71839700	0.03003600	2.98514200
H	-3.63666500	0.02654100	2.39652400
C	-2.75202500	0.04614300	4.37488800
H	-3.71308600	0.05553000	4.88915300
C	-1.54854100	0.04937000	5.07276500
H	-1.53763400	0.06157300	6.16397400
C	-0.35253800	0.03645200	4.36092000
H	0.60596400	0.03787200	4.87991700
C	0.90094900	-0.05027000	-1.64063200
C	-1.09023800	-0.06536500	-2.86300700
H	-2.18055600	-0.05873300	-2.83604700
C	-0.39930900	-0.09496300	-4.06937700
H	-0.95592000	-0.11243200	-5.00644700
C	0.99148300	-0.10078600	-4.04373000
H	1.56592500	-0.12302200	-4.97124300
C	1.64639300	-0.07801000	-2.81561700
H	2.73529900	-0.08278200	-2.76282400
C	-1.00153000	-3.02172900	0.37872100
H	0.00836400	-2.73283200	0.66566300
C	-1.37401100	-4.35460800	0.30293100
H	-0.65304300	-5.13870400	0.53157700
C	-2.67861700	-4.67041300	-0.06643200
C	-3.56891500	-3.63889100	-0.35192800
H	-4.59474400	-3.85611500	-0.64423400
C	-3.13564900	-2.32101900	-0.26019200
C	-4.00115700	-1.16422400	-0.53903300
C	-5.33149100	-1.18905700	-0.94456800
H	-5.87194600	-2.12214100	-1.10048900
C	-5.98170700	0.02778900	-1.15473400
C	-5.31030300	1.23682400	-0.96370900
H	-5.81689700	2.18596600	-1.13056800
C	-3.97915400	1.18978000	-0.55612700
C	-3.09205100	2.33390500	-0.29345900
C	-3.49988100	3.65737600	-0.39258700
H	-4.52136300	3.90752500	-0.67674100
C	-2.59232100	4.67702700	-0.12245000
C	-1.28974400	4.34443200	0.24079100
H	-0.54325000	5.10619500	0.46001500
C	-0.94258700	3.00348700	0.32681700
H	0.06404500	2.70016000	0.61086900
C	-3.07610500	-6.10783000	-0.13903900
O	-2.32035600	-7.01947100	0.10379700
O	-4.34979800	-6.26456400	-0.49757400
H	-4.53343900	-7.22341100	-0.52171600
C	-7.41203800	-0.01787700	-1.58287900
O	-8.02354500	-1.04729400	-1.75236700
O	-7.93090900	1.19736000	-1.75181700
H	-8.86147700	1.08538400	-2.02584200
C	-3.06941000	6.08783300	-0.22998900
O	-4.20301100	6.38418500	-0.52927500
O	-2.11019400	6.97025300	0.04250600
H	-2.49869300	7.86242100	-0.04204800
C	4.54258700	-0.00844200	1.86763400
C	5.07384800	0.00316100	3.14834200
S	5.83346700	-0.02092700	0.68702300
C	6.48737700	0.00176300	3.17135000
H	4.46164600	0.01183300	4.04977600
C	7.03805600	-0.01059500	1.91293200
H	7.08316200	0.00944500	4.08429100
H	8.08925800	-0.01428100	1.62652600
I	9.06280700	-0.02302500	-1.65090300

Se-Ar⁺···I⁻ adduct geometry optimized using PBE0

BSSE corrected $E_{PBE0-D3}$ = -4977.684192 Hartrees BSSE Energy = 0.000076745670 Hartrees

Ru	-1.64908500	-0.02192400	0.19091900	H	-5.02841400	-3.70054700	-0.76733400
N	-1.79325300	-0.11171700	2.27788100	C	-3.49929100	-2.24353100	-0.35515500
N	-0.78508100	0.02845800	-1.71780400	C	-4.32642000	-1.04365400	-0.55671000
N	-2.19995100	-2.00960500	-0.00778500	C	-5.66246100	-1.00031000	-0.94052000
N	-3.65270400	0.09550000	-0.33199100	H	-6.23791400	-1.90410100	-1.13764800
N	-2.01295700	2.01767400	0.17086700	C	-6.27123600	0.24820600	-1.07643100
C	0.56986300	-0.22035000	2.01599600	C	-5.55335200	1.42123100	-0.83511700
C	0.19654100	-0.13570800	0.65389900	H	-6.02839900	2.39453100	-0.94345700
C	1.17059600	-0.12724500	-0.37108900	C	-4.21755400	1.30606500	-0.45706500
C	2.51130600	-0.19992100	-0.03225800	C	-3.28192800	2.40240400	-0.15970100
H	3.27883600	-0.19857300	-0.80959000	C	-3.63576500	3.74455800	-0.20001300
C	2.90130600	-0.28306500	1.33074100	H	-4.64874800	4.04863500	-0.46145800
C	1.91098600	-0.29288100	2.35135900	C	-2.68361600	4.71433400	0.09910100
H	2.22127500	-0.34534200	3.39522200	C	-1.39200000	4.31423600	0.43287300
C	-0.58873400	-0.20828400	2.92162600	H	-0.61238900	5.03509200	0.67398900
C	-2.92133500	-0.08624500	2.99817500	C	-1.09863100	2.95804700	0.45694300
H	-3.85178500	-0.00719700	2.43454100	H	-0.10144800	2.60230800	0.71247600
C	-2.92288300	-0.15710600	4.38656200	C	-3.59017100	-6.03101700	-0.40603500
H	-3.87038300	-0.13282600	4.92496500	O	-2.86751100	-6.98215700	-0.22048600
C	-1.70539800	-0.25915600	5.05201300	O	-4.87656300	-6.12105200	-0.74246600
H	-1.66919700	-0.31871100	6.14108200	H	-5.09909800	-7.07018600	-0.80135000
C	-0.52805400	-0.28448300	4.31028500	C	-7.70749900	0.27390400	-1.48584900
H	0.44054800	-0.36407100	4.80385000	O	-8.35412500	-0.72421400	-1.70528100
C	0.58242200	-0.03563500	-1.71527700	O	-8.18901800	1.51249400	-1.57905900
C	-1.43421100	0.11807000	-2.88490000	H	-9.12573100	1.44477800	-1.84683900
H	-2.52236300	0.16651400	-2.83030800	C	-3.10069900	6.14727600	0.05077600
C	-0.77246400	0.14902000	-4.10736400	O	-4.22050400	6.50442300	-0.23396400
H	-1.35007200	0.22304300	-5.02885700	O	-2.10411500	6.97559500	0.35692300
C	0.61701400	0.08285100	-4.11769000	H	-2.45279100	7.88663900	0.30792200
H	1.16905000	0.10355200	-5.05875300	C	4.30461700	-0.35460000	1.69240200
C	1.29934000	-0.01065700	-2.90817700	C	4.84256600	-0.58197900	2.94836500
H	2.38791000	-0.06497000	-2.88383100	C	6.25768800	-0.59609400	3.01304800
C	-1.38059600	-3.05487500	0.19555300	H	4.22246800	-0.75249000	3.82962800
H	-0.35315200	-2.81972900	0.46906600	C	6.88930300	-0.37719500	1.81524200
C	-1.80788500	-4.36738300	0.06918800	H	6.80522500	-0.76942100	3.94108900
H	-1.11288200	-5.18803600	0.24380500	H	7.96128400	-0.34119800	1.61997000
C	-3.13273800	-4.61543400	-0.27976800	Se	5.68994800	-0.12638600	0.42826800
C	-3.98710600	-3.53776900	-0.49517200	I	9.08628000	0.17391500	-1.58664900

O-Me geometry optimized using B3LYP

E_{B3LYP} = -2243.38137734 Hartrees

Ru	0.35506	-0.019912	0.036871	C	-1.141088	-4.681509	-0.025829
N	0.727404	-0.031722	2.135132	C	-2.021004	-3.598027	-0.047781
N	0.898605	-0.044628	-2.020854	H	-3.09588	-3.762886	-0.083133
N	-0.162042	-2.051668	0.018042	C	-1.517199	-2.295245	-0.025928
N	-1.675783	0.060221	-0.035942	C	-2.381706	-1.100305	-0.051991
N	-0.00102	2.046657	0.014269	C	-3.774648	-1.072904	-0.089849
C	2.992753	-0.123262	1.367561	H	-4.367069	-1.986467	-0.102334
C	2.33505	-0.098266	0.116959	C	-4.427672	0.168594	-0.111212
C	3.093125	-0.129194	-1.068774	C	-3.681244	1.356603	-0.094657
C	4.501619	-0.184733	-1.021049	H	-4.182172	2.322278	-0.110751
H	5.089321	-0.207164	-1.938606	C	-2.288809	1.27113	-0.056601
C	5.138966	-0.209146	0.230771	C	-1.334086	2.395301	-0.032701
C	4.391267	-0.179174	1.425646	C	-1.731174	3.732804	-0.056732
H	4.939389	-0.200844	2.370395	H	-2.787354	3.996528	-0.09489
C	2.056954	-0.084575	2.500455	C	-0.770767	4.744967	-0.032075
C	-0.211679	0.008425	3.103055	C	0.581978	4.385626	0.016423
H	-1.247712	0.049809	2.765474	H	1.37065	5.136098	0.037323
C	0.095544	-0.001063	4.459893	C	0.917092	3.036799	0.037607
H	-0.709413	0.033385	5.194964	H	1.958478	2.71962	0.07502
C	1.439456	-0.054914	4.84205	C	-1.616853	-6.097705	-0.048695
H	1.719199	-0.06412	5.897334	O	-0.877454	-7.060816	-0.037512
C	2.419813	-0.096935	3.854968	O	-2.957927	-6.19251	-0.083018
H	3.475539	-0.138977	4.124716	C	-5.918613	0.172315	-0.150268
C	2.255001	-0.09814	-2.27392	O	-6.601356	-0.833283	-0.167385
C	0.042383	-0.013702	-3.063049	O	-6.436836	1.414446	-0.164154
H	-1.017892	0.02755	-2.811012	C	-1.233925	6.165347	-0.060584
C	0.459972	-0.032663	-4.389812	O	-2.401711	6.496357	-0.107385
H	-0.281434	-0.005527	-5.189203	O	-0.214902	7.041064	-0.029456
C	1.831441	-0.0867	-4.659244	O	6.496887	-0.262475	0.40261
H	2.197088	-0.103443	-5.687862	C	7.322246	-0.296021	-0.756942
C	2.727342	-0.119641	-3.594996	H	8.35678	-0.335111	-0.389682
H	3.801734	-0.163174	-3.776989	H	7.124598	-1.189705	-1.375051
C	0.676855	-3.111144	0.038667	H	7.193284	0.60799	-1.378192
H	1.739868	-2.875999	0.073155	H	-3.182712	-7.145493	-0.095885
C	0.234988	-4.427132	0.017683	H	-7.410665	1.319686	-0.191508
H	0.951988	-5.247313	0.035886	H	-0.60228	7.940242	-0.049909

S-Me geometry optimized using B3LYP

E_{B3LYP} = -2566.37534546 Hartrees

Ru	0.197016	-0.010668	0.044434	C	-1.198258	-4.704407	-0.037918
N	0.558284	-0.018552	2.144282	C	-2.101353	-3.640093	-0.061318
N	0.744656	-0.018411	-2.012096	H	-3.172463	-3.827145	-0.103049
N	-0.276327	-2.05429	0.019257	C	-1.625411	-2.327089	-0.032373
N	-1.836734	0.024176	-0.037491	C	-2.515916	-1.151443	-0.05902
N	-0.206635	2.047683	0.021989	C	-3.908993	-1.15569	-0.101948
C	2.827466	-0.05411	1.385196	H	-4.47993	-2.082752	-0.118533
C	2.173709	-0.042818	0.13264	C	-4.589426	0.070823	-0.12258
C	2.936746	-0.052896	-1.052555	C	-3.870075	1.275289	-0.101778
C	4.343012	-0.074293	-0.995987	H	-4.393335	2.229025	-0.118325
H	4.931385	-0.081392	-1.913605	C	-2.476167	1.220774	-0.059108
C	4.983067	-0.085727	0.255559	C	-1.54685	2.365955	-0.031743
C	4.227847	-0.075806	1.447416	C	-1.974176	3.69385	-0.061078
H	4.748775	-0.085155	2.408491	H	-3.035768	3.933327	-0.105354
C	1.886936	-0.040396	2.515362	C	-1.037078	4.727591	-0.03466
C	-0.386064	-0.004168	3.10779	C	0.323048	4.399206	0.022232
H	-1.421197	0.013044	2.765465	H	1.094283	5.167519	0.045729
C	-0.084796	-0.010365	4.465923	C	0.688635	3.058301	0.048332
H	-0.893656	0.00207	5.197402	H	1.736844	2.765249	0.091739
C	1.258228	-0.032563	4.853999	C	-1.643855	-6.130378	-0.069407
H	1.533369	-0.038134	5.910501	O	-0.885183	-7.078208	-0.05091
C	2.243917	-0.04766	3.871388	O	-2.982059	-6.251766	-0.121354
H	3.29882	-0.06499	4.147014	C	-6.080246	0.042346	-0.165361
C	2.101999	-0.038912	-2.261554	O	-6.741356	-0.977405	-0.186789
C	-0.109221	-0.005073	-3.056607	O	-6.624123	1.273313	-0.177425
H	-1.170845	0.01056	-2.807627	C	-1.531888	6.137172	-0.071574
C	0.312994	-0.010637	-4.382046	O	-2.706444	6.441743	-0.126501
H	-0.426376	0.000905	-5.183703	O	-0.53283	7.035368	-0.038722
C	1.68596	-0.031087	-4.647556	C	7.407056	-0.118042	-1.247616
H	2.054945	-0.03609	-5.675076	H	8.502113	-0.134878	-1.146181
C	2.579302	-0.045361	-3.580552	H	7.083208	-1.014735	-1.796506
H	3.654938	-0.062001	-3.759348	H	7.110641	0.791363	-1.791094
C	0.585069	-3.095357	0.042145	S	6.768987	-0.11349	0.461174
H	1.64268	-2.837991	0.083406	H	-0.940058	7.925642	-0.065241
C	0.17157	-4.420585	0.015118	H	-7.595811	1.158844	-0.207415
H	0.906109	-5.225047	0.035328	H	-3.188612	-7.208723	-0.138444

Se-Me geometry optimized using B3LYP

E_{B3LYP} = -4569.72144903 Hartrees

Ru	-0.124458	-0.007512	0.028732	C	-1.486074	-4.711743	-0.029357
N	0.265283	-0.013059	2.123114	C	-2.397246	-3.654149	-0.044412
N	0.393085	-0.010319	-2.035729	H	-3.467461	-3.848946	-0.072377
N	-0.583019	-2.054796	0.010576	C	-1.930527	-2.337661	-0.024316
N	-2.159493	0.011945	-0.026958	C	-2.829962	-1.168693	-0.04173
N	-0.544199	2.047662	0.013821	C	-4.223332	-1.183545	-0.070305
C	2.523793	-0.029354	1.332622	H	-4.787183	-2.115008	-0.081635
C	1.853078	-0.023911	0.089118	C	-4.913158	0.037763	-0.083489
C	2.598934	-0.027517	-1.107904	C	-4.202776	1.247634	-0.069314
C	4.00595	-0.03686	-1.069885	H	-4.733581	2.197287	-0.079981
H	4.582987	-0.039569	-1.995249	C	-2.808155	1.203639	-0.040886
C	4.660397	-0.042166	0.172878	C	-1.88733	2.355799	-0.022299
C	3.926235	-0.038417	1.375266	C	-2.325227	3.680421	-0.042278
H	4.460604	-0.042292	2.328961	H	-3.389091	3.911705	-0.07217
C	1.599098	-0.023634	2.475507	C	-1.395796	4.721249	-0.025023
C	-0.665585	-0.007185	3.099797	C	-0.032577	4.403148	0.013315
H	-1.705485	0.001074	2.771879	H	0.732921	5.177369	0.028878
C	-0.345182	-0.011225	4.45353	C	0.343591	3.06506	0.031233
H	-1.143674	-0.006101	5.196402	H	1.394489	2.780032	0.060427
C	1.003394	-0.021879	4.822707	C	-1.921509	-6.141045	-0.051624
H	1.293386	-0.025399	5.875229	O	-1.155848	-7.083324	-0.03877
C	1.975244	-0.028115	3.826382	O	-3.259294	-6.272056	-0.088311
H	3.034125	-0.036569	4.086727	C	-6.404118	-0.001627	-0.111451
C	1.746668	-0.020039	-2.304777	O	-7.058155	-1.02605	-0.125917
C	-0.475845	-0.003155	-3.067851	O	-6.956704	1.225483	-0.118566
H	-1.533911	0.004172	-2.803755	C	-1.901372	6.127254	-0.051099
C	-0.07261	-0.004863	-4.399197	O	-3.078655	6.423597	-0.09037
H	-0.82352	0.001265	-5.190125	O	-0.908435	7.032461	-0.027563
C	1.296506	-0.014498	-4.684452	C	7.153059	-0.04796	-1.528316
H	1.650704	-0.016144	-5.717158	H	8.252044	-0.052558	-1.495392
C	2.205172	-0.022165	-3.630377	H	6.794232	-0.949456	-2.043801
H	3.278296	-0.03006	-3.824271	H	6.801489	0.861459	-2.03484
C	0.28624	-3.08946	0.02541	Se	6.594633	-0.054871	0.352618
H	1.342354	-2.824346	0.053337	H	-1.32225	7.919902	-0.046588
C	-0.117843	-4.417742	0.006501	H	-7.927846	1.104361	-0.138171
H	0.622817	-5.216708	0.019616	H	-3.45949	-7.230415	-0.100487

S-Ar geometry optimized using B3LYP

E_{B3LYP} = -2680.70190752 Hartrees

Ru	-0.200765	-0.017616	0.022384
N	0.200673	-0.048912	2.114205
N	0.298262	-0.010096	-2.047212
N	-0.698725	-2.055431	-0.011208
N	-2.237916	0.040065	-0.019646
N	-0.584502	2.04542	0.03154
C	2.452352	-0.104973	1.306612
C	1.771061	-0.073325	0.068568
C	2.509181	-0.077321	-1.135963
C	3.909521	-0.119069	-1.10164
H	4.483541	-0.144976	-2.031097
C	4.594898	-0.147339	0.134408
C	3.852312	-0.135237	1.338886
H	4.388466	-0.132372	2.289958
C	1.536292	-0.088476	2.456593
C	-0.723023	-0.031715	3.097441
H	-1.764851	-0.000873	2.777069
C	-0.39282	-0.051539	4.448701
H	-1.185545	-0.035942	5.197571
C	0.957913	-0.091557	4.807815
H	1.255355	-0.108388	5.858117
C	1.922305	-0.11034	3.804364
H	2.982761	-0.142382	4.056334
C	1.648659	-0.044011	-2.327084
C	-0.578362	0.023693	-3.072143
H	-1.634026	0.049816	-2.799898
C	-0.184974	0.025623	-4.406517
H	-0.941475	0.054077	-5.191599
C	1.181256	-0.009613	-4.702475
H	1.527554	-0.009908	-5.737823
C	2.097654	-0.044749	-3.655461
H	3.168945	-0.073679	-3.857463
C	0.151608	-3.105695	-0.009273
H	1.212555	-2.860288	0.013869
C	-0.276825	-4.426287	-0.035033
H	0.449199	-5.238677	-0.032264
C	-1.650282	-4.69487	-0.063687
C	-2.541873	-3.620652	-0.065817
H	-3.61543	-3.796089	-0.088127
C	-2.051273	-2.313123	-0.04011
C	-2.929468	-1.127707	-0.042969
C	-4.323121	-1.116711	-0.065619
H	-4.904661	-2.037092	-0.085027
C	-4.990205	0.117149	-0.061657
C	-4.257885	1.313528	-0.036856
H	-4.770485	2.273155	-0.033559
C	-2.863999	1.243316	-0.017103
C	-1.921812	2.378343	0.008209
C	-2.335246	3.710875	0.007582
H	-3.394885	3.962132	-0.011666
C	-1.386614	4.734155	0.032157
C	-0.029437	4.390768	0.05623
H	0.750474	5.150329	0.075941
C	0.322116	3.045761	0.054832
H	1.367783	2.741431	0.073086
C	-2.112104	-6.115905	-0.091887
O	-1.363346	-7.071676	-0.093665
O	-3.452311	-6.222787	-0.115345
H	-3.66914	-7.177537	-0.132581
C	-6.482056	0.104267	-0.083881
O	-7.15308	-0.90882	-0.10722
O	-7.013603	1.340436	-0.075139
H	-7.98674	1.235946	-0.092034
C	-1.866885	6.149418	0.030928
O	-3.039143	6.466669	0.011225
O	-0.857662	7.036374	0.053702
H	-1.255492	7.931279	0.051445
C	6.066266	-0.197108	0.188867
C	6.878565	-0.69187	1.189925
S	7.059518	0.446211	-1.119061
C	8.274945	-0.567964	0.915549
H	6.483695	-1.155897	2.094406
C	8.532939	0.022376	-0.294138
H	9.057944	-0.915033	1.591528
H	9.494986	0.235095	-0.758425

Se-Ar geometry optimized using B3LYP

E_{B3LYP} = -4684.04293417 Hartrees

Ru	-0.522484	-0.025546	0.057416	H	-3.988233	-3.752103	-0.177483
N	-0.188467	-0.067336	2.160982	C	-2.405375	-2.291644	-0.071366
N	0.042713	-0.022146	-1.994974	C	-3.265072	-1.092677	-0.094416
N	-1.050561	-2.054438	-0.000166	C	-4.657256	-1.059254	-0.156552
N	-2.556302	0.064189	-0.047405	H	-5.252951	-1.96994	-0.194467
N	-0.873533	2.043105	0.060634	C	-5.304666	0.185089	-0.168486
C	2.086732	-0.162147	1.425551	C	-4.554567	1.369659	-0.119479
C	1.445727	-0.115499	0.166716	H	-5.051472	2.337483	-0.127857
C	2.221956	-0.13078	-1.013595	C	-3.163093	1.277274	-0.059537
C	3.619388	-0.198674	-0.935115	C	-2.20416	2.397209	-0.002318
H	4.224302	-0.230515	-1.844646	C	-2.59607	3.736188	-0.011057
C	4.266593	-0.24615	0.32138	H	-3.650544	4.004385	-0.061951
C	3.484355	-0.220744	1.501433	C	-1.632621	4.744176	0.046435
H	3.989444	-0.230698	2.469248	C	-0.28242	4.379217	0.110999
C	1.134751	-0.130471	2.545972	H	0.508571	5.126043	0.157319
C	-1.143164	-0.034458	3.11375	C	0.047656	3.028795	0.115412
H	-2.173365	0.015363	2.759874	H	1.087316	2.707755	0.164805
C	-0.857554	-0.060836	4.474963	C	-2.518124	-6.093319	-0.148781
H	-1.673818	-0.031595	5.197661	O	-1.781683	-7.058597	-0.138801
C	0.479946	-0.12481	4.877437	O	-3.858557	-6.183087	-0.205996
H	0.742869	-0.147368	5.936833	H	-4.08627	-7.135057	-0.235167
C	1.475953	-0.160073	3.905842	C	-6.795314	0.195408	-0.233307
H	2.526651	-0.210707	4.193062	O	-7.480605	-0.807281	-0.279728
C	1.400607	-0.079476	-2.231515	O	-7.308281	1.439475	-0.235053
C	-0.800148	0.027975	-3.04723	H	-8.281901	1.349811	-0.2821
H	-1.863279	0.072155	-2.808503	C	-2.090417	6.16686	0.03478
C	-0.364631	0.024455	-4.368407	O	-3.256277	6.502561	-0.022781
H	-1.095084	0.066814	-5.177155	O	-1.068779	7.037756	0.094303
C	1.009568	-0.034262	-4.620671	H	-1.452074	7.938856	0.08282
H	1.388602	-0.039643	-5.644501	C	5.733429	-0.33352	0.423199
C	1.891385	-0.086619	-3.545112	C	6.483913	-0.860796	1.451032
H	2.967839	-0.134535	-3.713175	C	7.90619	-0.797123	1.290388
C	-0.215893	-3.116961	0.019932	H	6.020847	-1.32303	2.32519
H	0.847281	-2.887078	0.076278	C	8.335245	-0.215327	0.129918
C	-0.662343	-4.430823	-0.027534	H	8.59699	-1.195101	2.037347
H	0.051328	-5.253857	-0.008539	H	9.360436	-0.068631	-0.208832
C	-2.038078	-4.678965	-0.098478	Se	6.891447	0.347487	-0.93178
C	-2.913579	-3.591825	-0.120667				

O-Me+ geometry optimized using B3LYP

E_{B3LYP} = -2243.21573210 Hartrees

Ru	0.409474	-0.019693	0.042175	C	-1.123505	-4.674589	-0.03491
N	0.722636	-0.034835	2.156503	C	-2.011817	-3.59743	-0.05251
N	0.889943	-0.037967	-2.035179	H	-3.085621	-3.767822	-0.086026
N	-0.165443	-2.048453	0.012948	C	-1.514745	-2.294095	-0.028264
N	-1.690776	0.05696	-0.032104	C	-2.383268	-1.10025	-0.050194
N	-0.018342	2.047608	0.015039	C	-3.778106	-1.082644	-0.08838
C	2.986469	-0.120558	1.383141	H	-4.365976	-1.998716	-0.102922
C	2.337221	-0.094871	0.118731	C	-4.430841	0.155909	-0.107328
C	3.085379	-0.1201	-1.084843	C	-3.692751	1.34575	-0.089863
C	4.479713	-0.169238	-1.039584	H	-4.195092	2.310265	-0.105589
H	5.073712	-0.186845	-1.952065	C	-2.29845	1.259632	-0.052051
C	5.114954	-0.192799	0.224535	C	-1.347612	2.389161	-0.029699
C	4.371893	-0.169662	1.435758	C	-1.746783	3.724192	-0.054341
H	4.930475	-0.190492	2.372947	H	-2.803063	3.986796	-0.091933
C	2.047509	-0.08787	2.522328	C	-0.784808	4.7355	-0.031032
C	-0.21983	0.000951	3.114366	C	0.565409	4.379926	0.016775
H	-1.25479	0.043077	2.774545	H	1.354687	5.129482	0.03688
C	0.08874	-0.014116	4.474062	C	0.904438	3.030195	0.037633
H	-0.716929	0.016817	5.208185	H	1.946018	2.716028	0.073822
C	1.428542	-0.069023	4.856715	C	-1.592424	-6.097385	-0.060102
H	1.70701	-0.082893	5.911935	O	-0.844016	-7.051701	-0.052091
C	2.414589	-0.106541	3.870048	O	-2.930823	-6.194969	-0.092409
H	3.469207	-0.149535	4.142455	C	-5.928834	0.15421	-0.14628
C	2.240552	-0.088406	-2.294085	O	-6.599279	-0.85687	-0.167024
C	0.026916	-0.007006	-3.064686	O	-6.445452	1.392901	-0.155207
H	-1.0319	0.031828	-2.807694	C	-1.250513	6.159232	-0.061835
C	0.443194	-0.02358	-4.395704	O	-2.418933	6.482036	-0.112955
H	-0.301389	0.003203	-5.191875	O	-0.232251	7.031801	-0.027961
C	1.809382	-0.074631	-4.670585	O	6.448591	-0.237191	0.39245
H	2.170845	-0.089418	-5.700276	C	7.305807	-0.260426	-0.758542
C	2.713862	-0.10765	-3.608455	H	8.327727	-0.288704	-0.36092
H	3.787154	-0.149501	-3.79519	H	7.121832	-1.157781	-1.370635
C	0.685491	-3.095387	0.029354	H	7.171048	0.645739	-1.370488
H	1.746909	-2.856398	0.061653	H	-3.157831	-7.147883	-0.107057
C	0.248234	-4.414767	0.006182	H	-7.420467	1.304	-0.183247
H	0.971366	-5.229396	0.021003	H	-0.614674	7.933567	-0.050219

S-Me⁺ geometry optimized using B3LYP

E_{B3LYP} = -2566.20768450 Hartrees

Ru	-0.249728	-0.009762	0.053567	C	1.180615	-4.697891	-0.043914
N	-0.732792	-0.01299	-2.022827	C	2.092202	-3.640226	-0.062859
N	-0.560642	-0.019224	2.167407	H	3.162222	-3.832754	-0.104014
N	0.279586	-2.051789	0.020142	C	1.622895	-2.326708	-0.029831
N	1.850212	0.020142	-0.028265	C	2.516845	-1.152024	-0.052548
N	0.222447	2.047074	0.026079	C	3.911416	-1.165477	-0.097434
C	-2.927996	-0.043129	-1.070119	H	4.477854	-2.094878	-0.116716
C	-2.178414	-0.037061	0.133579	C	4.591411	0.058263	-0.116702
C	-2.826682	-0.049359	1.397788	C	3.87995	1.264251	-0.094943
C	-4.214869	-0.068177	1.451643	H	4.40448	2.216874	-0.112557
H	-4.747961	-0.078592	2.404606	C	2.484374	1.209021	-0.050803
C	-4.962962	-0.073574	0.242482	C	1.558764	2.358968	-0.026078
C	-4.321658	-0.060887	-1.018867	C	1.987751	3.684428	-0.05866
H	-4.91289	-0.06461	-1.933239	H	3.049362	3.922921	-0.103119
C	-2.084283	-0.029545	-2.280698	C	1.048885	4.717159	-0.035545
C	0.129668	-0.000688	-3.053563	C	-0.308668	4.392069	0.022167
H	1.189388	0.01187	-2.797887	H	-1.080675	5.159408	0.04379
C	-0.288494	-0.003787	-4.383966	C	-0.677764	3.05036	0.050582
H	0.455582	0.006665	-5.181002	H	-1.725972	2.759963	0.093292
C	-1.655894	-0.020399	-4.657516	C	1.617848	-6.130446	-0.081856
H	-2.018746	-0.023425	-5.686813	O	0.849569	-7.068681	-0.060758
C	-2.55964	-0.033578	-3.594438	O	2.952746	-6.255833	-0.143473
H	-3.633854	-0.047521	-3.779958	C	6.08881	0.024707	-0.160932
C	-1.885772	-0.0397	2.535946	O	6.738217	-0.999932	-0.185735
C	0.384524	-0.008061	3.123393	O	6.630599	1.25261	-0.169048
H	1.419449	0.008324	2.781355	C	1.54587	6.129901	-0.078999
C	0.078593	-0.016615	4.483676	O	2.720661	6.426633	-0.141229
H	0.886342	-0.006822	5.216109	O	0.547304	7.024804	-0.043412
C	-1.261388	-0.037739	4.869129	C	-7.379685	-0.098456	-1.256784
H	-1.537895	-0.045189	5.924917	H	-8.471923	-0.112865	-1.13323
C	-2.250167	-0.049466	3.884602	H	-7.085276	0.81304	-1.79606
H	-3.304701	-0.065948	4.160276	H	-7.061907	-0.997925	-1.802914
C	-0.593496	-3.080237	0.040104	S	-6.717794	-0.096542	0.442679
H	-1.649353	-2.818801	0.080425	H	0.949486	7.917711	-0.074506
C	-0.184817	-4.408599	0.009764	H	7.603546	1.144144	-0.200604
H	-0.925212	-5.207513	0.027218	H	3.160283	-7.212951	-0.164486

Se-Me$^+$ geometry optimized using B3LYP

E_{B3LYP} = -4569.55389187 Hartrees

Ru	0.070392	-0.006022	0.036225
N	-0.385938	-0.006186	-2.046749
N	-0.268827	-0.010627	2.144955
N	0.581338	-2.052958	0.013099
N	2.16902	0.006728	-0.019158
N	0.557165	2.046935	0.017123
C	-2.593682	-0.018296	-1.123603
C	-1.85888	-0.016564	0.09064
C	-2.524639	-0.02134	1.346332
C	-3.914399	-0.028375	1.38207
H	-4.459729	-0.03202	2.328057
C	-4.644644	-0.030751	0.163962
C	-3.987725	-0.02534	-1.088992
H	-4.568533	-0.026693	-2.010598
C	-1.733926	-0.01236	-2.322585
C	0.490162	-0.000637	-3.066286
H	1.546529	0.003982	-2.796681
C	0.089779	-0.000766	-4.401998
H	0.844427	0.003901	-5.189116
C	-1.274158	-0.006988	-4.693702
H	-1.623275	-0.007339	-5.727751
C	-2.191828	-0.012923	-3.642757
H	-3.263677	-0.01818	-3.842017
C	-1.598861	-0.018113	2.496078
C	0.663604	-0.007216	3.113711
H	1.703049	-0.001354	2.785253
C	0.33967	-0.010962	4.469712
H	1.13764	-0.007976	5.212867
C	-1.005667	-0.018583	4.837544
H	-1.296118	-0.021794	5.889605
C	-1.981139	-0.022212	3.840053
H	-3.039567	-0.028274	4.100907
C	-0.299317	-3.075172	0.025882
H	-1.353554	-2.805615	0.053395
C	0.099523	-4.406633	0.004241
H	-0.647007	-5.199919	0.015322
C	1.463352	-4.706466	-0.032698
C	2.382981	-3.655666	-0.044287
H	3.452016	-3.856181	-0.072175
C	1.923117	-2.338482	-0.020827
C	2.82657	-1.170935	-0.035708
C	4.221347	-1.195644	-0.066085
H	4.780294	-2.129675	-0.079207
C	4.911501	0.022531	-0.079131
C	4.209553	1.234307	-0.06459
H	4.742349	2.182444	-0.076739
C	2.813265	1.190545	-0.03445
C	1.896766	2.347906	-0.018093
C	2.337481	3.669789	-0.041333
H	3.40155	3.899154	-0.071761
C	1.407362	4.710546	-0.027297
C	0.046378	4.396762	0.012283
H	-0.719167	5.170725	0.026066
C	-0.334322	3.058228	0.032795
H	-1.385409	2.776646	0.061724
C	1.889976	-6.142282	-0.060721
O	1.114518	-7.074745	-0.043722
O	3.224546	-6.278105	-0.107317
C	6.408758	-0.022801	-0.108703
O	7.050896	-1.052277	-0.125255
O	6.960066	1.201028	-0.114884
C	1.916312	6.119112	-0.060409
O	3.094033	6.406984	-0.108325
O	0.924617	7.022025	-0.033158
C	-7.124223	-0.040508	-1.528
H	-8.22179	-0.045603	-1.473683
H	-6.777064	0.869816	-2.034842
H	-6.768789	-0.945427	-2.038727
Se	-6.541539	-0.041893	0.342358
H	1.334448	7.911659	-0.057353
H	7.932396	1.085043	-0.136155
H	3.425054	-7.236791	-0.122347

S-Ar⁺ geometry optimized using B3LYP

E_{B3LYP} = -2680.53130215 Hartrees

Ru	-0.149752	-0.006756	0.035454	H	-3.535424	-3.856393	-0.067242
N	0.197616	-0.015313	2.143263	C	-2.00696	-2.33835	-0.019045
N	0.298735	-0.003028	-2.050589	C	-2.91005	-1.170709	-0.02962
N	-0.665476	-2.052708	0.011182	C	-4.304924	-1.195401	-0.056666
N	-2.252356	0.006503	-0.012495	H	-4.863806	-2.129443	-0.070596
N	-0.640767	2.045653	0.022867	C	-4.994851	0.022776	-0.065104
C	2.450069	-0.027158	1.33345	C	-4.292828	1.234325	-0.049437
C	1.77875	-0.018565	0.082543	H	-4.825277	2.182681	-0.057864
C	2.508781	-0.01746	-1.133901	C	-2.896442	1.190094	-0.023372
C	3.89866	-0.026564	-1.095783	C	-1.979957	2.347258	-0.007305
H	4.471398	-0.030727	-2.024718	C	-2.419794	3.669417	-0.025896
C	4.584163	-0.036393	0.151127	H	-3.483795	3.899542	-0.052225
C	3.838883	-0.035245	1.36596	C	-1.488703	4.709318	-0.012312
H	4.369983	-0.03515	2.317329	C	-0.127922	4.39458	0.021909
C	1.529263	-0.024364	2.487741	H	0.638325	5.167846	0.034896
C	-0.730163	-0.012679	3.116128	C	0.252031	3.055736	0.037996
H	-1.771151	-0.005723	2.792646	H	1.303013	2.773481	0.062728
C	-0.399246	-0.018566	4.470644	C	-1.972278	-6.14209	-0.064136
H	-1.193452	-0.016141	5.217803	O	-1.196152	-7.073961	-0.051647
C	0.94774	-0.027591	4.831771	O	-3.306819	-6.278143	-0.106821
H	1.243372	-0.032464	5.882354	H	-3.507372	-7.236824	-0.123197
C	1.918436	-0.03058	3.829364	C	-6.492479	-0.022389	-0.091222
H	2.978328	-0.03787	4.08419	O	-7.1343	-1.051933	-0.108411
C	1.645705	-0.009276	-2.330868	O	-7.04336	1.201442	-0.092625
C	-0.580882	0.004851	-3.066877	H	-8.015838	1.086046	-0.111489
H	-1.636322	0.009617	-2.79379	C	-1.996783	6.118558	-0.039939
C	-0.184659	0.006848	-4.403917	O	-3.174488	6.407002	-0.082617
H	-0.941816	0.013382	-5.188586	O	-1.004298	7.020413	-0.014227
C	1.178159	0.000208	-4.699986	H	-1.413182	7.910601	-0.03485
H	1.523977	0.00132	-5.735114	C	6.040569	-0.049192	0.20275
C	2.099517	-0.008048	-3.652034	C	6.866836	-0.138474	1.316148
H	3.170714	-0.013858	-3.854903	S	7.025926	0.05549	-1.259559
C	0.216122	-3.0741	0.019749	C	8.251369	-0.124668	1.005907
H	1.270355	-2.804384	0.044173	H	6.487982	-0.218355	2.334489
C	-0.182439	-4.405673	-0.002558	C	8.494631	-0.023549	-0.343804
H	0.564562	-5.198554	0.004845	H	9.042107	-0.188389	1.753858
C	-1.546154	-4.705903	-0.035444	H	9.453947	0.010482	-0.858863
C	-2.466382	-3.655581	-0.04258				

Se-Ar⁺ geometry optimized using B3LYP

E_{B3LYP} = -4683.87309036 Hartrees

Ru	-0.470859	-0.016258	0.072138	H	-3.910926	-3.809766	-0.15286
N	-0.206353	-0.02492	2.192874	C	-2.360888	-2.31658	-0.046716
N	0.058961	-0.027422	-1.993094	C	-3.244646	-1.134217	-0.08073
N	-1.016219	-2.053226	0.026658	C	-4.638722	-1.135283	-0.141485
N	-2.568242	0.032265	-0.048403	H	-5.213325	-2.059507	-0.167227
N	-0.923208	2.044792	0.032745	C	-5.307756	0.094466	-0.167095
C	2.07565	-0.061384	1.471654	C	-4.586042	1.294171	-0.13666
C	1.451985	-0.052468	0.195294	H	-5.101766	2.251474	-0.158627
C	2.231488	-0.059163	-0.991223	C	-3.191504	1.226721	-0.077651
C	3.618056	-0.074281	-0.899056	C	-2.255911	2.368394	-0.040613
H	4.226622	-0.080622	-1.804788	C	-2.672743	3.697528	-0.081476
C	4.258528	-0.082221	0.373445	H	-3.731462	3.945278	-0.14255
C	3.461347	-0.075929	1.557622	C	-1.725647	4.722264	-0.044134
H	3.951581	-0.075621	2.530343	C	-0.372008	4.385471	0.034292
C	1.110577	-0.048397	2.589639	H	0.406361	5.145975	0.067007
C	-1.171979	-0.011407	3.128298	C	-0.015044	3.040653	0.069616
H	-2.199359	0.007478	2.764303	H	1.029972	2.741953	0.128757
C	-0.895711	-0.020381	4.494823	C	-2.386349	-6.120108	-0.113442
H	-1.719073	-0.008735	5.209612	O	-1.625977	-7.064625	-0.085748
C	0.435693	-0.044444	4.909088	O	-3.721262	-6.234755	-0.193751
H	0.689325	-0.052445	5.970623	H	-3.935588	-7.190224	-0.221693
C	1.44535	-0.058641	3.946229	C	-6.804817	0.073683	-0.2264
H	2.493629	-0.077965	4.244601	O	-7.462347	-0.945504	-0.260625
C	1.415805	-0.047622	-2.221046	O	-7.336659	1.305954	-0.236353
C	-0.780688	-0.018124	-3.042944	H	-8.310038	1.205225	-0.278503
H	-1.845703	-0.002844	-2.810689	C	-2.210321	6.139031	-0.094337
C	-0.333576	-0.027193	-4.363617	O	-3.381912	6.445282	-0.169772
H	-1.059852	-0.018919	-5.176916	O	-1.204983	7.025875	-0.048081
C	1.03964	-0.046479	-4.606838	H	-1.599516	7.922004	-0.083466
H	1.425115	-0.053783	-5.627882	C	5.710064	-0.093805	0.48325
C	1.91962	-0.057015	-3.52419	C	6.474101	-0.16574	1.64022
H	2.997566	-0.073115	-3.686027	C	7.883753	-0.154845	1.458925
C	-0.152188	-3.089075	0.05688	H	6.028123	-0.232499	2.633052
H	0.905061	-2.836805	0.115561	C	8.29869	-0.072607	0.15241
C	-0.571299	-4.413803	0.014265	H	8.583261	-0.208513	2.295209
H	0.161973	-5.219025	0.040584	H	9.322451	-0.048456	-0.22034
C	-1.938048	-4.691406	-0.062789	Se	6.862616	0.000715	-1.037875
C	-2.840294	-3.626062	-0.092718				

O-Me geometry optimized using M06

E_{M06} = -2241.90220251 Hartrees

Ru	0.35666	-0.01059	0.029207	C	-1.020322	-4.677884	-0.042073
N	0.695312	-0.015206	2.112843	C	-1.926651	-3.623348	-0.062761
N	0.910041	-0.022959	-2.005274	H	-3.000025	-3.813951	-0.09844
N	-0.112957	-2.03968	0.006117	C	-1.456454	-2.314254	-0.038874
N	-1.672975	0.025512	-0.050783	C	-2.347146	-1.146309	-0.062844
N	-0.039249	2.034807	0.004064	C	-3.735592	-1.149125	-0.091744
C	2.96115	-0.060702	1.384222	H	-4.312456	-2.075652	-0.101067
C	2.321975	-0.047948	0.128809	C	-4.409075	0.075725	-0.106877
C	3.088498	-0.064431	-1.046236	C	-3.693413	1.276812	-0.093528
C	4.491486	-0.093483	-0.982257	H	-4.21826	2.232699	-0.104156
H	5.089389	-0.105622	-1.897021	C	-2.304475	1.219582	-0.064924
C	5.11197	-0.105914	0.272421	C	-1.373452	2.355418	-0.04242
C	4.353596	-0.090074	1.455132	C	-1.794267	3.679556	-0.067954
H	4.895744	-0.101846	2.406219	H	-2.857234	3.925758	-0.105824
C	2.012893	-0.04109	2.498361	C	-0.853949	4.703147	-0.046399
C	-0.261257	0.005428	3.056826	C	0.50092	4.373103	0.00105
H	-1.294692	0.026096	2.696971	H	1.2741	5.141511	0.019559
C	0.020511	0.001506	4.413709	C	0.860252	3.03536	0.024561
H	-0.797072	0.019029	5.135939	H	1.910054	2.733814	0.061741
C	1.354621	-0.024995	4.816821	C	-1.4545	-6.100409	-0.067716
H	1.614743	-0.028902	5.877684	O	-0.691133	-7.037741	-0.049501
C	2.351728	-0.046376	3.852885	O	-2.782919	-6.225922	-0.113806
H	3.405396	-0.066836	4.139786	C	-5.893669	0.04687	-0.134238
C	2.261847	-0.049607	-2.251192	O	-6.550329	-0.969606	-0.148046
C	0.055999	-0.008365	-3.043172	O	-6.430431	1.27055	-0.141302
H	-1.008636	0.012348	-2.789748	C	-1.342921	6.107675	-0.078297
C	0.476025	-0.018652	-4.363532	O	-2.512399	6.412021	-0.125557
H	-0.262144	-0.005832	-5.166837	O	-0.345727	6.993959	-0.050164
C	1.845379	-0.045478	-4.626018	O	6.459896	-0.133817	0.456088
H	2.213619	-0.054454	-5.654373	C	7.278981	-0.151579	-0.690441
C	2.737676	-0.061101	-3.564809	H	8.317136	-0.172807	-0.333556
H	3.815266	-0.082807	-3.742195	H	7.0987	-1.04709	-1.312361
C	0.752684	-3.070878	0.025436	H	7.135853	0.749702	-1.313723
H	1.812249	-2.805407	0.060854	H	-2.984792	-7.181787	-0.1272
C	0.344411	-4.392814	0.002518	H	-7.400465	1.157839	-0.160578
H	1.078899	-5.199152	0.019619	H	-0.748077	7.884229	-0.073532

S-Me geometry optimized using M06

E_{M06} = -2564.89743929 Hartrees

Ru	0.199698	-0.0074	0.034517	C	-1.142534	-4.685603	-0.045474
N	0.532228	-0.011093	2.118476	C	-2.056717	-3.6379	-0.068582
N	0.750599	-0.013133	-2.000623	H	-3.128542	-3.836235	-0.108844
N	-0.25521	-2.040823	0.009012	C	-1.596202	-2.325484	-0.040999
N	-1.832264	0.012474	-0.05061	C	-2.496124	-1.164516	-0.066405
N	-0.214586	2.034969	0.009251	C	-3.884459	-1.179115	-0.100137
C	2.798662	-0.033509	1.392801	H	-4.453376	-2.110476	-0.1124
C	2.160621	-0.026837	0.136825	C	-4.567779	0.040127	-0.116396
C	2.928296	-0.03421	-1.040307	C	-3.862302	1.247077	-0.100025
C	4.328283	-0.048026	-0.971428	H	-4.395289	2.198419	-0.112176
H	4.925258	-0.053127	-1.887458	C	-2.472935	1.201071	-0.066662
C	4.953731	-0.054684	0.281801	C	-1.551089	2.344538	-0.041563
C	4.19242	-0.047731	1.46411	C	-1.982754	3.66502	-0.069754
H	4.709241	-0.053758	2.430776	H	-3.047541	3.902342	-0.11142
C	1.84909	-0.024378	2.506454	C	-1.05089	4.696271	-0.046163
C	-0.426833	-0.001575	3.060117	C	0.306319	4.377491	0.006306
H	-1.459511	0.008999	2.697739	H	1.073073	5.152254	0.026799
C	-0.147962	-0.004616	4.417525	C	0.676644	3.042682	0.032131
H	-0.967152	0.003491	5.138087	H	1.728857	2.750205	0.072972
C	1.185491	-0.018114	4.823323	C	-1.565401	-6.11163	-0.075321
H	1.44342	-0.020919	5.884709	O	-0.794613	-7.042667	-0.051454
C	2.185013	-0.028027	3.861693	O	-2.892252	-6.24743	-0.132529
H	3.237802	-0.038503	4.152265	C	-6.05228	-0.000557	-0.148484
C	2.101911	-0.026301	-2.246722	O	-6.700673	-1.022168	-0.16532
C	-0.103743	-0.005574	-3.038329	O	-6.598155	1.21893	-0.156024
H	-1.168488	0.004575	-2.784982	C	-1.551038	6.096931	-0.081921
C	0.316835	-0.010227	-4.358587	O	-2.722662	6.391752	-0.134734
H	-0.421244	-0.003645	-5.162045	O	-0.561006	6.990948	-0.050503
C	1.686208	-0.023384	-4.621025	C	7.348796	-0.080956	-1.19723
H	2.054599	-0.02747	-5.649322	H	8.444807	-0.092006	-1.110383
C	2.578619	-0.031516	-3.559625	H	7.026343	-0.982091	-1.741325
H	3.65633	-0.042249	-3.736883	H	7.044977	0.82464	-1.74465
C	0.61805	-3.065361	0.031093	S	6.727906	-0.0716	0.503873
H	1.675516	-2.79243	0.070941	H	-0.970088	7.878097	-0.076712
C	0.219629	-4.390385	0.005501	H	-7.567325	1.099346	-0.178623
H	0.960126	-5.191137	0.024938	H	-3.086935	-7.204743	-0.148

Se-Me geometry optimized using M06

E_{M06} = -4568.15679164 Hartrees

Ru	-0.119457	-0.005383	0.007629	C	-1.4426	-4.689825	-0.040958
N	0.23782	-0.009792	2.087948	C	-2.361397	-3.645976	-0.053155
N	0.405115	-0.005845	-2.035184	H	-3.432937	-3.848737	-0.074134
N	-0.565575	-2.041103	-0.011493	C	-1.905926	-2.331597	-0.038891
N	-2.152978	0.005295	-0.047577	C	-2.811343	-1.174728	-0.052224
N	-0.544007	2.034929	-0.007616	C	-4.199953	-1.196008	-0.063204
C	2.494777	-0.019302	1.333325	H	-4.764493	-2.130097	-0.066398
C	1.842185	-0.015045	0.084991	C	-4.889211	0.019959	-0.067736
C	2.595086	-0.016823	-1.102654	C	-4.189349	1.230257	-0.061006
C	3.99577	-0.023001	-1.049246	H	-4.727062	2.179022	-0.062457
H	4.582919	-0.024104	-1.972464	C	-2.799394	1.190821	-0.050372
C	4.634647	-0.027459	0.196335	C	-1.882552	2.338531	-0.035602
C	3.890287	-0.025651	1.387287	C	-2.320668	3.657107	-0.049569
H	4.418704	-0.02932	2.348027	H	-3.387083	3.889518	-0.071866
C	1.559578	-0.016426	2.458477	C	-1.393227	4.692531	-0.036133
C	-0.70838	-0.0068	3.042658	C	-0.033895	4.379766	-0.008421
H	-1.746016	-0.001611	2.694697	H	0.729521	5.157972	0.002982
C	-0.411175	-0.010014	4.396164	C	0.342907	3.046602	0.004971
H	-1.220646	-0.007365	5.12766	H	1.396982	2.758917	0.02705
C	0.927693	-0.016674	4.784112	C	-1.860001	-6.117721	-0.05558
H	1.199891	-0.019414	5.841922	O	-1.085012	-7.045481	-0.043838
C	1.914028	-0.019907	3.809039	O	-3.187208	-6.259029	-0.083478
H	2.970857	-0.025193	4.084713	C	-6.373873	-0.027707	-0.076003
C	1.753129	-0.011617	-2.298277	O	-7.017716	-1.052311	-0.081649
C	-0.461967	-0.000949	-3.062401	O	-6.925347	1.189269	-0.075753
H	-1.523572	0.003359	-2.796088	C	-1.899869	6.091189	-0.054525
C	-0.057511	-0.001294	-4.387753	O	-3.073462	6.381412	-0.083833
H	-0.805548	0.002837	-5.181975	O	-0.913117	6.989157	-0.036041
C	1.308545	-0.00705	-4.667148	C	7.097475	-0.040373	-1.458255
H	1.664164	-0.007551	-5.699923	H	8.196914	-0.046463	-1.437609
C	2.213883	-0.012287	-3.616816	H	6.739242	-0.943561	-1.973786
H	3.289455	-0.017095	-3.806889	H	6.74937	0.866052	-1.975065
C	0.312212	-3.061956	-0.000795	Se	6.548568	-0.036234	0.400653
H	1.369044	-2.784634	0.020457	H	-1.326273	7.87471	-0.049807
C	-0.081037	-4.388735	-0.014895	H	-7.894214	1.065359	-0.081168
H	0.663114	-5.186252	-0.005112	H	-3.378477	-7.217112	-0.09093

S-Ar geometry optimized using M06

E_{M06} = -2679.13775790 Hartrees

Ru	-0.196567	-0.014023	0.011345
N	0.181546	-0.040081	2.0885
N	0.301236	-0.005874	-2.039577
N	-0.677521	-2.041538	-0.021001
N	-2.232936	0.02924	-0.030561
N	-0.590652	2.033474	0.017944
C	2.428752	-0.088323	1.307134
C	1.759689	-0.05949	0.067355
C	2.499645	-0.062786	-1.131098
C	3.893864	-0.100468	-1.086924
H	4.475062	-0.125866	-2.015898
C	4.567413	-0.126315	0.149086
C	3.822641	-0.115773	1.345489
H	4.35618	-0.111788	2.301567
C	1.507029	-0.073819	2.443169
C	-0.75283	-0.024434	3.054666
H	-1.794197	0.001957	2.719251
C	-0.439212	-0.040401	4.404419
H	-1.23953	-0.026327	5.145784
C	0.903853	-0.074765	4.77607
H	1.188625	-0.088493	5.830468
C	1.87803	-0.091895	3.789042
H	2.937972	-0.119454	4.051109
C	1.645253	-0.034619	-2.317785
C	-0.576254	0.022992	-3.057428
H	-1.63486	0.045346	-2.780561
C	-0.185139	0.024742	-4.386963
H	-0.941145	0.04911	-5.173227
C	1.177275	-0.005479	-4.681022
H	1.522036	-0.005796	-5.717421
C	2.093181	-0.035499	-3.64018
H	3.166568	-0.060476	-3.840899
C	0.184831	-3.075349	-0.021113
H	1.245878	-2.814559	-0.00147
C	-0.228487	-4.395923	-0.044424
H	0.503548	-5.204639	-0.043669
C	-1.594426	-4.675896	-0.068139
C	-2.497369	-3.618137	-0.068317
H	-3.57179	-3.804944	-0.086529
C	-2.02223	-2.311019	-0.045691
C	-2.909704	-1.139785	-0.046741
C	-4.298648	-1.138434	-0.058665
H	-4.878743	-2.062838	-0.072209
C	-4.967712	0.088581	-0.050517
C	-4.248589	1.287211	-0.031721
H	-4.770288	2.244785	-0.023923
C	-2.859385	1.224775	-0.022665
C	-1.924221	2.357876	-0.001013
C	-2.341812	3.683094	0.000031
H	-3.404562	3.932539	-0.015173
C	-1.398228	4.703833	0.020722
C	-0.044026	4.369897	0.039277
H	0.731657	5.135773	0.05555
C	0.312	3.030821	0.037186
H	1.361631	2.727023	0.051811
C	-2.03433	-6.096995	-0.093441
O	-1.274076	-7.036875	-0.092152
O	-3.363661	-6.21701	-0.117841
H	-3.569743	-7.172015	-0.132545
C	-6.453308	0.064365	-0.060175
O	-7.112302	-0.950298	-0.077728
O	-6.985993	1.289398	-0.047044
H	-7.956657	1.180341	-0.054911
C	-1.88383	6.110224	0.02124
O	-3.053261	6.417625	0.006461
O	-0.883676	6.99311	0.039368
H	-1.283429	7.884896	0.038501
C	6.031595	-0.172048	0.213034
C	6.836161	-0.646365	1.223035
S	7.01749	0.449439	-1.097341
C	8.226324	-0.523051	0.95181
H	6.435031	-1.096863	2.133828
C	8.483168	0.046069	-0.263551
H	9.010834	-0.855892	1.634218
H	9.445236	0.255555	-0.732186

Se-Ar geometry optimized using M06

E_{M06} = -4682.39155947 Hartrees

Ru	-0.515589	-0.019944	0.051367
N	-0.200754	-0.050693	2.137443
N	0.043032	-0.019673	-1.982186
N	-1.019907	-2.040273	-0.002395
N	-2.548653	0.048873	-0.051058
N	-0.883404	2.031957	0.047445
C	2.068586	-0.133388	1.425945
C	1.437123	-0.093976	0.166737
C	2.212194	-0.111293	-1.009277
C	3.603514	-0.174283	-0.9236
H	4.212218	-0.209818	-1.834773
C	4.24227	-0.214663	0.33123
C	3.460335	-0.187706	1.504476
H	3.964063	-0.193429	2.476594
C	1.112624	-0.102845	2.533767
C	-1.166139	-0.019884	3.072109
H	-2.19533	0.021423	2.702017
C	-0.89707	-0.038119	4.431294
H	-1.720626	-0.011378	5.146388
C	0.432797	-0.090817	4.845915
H	0.68306	-0.106565	5.909036
C	1.438547	-0.123553	3.891396
H	2.488469	-0.165182	4.189341
C	1.393992	-0.070382	-2.221166
C	-0.80513	0.020392	-3.024071
H	-1.870537	0.059802	-2.776523
C	-0.377024	0.012965	-4.341944
H	-1.109854	0.047203	-5.149476
C	0.992653	-0.039048	-4.59697
H	1.366606	-0.047197	-5.623203
C	1.878529	-0.081138	-3.530666
H	2.956555	-0.123787	-3.701385
C	-0.169261	-3.083562	0.013676
H	0.893523	-2.834731	0.067178
C	-0.59604	-4.398999	-0.034309
H	0.126714	-5.215898	-0.019516
C	-1.963693	-4.663249	-0.101011
C	-2.854666	-3.595452	-0.118105
H	-3.929904	-3.770829	-0.170955
C	-2.366355	-2.293856	-0.069258
C	-3.239379	-1.111792	-0.089657
C	-4.627321	-1.092557	-0.140954
H	-5.218737	-2.009314	-0.172884
C	-5.280868	0.142837	-0.149031
C	-4.547538	1.332241	-0.108215
H	-5.056639	2.296542	-0.113948
C	-3.16006	1.252368	-0.060147
C	-2.211516	2.37361	-0.01188
C	-2.61116	3.704313	-0.02622
H	-3.669585	3.967858	-0.075111
C	-1.655204	4.712675	0.021928
C	-0.306758	4.360929	0.083569
H	0.478494	5.116089	0.123078
C	0.031284	3.017305	0.09383
H	1.07583	2.699591	0.141234
C	-2.417568	-6.079121	-0.153759
O	-1.667167	-7.026832	-0.140856
O	-3.746791	-6.185325	-0.217406
H	-3.961383	-7.138139	-0.247453
C	-6.765766	0.136862	-0.200622
O	-7.435902	-0.869801	-0.241603
O	-7.284353	1.368032	-0.196455
H	-8.255519	1.270423	-0.234667
C	-2.122498	6.125159	0.002541
O	-3.286823	6.447114	-0.052207
O	-1.112446	6.995538	0.051153
H	-1.500508	7.892283	0.03504
C	5.702959	-0.295464	0.437928
C	6.446379	-0.793745	1.480319
C	7.861971	-0.730234	1.319439
H	5.979611	-1.234526	2.365893
C	8.284649	-0.177832	0.145637
H	8.555799	-1.106576	2.075777
H	9.310784	-0.036346	-0.19708
Se	6.848885	0.344727	-0.917228

O-Me+ geometry optimized using M06

E_{M06} = -2241.73242934 Hartrees

Ru	0.410976	-0.012672	0.036731	C	-1.036145	-4.665362	-0.05317
N	0.689701	-0.024235	2.136068	C	-1.942695	-3.610243	-0.067703
N	0.899354	-0.02206	-2.018389	H	-3.016321	-3.798184	-0.100667
N	-0.128856	-2.037752	0.000436	C	-1.468851	-2.304553	-0.040764
N	-1.684984	0.032753	-0.044525	C	-2.354986	-1.13068	-0.059512
N	-0.039779	2.035658	0.004058	C	-3.744911	-1.133462	-0.089312
C	2.953831	-0.07622	1.401538	H	-4.32418	-2.057971	-0.101781
C	2.322028	-0.057866	0.13149	C	-4.409414	0.093318	-0.101648
C	3.079398	-0.073006	-1.063106	C	-3.693432	1.291051	-0.08719
C	4.467653	-0.104904	-1.002471	H	-4.212977	2.249307	-0.097931
H	5.071717	-0.115488	-1.911833	C	-2.303369	1.223116	-0.058356
C	5.085324	-0.121795	0.265188	C	-1.368374	2.358261	-0.038608
C	4.332537	-0.108521	1.465932	C	-1.783268	3.682246	-0.065091
H	4.886162	-0.124138	2.408687	H	-2.844742	3.934053	-0.101115
C	2.002776	-0.057567	2.52204	C	-0.835043	4.699342	-0.046963
C	-0.269366	-0.003581	3.069906	C	0.515135	4.364661	-0.002033
H	-1.30157	0.023525	2.708571	H	1.293618	5.127372	0.013837
C	0.014272	-0.015042	4.429925	C	0.870066	3.023759	0.021514
H	-0.803489	0.00282	5.151498	H	1.918456	2.72024	0.055839
C	1.34391	-0.049408	4.833158	C	-1.473585	-6.091014	-0.082847
H	1.602838	-0.059546	5.893853	O	-0.707967	-7.024753	-0.066867
C	2.346608	-0.070994	3.869493	O	-2.800152	-6.209881	-0.129485
H	3.398943	-0.097755	4.158835	C	-5.900719	0.069313	-0.129526
C	2.245377	-0.052763	-2.2713	O	-6.552236	-0.947934	-0.147739
C	0.038545	-0.003153	-3.04304	O	-6.427628	1.293443	-0.131208
H	-1.024217	0.020611	-2.784574	C	-1.318105	6.10998	-0.081533
C	0.457082	-0.013112	-4.368151	O	-2.486338	6.413083	-0.132617
H	-0.28441	0.003207	-5.16795	O	-0.316224	6.987184	-0.051577
C	1.820491	-0.044061	-4.636911	O	6.408958	-0.150985	0.444765
H	2.183704	-0.05297	-5.666514	C	7.260213	-0.165448	-0.693638
C	2.721922	-0.064368	-3.578062	H	8.285362	-0.186113	-0.305609
H	3.798085	-0.090054	-3.760511	H	7.085691	-1.062377	-1.309982
C	0.73995	-3.063382	0.01402	H	7.120593	0.739713	-1.306917
H	1.80011	-2.803778	0.045937	H	-3.010664	-7.164403	-0.145522
C	0.32625	-4.385492	-0.011654	H	-7.39967	1.192836	-0.151451
H	1.060381	-5.191923	0.000685	H	-0.708345	7.882505	-0.076945

S-Me^{+} geometry optimized using M06

E_{M06} = -2564.72585286 Hartrees

Ru	-0.252606	-0.007565	0.044188	C	1.13974	-4.677523	-0.055444
N	-0.73898	-0.007839	-2.011566	C	2.058011	-3.632656	-0.072825
N	-0.532611	-0.016737	2.142312	H	3.129389	-3.832218	-0.111704
N	0.262057	-2.039655	0.006604	C	1.598681	-2.321814	-0.040836
N	1.84258	0.012943	-0.041203	C	2.498326	-1.158458	-0.060985
N	0.221747	2.035248	0.013232	C	3.887928	-1.178244	-0.096886
C	-2.919828	-0.030385	-1.058108	H	4.455462	-2.109927	-0.113134
C	-2.163584	-0.026985	0.138596	C	4.567325	0.04036	-0.110567
C	-2.796557	-0.037572	1.407618	C	3.865994	1.246737	-0.09227
C	-4.17757	-0.051816	1.470846	H	4.397644	2.198323	-0.104943
H	-4.707135	-0.061035	2.428927	C	2.475331	1.195713	-0.057456
C	-4.931702	-0.054349	0.27	C	1.554053	2.341853	-0.034917
C	-4.306725	-0.04352	-0.994426	C	1.985012	3.660556	-0.065664
H	-4.907465	-0.045195	-1.906342	H	3.049279	3.89916	-0.106605
C	-2.084746	-0.019646	-2.266003	C	1.04943	4.689176	-0.045835
C	0.123053	0.001598	-3.035691	C	-0.30451	4.371031	0.005918
H	1.185808	0.010541	-2.776461	H	-1.073456	5.143336	0.023846
C	-0.294439	0.000058	-4.361005	C	-0.675689	3.03458	0.033304
H	0.447866	0.008001	-5.160183	H	-1.727659	2.744251	0.07217
C	-1.65797	-0.011864	-4.631106	C	1.559917	-6.108215	-0.091395
H	-2.020331	-0.013602	-5.661042	O	0.783314	-7.032653	-0.066499
C	-2.560449	-0.021983	-3.573142	O	2.884183	-6.243013	-0.15585
H	-3.636652	-0.032166	-3.756634	C	6.058061	-0.001074	-0.144363
C	-1.845986	-0.031936	2.528693	O	6.697991	-1.025588	-0.165891
C	0.427188	-0.010033	3.075806	O	6.598768	1.217076	-0.146625
H	1.459432	0.002287	2.713847	C	1.54917	6.093798	-0.087033
C	0.144163	-0.017895	4.435857	O	2.720536	6.383039	-0.146142
H	0.962426	-0.011894	5.15706	O	0.557966	6.982917	-0.053136
C	-1.185712	-0.033402	4.839657	C	-7.322072	-0.072509	-1.203222
H	-1.444319	-0.040037	5.900446	H	-8.415262	-0.083884	-1.09193
C	-2.18906	-0.040564	3.876576	H	-7.023724	0.838221	-1.743076
H	-3.241139	-0.05269	4.167803	H	-7.005262	-0.973694	-1.748546
C	-0.617937	-3.0557	0.023821	S	-6.674902	-0.071316	0.487784
H	-1.675011	-2.784569	0.061456	H	0.96059	7.873436	-0.083283
C	-0.21912	-4.382321	-0.005332	H	7.569556	1.105756	-0.170912
H	-0.962149	-5.180495	0.010039	H	3.083228	-7.199888	-0.175208

Se-Me⁺ geometry optimized using M06

E_{M06} = -4567.98550544 Hartrees

Ru	0.065614	-0.004409	0.016008	C	1.428315	-4.684508	-0.045038
N	-0.396514	-0.001451	-2.046032	C	2.351957	-3.644335	-0.054197
N	-0.241644	-0.010443	2.11012	H	3.42279	-3.849564	-0.075033
N	0.563209	-2.040819	-0.010617	C	1.899077	-2.330811	-0.037049
N	2.158144	0.002805	-0.039989	C	2.806407	-1.173216	-0.047844
N	0.549491	2.03549	-0.003279	C	4.196201	-1.202086	-0.061683
C	-2.589396	-0.010751	-1.120855	H	4.757769	-2.137517	-0.067854
C	-1.845423	-0.011051	0.085889	C	4.88402	0.012004	-0.06556
C	-2.495624	-0.016191	1.347328	C	4.190423	1.223143	-0.057405
C	-3.877226	-0.021512	1.393698	H	4.728891	2.171012	-0.060254
H	-4.41886	-0.02582	2.345284	C	2.799089	1.181686	-0.044183
C	-4.617092	-0.021745	0.184754	C	1.884706	2.333658	-0.030533
C	-3.976048	-0.015982	-1.072657	C	2.32506	3.649665	-0.046775
H	-4.567334	-0.015617	-1.99162	H	3.391465	3.880867	-0.069724
C	-1.738932	-0.005165	-2.317516	C	1.39655	4.684765	-0.035226
C	0.477885	0.00373	-3.060276	C	0.039678	4.375436	-0.006361
H	1.537507	0.006457	-2.788271	H	-0.724053	5.153022	0.004026
C	0.076796	0.005531	-4.390424	C	-0.340741	3.041522	0.008381
H	0.829023	0.009811	-5.180349	H	-1.395146	2.758148	0.029861
C	-1.283623	0.001642	-4.677464	C	1.841609	-6.117243	-0.063819
H	-1.633166	0.002795	-5.711838	O	1.0601	-7.037897	-0.050838
C	-2.19861	-0.003855	-3.630712	O	3.166423	-6.259215	-0.097339
H	-3.272704	-0.007288	-3.82666	C	6.374467	-0.039191	-0.076181
C	-1.559872	-0.015741	2.479418	O	7.008614	-1.067638	-0.084638
C	0.705168	-0.009982	3.05752	O	6.923205	1.175597	-0.0743
H	1.742467	-0.005862	2.710205	C	1.905831	6.086063	-0.0591
C	0.403738	-0.014521	4.413345	O	3.079781	6.368756	-0.096548
H	1.212239	-0.013971	5.145537	O	0.919839	6.981528	-0.036209
C	-0.931845	-0.019774	4.799515	C	-7.0691	-0.03183	-1.455811
H	-1.20471	-0.023488	5.856758	H	-8.167114	-0.037661	-1.410835
C	-1.921444	-0.020423	3.822888	H	-6.725858	0.877589	-1.969308
H	-2.97782	-0.02466	4.098565	H	-6.716235	-0.936355	-1.971379
C	-0.32169	-3.052925	-0.002398	Se	-6.490304	-0.031163	0.38891
H	-1.377792	-2.776039	0.017817	H	1.328939	7.869396	-0.054307
C	0.07027	-4.381743	-0.018738	H	7.893437	1.05767	-0.081505
H	-0.67713	-5.175944	-0.011103	H	3.36075	-7.217146	-0.107325

S-Ar⁺ geometry optimized using M06

E_{M06} = -2678.96298449 Hartrees

Ru	-0.145179	-0.005189	0.027521	H	-3.508784	-3.849577	-0.068739
N	0.181658	-0.01327	2.120354	C	-1.985756	-2.330494	-0.025273
N	0.2976	0.000154	-2.041336	C	-2.892407	-1.172631	-0.03436
N	-0.650451	-2.040444	0.002166	C	-4.282159	-1.201496	-0.054467
N	-2.243928	0.002631	-0.021001	H	-4.843618	-2.136911	-0.065613
N	-0.63555	2.033998	0.014386	C	-4.969424	0.012665	-0.059043
C	2.42832	-0.020654	1.333239	C	-4.275695	1.223446	-0.046177
C	1.765489	-0.012682	0.080641	H	-4.813574	2.171604	-0.050809
C	2.496831	-0.010498	-1.132452	C	-2.884408	1.181152	-0.027268
C	3.879784	-0.017945	-1.087326	C	-1.970094	2.332897	-0.012476
H	4.458558	-0.02146	-2.016306	C	-2.409269	3.64913	-0.02838
C	4.55522	-0.027433	0.159757	H	-3.475428	3.881342	-0.051803
C	3.810348	-0.027337	1.369413	C	-1.479541	4.683145	-0.01586
H	4.340585	-0.026893	2.324655	C	-0.123164	4.372761	0.013837
C	1.503928	-0.019636	2.474989	H	0.641431	5.149462	0.025224
C	-0.754202	-0.012659	3.077941	C	0.256309	3.038457	0.027573
H	-1.79522	-0.007853	2.742357	H	1.310492	2.754542	0.049079
C	-0.437011	-0.017947	4.430616	C	-1.926056	-6.116799	-0.063964
H	-1.23721	-0.01724	5.171839	O	-1.143663	-7.036521	-0.049181
C	0.902494	-0.024176	4.801679	O	-3.250402	-6.259229	-0.105184
H	1.187262	-0.028481	5.855726	H	-3.444554	-7.217195	-0.118744
C	1.881138	-0.025124	3.813634	C	-6.460248	-0.038118	-0.077297
H	2.940813	-0.030232	4.076303	O	-7.093964	-1.066591	-0.091691
C	1.637722	-0.003674	-2.32301	O	-7.008182	1.17675	-0.075118
C	-0.584201	0.006737	-3.048756	H	-7.978523	1.059812	-0.088445
H	-1.641879	0.009667	-2.769666	C	-1.987662	6.085203	-0.039852
C	-0.192399	0.009694	-4.38196	O	-3.161371	6.36831	-0.078237
H	-0.950347	0.01512	-5.166345	O	-1.000857	6.979425	-0.016108
C	1.165547	0.005366	-4.678909	H	-1.408659	7.867928	-0.03449
H	1.507669	0.00725	-5.71571	C	6.003731	-0.039211	0.217738
C	2.088382	-0.001502	-3.638739	C	6.82189	-0.124174	1.331018
H	3.161028	-0.005446	-3.842449	S	6.982514	0.059847	-1.238218
C	0.235627	-3.05144	0.009438	C	8.200475	-0.111524	1.024246
H	1.291631	-2.774578	0.030977	H	6.437042	-0.200675	2.349291
C	-0.156005	-4.380391	-0.010177	C	8.442357	-0.015365	-0.321632
H	0.591901	-5.174102	-0.003658	H	8.992379	-0.172464	1.771952
C	-1.513687	-4.683542	-0.039297	H	9.40206	0.017286	-0.838528
C	-2.438072	-3.643996	-0.046313				

Se-Ar+ geometry optimized using M06

E_{M06} = -4682.21775208 Hartrees

Ru	-0.46391	-0.019937	0.065256	H	-3.912144	-3.781534	-0.149061
N	-0.21811	-0.031249	2.167328	C	-2.356908	-2.297674	-0.049404
N	0.055535	-0.034591	-1.982177	C	-3.234422	-1.117279	-0.080653
N	-1.016029	-2.040163	0.018953	C	-4.623603	-1.109112	-0.134823
N	-2.555936	0.041369	-0.052478	H	-5.209948	-2.028962	-0.158146
N	-0.895954	2.031808	0.025649	C	-5.278339	0.122862	-0.157331
C	2.056905	-0.075516	1.468667	C	-4.553527	1.315025	-0.129885
C	1.440386	-0.065331	0.191381	H	-5.065187	2.277346	-0.1492
C	2.218885	-0.074319	-0.993378	C	-3.164713	1.236612	-0.077517
C	3.598234	-0.092253	-0.896196	C	-2.221238	2.364543	-0.042822
H	4.211244	-0.101365	-1.80313	C	-2.625486	3.69162	-0.080712
C	4.230533	-0.100165	0.375487	H	-3.684212	3.951279	-0.138064
C	3.435911	-0.092983	1.555567	C	-1.67043	4.701926	-0.046075
H	3.927106	-0.092591	2.531264	C	-0.323956	4.357137	0.026035
C	1.088872	-0.059145	2.574711	H	0.460184	5.113623	0.055619
C	-1.193142	-0.015373	3.084931	C	0.020295	3.013701	0.059043
H	-2.219039	0.006763	2.705538	H	1.065872	2.703529	0.114346
C	-0.932605	-0.025658	4.449381	C	-2.3843	-6.083861	-0.11516
H	-1.7624	-0.01197	5.157183	O	-1.62476	-7.022369	-0.08842
C	0.390483	-0.053568	4.874768	O	-3.710258	-6.194762	-0.19111
H	0.631621	-0.062522	5.939672	H	-3.925443	-7.148039	-0.216928
C	1.40925	-0.070613	3.928414	C	-6.768965	0.110126	-0.210808
H	2.456185	-0.093293	4.237024	O	-7.427457	-0.90225	-0.24454
C	1.404745	-0.061024	-2.215573	O	-7.28722	1.338009	-0.216261
C	-0.791043	-0.024214	-3.019388	H	-8.259368	1.244268	-0.255354
H	-1.857422	-0.003886	-2.775983	C	-2.142703	6.115794	-0.0929
C	-0.353775	-0.038101	-4.337896	O	-3.307792	6.427023	-0.163862
H	-1.083598	-0.028855	-5.148469	O	-1.135326	6.98617	-0.049223
C	1.013773	-0.063332	-4.586902	H	-1.520696	7.8841	-0.083041
H	1.391818	-0.074571	-5.611158	C	5.674387	-0.11107	0.486567
C	1.900004	-0.075115	-3.515392	C	6.43043	-0.194946	1.64368
H	2.978742	-0.096215	-3.682296	C	7.83337	-0.179586	1.463143
C	-0.154283	-3.071509	0.043839	H	5.979971	-0.27518	2.635501
H	0.906733	-2.818968	0.097833	C	8.242138	-0.080569	0.158614
C	-0.575747	-4.390637	0.002366	H	8.535611	-0.242544	2.29723
H	0.152742	-5.201976	0.024253	H	9.266992	-0.049552	-0.214543
C	-1.938894	-4.661282	-0.067566	Se	6.815565	0.003359	-1.016227
C	-2.838281	-3.600214	-0.093418				

O-Me geometry optimized using mPW1PW91

$E_{mPW1PW91}$ = -2242.92361392 Hartrees

Ru	0.34626	-0.016461	0.035243
N	0.699381	-0.025693	2.108822
N	0.880844	-0.037231	-1.997481
N	-0.141126	-2.028707	0.016535
N	-1.664472	0.052143	-0.039087
N	-0.003929	2.024369	0.012474
C	2.961374	-0.10707	1.367936
C	2.309077	-0.084834	0.120069
C	3.068403	-0.112736	-1.058916
C	4.471968	-0.162508	-1.008622
H	5.062526	-0.18263	-1.923598
C	5.10356	-0.184452	0.241417
C	4.355021	-0.157479	1.431029
H	4.900378	-0.177247	2.376014
C	2.017952	-0.071959	2.487955
C	-0.247075	0.010785	3.0618
H	-1.278182	0.047151	2.712445
C	0.043052	0.003826	4.418072
H	-0.769776	0.035135	5.142225
C	1.378406	-0.043318	4.814518
H	1.645853	-0.050245	5.871436
C	2.366813	-0.08148	3.841168
H	3.418787	-0.118179	4.12085
C	2.228908	-0.08508	-2.256862
C	0.022753	-0.008924	-3.030985
H	-1.03513	0.028328	-2.773498
C	0.43172	-0.025608	-4.356014
H	-0.313339	-0.000722	-5.149944
C	1.797577	-0.074527	-4.631783
H	2.157656	-0.089553	-5.660773
C	2.695782	-0.104444	-3.574771
H	3.768503	-0.143787	-3.759784
C	0.70884	-3.071672	0.039056
H	1.767185	-2.820921	0.075279
C	0.28555	-4.389657	0.017935
H	1.011525	-5.200279	0.037799
C	-1.082354	-4.659575	-0.028141
C	-1.974255	-3.592036	-0.051653
H	-3.046376	-3.769057	-0.088568
C	-1.48576	-2.288532	-0.028962
C	-2.35931	-1.107129	-0.055129
C	-3.747936	-1.089662	-0.091833
H	-4.335875	-2.005105	-0.104192
C	-4.403498	0.144881	-0.111852
C	-3.668219	1.33404	-0.095544
H	-4.174396	2.296072	-0.110786
C	-2.279875	1.254302	-0.058708
C	-1.329065	2.374545	-0.034732
C	-1.724507	3.707584	-0.059281
H	-2.77919	3.973673	-0.097766
C	-0.763801	4.712975	-0.034873
C	0.583499	4.352779	0.013755
H	1.373322	5.10042	0.034451
C	0.913767	3.006818	0.035503
H	1.952728	2.685403	0.073172
C	-1.537881	-6.077288	-0.05193
O	-0.785739	-7.024627	-0.037176
O	-2.867745	-6.188206	-0.091557
C	-5.889923	0.138851	-0.148874
O	-6.559399	-0.870046	-0.166067
O	-6.412561	1.368307	-0.160849
C	-1.223553	6.129336	-0.06379
O	-2.387843	6.455934	-0.110291
O	-0.211187	6.997931	-0.033545
O	6.452605	-0.232412	0.413667
C	7.267709	-0.263435	-0.740958
H	8.302525	-0.299785	-0.380729
H	7.071139	-1.156062	-1.357191
H	7.1351	0.638759	-1.360383
H	-3.079168	-7.140452	-0.104472
H	-7.382109	1.266617	-0.186759
H	-0.597404	7.893625	-0.054214

S-Me geometry optimized using mPW1PW91

$E_{mPW1PW91}$ = -2565.94221193 Hartrees

Ru	0.18939	-0.009137	0.041735	C	-1.152862	-4.678643	-0.037712
N	0.531957	-0.014078	2.116523	C	-2.064853	-3.628096	-0.062639
N	0.726696	-0.017297	-1.990286	H	-3.133527	-3.824549	-0.10544
N	-0.26152	-2.03091	0.018911	C	-1.600592	-2.315884	-0.033424
N	-1.824175	0.020925	-0.040276	C	-2.496198	-1.151038	-0.060377
N	-0.201353	2.025042	0.017973	C	-3.884852	-1.160463	-0.101218
C	2.79708	-0.046675	1.383621	H	-4.454483	-2.087365	-0.116718
C	2.148596	-0.037588	0.133753	C	-4.563554	0.061347	-0.121085
C	2.911988	-0.047783	-1.045344	C	-3.851352	1.264338	-0.101896
C	4.313077	-0.067011	-0.987012	H	-4.376497	2.216135	-0.117967
H	4.904285	-0.074375	-1.901972	C	-2.461809	1.210826	-0.061307
C	4.947392	-0.076076	0.262909	C	-1.532682	2.349116	-0.03509
C	4.192313	-0.066168	1.450277	C	-1.954184	3.673889	-0.064782
H	4.711843	-0.073859	2.410894	H	-3.013633	3.919094	-0.108539
C	1.849421	-0.033119	2.501125	C	-1.013416	4.697968	-0.039388
C	-0.419495	-0.000067	3.065068	C	0.340289	4.364435	0.016529
H	-1.449531	0.014922	2.711017	H	1.115178	5.127522	0.039119
C	-0.135221	-0.003915	4.422541	C	0.696806	3.025152	0.043116
H	-0.951685	0.008113	5.143189	H	1.741813	2.724699	0.086135
C	1.199047	-0.023214	4.824678	C	-1.580674	-6.104955	-0.070088
H	1.462026	-0.026817	5.882731	O	-0.810697	-7.037565	-0.045281
C	2.192518	-0.037918	3.855774	O	-2.907261	-6.240809	-0.130611
H	3.243405	-0.052958	4.141485	C	-6.049836	0.028361	-0.160974
C	2.075408	-0.03621	-2.246708	O	-6.701251	-0.992115	-0.180453
C	-0.12949	-0.005896	-3.025684	O	-6.593495	1.248544	-0.171756
H	-1.188514	0.008704	-2.770775	C	-1.500579	6.10508	-0.075929
C	0.283411	-0.012128	-4.349618	O	-2.670766	6.408671	-0.128395
H	-0.45994	-0.002158	-5.145501	O	-0.505317	6.993081	-0.045563
C	1.650497	-0.031248	-4.622106	C	7.351335	-0.103505	-1.222264
H	2.013376	-0.036836	-5.650191	H	8.444725	-0.117353	-1.121098
C	2.546603	-0.04339	-3.562757	H	7.03382	-1.000066	-1.771777
H	3.620356	-0.058866	-3.745138	H	7.056248	0.803983	-1.766337
C	0.607779	-3.057519	0.043965	S	6.718088	-0.10067	0.469897
H	1.661131	-2.787634	0.08642	H	-0.9087	7.881107	-0.071422
C	0.209252	-4.383198	0.017558	H	-7.561215	1.130806	-0.199253
H	0.950337	-5.179975	0.039458	H	-3.101328	-7.196615	-0.147546

Se-Me geometry optimized using mPW1PW91

$E_{mPW1PW91}$ = -4569.43475868 Hartrees

Ru	-0.129547	-0.006052	0.025862	C	-1.437421	-4.685782	-0.029101
N	0.243081	-0.009219	2.095579	C	-2.357715	-3.642293	-0.045141
N	0.376277	-0.009009	-2.014505	H	-3.425376	-3.846846	-0.073487
N	-0.565638	-2.031241	0.009564	C	-1.903051	-2.326541	-0.025229
N	-2.14468	0.008498	-0.02912	C	-2.807786	-1.168548	-0.042489
N	-0.536497	2.02497	0.010246	C	-4.19669	-1.18865	-0.068774
C	2.497104	-0.022668	1.328925	H	-4.759164	-2.119953	-0.079025
C	1.830569	-0.018885	0.088605	C	-4.884797	0.027946	-0.080986
C	2.575877	-0.022486	-1.103028	C	-4.181707	1.236365	-0.068295
C	3.977672	-0.030225	-1.064508	H	-4.714497	2.183971	-0.078202
H	4.556634	-0.033041	-1.987749	C	-2.791485	1.193484	-0.042192
C	4.627481	-0.033958	0.176276	C	-1.870857	2.33885	-0.024773
C	3.894344	-0.030133	1.374911	C	-2.302994	3.660358	-0.044618
H	4.428044	-0.032842	2.327726	H	-3.364747	3.897388	-0.073569
C	1.566286	-0.017407	2.460164	C	-1.36989	4.691614	-0.02834
C	-0.693755	-0.003907	3.058744	C	-0.013052	4.368452	0.008686
H	-1.729212	0.002459	2.720851	H	0.756011	5.137562	0.023414
C	-0.388633	-0.006237	4.411659	C	0.354172	3.031938	0.026592
H	-1.193999	-0.001649	5.14475	H	1.401932	2.739593	0.055039
C	0.951779	-0.014509	4.793382	C	-1.854385	-6.115513	-0.051248
H	1.230904	-0.016632	5.847268	O	-1.076705	-7.041871	-0.033572
C	1.930271	-0.020149	3.809368	O	-3.180516	-6.261965	-0.093723
H	2.985632	-0.026725	4.078334	C	-6.371178	-0.015905	-0.105996
C	1.720916	-0.017021	-2.291514	O	-7.015505	-1.040977	-0.118805
C	-0.495475	-0.003652	-3.036953	O	-6.92341	1.200432	-0.112234
H	-1.550672	0.002439	-2.766657	C	-1.867923	6.095159	-0.053702
C	-0.102511	-0.00567	-4.366937	O	-3.040848	6.390369	-0.090597
H	-0.857925	-0.00103	-5.151418	O	-0.878882	6.990295	-0.032352
C	1.260346	-0.013709	-4.660137	C	7.096562	-0.040459	-1.50314
H	1.607679	-0.015593	-5.693559	H	8.193933	-0.044564	-1.47029
C	2.172366	-0.019446	-3.61442	H	6.742525	-0.941627	-2.019259
H	3.243368	-0.02601	-3.812702	H	6.749067	0.866786	-2.013052
C	0.311738	-3.051131	0.025656	Se	6.542279	-0.044718	0.356669
H	1.363445	-2.773341	0.05388	H	-1.289043	7.875444	-0.050601
C	-0.077035	-4.379833	0.007653	H	-7.890555	1.076092	-0.12955
H	0.67032	-5.170864	0.021804	H	-3.367459	-7.219266	-0.105318

S-Ar geometry optimized using mPW1PW91

$E_{mPW1PW91}$ = -2680.24697356 Hartrees

Ru	-0.206088	-0.016595	0.021333
N	0.179774	-0.046548	2.088598
N	0.279869	-0.009629	-2.024574
N	-0.685694	-2.032065	-0.010775
N	-2.223403	0.040421	-0.019719
N	-0.573035	2.022965	0.029781
C	2.426383	-0.103605	1.303063
C	1.748576	-0.072325	0.068593
C	2.48495	-0.077429	-1.131325
C	3.880476	-0.119895	-1.097145
H	4.456032	-0.147338	-2.024722
C	4.560253	-0.146881	0.136456
C	3.821655	-0.13383	1.337444
H	4.358496	-0.130842	2.287073
C	1.505197	-0.0862	2.442078
C	-0.748949	-0.028481	3.059367
H	-1.786712	0.002402	2.730081
C	-0.432766	-0.047527	4.409613
H	-1.231733	-0.031127	5.149509
C	0.910243	-0.087796	4.780075
H	1.197841	-0.104101	5.831553
C	1.880336	-0.107551	3.787928
H	2.937591	-0.13984	4.047516
C	1.621048	-0.044631	-2.313312
C	-0.600067	0.024589	-3.039292
H	-1.652632	0.051895	-2.760353
C	-0.217857	0.025596	-4.372488
H	-0.979337	0.05458	-5.15055
C	1.141798	-0.011298	-4.67729
H	1.480525	-0.012568	-5.713535
C	2.062114	-0.046717	-3.639258
H	3.131065	-0.076843	-3.846323
C	0.170201	-3.070106	-0.008507
H	1.227721	-2.814805	0.01503
C	-0.246411	-4.390353	-0.034624
H	0.484389	-5.196852	-0.031913
C	-1.613018	-4.667335	-0.064047
C	-2.511051	-3.60456	-0.065649
H	-3.582718	-3.786846	-0.087811
C	-2.029063	-2.298891	-0.039466
C	-2.910047	-1.122358	-0.041958
C	-4.299274	-1.113698	-0.063116
H	-4.881485	-2.032659	-0.081667
C	-4.962075	0.116823	-0.058849
C	-4.234471	1.310315	-0.035331
H	-4.746934	2.269095	-0.031921
C	-2.845113	1.238287	-0.0169
C	-1.900734	2.364411	0.007319
C	-2.305605	3.694607	0.006285
H	-3.362492	3.953726	-0.012295
C	-1.351354	4.70625	0.029405
C	-0.001429	4.354965	0.052686
H	0.783653	5.107599	0.071303
C	0.338322	3.010961	0.051856
H	1.380102	2.697315	0.069564
C	-2.06134	-6.087558	-0.093992
O	-1.304101	-7.030794	-0.093202
O	-3.390757	-6.204542	-0.122742
H	-3.598326	-7.157571	-0.140711
C	-6.449428	0.102162	-0.079305
O	-7.112482	-0.910615	-0.101811
O	-6.97874	1.328426	-0.069728
H	-7.948083	1.222213	-0.08524
C	-1.821076	6.11994	0.027223
O	-2.988229	6.43852	0.007088
O	-0.814101	6.994751	0.049084
H	-1.206239	7.888171	0.046064
C	6.02678	-0.196067	0.190842
C	6.836354	-0.697053	1.186539
S	7.008221	0.450456	-1.102001
C	8.226279	-0.569634	0.909635
H	6.442343	-1.166275	2.087205
C	8.475379	0.028396	-0.295053
H	9.013065	-0.918671	1.577638
H	9.433646	0.247112	-0.761802

Se-Ar geometry optimized using mPW1PW91

$E_{mPW1PW91}$ = -4683.73432312 Hartrees

Ru	-0.525043	-0.025362	0.055265	H	-3.961993	-3.735425	-0.174522
N	-0.204849	-0.066214	2.132716	C	-2.385884	-2.273741	-0.069522
N	0.023995	-0.024696	-1.973856	C	-3.245375	-1.081336	-0.092764
N	-1.039467	-2.030844	0.000934	C	-4.633105	-1.046659	-0.153777
N	-2.53851	0.068777	-0.046822	H	-5.231949	-1.95429	-0.190675
N	-0.854178	2.020312	0.058399	C	-5.273039	0.19595	-0.166168
C	2.064079	-0.165896	1.418346	C	-4.52459	1.375761	-0.118576
C	1.425584	-0.119081	0.163589	H	-5.018746	2.344062	-0.127822
C	2.198741	-0.136954	-1.012924	C	-3.137962	1.277967	-0.058896
C	3.591306	-0.207003	-0.9359	C	-2.174096	2.38642	-0.002181
H	4.196533	-0.241435	-1.844236	C	-2.553866	3.723989	-0.00925
C	4.233946	-0.253353	0.31748	H	-3.604783	4.002922	-0.057814
C	3.456967	-0.226355	1.494875	C	-1.582123	4.717513	0.046798
H	3.963931	-0.236794	2.46069	C	-0.240201	4.341038	0.108509
C	1.10781	-0.131294	2.528139	H	0.557984	5.078611	0.153515
C	-1.164329	-0.031125	3.072549	C	0.074379	2.990949	0.111884
H	-2.189888	0.020146	2.70895	H	1.10915	2.657745	0.159445
C	-0.89274	-0.05694	4.4323	C	-2.480188	-6.061472	-0.144747
H	-1.714783	-0.025814	5.145952	O	-1.737918	-7.016464	-0.131346
C	0.436651	-0.122608	4.845757	O	-3.810235	-6.157767	-0.205545
H	0.689691	-0.144647	5.906006	H	-4.031162	-7.107616	-0.233964
C	1.438096	-0.16026	3.885769	C	-6.759184	0.208029	-0.230062
H	2.485205	-0.212275	4.180726	O	-7.438836	-0.792895	-0.27569
C	1.372515	-0.085728	-2.221053	O	-7.267203	1.443352	-0.231813
C	-0.823997	0.026242	-3.014733	H	-8.237283	1.354063	-0.27814
H	-1.88376	0.073579	-2.767218	C	-2.025174	6.139801	0.036358
C	-0.401414	0.019752	-4.335637	O	-3.184821	6.480527	-0.020681
H	-1.137914	0.06314	-5.136711	O	-1.003285	6.995316	0.096226
C	0.966038	-0.043349	-4.598595	H	-1.378051	7.896058	0.085423
H	1.336096	-0.051511	-5.62406	C	5.696186	-0.340965	0.418008
C	1.8535	-0.096509	-3.533089	C	6.44311	-0.880502	1.438222
H	2.927421	-0.14802	-3.707732	C	7.859173	-0.81304	1.274209
C	-0.201814	-3.08342	0.02258	H	5.98012	-1.351933	2.305839
H	0.858719	-2.846371	0.079043	C	8.277757	-0.216827	0.120041
C	-0.639939	-4.39603	-0.023541	H	8.555154	-1.217343	2.010451
H	0.076412	-5.215131	-0.003516	H	9.299544	-0.062587	-0.222102
C	-2.009612	-4.649028	-0.094843	Se	6.83927	0.349138	-0.915157
C	-2.888786	-3.570901	-0.117928				

O-Me+ geometry optimized using mPW1PW91

$E_{mPW1PW91}$ = -2242.75427500 Hartrees

Ru	0.40111	-0.022133	0.039151	C	-1.125467	-4.64056	-0.03626
N	0.692386	-0.039694	2.128699	C	-2.012057	-3.568007	-0.055764
N	0.873616	-0.040504	-2.013282	H	-3.085188	-3.737482	-0.089967
N	-0.171657	-2.026089	0.009663	C	-1.513851	-2.270359	-0.032127
N	-1.679053	0.068812	-0.036924	C	-2.375653	-1.078374	-0.054666
N	0.004303	2.025036	0.010983	C	-3.765699	-1.052424	-0.092032
C	2.953182	-0.139132	1.384184	H	-4.361376	-1.962416	-0.106147
C	2.312516	-0.108528	0.121891	C	-4.404858	0.187705	-0.110642
C	3.060683	-0.138771	-1.074671	C	-3.66241	1.369108	-0.093409
C	4.450071	-0.197941	-1.025796	H	-4.157001	2.336691	-0.108736
H	5.047352	-0.219337	-1.935161	C	-2.273617	1.270831	-0.056149
C	5.077273	-0.227141	0.23702	C	-1.312817	2.384751	-0.033107
C	4.333614	-0.198705	1.442625	C	-1.69335	3.720187	-0.056059
H	4.888799	-0.224619	2.380336	H	-2.74477	3.998626	-0.09284
C	2.005812	-0.100134	2.509463	C	-0.718274	4.712353	-0.031638
C	-0.257932	0.001916	3.070642	C	0.621921	4.338833	0.014349
H	-1.287361	0.049976	2.718833	H	1.421982	5.075223	0.034468
C	0.032218	-0.014116	4.429946	C	0.9392	2.987885	0.033563
H	-0.781521	0.021913	5.152514	H	1.974605	2.657474	0.068909
C	1.362561	-0.07616	4.827939	C	-1.591643	-6.059063	-0.059991
H	1.627571	-0.0907	5.885066	O	-0.841668	-7.006335	-0.049447
C	2.35732	-0.119891	3.856224	O	-2.920283	-6.156991	-0.093704
H	3.407359	-0.168405	4.139822	C	-5.898108	0.195058	-0.149072
C	2.215862	-0.100712	-2.277275	O	-6.567618	-0.811089	-0.171275
C	0.009785	-0.002085	-3.034623	O	-6.403903	1.427651	-0.155738
H	-1.046412	0.044766	-2.773555	C	-1.162524	6.137833	-0.059014
C	0.418398	-0.020989	-4.363429	O	-2.323292	6.470991	-0.109839
H	-0.328725	0.012264	-5.154833	O	-0.139943	6.99026	-0.022182
C	1.778578	-0.082962	-4.643411	O	6.402609	-0.282539	0.406788
H	2.134944	-0.100423	-5.673243	C	7.24944	-0.312913	-0.738122
C	2.684361	-0.123482	-3.588107	H	8.271423	-0.35113	-0.347173
H	3.755527	-0.174067	-3.777292	H	7.057049	-1.206528	-1.349677
C	0.677103	-3.067126	0.027628	H	7.123381	0.593205	-1.348629
H	1.73718	-2.827194	0.060418	H	-3.144908	-7.106746	-0.106999
C	0.241503	-4.382882	0.005635	H	-7.375744	1.343908	-0.18358
H	0.963206	-5.19716	0.021934	H	-0.509432	7.893482	-0.042359

S-Me^{+} geometry optimized using mPW1PW91

$E_{mPW1PW91}$ = -2565.77068090 Hartrees

Ru	-0.243359	-0.010201	0.048968
N	-0.71891	-0.013415	-2.002876
N	-0.531482	-0.020192	2.138024
N	0.273977	-2.02985	0.016064
N	1.837703	0.023169	-0.03354
N	0.209508	2.025355	0.020379
C	-2.906106	-0.046326	-1.061285
C	-2.156168	-0.039621	0.135534
C	-2.795223	-0.052642	1.398273
C	-4.178686	-0.072966	1.458391
H	-4.709309	-0.084242	2.41149
C	-4.927581	-0.078876	0.254635
C	-4.295106	-0.065441	-1.006191
H	-4.890639	-0.069662	-1.916908
C	-2.062487	-0.031756	-2.265388
C	0.144161	-0.000119	-3.025844
H	1.20168	0.013837	-2.766611
C	-0.266974	-0.003981	-4.35387
H	0.479612	0.007349	-5.146407
C	-1.628869	-0.022589	-4.632013
H	-1.987133	-0.026426	-5.661313
C	-2.533765	-0.036777	-3.575375
H	-3.606269	-0.052354	-3.762885
C	-1.845225	-0.041905	2.522123
C	0.422348	-0.008047	3.077355
H	1.451757	0.00934	2.722752
C	0.135623	-0.016666	4.43738
H	0.952077	-0.005965	5.157705
C	-1.194898	-0.038911	4.838911
H	-1.457389	-0.046345	5.896723
C	-2.193265	-0.051768	3.870025
H	-3.2431	-0.069108	4.157898
C	-0.602333	-3.047663	0.037363
H	-1.655361	-2.779769	0.077909
C	-0.202493	-4.374521	0.008084
H	-0.945846	-5.169001	0.026752
C	1.156531	-4.669181	-0.046626
C	2.072167	-3.621147	-0.066763
H	3.140546	-3.818289	-0.10829
C	1.608707	-2.31073	-0.033925
C	2.502301	-1.14271	-0.056319
C	3.892282	-1.155468	-0.098183
H	4.461443	-2.082199	-0.116415
C	4.565539	0.066441	-0.115663
C	3.85623	1.268056	-0.095446
H	4.378648	2.220898	-0.111657
C	2.465444	1.208131	-0.054461
C	1.535983	2.347867	-0.030546
C	1.95418	3.671605	-0.062157
H	3.01272	3.919829	-0.105631
C	1.00792	4.691239	-0.039124
C	-0.341985	4.356069	0.016811
H	-1.12053	5.115165	0.038039
C	-0.697263	3.014729	0.04426
H	-1.741493	2.714424	0.085987
C	1.582198	-6.100047	-0.084383
O	0.80658	-7.026138	-0.057261
O	2.906139	-6.234345	-0.152723
C	6.058345	0.034219	-0.156005
O	6.701858	-0.988715	-0.179751
O	6.595414	1.253536	-0.161976
C	1.492316	6.103182	-0.080119
O	2.661844	6.40285	-0.138569
O	0.494447	6.984524	-0.046914
C	-7.327565	-0.104692	-1.222523
H	-8.418051	-0.120445	-1.098548
H	-7.039399	0.806373	-1.762703
H	-7.013993	-1.002189	-1.771203
S	-6.669086	-0.103488	0.459245
H	0.889742	7.8766	-0.076041
H	7.564861	1.145345	-0.190758
H	3.105092	-7.189593	-0.172815

Se-Me⁺ geometry optimized using mPW1PW91

$E_{mPW1PW91}$ = -4569.26310821 Hartrees

Ru	0.074044	-0.00512	0.031888	C	1.422972	-4.680276	-0.032622
N	-0.371664	-0.005899	-2.027467	C	2.349985	-3.642159	-0.045562
N	-0.245916	-0.007673	2.116528	H	3.416699	-3.850752	-0.073549
N	0.567964	-2.030958	0.010459	C	1.900165	-2.326644	-0.022949
N	2.155113	0.004746	-0.022976	C	2.80689	-1.1686	-0.037592
N	0.54869	2.025243	0.011616	C	4.197018	-1.196943	-0.064709
C	-2.572815	-0.015209	-1.118771	H	4.75573	-2.130112	-0.076323
C	-1.839671	-0.013074	0.089802	C	4.884068	0.017373	-0.076265
C	-2.498207	-0.016113	1.343012	C	4.188005	1.226903	-0.063708
C	-3.883324	-0.021862	1.382845	H	4.721716	2.173568	-0.074575
H	-4.427966	-0.024215	2.327991	C	2.796348	1.182674	-0.036922
C	-4.612466	-0.024987	0.168922	C	1.879453	2.332692	-0.021932
C	-3.962425	-0.02124	-1.082715	C	2.313516	3.651569	-0.044363
H	-4.545811	-0.023107	-2.001774	H	3.37533	3.887353	-0.073333
C	-1.7113	-0.011002	-2.309876	C	1.379066	4.682204	-0.031132
C	0.506225	-0.001888	-3.038088	C	0.024682	4.362541	0.006433
H	1.560037	0.001932	-2.764007	H	-0.74504	5.130769	0.019379
C	0.114677	-0.002607	-4.371919	C	-0.346403	3.025401	0.026202
H	0.872966	0.000801	-5.153371	H	-1.394481	2.737128	0.053733
C	-1.243231	-0.007818	-4.670098	C	1.833023	-6.115809	-0.059661
H	-1.586379	-0.008639	-5.70455	O	1.046754	-7.033042	-0.037301
C	-2.163389	-0.01212	-3.626811	O	3.156127	-6.265376	-0.111882
H	-3.233243	-0.016509	-3.829522	C	6.376644	-0.03083	-0.101704
C	-1.565552	-0.013036	2.480382	O	7.010036	-1.060299	-0.116133
C	0.692845	-0.004667	3.071279	O	6.926325	1.183	-0.106382
H	1.727916	-0.000509	2.733408	C	1.879722	6.088553	-0.062486
C	0.384668	-0.006745	4.426542	O	3.053016	6.37573	-0.107236
H	1.189777	-0.004152	5.159625	O	0.891362	6.980897	-0.037221
C	-0.952318	-0.012201	4.807347	C	-7.070741	-0.037783	-1.499124
H	-1.2314	-0.014037	5.860931	H	-8.166814	-0.043267	-1.445903
C	-1.935028	-0.015425	3.822792	H	-6.728899	0.870265	-2.010471
H	-2.989717	-0.019791	4.092966	H	-6.719772	-0.942147	-2.010772
C	-0.319168	-3.039586	0.024677	Se	-6.49298	-0.035332	0.350136
H	-1.369672	-2.760393	0.051963	H	1.296982	7.868521	-0.059911
C	0.0666	-4.370717	0.004737	H	7.89484	1.064891	-0.124429
H	-0.685443	-5.157086	0.017049	H	3.344564	-7.222814	-0.125893

S-Ar^{+} geometry optimized using mPW1PW91

$E_{mPW1PW91}$ = -2680.07215147 Hartrees

Ru	-0.154121	-0.009512	0.03177	H	-3.525783	-3.832656	-0.069477
N	0.175379	-0.019747	2.115841	C	-1.999922	-2.318129	-0.02182
N	0.283394	-0.006904	-2.030856	C	-2.898731	-1.154165	-0.032368
N	-0.66611	-2.031115	0.008241	C	-4.289048	-1.17336	-0.057387
N	-2.23916	0.014455	-0.016262	H	-4.853952	-2.10277	-0.070694
N	-0.619893	2.023488	0.0193	C	-4.967956	0.045422	-0.064494
C	2.424096	-0.038662	1.329619	C	-4.263959	1.250159	-0.049528
C	1.760023	-0.028221	0.081687	H	-4.790857	2.200646	-0.056692
C	2.487142	-0.028314	-1.129456	C	-2.872533	1.196337	-0.025757
C	3.873068	-0.04054	-1.089899	C	-1.948247	2.340192	-0.009763
H	4.448319	-0.046512	-2.016506	C	-2.372786	3.662138	-0.026424
C	4.550656	-0.051261	0.155077	H	-3.432969	3.905666	-0.051641
C	3.809087	-0.049342	1.365431	C	-1.43072	4.685805	-0.011756
H	4.339802	-0.049912	2.315994	C	-0.078697	4.356395	0.020588
C	1.497049	-0.033291	2.472007	H	0.696731	5.118833	0.033923
C	-0.757802	-0.014057	3.075689	C	0.282964	3.016567	0.034698
H	-1.794738	-0.003636	2.743931	H	1.329098	2.721126	0.058294
C	-0.441497	-0.021147	4.429263	C	-1.956619	-6.107377	-0.063684
H	-1.242278	-0.016012	5.167033	O	-1.176321	-7.029714	-0.047223
C	0.897459	-0.034849	4.802226	O	-3.280851	-6.247605	-0.109884
H	1.182644	-0.040839	5.854129	H	-3.476164	-7.20368	-0.125022
C	1.874515	-0.041131	3.811811	C	-6.461117	0.006678	-0.088332
H	2.930933	-0.052113	4.075046	O	-7.100337	-1.019029	-0.10528
C	1.621908	-0.017511	-2.318097	O	-7.003137	1.223737	-0.088035
C	-0.598241	0.003838	-3.037974	H	-7.972428	1.111841	-0.105313
H	-1.651003	0.011966	-2.760152	C	-1.921501	6.096007	-0.034958
C	-0.211252	0.004479	-4.373206	O	-3.093038	6.391133	-0.071283
H	-0.972239	0.013471	-5.151954	O	-0.926663	6.980987	-0.012773
C	1.145396	-0.006745	-4.676134	H	-1.325705	7.871721	-0.02989
H	1.484937	-0.006958	-5.711746	C	6.004042	-0.064996	0.20788
C	2.069491	-0.017947	-3.636152	C	6.826217	-0.167022	1.317731
H	3.138587	-0.027473	-3.842774	S	6.978014	0.056093	-1.240462
C	0.215407	-3.044616	0.018102	C	8.205648	-0.149743	1.004847
H	1.267708	-2.77205	0.042416	H	6.448575	-0.258616	2.334291
C	-0.178724	-4.37335	-0.002847	C	8.441113	-0.033012	-0.341432
H	0.568669	-5.164189	0.00572	H	8.999234	-0.222344	1.746764
C	-1.537007	-4.674391	-0.036139	H	9.396648	0.007358	-0.860673
C	-2.457776	-3.630692	-0.04463				

Se-Ar⁺ geometry optimized using mPW1PW91

$E_{mPW1PW91}$ = -4683.56015234 Hartrees

Ru	-0.472189	-0.022088	0.068182	H	-3.922093	-3.766164	-0.154072
N	-0.226716	-0.036613	2.162499	C	-2.365448	-2.285939	-0.049317
N	0.044516	-0.037645	-1.97348	C	-3.236814	-1.101233	-0.084066
N	-1.026764	-2.030096	0.023698	C	-4.626167	-1.087199	-0.144704
N	-2.550517	0.051926	-0.051869	H	-5.213284	-2.002494	-0.170379
N	-0.88513	2.021857	0.03045	C	-5.27565	0.147451	-0.170544
C	2.051143	-0.087944	1.467678	C	-4.543818	1.335189	-0.139684
C	1.43506	-0.075805	0.194215	H	-5.046863	2.298259	-0.161363
C	2.21096	-0.08648	-0.987535	C	-3.155029	1.248676	-0.080288
C	3.593293	-0.108337	-0.893869	C	-2.204125	2.37034	-0.041083
H	4.205273	-0.119377	-1.796487	C	-2.595648	3.702183	-0.076999
C	4.225424	-0.116799	0.376313	H	-3.648417	3.971307	-0.136417
C	3.433092	-0.107897	1.556561	C	-1.630133	4.703066	-0.0366
H	3.924203	-0.107903	2.527996	C	-0.288044	4.341388	0.038541
C	1.078984	-0.069643	2.572724	H	0.505122	5.084723	0.07253
C	-1.199611	-0.019343	3.081917	C	0.040437	2.993315	0.069115
H	-2.221247	0.006744	2.705762	H	1.077976	2.672943	0.126472
C	-0.94109	-0.032942	4.447514	C	-2.407083	-6.074872	-0.11009
H	-1.771968	-0.017782	5.151045	O	-1.648914	-7.015235	-0.080673
C	0.380841	-0.065938	4.875975	O	-3.732726	-6.184714	-0.18969
H	0.621641	-0.077614	5.938919	H	-3.948281	-7.136272	-0.215675
C	1.398773	-0.084683	3.927804	C	-6.768154	0.142678	-0.230802
H	2.442158	-0.11159	4.237541	O	-7.429123	-0.868701	-0.266364
C	1.392729	-0.069607	-2.209085	O	-7.283746	1.37121	-0.239776
C	-0.798612	-0.023016	-3.013136	H	-8.254397	1.279864	-0.282798
H	-1.860719	0.001746	-2.773981	C	-2.086392	6.124343	-0.079506
C	-0.361732	-0.038109	-4.332655	O	-3.249073	6.44688	-0.151205
H	-1.092351	-0.025171	-5.13989	O	-1.072061	6.985957	-0.031041
C	1.005315	-0.069344	-4.583684	H	-1.449201	7.885747	-0.061586
H	1.38366	-0.081838	-5.605727	C	5.674281	-0.129323	0.484181
C	1.88927	-0.08536	-3.509598	C	6.437091	-0.220607	1.635184
H	2.965035	-0.111029	-3.675957	C	7.84152	-0.203779	1.4459
C	-0.169102	-3.063398	0.055183	H	5.994388	-0.306698	2.626686
H	0.887805	-2.814466	0.113694	C	8.242361	-0.096677	0.14036
C	-0.592282	-4.382709	0.01439	H	8.548042	-0.271699	2.273151
H	0.136149	-5.190653	0.041922	H	9.261288	-0.063176	-0.241568
C	-1.955452	-4.65226	-0.062219	Se	6.80639	-0.005432	-1.02336
C	-2.851429	-3.587723	-0.093876				

O-Me geometry optimized using BP86

E_{BP86} = -2243.52835398 Hartrees

Ru	0.343825	-0.021899	0.03359	C	-1.139514	-4.682966	-0.021733
N	0.70302	-0.036769	2.109082	C	-2.030184	-3.597612	-0.045686
N	0.881599	-0.048495	-2.000904	H	-3.113782	-3.767879	-0.080336
N	-0.166794	-2.02608	0.015584	C	-1.535151	-2.284159	-0.027567
N	-1.673524	0.073135	-0.039779	C	-2.398829	-1.091806	-0.055713
N	0.022676	2.02147	0.012299	C	-3.797923	-1.051286	-0.095201
C	2.981023	-0.145818	1.378403	H	-4.403033	-1.967676	-0.10717
C	2.320687	-0.115365	0.117824	C	-4.446769	0.202615	-0.118939
C	3.087842	-0.151526	-1.074935	C	-3.686133	1.39271	-0.102052
C	4.502795	-0.2175	-1.023052	H	-4.18302	2.370671	-0.119903
H	5.096373	-0.243154	-1.947626	C	-2.287492	1.299296	-0.061378
C	5.140497	-0.247292	0.238699	C	-1.316612	2.406301	-0.034922
C	4.386721	-0.212373	1.439687	C	-1.682451	3.759744	-0.054939
H	4.934809	-0.238453	2.394487	H	-2.741768	4.049217	-0.092395
C	2.037771	-0.099963	2.501265	C	-0.696015	4.75764	-0.026581
C	-0.260002	0.011206	3.068489	C	0.658037	4.363511	0.020723
H	-1.297037	0.060646	2.708773	H	1.469734	5.102066	0.043763
C	0.029512	-0.000168	4.436088	C	0.968387	3.003254	0.038119
H	-0.794568	0.040875	5.162112	H	2.009831	2.657854	0.075073
C	1.374256	-0.064064	4.844934	C	-1.603378	-6.105343	-0.040497
H	1.636944	-0.074821	5.913087	O	-0.850977	-7.074041	-0.027184
C	2.376137	-0.114079	3.869918	O	-2.957115	-6.20614	-0.07415
H	3.435985	-0.164342	4.157346	C	-5.940046	0.212477	-0.159984
C	2.24674	-0.112746	-2.274237	O	-6.636746	-0.798602	-0.175918
C	0.005656	-0.008239	-3.040624	O	-6.452581	1.471114	-0.177975
H	-1.058584	0.041334	-2.771539	C	-1.133689	6.18844	-0.049098
C	0.412548	-0.028075	-4.37777	O	-2.30592	6.54801	-0.09731
H	-0.345153	0.006901	-5.173113	O	-0.084837	7.048552	-0.010239
C	1.788385	-0.093143	-4.667591	O	6.50241	-0.310513	0.416167
H	2.142803	-0.111101	-5.708826	C	7.322523	-0.348157	-0.760181
C	2.701902	-0.135606	-3.609437	H	8.36591	-0.395059	-0.395391
H	3.78276	-0.188316	-3.80382	H	7.111625	-1.247071	-1.381378
C	0.683745	-3.092683	0.037681	H	7.193062	0.565222	-1.382607
H	1.753029	-2.845962	0.071163	H	-3.163182	-7.174211	-0.084277
C	0.244565	-4.415249	0.02004	H	-7.435899	1.364472	-0.206832
H	0.971321	-5.238669	0.039622	H	-0.472411	7.959276	-0.027804

S-Me geometry optimized using BP86

E_{BP86} = -2566.54675891 Hartrees

Ru	0.186005	-0.010604	0.040096	C	-1.166066	-4.712131	-0.037672
N	0.531942	-0.018526	2.117009	C	-2.087118	-3.652363	-0.061485
N	0.730222	-0.018874	-1.992581	H	-3.165638	-3.851916	-0.102298
N	-0.268843	-2.029279	0.015927	C	-1.628859	-2.325778	-0.034864
N	-1.834648	0.026414	-0.041757	C	-2.525892	-1.158406	-0.062271
N	-0.195297	2.023184	0.017364	C	-3.925537	-1.158371	-0.10445
C	2.815543	-0.056623	1.398328	H	-4.503113	-2.092299	-0.120088
C	2.160405	-0.044881	0.134865	C	-4.609911	0.076372	-0.125461
C	2.933756	-0.055503	-1.056257	C	-3.88389	1.287787	-0.105373
C	4.347155	-0.07795	-0.994435	H	-4.408892	2.25097	-0.122148
H	4.943165	-0.085371	-1.918062	C	-2.483228	1.234085	-0.063047
C	4.98569	-0.089839	0.267401	C	-1.544835	2.368498	-0.03523
C	4.223569	-0.079422	1.464921	C	-1.950471	3.710234	-0.061754
H	4.745064	-0.089095	2.435751	H	-3.017714	3.967775	-0.104364
C	1.865468	-0.041724	2.517255	C	-0.993871	4.73679	-0.035266
C	-0.438494	-0.002914	3.069944	C	0.370822	4.382785	0.018906
H	-1.474114	0.015266	2.703403	H	1.160333	5.144947	0.042105
C	-0.157586	-0.009059	4.439285	C	0.721053	3.032284	0.043288
H	-0.987186	0.00436	5.160024	H	1.772115	2.718225	0.085486
C	1.1857	-0.032383	4.856494	C	-1.589019	-6.147153	-0.067657
H	1.441878	-0.037869	5.926231	O	-0.809788	-7.094201	-0.046156
C	2.195101	-0.048745	3.887998	O	-2.938545	-6.284993	-0.122604
H	3.253493	-0.067142	4.184207	C	-6.103051	0.044895	-0.166756
C	2.097142	-0.040525	-2.260497	O	-6.771863	-0.984671	-0.185965
C	-0.142155	-0.00445	-3.035997	O	-6.648991	1.289233	-0.180174
H	-1.208485	0.012012	-2.771473	C	-1.473124	6.154092	-0.068166
C	0.271438	-0.009891	-4.371236	O	-2.655464	6.478712	-0.116691
H	-0.483418	0.002536	-5.169951	O	-0.449694	7.044536	-0.039418
C	1.649613	-0.031306	-4.655207	C	7.411571	-0.120189	-1.233893
H	2.008953	-0.036205	-5.694847	H	8.514535	-0.136931	-1.128661
C	2.559391	-0.046708	-3.592894	H	7.087723	-1.023862	-1.788326
H	3.642228	-0.064319	-3.782255	H	7.114714	0.797797	-1.779888
C	0.611305	-3.071388	0.039515	S	6.771566	-0.118554	0.478558
H	1.673077	-2.795428	0.080161	H	-0.864051	7.943356	-0.063369
C	0.209453	-4.405702	0.014336	H	-7.629252	1.157045	-0.20886
H	0.959059	-5.208315	0.034951	H	-3.119031	-7.257952	-0.138552

Se-Me geometry optimized using BP86

E_{BP86} = -4570.10753997 Hartrees

Ru	-0.13576	-0.006414	0.023755	C	-1.441335	-4.721497	-0.030685
N	0.239743	-0.0106	2.095602	C	-2.373411	-3.671261	-0.044881
N	0.377552	-0.009119	-2.017237	H	-3.450346	-3.881648	-0.071274
N	-0.570897	-2.029605	0.006596	C	-1.928343	-2.340046	-0.026534
N	-2.158106	0.009868	-0.030541	C	-2.837424	-1.181892	-0.043371
N	-0.538594	2.023262	0.008216	C	-4.237422	-1.19617	-0.069097
C	2.512701	-0.025176	1.343389	H	-4.80544	-2.135985	-0.078812
C	1.83971	-0.020583	0.089326	C	-4.934386	0.031555	-0.081363
C	2.595162	-0.023909	-1.114138	C	-4.220641	1.250293	-0.068837
C	4.009681	-0.032123	-1.07153	H	-4.755838	2.207945	-0.078609
H	4.594117	-0.034559	-2.003061	C	-2.819154	1.21084	-0.043041
C	4.662862	-0.0367	0.181061	C	-1.892197	2.35473	-0.025562
C	3.922893	-0.033272	1.389546	C	-2.312082	3.692199	-0.043595
H	4.458774	-0.03667	2.352509	H	-3.382416	3.938499	-0.070991
C	1.579297	-0.019802	2.47584	C	-1.365849	4.728528	-0.028309
C	-0.716148	-0.005003	3.063358	C	0.003001	4.388572	0.006455
H	-1.757383	0.002124	2.712882	H	0.784748	5.158894	0.020309
C	-0.414355	-0.007977	4.428203	C	0.36751	3.041815	0.023565
H	-1.23303	-0.003132	5.161426	H	1.422227	2.738634	0.050803
C	0.935274	-0.017183	4.82508	C	-1.849315	-6.160929	-0.051741
H	1.207601	-0.019812	5.890806	O	-1.05955	-7.099389	-0.037543
C	1.929856	-0.02312	3.841383	O	-3.197814	-6.313449	-0.089426
H	2.992908	-0.03048	4.120862	C	-6.427476	-0.014605	-0.105667
C	1.740378	-0.017503	-2.305752	O	-7.08657	-1.050576	-0.117548
C	-0.510409	-0.00298	-3.047616	O	-6.985244	1.224488	-0.112681
H	-1.572801	0.003297	-2.767583	C	-1.859373	6.141017	-0.051844
C	-0.116831	-0.004453	-4.388836	O	-3.045306	6.454474	-0.084005
H	-0.883745	0.00076	-5.176057	O	-0.844244	7.041138	-0.034419
C	1.25698	-0.012677	-4.693467	C	7.156383	-0.043637	-1.518668
H	1.600753	-0.014082	-5.738334	H	8.263527	-0.048001	-1.482421
C	2.182602	-0.019246	-3.644878	H	6.796997	-0.953857	-2.036352
H	3.262555	-0.026071	-3.850407	H	6.803979	0.872737	-2.030291
C	0.320052	-3.062691	0.020771	Se	6.597691	-0.047651	0.366596
H	1.379361	-2.775993	0.047354	H	-1.26762	7.935969	-0.051529
C	-0.068508	-4.400984	0.003194	H	-7.964525	1.083058	-0.129544
H	0.689423	-5.195864	0.015636	H	-3.368003	-7.288349	-0.100872

S-Ar geometry optimized using BP86

E_{BP86} = -2680.87729286 Hartrees

Ru	-0.212486	-0.01701	0.020249
N	0.174985	-0.04813	2.090072
N	0.282384	-0.00842	-2.026325
N	-0.690034	-2.031082	-0.012676
N	-2.236623	0.040778	-0.022197
N	-0.576257	2.021093	0.028565
C	2.441275	-0.105205	1.319803
C	1.757246	-0.072766	0.071555
C	2.504718	-0.075426	-1.139362
C	3.912259	-0.117087	-1.100084
H	4.493027	-0.144274	-2.036265
C	4.59856	-0.145175	0.145192
C	3.848824	-0.134588	1.355339
H	4.386204	-0.13034	2.315958
C	1.516415	-0.088608	2.459834
C	-0.773514	-0.029927	3.064682
H	-1.816949	0.001411	2.722259
C	-0.461451	-0.0494	4.427215
H	-1.274222	-0.032903	5.166829
C	0.890547	-0.090177	4.813611
H	1.170771	-0.106753	5.877174
C	1.877498	-0.110139	3.822257
H	2.94231	-0.142998	4.093119
C	1.641599	-0.04159	-2.325596
C	-0.613182	0.026484	-3.049272
H	-1.673165	0.052106	-2.761528
C	-0.229327	0.030017	-4.393497
H	-1.001704	0.059162	-5.174841
C	1.141363	-0.004481	-4.708713
H	1.477353	-0.003527	-5.75609
C	2.074611	-0.040462	-3.66714
H	3.152606	-0.069116	-3.8809
C	0.179554	-3.082119	-0.01006
H	1.244766	-2.817824	0.013223
C	-0.236632	-4.412202	-0.035048
H	0.505046	-5.222406	-0.031983
C	-1.615799	-4.704017	-0.063765
C	-2.525905	-3.63461	-0.066107
H	-3.606903	-3.822917	-0.088037
C	-2.053494	-2.313101	-0.041515
C	-2.939215	-1.136679	-0.044895
C	-4.339358	-1.122733	-0.06674
H	-4.926691	-2.050487	-0.085356
C	-5.011503	0.118722	-0.062996
C	-4.273609	1.322674	-0.038901
H	-4.788979	2.291161	-0.035986
C	-2.872909	1.254576	-0.019299
C	-1.922884	2.379609	0.005859
C	-2.31591	3.725305	0.005918
H	-3.38123	3.993453	-0.012342
C	-1.349031	4.742258	0.029441
C	0.012709	4.374774	0.052119
H	0.810045	5.12882	0.070834
C	0.350279	3.020692	0.050854
H	1.398976	2.696763	0.068515
C	-2.054999	-6.134472	-0.091825
O	-1.285975	-7.089957	-0.0913
O	-3.40677	-6.257085	-0.118652
H	-3.598271	-7.228016	-0.135683
C	-6.505698	0.101397	-0.084008
O	-7.18352	-0.922087	-0.105223
O	-7.040337	1.350454	-0.076697
H	-8.022131	1.227409	-0.092687
C	-1.815413	6.164544	0.028633
O	-2.995443	6.500114	0.008948
O	-0.783356	7.044933	0.051967
H	-1.189251	7.947947	0.049683
C	6.070289	-0.191356	0.202805
C	6.895033	-0.650279	1.227149
S	7.062176	0.405638	-1.137701
C	8.291898	-0.534671	0.94065
H	6.500356	-1.084636	2.156391
C	8.544884	0.014685	-0.301936
H	9.086824	-0.856654	1.628441
H	9.509444	0.215422	-0.78584

Se-Ar geometry optimized using BP86

E_{BP86} = -4684.43370987 Hartrees

Ru	-0.534461	-0.02711	0.059574	H	-4.001158	-3.761916	-0.170029
N	-0.213964	-0.066058	2.139983	C	-2.420458	-2.282995	-0.065566
N	0.025068	-0.032255	-1.969849	C	-3.280586	-1.087746	-0.097642
N	-1.053087	-2.029644	0.009604	C	-4.678526	-1.043528	-0.168366
N	-2.554206	0.074688	-0.049981	H	-5.285285	-1.958097	-0.205542
N	-0.853527	2.017822	0.058303	C	-5.323403	0.21214	-0.191305
C	2.074785	-0.164367	1.443054	C	-4.561138	1.400036	-0.142319
C	1.430869	-0.122347	0.173985	H	-5.055947	2.379056	-0.160376
C	2.216358	-0.139705	-1.012431	C	-3.163737	1.302184	-0.070122
C	3.620752	-0.205094	-0.928961	C	-2.190485	2.406309	-0.008146
H	4.231332	-0.240382	-1.845865	C	-2.552402	3.760813	-0.013081
C	4.269783	-0.245149	0.336496	H	-3.609989	4.054037	-0.063746
C	3.480019	-0.218805	1.522099	C	-1.564251	4.755406	0.048665
H	3.985453	-0.222414	2.499856	C	-0.212539	4.357305	0.114136
C	1.114039	-0.130013	2.553116	H	0.600571	5.092968	0.163142
C	-1.193974	-0.031721	3.082371	C	0.094131	2.995986	0.116711
H	-2.224506	0.018244	2.704506	H	1.134009	2.647966	0.167962
C	-0.927769	-0.056789	4.454449	C	-2.497094	-6.10395	-0.122001
H	-1.764071	-0.026571	5.166864	O	-1.746452	-7.073782	-0.103402
C	0.409951	-0.12047	4.885044	O	-3.849712	-6.201473	-0.18554
H	0.654638	-0.141693	5.957285	H	-4.05759	-7.168947	-0.210443
C	1.428876	-0.157434	3.927055	C	-6.815902	0.226179	-0.26714
H	2.48303	-0.20861	4.234207	O	-7.51346	-0.783192	-0.312191
C	1.392224	-0.091402	-2.225518	O	-7.324779	1.485964	-0.280936
C	-0.837166	0.015177	-3.020521	H	-8.307487	1.382968	-0.335294
H	-1.90497	0.060574	-2.765631	C	-1.998108	6.187916	0.041075
C	-0.411026	0.007559	-4.351906	O	-3.168489	6.550979	-0.021079
H	-1.157501	0.047812	-5.157536	O	-0.947871	7.043923	0.111089
C	0.968246	-0.052038	-4.623316	H	-1.332154	7.956193	0.100925
H	1.337043	-0.060305	-5.659591	C	5.737185	-0.320125	0.440304
C	1.867407	-0.101739	-3.5527	C	6.506694	-0.773671	1.505569
H	2.95099	-0.150848	-3.731958	C	7.928099	-0.714135	1.326198
C	-0.205551	-3.098154	0.039159	H	6.049365	-1.178829	2.420812
H	0.863424	-2.855354	0.098397	C	8.345488	-0.208708	0.112806
C	-0.647675	-4.419311	-0.002201	H	8.636206	-1.056902	2.096195
H	0.076479	-5.244783	0.024571	H	9.372352	-0.076755	-0.253217
C	-2.030827	-4.682547	-0.077526	Se	6.884466	0.270953	-0.97383
C	-2.918238	-3.594762	-0.10927				

O-Me+ geometry optimized using BP86

E_{BP86} = -2243.35524531 Hartrees

Ru	0.388109	-0.030095	0.036	C	-1.202952	-4.659991	-0.021085
N	0.6938	-0.05302	2.127356	C	-2.081279	-3.565072	-0.044008
N	0.868182	-0.057109	-2.019502	H	-3.166626	-3.722025	-0.076151
N	-0.202198	-2.024361	0.011754	C	-1.568433	-2.259276	-0.027749
N	-1.676597	0.097345	-0.038442	C	-2.412566	-1.051768	-0.055076
N	0.045944	2.022956	0.012539	C	-3.812648	-0.994885	-0.096052
C	2.969207	-0.192346	1.391191	H	-4.429745	-1.902869	-0.1079
C	2.315438	-0.152043	0.116285	C	-4.439963	0.266685	-0.122616
C	3.074153	-0.195838	-1.096151	C	-3.665189	1.444053	-0.106257
C	4.472268	-0.276888	-1.048521	H	-4.145037	2.429988	-0.127624
H	5.073743	-0.307249	-1.966762	C	-2.266848	1.326401	-0.062007
C	5.106053	-0.314495	0.226986	C	-1.282374	2.422831	-0.033725
C	4.359553	-0.273943	1.44697	C	-1.628263	3.780949	-0.049979
H	4.920648	-0.306818	2.392679	H	-2.682748	4.087092	-0.085943
C	2.022134	-0.135515	2.519826	C	-0.624659	4.760968	-0.018198
C	-0.269538	0.006306	3.078655	C	0.720468	4.347915	0.028027
H	-1.305332	0.071026	2.718697	H	1.545069	5.071808	0.053139
C	0.022237	-0.012066	4.449141	C	1.011951	2.981935	0.041305
H	-0.801295	0.038906	5.174867	H	2.048226	2.623894	0.077745
C	1.361832	-0.095177	4.857581	C	-1.684363	-6.080922	-0.036657
H	1.624348	-0.111702	5.925323	O	-0.938427	-7.052843	-0.026491
C	2.36678	-0.157722	3.881839	O	-3.036833	-6.163364	-0.062506
H	3.42503	-0.223499	4.170406	C	-5.939049	0.297294	-0.167315
C	2.225296	-0.142714	-2.298802	O	-6.641757	-0.707142	-0.183743
C	-0.011408	-0.00079	-3.048083	O	-6.428582	1.561069	-0.187608
H	-1.073652	0.065381	-2.776303	C	-1.040335	6.202655	-0.036813
C	0.394541	-0.026029	-4.38953	O	-2.206648	6.575296	-0.092437
H	-0.365237	0.022366	-5.181959	O	0.022315	7.040548	0.013705
C	1.763408	-0.113957	-4.684238	O	6.441682	-0.388689	0.401407
H	2.114102	-0.137527	-5.726145	C	7.302986	-0.428383	-0.76181
C	2.682972	-0.17311	-3.627393	H	8.329906	-0.473292	-0.357669
H	3.761642	-0.245302	-3.82563	H	7.098244	-1.3316	-1.372726
C	0.640456	-3.094798	0.032116	H	7.177827	0.486852	-1.376276
H	1.712711	-2.865242	0.062315	H	-3.259713	-7.128292	-0.070806
C	0.181729	-4.412188	0.01648	H	-7.414506	1.476836	-0.219776
H	0.899281	-5.243502	0.034771	H	-0.343168	7.960827	-0.002863

S-Me+ geometry optimized using BP86

E_{BP86} = -2566.37312197 Hartrees

Ru	-0.229678	-0.0112	0.046951	C	1.164692	-4.705682	-0.047509
N	-0.719059	-0.010544	-2.005954	C	2.087361	-3.647639	-0.067484
N	-0.529946	-0.027128	2.137218	H	3.165811	-3.846367	-0.108386
N	0.272741	-2.030601	0.013396	C	1.627819	-2.322374	-0.036666
N	1.835846	0.027425	-0.034923	C	2.521981	-1.151948	-0.059856
N	0.197892	2.025789	0.024823	C	3.92287	-1.15528	-0.103805
C	-2.925062	-0.050063	-1.07453	H	4.498775	-2.08983	-0.123045
C	-2.161332	-0.045463	0.135372	C	4.604098	0.078249	-0.12179
C	-2.811998	-0.063576	1.411365	C	3.880578	1.288006	-0.099431
C	-4.206836	-0.087602	1.471809	H	4.403006	2.252211	-0.11593
H	-4.742145	-0.103125	2.43371	C	2.478688	1.22992	-0.055894
C	-4.960672	-0.091316	0.255307	C	1.542445	2.367001	-0.028121
C	-4.324252	-0.072054	-1.018447	C	1.948739	3.707674	-0.05648
H	-4.926306	-0.074082	-1.936792	H	3.015721	3.965104	-0.10115
C	-2.079633	-0.030168	-2.280648	C	0.990155	4.731918	-0.029402
C	0.157923	0.007276	-3.038867	C	-0.371692	4.379179	0.028368
H	1.223153	0.021955	-2.771454	H	-1.162806	5.139408	0.053066
C	-0.254273	0.007049	-4.378293	C	-0.723669	3.027689	0.053295
H	0.50374	0.02191	-5.173764	H	-1.774747	2.716756	0.096942
C	-1.627128	-0.012531	-4.667819	C	1.586993	-6.144668	-0.08401
H	-1.982748	-0.01352	-5.708297	O	0.80358	-7.086528	-0.062023
C	-2.543801	-0.031478	-3.607267	O	2.934013	-6.279663	-0.145712
H	-3.625607	-0.048477	-3.800632	C	6.102772	0.046375	-0.163663
C	-1.858868	-0.053408	2.536527	O	6.764148	-0.98567	-0.185809
C	0.440553	-0.01493	3.083417	O	6.643596	1.2893	-0.173007
H	1.476105	0.006043	2.71775	C	1.46982	6.15284	-0.067273
C	0.156203	-0.027717	4.455203	O	2.652061	6.471654	-0.122626
H	0.985295	-0.016739	5.176301	O	0.445973	7.038961	-0.035308
C	-1.183566	-0.054384	4.870858	C	-7.390278	-0.116378	-1.230041
H	-1.44066	-0.065184	5.939969	H	-8.488863	-0.136189	-1.092765
C	-2.195747	-0.067436	3.900847	H	-7.100702	0.807611	-1.767715
H	-3.253534	-0.088414	4.197558	H	-7.069604	-1.019728	-1.784842
C	-0.612386	-3.066173	0.034835	S	-6.711223	-0.120801	0.464558
H	-1.674131	-2.793603	0.075324	H	0.854404	7.940819	-0.063007
C	-0.208236	-4.401074	0.006101	H	7.625411	1.165165	-0.202568
H	-0.959226	-5.202261	0.024548	H	3.120223	-7.251895	-0.165691

Se-Me^{+} geometry optimized using BP86

E_{BP86} = -4569.93539361 Hartrees

Ru	0.092388	-0.003705	0.027861	C	1.407781	-4.721624	-0.032143
N	-0.371576	-0.003119	-2.030986	C	2.347311	-3.678305	-0.044736
N	-0.240445	-0.005784	2.11279	H	3.422998	-3.894198	-0.071465
N	0.557347	-2.031558	0.008131	C	1.908594	-2.345633	-0.02448
N	2.154892	0.000849	-0.025715	C	2.822195	-1.190097	-0.039644
N	0.548457	2.026	0.009698	C	4.223274	-1.216109	-0.06581
C	-2.591048	-0.008983	-1.131231	H	4.783833	-2.160083	-0.076437
C	-1.843043	-0.008034	0.08898	C	4.924816	0.006292	-0.077449
C	-2.512195	-0.010499	1.355239	C	4.220698	1.227926	-0.065456
C	-3.909268	-0.014972	1.397089	H	4.759754	2.183086	-0.076105
H	-4.457682	-0.01713	2.351607	C	2.817782	1.193134	-0.039244
C	-4.644432	-0.017414	0.171764	C	1.899695	2.345119	-0.02351
C	-3.991605	-0.013531	-1.092952	C	2.329089	3.678819	-0.043659
H	-4.582665	-0.014311	-2.019055	H	3.400859	3.917868	-0.071715
C	-1.728403	-0.005728	-2.325029	C	1.387803	4.719335	-0.030148
C	0.520203	-0.000246	-3.052087	C	0.019258	4.38936	0.006311
H	1.581691	0.001623	-2.769915	H	-0.758905	5.16314	0.019636
C	0.127548	0.000311	-4.396949	C	-0.35559	3.044069	0.024952
H	0.896982	0.002681	-5.181566	H	-1.412293	2.750628	0.052442
C	-1.241541	-0.002316	-4.706008	C	1.806331	-6.16732	-0.057479
H	-1.582267	-0.00204	-5.751497	O	1.006796	-7.095844	-0.041987
C	-2.17307	-0.00543	-3.658723	O	3.151682	-6.325888	-0.101163
H	-3.25215	-0.007827	-3.867393	C	6.42281	-0.049184	-0.101254
C	-1.575353	-0.008871	2.493002	O	7.069229	-1.091053	-0.113133
C	0.716139	-0.004412	3.07393	O	6.982938	1.185528	-0.107165
H	1.757237	-0.002071	2.723877	C	1.890754	6.132076	-0.058996
C	0.411734	-0.005891	4.441115	O	3.078436	6.433107	-0.097556
H	1.230245	-0.004696	5.174334	O	0.88062	7.034417	-0.039285
C	-0.934598	-0.008935	4.837384	C	-7.135478	-0.033577	-1.503121
H	-1.207257	-0.010204	5.902719	H	-8.240289	-0.039197	-1.436037
C	-1.932104	-0.010468	3.852735	H	-6.787981	0.883442	-2.015878
H	-2.99463	-0.012955	4.132959	H	-6.778314	-0.948427	-2.013036
C	-0.343726	-3.053579	0.022352	Se	-6.535115	-0.027735	0.361703
H	-1.401484	-2.764124	0.04915	H	1.303829	7.929635	-0.060527
C	0.039187	-4.39475	0.003391	H	7.963005	1.046303	-0.123633
H	-0.724537	-5.183907	0.015299	H	3.321201	-7.301245	-0.115509

S-Ar$^+$ geometry optimized using BP86

E_{BP86} = -2680.70038162 Hartrees

Ru	-0.170305	-0.003082	0.030576
N	0.169345	-0.005703	2.115742
N	0.282548	-0.000263	-2.032694
N	-0.637782	-2.031448	0.011152
N	-2.238642	-0.002536	-0.017537
N	-0.636849	2.025341	0.016595
C	2.437666	-0.009114	1.345817
C	1.760678	-0.005112	0.08379
C	2.504071	-0.003914	-1.139649
C	3.899213	-0.008776	-1.097537
H	4.480724	-0.012938	-2.032272
C	4.585264	-0.015617	0.160783
C	3.832689	-0.013641	1.383049
H	4.367724	-0.010688	2.342586
C	1.506061	-0.007776	2.488708
C	-0.781928	-0.00523	3.081369
H	-1.824919	-0.003847	2.737262
C	-0.469846	-0.006476	4.44728
H	-1.284438	-0.006026	5.184832
C	0.8782	-0.00839	4.836249
H	1.156679	-0.009411	5.900036
C	1.87057	-0.009147	3.84598
H	2.934624	-0.010851	4.120262
C	1.637894	-0.00122	-2.331407
C	-0.612855	0.002291	-3.050073
H	-1.673367	0.003093	-2.76447
C	-0.224402	0.00385	-4.396469
H	-0.996428	0.005898	-5.178511
C	1.143297	0.002533	-4.710064
H	1.480594	0.003461	-5.756627
C	2.078703	-0.00009	-3.665825
H	3.157145	-0.001554	-3.878008
C	0.266294	-3.050714	0.022997
H	1.323504	-2.759513	0.048316
C	-0.113891	-4.392748	0.003113
H	0.651887	-5.179927	0.012859
C	-1.481531	-4.7226	-0.030849
C	-2.423638	-3.681637	-0.04068
H	-3.498876	-3.899849	-0.066053
C	-1.987827	-2.348094	-0.019623
C	-2.903318	-1.194182	-0.032182
C	-4.304491	-1.223368	-0.056952
H	-4.862812	-2.168618	-0.068431
C	-5.008217	-0.002522	-0.066089
C	-4.306719	1.220315	-0.053069
H	-4.847497	2.174489	-0.061739
C	-2.903612	1.187852	-0.028688
C	-1.988149	2.341763	-0.013266
C	-2.419901	3.674651	-0.03081
H	-3.492121	3.911837	-0.056244
C	-1.480116	4.716482	-0.018046
C	-0.111193	4.388872	0.014861
H	0.665959	5.163678	0.027222
C	0.266201	3.044134	0.03104
H	1.323491	2.752887	0.055555
C	-1.876382	-6.169688	-0.057784
O	-1.073934	-7.095556	-0.043589
O	-3.221084	-6.331496	-0.10164
H	-3.388584	-7.307243	-0.117377
C	-6.506678	-0.060918	-0.088722
O	-7.150434	-1.104223	-0.101576
O	-7.068583	1.17264	-0.092485
H	-8.048591	1.032474	-0.108425
C	-1.985471	6.128825	-0.043967
O	-3.17372	6.427491	-0.079638
O	-0.976655	7.032231	-0.024888
H	-1.400659	7.927179	-0.044193
C	6.036216	-0.027123	0.215859
C	6.874151	-0.098736	1.342347
S	7.023833	0.052938	-1.258493
C	8.255243	-0.090041	1.027143
H	6.490442	-0.162632	2.369051
C	8.495075	-0.010403	-0.340018
H	9.058663	-0.141302	1.774389
H	9.45998	0.015824	-0.863457

Se-Ar$^+$ geometry optimized using BP86

E_{BP86} = -4684.25823616 Hartrees

Ru	-0.492129	-0.007955	0.067069	H	-3.728014	3.960627	-0.134968
N	0.041458	-0.007366	-1.974714	C	-2.253705	2.375579	-0.036253
N	-0.234105	-0.027386	2.162357	C	-3.192803	1.241052	-0.079892
N	-0.912139	2.029994	0.039702	C	-4.592004	1.299131	-0.141427
N	-2.553629	0.035357	-0.052683	H	-5.129536	2.256105	-0.16173
N	-0.994903	-2.025379	0.019833	C	-5.320357	0.093182	-0.176369
C	2.226453	-0.037548	-0.99608	C	-4.644478	-1.144124	-0.152292
C	1.434413	-0.038921	0.196701	H	-5.205196	-2.08635	-0.180601
C	2.062981	-0.051737	1.483798	C	-3.242354	-1.141456	-0.089336
C	3.455342	-0.061921	1.573937	C	-2.351075	-2.314129	-0.054894
H	3.949406	-0.064685	2.554957	C	-2.808247	-3.63804	-0.098593
C	4.259845	-0.058812	0.38256	H	-3.883436	-3.854116	-0.160407
C	3.618398	-0.047578	-0.900762	C	-1.890014	-4.698566	-0.066213
H	4.234769	-0.046761	-1.812828	C	-0.516358	-4.398838	0.011865
C	1.407345	-0.022727	-2.22072	H	0.244311	-5.189423	0.041189
C	-0.814695	0.005213	-3.025602	C	-0.113219	-3.062251	0.052417
H	-1.885067	0.016908	-2.779051	H	0.948264	-2.791719	0.112001
C	-0.375131	0.003853	-4.355915	C	-2.057231	6.194209	-0.066016
H	-1.116273	0.014427	-5.167197	O	-1.234749	7.101484	-0.022544
C	1.003839	-0.01083	-4.616665	O	-3.395619	6.387339	-0.15608
H	1.380873	-0.012157	-5.64964	H	-3.539261	7.366747	-0.176951
C	1.898515	-0.024314	-3.537592	C	-6.816087	0.181654	-0.236595
H	2.984209	-0.0369	-3.708189	O	-7.43931	1.237207	-0.263417
C	1.086428	-0.047498	2.589474	O	-7.4017	-1.040889	-0.256973
C	-1.225211	-0.023725	3.087155	H	-8.378096	-0.882271	-0.297732
H	-2.252434	-0.008133	2.698313	C	-2.421399	-6.100337	-0.12072
C	-0.971148	-0.038341	4.46466	O	-3.61339	-6.376492	-0.194111
H	-1.81569	-0.03434	5.167667	O	-1.430776	-7.02315	-0.080908
C	0.359661	-0.057775	4.909096	H	-1.87089	-7.909565	-0.120613
H	0.593727	-0.069591	5.98352	C	5.705838	-0.063027	0.494799
C	1.392677	-0.062659	3.961433	C	6.483007	-0.121131	1.664607
H	2.443439	-0.078972	4.282237	C	7.887592	-0.104976	1.474066
C	0.012524	3.02982	0.084837	H	6.034173	-0.180719	2.665981
H	1.061769	2.715773	0.146249	C	8.296462	-0.031687	0.14756
C	-0.337728	4.379622	0.054649	H	8.603285	-0.147373	2.307951
H	0.443548	5.150503	0.092716	H	9.325633	-0.004846	-0.235484
C	-1.696013	4.738845	-0.026994	Se	6.857401	0.023534	-1.040092
C	-2.659381	3.718479	-0.072102				
