# Supplementary material for: Resolving orbital pathways for intermolecular electron transfer
Source: Nat Commun. 2018 Nov 21;9:4916. doi: 10.1038/s41467-018-07263-1 (PMC6249235; doi:10.1038/s41467-018-07263-1)
Supplement: Supplementary file 4 — Supplementary Data 2 [file 41467_2018_7263_MOESM4_ESM.pdf]

Supplementary Data 2 for

Resolving Orbital Pathways for Intermolecular Electron Transfer

TD-DFT Calculated Optical Transitions

Cameron W. Kellett[1]; Wesley B. Swords[2]; Michael D. Turlington[2]; Gerald J. Meyer[2,*]; Curtis P. Berlinguette[1,3,4,*]

correspondence to: gjmeyer@email.unc.edu; cberling@chem.ubc.ca

[1]Department of Chemistry, 2036 Main Mall, University of British Columbia, Vancouver, BC V6T 1Z1, Canada.

[2]Department of Chemistry, University of North Carolina at Chapel Hill, Murray Hall 2202B, Chapel Hill, NC 27599-3290, USA.

[3]Department of Chemical and Biological Engineering, 2360 East Mall, University of British Columbia, Vancouver, BC V6T 1Z3, Canada.

[4]Stewart Blusson Quantum Matter Institute, 2355 East Mall, University of British Columbia, Vancouver, BC V6T 1Z4, Canada

Calculated optical transitions. Optical transitions of the ruthenium complexes were predicted by time-dependent density functional theory (TD-DFT). Only the first transition and the six most intense transitions below 400 nm are reported.

	No.	Energy (cm-1)	Wavelength (nm)	Osc. Strength	Symmetry	Major contribs	Minor contribs
O-Me	1	13339.6	749.647	0.0001	Singlet-A	HOMO→LUMO (96%)	H-4→LUMO (3%)
	2	16519.8	605.332	0.0348	Singlet-A	H-2→LUMO (93%)	H-1→L+1 (3%)
	4	18545.1	539.226	0.0459	Singlet-A	H-2→L+1 (24%), H-1→LUMO (73%)	
	5	20027.6	499.312	0.1139	Singlet-A	H-1→L+1 (94%)	H-2→LUMO (2%)
	7	21900.4	456.613	0.085	Singlet-A	HOMO→L+3 (57%), HOMO→L+4 (39%)	
	8	22369.0	447.048	0.0932	Singlet-A	H-2→L+1 (59%), H-1→LUMO (15%)	H-2→L+3 (4%), H-1→L+2 (9%), HOMO→L+4 (2%), HOMO→L+5 (8%)
	9	22543.2	443.593	0.0657	Singlet-A	HOMO→L+3 (40%), HOMO→L+4 (55%)	
S-Me	1	13593.7	735.637	0.0001	Singlet-A	HOMO→LUMO (94%)	H-3→ LUMO (5%)
	2	16632.8	601.223	0.0353	Singlet-A	H-2→LUMO (94%)	H-1→L+1 (2%)
	4	18648.3	536.241	0.0504	Singlet-A	H-2→L+1 (23%), H-1→LUMO (73%)	
	5	20174.3	495.679	0.1117	Singlet-A	H-1→L+1 (95%)	H-2→LUMO (2%)
	7	21759.2	459.575	0.1026	Singlet-A	HOMO→L+3 (38%) HOMO→L+4 (57%)	

	No.	Energy (cm-1)	Wavelength (nm)	Osc. Strength	Symmetry	Major contribs	Minor contribs
	**8**	22458.5	445.266	0.1039	Singlet-A	H-2→L+1 (59%), H-1→LUMO (14%)	H-2→L+3 (4%) H-1→L+2 (8%) HOMO→L+3 (2%) HOMO→L+4 (2%) HOMO→L+5 (8%)
	**9**	22578.7	442.896	0.0319	Singlet-A	HOMO→L+3 (56%), HOMO→L+4 (37%)	H-2→L+1 (3%)
**Se-Me**	**1**	13717.9	728.976	0.0001	Singlet-A	HOMO→LUMO (92%)	H-3→LUMO (6%)
	**2**	16637.6	601.048	0.0354	Singlet-A	H-2→LUMO (94%)	H-1→L+1 (2%)
	**4**	18658.0	535.962	0.0514	Singlet-A	H-2→L+1 (23%), H-1→LUMO (74%)	
	**5**	20193.7	495.204	0.1111	Singlet-A	H-1→L+1 (95%)	
	**7**	21842.3	457.827	0.0999	Singlet-A	HOMO→L+3 (36%) HOMO→L+4 (59%)	
	**8**	22476.3	444.914	0.1109	Singlet-A	H-2→L+1 (62%) H-1→LUMO (15%)	H-2→L+3 (4%) H-1→L+2 (8%) HOMO→L+5 (7%)
	**9**	22681.9	440.880	0.0256	Singlet-A	HOMO→L+3 (60%), HOMO→L+4 (36%)	
**S-Ar**	**1**	14340.5	697.324	0.0	Singlet-A	HOMO→LUMO (94%)	H-3→LUMO (5%)
	**2**	16753.7	596.881	0.036	Singlet-A	H-2→LUMO (94%)	H-1→L+1 (2%)
	**4**	18758.0	533.105	0.0614	Singlet-A	H-2→L+1 (23%), H-1→LUMO (74%)	
	**5**	20342.9	491.572	0.1088	Singlet-A	H-1→L+1 (95%)	

	No.	Energy (cm-1)	Wavelength (nm)	Osc. Strength	Symmetry	Major contribs	Minor contribs
	**7**	22571.4	443.038	0.1378	Singlet-A	H-2→L+1 (49%), H-1→LUMO (12%), HOMO→L+4 (13%)	H-2→L+3 (3%), H-1→L+2 (6%), HOMO→L+3 (9%), HOMO→L+5 (5%)
	**8**	22598.0	442.516	0.1064	Singlet-A	H-2→L+1 (15%), HOMO→L+3 (29%), HOMO→L+4 (43%)	H-1→LUMO (4%)
	**9**	23437.7	426.664	0.0267	Singlet-A	HOMO→L+3 (57%), HOMO→L+4 (40%)	
**Se-Ar**	**1**	14252.6	701.625	0.0	Singlet-A	HOMO→LUMO (94%)	H-3→LUMO (5%)
	**2**	16734.4	597.572	0.0359	Singlet-A	H-2→LUMO (94%)	H-1→L+1 (2%)
	**4**	18740.3	533.610	0.0629	Singlet-A	H-2→L+1 (23%), H-1→LUMO (74%)	
	**5**	20322.8	492.059	0.1088	Singlet-A	H-1→L+1 (95%)	
	**7**	22539.2	443.672	0.1184	Singlet-A	H-2→L+1 (27%), HOMO→L+3 (23%), HOMO→L+4 (31%)	H-1→LUMO (6%), H-1→L+2 (3%), HOMO→L+5 (3%)
	**8**	22559.3	443.276	0.1275	Singlet-A	H-2→L+1 (37%), HOMO→L+3 (16%), HOMO→L+4 (24%)	H-2→L+3 (2%), H-1→LUMO (9%), H-1→L+2 (5%), HOMO→L+5 (4%)
	**9**	23339.3	428.462	0.0305	Singlet-A	HOMO→L+3 (57%), HOMO→L+4 (40%)	
**O-Me+**	**1**	5303.9	1885.404	0.0005	2.025-A	HOMO(B)→ LUMO(B) (92%)	H-3(B)→ LUMO(B) (2%), HOMO(B)→ L+6(B) (2%)
	**3**	15243.1	656.036	0.1498	2.024-A	H-2(B)→LUMO(B) (88%)	H-3(B)→ LUMO(B) (5%)

	No.	Energy (cm-1)	Wavelength (nm)	Osc. Strength	Symmetry	Major contribs	Minor contribs
	**4**	16974.7	589.110	0.0311	2.115-A	H-3(B)→LUMO(B) (87%)	H-2(B)→LUMO(B) (5%), HOMO(B)→LUMO(B) (3%)
	**9**	21662.4	461.629	0.0195	2.306-A	H-1(A)→LUMO(A) (69%), HOMO(B)→L+1(B) (15%)	H-3(A→LUMO(A) (6%), H-6(B)→LUMO(B) (4%)
	**10**	22256.1	449.316	0.0488	3.048-A	H-1(A)→L+3(A) (10%), H-5(B)→LUMO(B) (29%)	H-3(A)→L+3(A) (9%), H-3(A)→L+4(A) (3%), H-1(A)→L+4(A) (4%), HOMO(A)→L+3(A) (3%), HOMO(A)→L+5(A) (3%), H-3(B)→L+4(B) (2%), H-3(B)→L+5(B) (6%), H-2(B)→L+5(B) (4%), HOMO(B)→L+4(B) (2%), HOMO(B)→L+5(B) (6%)
	**14**	23294.1	429.293	0.0226	2.184-A	H-2(A)→LUMO(A) (47%), H-1(B)→L+1(B) (26%), HOMO(B)→L+2(B) (11%)	H-1(A)→L+1(A) (4%)
	**17**	23795.0	420.257	0.08	2.564-A	HOMO(A)→L+3(A) (60%), HOMO(A)→L+4(A) (17%)	HOMO(A)→L+2(A) (5%), H-5(B)→LUMO(B) (2%), H-3(B)→L+5(B) (2%), H-2(B)→L+5(B) (3%)
**S-Me^{+}**	**1**	5012.7	1994.919	0.0004	2.029-A	HOMO(B)→LUMO(B) (90%)	H-3(B)→LUMO(B) (2%), HOMO(B)→L+6(B) (2%)

	No.	Energy (cm-1)	Wavelength (nm)	Osc. Strength	Symmetry	Major contribs	Minor contribs
	**3**	13192.0	758.035	0.247	2.019-A	H-1(B)→LUMO(B) (90%)	H-5(B)→LUMO(B) (3%)
	**4**	16578.7	603.183	0.0267	2.112-A	H-3(B)→LUMO(B) (93%)	HOMO(B)→LUMO(B) (3%)
	**8**	20937.3	477.615	0.0348	2.542-A	H-5(B)→LUMO(B) (68%)	H-3(A)→L+2(A) (5%), H-1(A)→L+2(A) (4%), H-3(B)→L+4(B) (3%), H-3(B)→L+5(B) (4%), H-1(B)→LUMO(B) (3%), HOMO(B)→L+5(B) (2%)
	**10**	21597.9	463.008	0.0169	2.279-A	H-1(A)→LUMO(A) (62%), H-6(B)→LUMO(B) (13%), HOMO(B)→L+1(B) (13%)	H-3(A)→LUMO(A) (5%), H-10(B)→LUMO(B) (2%)
	**15**	23189.2	431.234	0.0368	2.282-A	H-2(A)→LUMO(A) (44%), H-2(B)→L+1(B) (25%)	H-1(A)→L+1(A) (3%), HOMO(A)→L+5(A) (2%), H-5(B)→LUMO(B) (2%), HOMO(B)→L+2(B) (7%), HOMO(B)→L+4(B) (2%)
	**16**	23529.6	424.996	0.0621	2.531-A	HOMO(A)→L+2(A) (64%), HOMO(A)→L+4(A) (12%)	H-2(A)→LUMO(A) (2%), HOMO(A)→L+3(A) (3%), H-5(B)→LUMO(B) (3%), H-1(B)→L+4(B) (2%), H-1(B)→L+5(B) (3%)
**Se-Me$^+$**	**1**	5157.1	1939.071	0.0004	2.029-A	H-1(B)→LUMO(B) (92%)	H-3(B)→LUMO(B) (2%), H-1(B)→L+6(B) (2%)

	No.	Energy (cm-1)	Wavelength (nm)	Osc. Strength	Symmetry	Major contribs	Minor contribs
	**3**	11748.3	851.189	0.297	2.017-A	HOMO(B)→LUMO(B) (93%)	H-5(B)→LUMO(B) (3%)
	**4**	16357.7	611.332	0.0255	2.107-A	H-3(B)→LUMO(B) (93%)	H-1(B)→LUMO(B) (2%)
	**8**	20227.6	494.375	0.0202	2.364-A	H-5(B)→LUMO(B) (78%)	H-3(A)→L+3(A) (3%), H-1(A)→L+3(A) (2%), H-3(B)→L+5(B) (3%), H-1(B)→L+5(B) (2%), HOMO(B)→LUMO(B) (3%)
	**10**	21383.4	467.653	0.0189	2.280-A	H-1(A)→LUMO(A) (65%), H-6(B)→LUMO(B) (10%), H-1(B)→L+1(B) (14%)	H-3(A)→LUMO(A) (5%)
	**15**	22969.9	435.353	0.0437	2.316-A	H-2(A)→LUMO(A) (42%), H-2(B)→L+1(B) (25%)	H-1(A→L+1(A) (4%), HOMO(A)→L+3(A) (2%), HOMO(A)→L+5(A) (2%), H-1(B)→L+2(B) (6%), H-1(B)→L+4(B) (2%)
	**17**	23240.1	430.292	0.0587	2.499-A	HOMO(A)→L+3(A) (63%), HOMO(A)→L+4(A) (17%)	H-2(A)→LUMO(A) (3%), HOMO(B)→L+5(B) (3%)
**S-Ar^{+}**	**1**	4602.2	2172.874	0.0004	2.041-A	H-1(B)→LUMO(B) (13%), HOMO(B)→LUMO(B) (77%)	H-3(B)→LUMO(B) (2%), HOMO(B)→L+6(B) (2%)
	**3**	12705.7	787.051	0.2979	2.054-A	H-1(B)→LUMO(B) (77%), HOMO(B)→LUMO(B) (13%)	HOMO(A)→L+5(A) (2%), H-6(B)→LUMO(B) (3%)
	**4**	16052.0	622.974	0.0252	2.114-A	H-3(B)→LUMO(B) (92%)	HOMO(B)→LUMO(B) (3%)

	No.	Energy (cm-1)	Wavelength (nm)	Osc. Strength	Symmetry	Major contribs	Minor contribs
	**8**	20417.1	489.785	0.0235	2.356-A	H-6(B)→LUMO(B) (79%)	H-3(A)→L+3(A) (2%), H-3(B)→L+5(B) (2%), H-1(B)→LUMO(B) (2%)
	**10**	21295.5	469.584	0.0461	2.361-A	H-4(B)→LUMO(B) (72%)	HOMO(A)→L+5(A) (3%), HOMO(A)→L+6(A) (3%), H-7(B)→LUMO(B) (4%), H-1(B)→L+8(B) (2%)
	**14**	22404.5	446.340	0.0984	2.928-A	HOMO(A)→L+5(A) (16%), HOMO(A)→L+6(A) (11%), H-4(B)→LUMO(B) (18%)	H-4(A)→L+6(A) (2%), H-3(A)→L+3(A) (4%), H-3(A)→L+4(A) (2%), H-2(A)→LUMO(A) (5%), H-1(A)→L+3(A) (5%), H-1(A)→L+4(A) (3%), H-6(B)→LUMO(B) (3%), H-3(B)→L+4(B) (2%), H-3(B)→L+5(B) (4%), H-2(B)→L+1(B) (3%), H-1(B)→L+6(B) (2%), H-1(B)→L+8(B) (7%), HOMO(B)→L+5(B) (2%)
	**21**	24304.7	411.443	0.0602	2.610-A	HOMO(A)→L+3(A) (59%), HOMO(A)→L+4(A) (24%)	H-1(B)→L+4(B) (2%), H-1(B)→L+5(B) (3%)
**$Se\text{-}Ar^{+}$**	**1**	4801.4	2082.718	0.0003	2.044-A	H-2(B)→LUMO(B) (16%), H-1(B)→LUMO(B) (66%)	H-1(B)→L+6(B) (2%), HOMO(B)→LUMO(B) (9%)

No.	Energy (cm-1)	Wavelength (nm)	Osc. Strength	Symmetry	Major contribs	Minor contribs
**3**	12233.8	817.406	0.3219	2.046-A	H-1(B)→LUMO(B) (11%), HOMO(B)→LUMO(B) (79%)	HOMO(A)→L+5(A) (2%), H-6(B)→LUMO(B) (3%)
**4**	15845.6	631.091	0.0209	2.113-A	H-4(B)→LUMO(B) (32%), H-3(B)→LUMO(B) (60%)	H-1(B)→LUMO(B) (3%)
**9**	20330.0	491.884	0.0239	2.339-A	H-6(B)→LUMO(B) (80%)	H-4(B)→L+5(B) (2%), H-1(B)→L+5(B) (2%), HOMO(B)→LUMO(B) (2%)
**12**	21830.2	458.081	0.1594	3.023-A	HOMO(A)→L+5(A) (22%), HOMO(A)→L+6(A) (15%), HOMO(B)→L+7(B) (11%)	H-5(A)→L+6(A) (3%), H-4(A)→L+3(A) (2%), H-4(A)→L+4(A) (2%), H-2(A)→LUMO(A) (3%), H-1(A)→LUMO(A) (2%), H-1(A)→L+3(A) (4%), H-1(A)→L+4(A) (3%), H-7(B)→LUMO(B) (3%), H-4(B)→L+5(B) (3%), H-2(B)→L+1(B) (2%), H-1(B)→L+5(B) (2%), HOMO(B)→L+6(B) (2%)
**13**	21881.0	457.017	0.0182	2.189-A	H-1(A)→LUMO(A) (19%), H-7(B)→LUMO(B) (49%)	H-11(B)→LUMO(B) (9%), H-9(B)→LUMO(B) (2%), H-1(B)→L+1(B) (5%)

No.	Energy (cm-1)	Wavelength (nm)	Osc. Strength	Symmetry	Major contribs	Minor contribs
**20**	24069.2	415.469	0.0559	2.610-A	HOMO(A)→L+3(A) (51%), HOMO(A)→L+4(A) (30%)	H-2(A)→L+9(A) (2%), H-2(B)→L+11(B) (2%), HOMO(B)→L+5(B) (2%)
